# General scales unlock AI evaluation with explanatory and predictive power

**DOI:** 10.1038/s41586-026-10303-2

**Published:** 2026-04-01

**Authors:** Lexin Zhou, Lorenzo Pacchiardi, Fernando Martínez-Plumed, Katherine M. Collins, Yael Moros-Daval, Seraphina Zhang, Qinlin Zhao, Yitian Huang, Luning Sun, Jonathan E. Prunty, Zongqian Li, Pablo Sánchez-García, Kexin Jiang-Chen, Pablo A. M. Casares, Jiyun Zu, John Burden, Behzad Mehrbakhsh, David Stillwell, Manuel Cebrian, Jindong Wang, Peter Henderson, Sherry Tongshuang Wu, Patrick C. Kyllonen, Lucy Cheke, Xing Xie, José Hernández-Orallo

**Affiliations:** 1 Princeton University, Princeton, NJ USA; 2 Leverhulme Centre for the Future of Intelligence, University of Cambridge, Cambridge, UK; 3 Microsoft Research Asia, Beijing, China; 4 Valencian Research Institute for Artificial Intelligence (VRAIN), Universitat Politècnica de València, València, Spain; 5 Department of Engineering, University of Cambridge, Cambridge, UK; 6 Department of Psychology, University of Cambridge, Cambridge, UK; 7 The Psychometrics Centre, University of Cambridge, Cambridge, UK; 8 Department of Theoretical and Applied Linguistics, University of Cambridge, Cambridge, UK; 9 KU Leuven, Leuven, Belgium; 10 Educational Testing Service, Princeton, NJ USA; 11 Center for Automation and Robotics (CAR), Spanish National Research Council (CSIC-UPM), Madrid, Spain; 12 William & Mary, Williamsburg, VA USA; 13 Carnegie Mellon University, Pittsburgh, PA USA

**Keywords:** Computer science, Human behaviour

## Abstract

Ensuring safe and effective use of artificial intelligence (AI) requires understanding and anticipating its performance on new tasks, from advanced scientific challenges to transformed workplace activities^1–3^. So far, benchmarking has guided progress in AI but has offered limited explanatory and predictive power for general-purpose AI systems^4–8^, attributed to limited transferability across specific tasks^9–11^. Here we introduce general scales for AI evaluation that elicit demand profiles explaining what capabilities common AI benchmarks truly measure, extract ability profiles quantifying the general strengths and limits of AI systems and robustly predict AI performance for new task instances. Our fully automated methodology builds on 18 rubrics, capturing a broad range of cognitive and intellectual demands, which place different task instances on the same general scales, illustrated on 15 large language models (LLMs) and 63 tasks. Both the demand and the ability profiles on these scales bring new insights such as construct validity through benchmark sensitivity and specificity and explain conflicting claims about whether AI has reasoning capabilities. Ultimately, high predictive power at the instance level becomes possible using the general scales, providing superior estimates over strong black-box baseline predictors, especially in out-of-distribution settings (new tasks and benchmarks). The scales, rubrics, battery, techniques and results presented here constitute a solid foundation for a science of AI evaluation, underpinning the reliable deployment of AI in the years ahead.

## Abstract (web summary)

A fully automated methodology based on rubrics capturing a broad range of cognitive and intellectual demands is illustrated using LLMs and tasks, demonstrating a new way to evaluate the capabilities of AI systems and anticipate their performance.

## Main

Present general-purpose AI systems, such as LLMs, are highly unreliable and unpredictable^6,12^. This places a large burden on AI evaluation in terms of explanatory and predictive power: we need to understand why the AI system is failing and anticipate where it can be applied successfully. The traditional performance-oriented evaluation approach has shown limited predictive power at the instance level, inside or outside the benchmark^9,10^. If DeepSeek-R1 achieves 79.8% average performance^13^ on a popular mathematical benchmark such as the American Invitational Mathematics Examination dataset^14^, we cannot make informed estimates of success on individual items sampled from that benchmark. This performance score is even less informative for out-of-distribution instances from other mathematical benchmarks, let alone benchmarks from other domains. Indeed, aggregate performance scores are a function of both the benchmark and the AI system, not invariable properties of the system only—its ‘capabilities’—that delineate the limits of the system, generalizable across a wide range of scenarios.

Instead of aggregating performance, other evaluation paradigms do estimate some properties of the subject (the human or the AI system), which, jointly with some properties of the item (the specific problem instance), can predict performance; we provide a glossary for technical terms such as subject, item, ability and contamination in Supplementary Information Section 1.16. Several techniques from psychometrics and other behavioural sciences have been applied to AI evaluation^15^, such as factor analysis^16,17^ and item response theory (IRT)^18^. However, the extracted factors or parameters are populational: they depend heavily on the population of systems and benchmarks used, which makes them quickly outdated with the fast pace of AI progress. More recently, score prediction metamodels related to uncertainty estimation and calibration methods, known as ‘assessors’^19,20^, have been used to anticipate performance for new tasks at the instance level, by means of latent features. Nonetheless, these features are difficult to interpret and typically extrapolate poorly out of distribution^21,22^. Alternatively, these features can be engineered by humans through cognitively inspired approaches^23^, but the scalability of this approach is limited by the need for experts who develop the cognitive models and annotate the testing items.

These perspectives differ in what is measured and how^8^, but they have all grappled with explanatory depth and predictive power. Also, most of these frameworks derive features, parameters or scales that are regularly saturated by an extremely volatile space of AI systems and benchmarks, soon becoming obsolete^24,25^. Lack of construct validity^10,26–28^ is also an issue in the common benchmarking paradigm^8^. Solving all of these issues is a prerequisite for more robust assessment in the real world^9,29^, such as interactive, subjective and adaptive evaluations^30–32^. Table 1 summarizes the problems and associated findings presented in this paper, the solutions it brings and its numerous new applications. Supplementary Information Section 1.1 further details related work.

**Table 1:** Diagnosis of the challenges of present AI evaluation paradigms, associated new findings revealed by the methodological solutions contributed in this paper and the potential applications of the new methodology (expanded in Methods section ‘Pipeline and guidelines for applications and extensions’)

Challenge	Finding	Solution	Applications
Construct validity: common benchmarks do not measure the abilities that they claim to measure	Narrow ranges of task demand levels on targeted abilities, confounded by unwanted demands	Benchmark profiles quantifying what abilities the benchmark truly measures	Resolution of contradictory claims (for example, LLMs can and can’t reason), better benchmarks with construct validity by design
Commensurability: incomparable measurements across benchmarks and distributions	Aggregate percentages mostly reflect sufficiency of capabilities, not capability estimates	Standardized general scales fixing demand levels to infer general capability profiles of AI	Interoperability of benchmarks, instance reuse into new batteries, meaningful scaling laws through non-saturated general scales
Population independence: frequent benchmark saturation and replacement dynamics	Instances in saturated benchmarks can still be informative, owing to uneven demand profiles	Non-populational benchmark demand and model ability profiles through standardized scales	Measurements robust to changing populations (of benchmarks and AI systems), capability catalogue accommodating AI progress
Explanatory power: benchmarks do not explain why LLMs fail on particular instances and what they lack	Failures monotonically increase in a sigmoidal way as demands increase	Defining general scales and rubrics that are interpretable by humans	Capability profiles bringing explanatory power, enabling model diagnosis and counterfactual explanations of AI failures across domains
Predictive power: poor anticipation of AI performance for new tasks and new domains	Capability-based instance-level predictions are highly accurate, robust to out-of-distribution cases	Predicting performance with demand levels as features, optionally with system profiles	Routing instances to the LLM with highest predicted probability or rejecting queries, monitoring AIs, guiding red teaming

We present a new methodology that can accompany, map and inform AI progress, regulation and deployment in the coming decades. This is instantiated and demonstrated for LLMs—the most popular form of general-purpose AI—but the methodology is extendable to AI systems with other architectures and affordances. The core element is an array of 18 scales in the range (0, *∞*) corresponding to general capabilities relevant to tasks expressed in natural language—such as verbal comprehension and logical reasoning—and broad areas of knowledge—such as natural and formal sciences. The precise values on these scales (the demand levels) are obtained through 18 carefully crafted demand-level-annotation (DeLeAn) rubrics in the range 0 to 5+, which humans can interpret and apply to any testing instance, but ultimately applied by a LLM judge for scalability.

By running the rubrics through a collection of 20 benchmarks, we obtain the annotated-demand-levels (ADeLe) battery, whose 18 histograms of demand levels form a demand profile examining the sensitivity of each benchmark (measuring what they claim to measure) and specificity (not measuring other capabilities beyond what they claim to measure). For each LLM on which ADeLe is applied, we get 18 characteristic curves, delineating LLM performance as a function of the demand levels. Each curve is summarized into an ability estimate that is commensurate to each demand scale, hence composing an ability profile of 18 ability levels. Notably, the demand levels for a particular task or benchmark and the ability levels for an AI system are independent of other benchmarks and systems and any population thereof. Most notably, the demand levels can be used to build strong predictive models for the success of AI systems on unseen in-distribution and, particularly, out-of-distribution instances (new tasks and benchmarks).

**Fig. 1:**
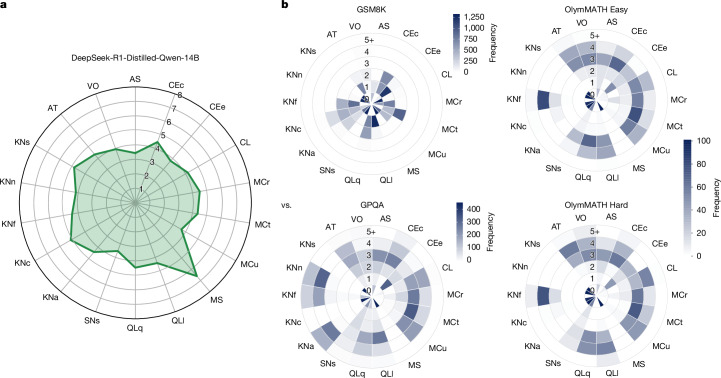
Commensurate LLM and benchmark profiles can be compared to explain and predict performance. Here we show LLM capability profiles (**a**; DeepSeek-R1-Distilled-Qwen-14B, estimated as discussed in the section titled ‘Explanatory power analysis: profiling LLM abilities’) and four different ‘reasoning’ benchmark demand profiles (**b**; GSM8K, OlymMATH Easy, GPQA and OlymMATH Hard, for which each slice represents the frequency of demand levels for each capability, with darker colours representing higher frequency). Researchers, developers and users can intuit that performance is expected to be high for the benchmark GSM8K but worse for the other three. Moreover, these profiles explain apparently contradicting findings, such as the accuracy of DeepSeek-R1-Distilled-Qwen-14B on GSM8K, OlymMATH Easy, GPQA and OlymMATH Hard being 90.50%, 61.80%, 59.10% and 13.30%, respectively, despite all of these benchmarks supposedly testing mathematical reasoning according to their creators. Indeed, OlymMATH Easy has lower demands for quantitative reasoning (QLq), logical reasoning (QLl) and inductive reasoning (CL) than OlymMATH Hard but similar demands for all other dimensions. Instead, GPQA yields worse performance than OlymMATH Easy, despite being easier in reasoning dimensions, because of its low specificity, with high demands for some knowledge dimensions beyond KNf (Formal Sciences), such as KNn (Natural Sciences) and KNa (Applied Sciences). Further details of these benchmarks associated with ‘reasoning’ benchmarks are discussed in Supplementary Information Section 1.12. Source data

As an example, by annotating several benchmarks that claim to evaluate ‘reasoning’ (Fig. 1) and comparing the annotated demands with the measured capabilities for an AI system, we can obtain causal explanation and prediction: if an AI system such as DeepSeek-R1-Distilled-Qwen-14B has a profile with quantitative reasoning (QLq), logical reasoning (QLl) and inductive reasoning (CL) abilities of 4.5, 4.3 and 4.2, respectively, as shown in Fig. 1a, we can anticipate success in a typical instance from GSM8K with 2, 1 and 0 demands in these same dimensions (and low on the others). We can also predict a less optimistic outcome on a typical instance from OlymMATH Hard, with values around 4 and even 5 for some dimensions (Fig. 1b). We can also perform counterfactual analyses, such as arguing that, if the capability of DeepSeek-R1-Distilled-Qwen-14B in QLq were reduced to 3, its performance on GSM8K would be marginally affected. However, it would be greatly affected if its capability in QLq were reduced to 1.

**Fig. 2:**
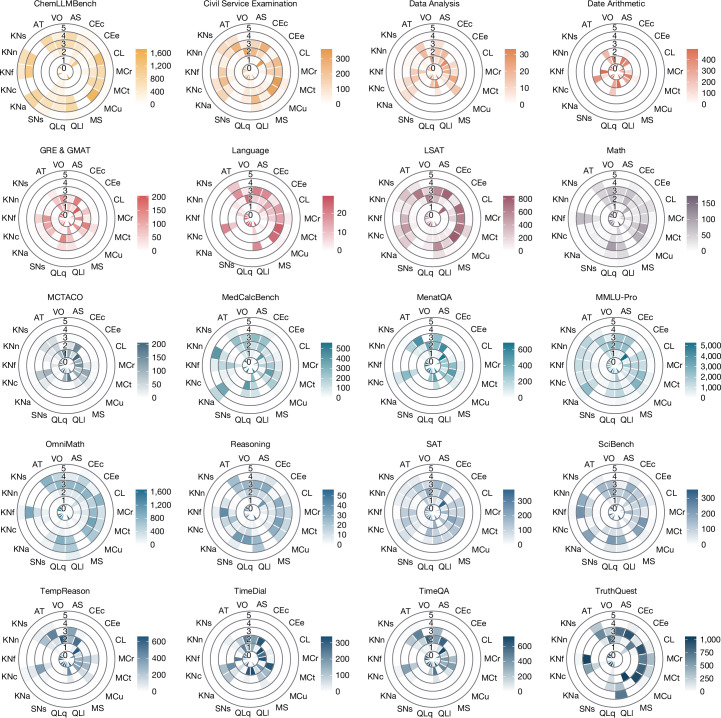
Distribution of level frequencies for the 18 demands (that is, demand profiles) of the 20 benchmarks in the ADeLe v1.0 battery. Supplementary Information Section 1.12 reconciles common myths in LLM ‘reasoning’ and also describes the demand profiles for 20 so-called ‘reasoning’ benchmarks. Source data

**Fig. 3:**
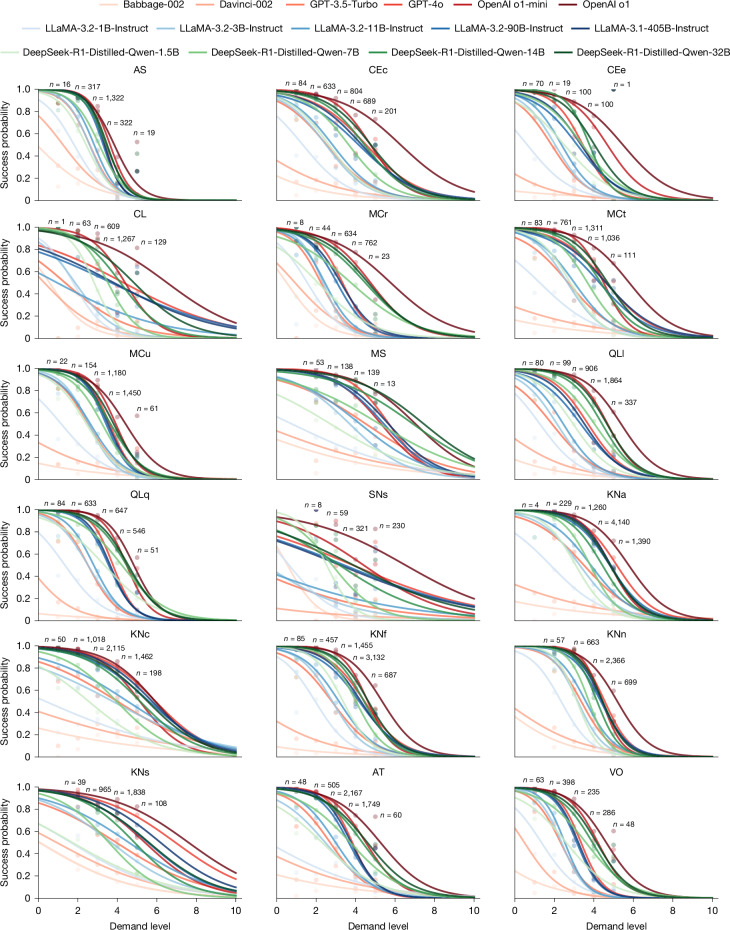
Characteristic curves for the 18 demands and the 15 LLMs. The *x*-axis shows the demand levels for that dimension and the *y*-axis the average performance (probability of success) for each level. We ensure all bins are weighted the same in the fit as the largest one (except those bins with less than 100 instances, which use a proportional weight for robustness). The curve is a logistic fit with an anchor at coordinates (20, 0), accounting for 50% of the total weight. The curves thus prolong beyond level 5 and this is why we show the *x*-axis from 0 to 10, even if the present version of the scales only has levels up to 5. Source data

**Fig. 4:**
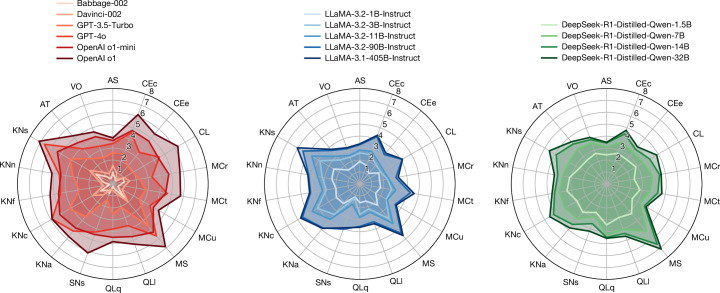
Ability profiles of the 15 LLMs. An ability of *l* means that there is 50% probability of the model to succeed on questions at demand level *l* (that is why some abilities go beyond 5). In contrast to radial plots usually shown for LLMs in the literature^47,48^, the values shown here are actual abilities on a ratio scale (0, *∞*) and the values (in expectation) are more robust to changes in the difficulty distribution of the benchmarks used. In Supplementary Information Section 1.4, we show clear scaling curves of model abilities as a function of number of parameters. Source data

**Extended Data Fig. 1:**
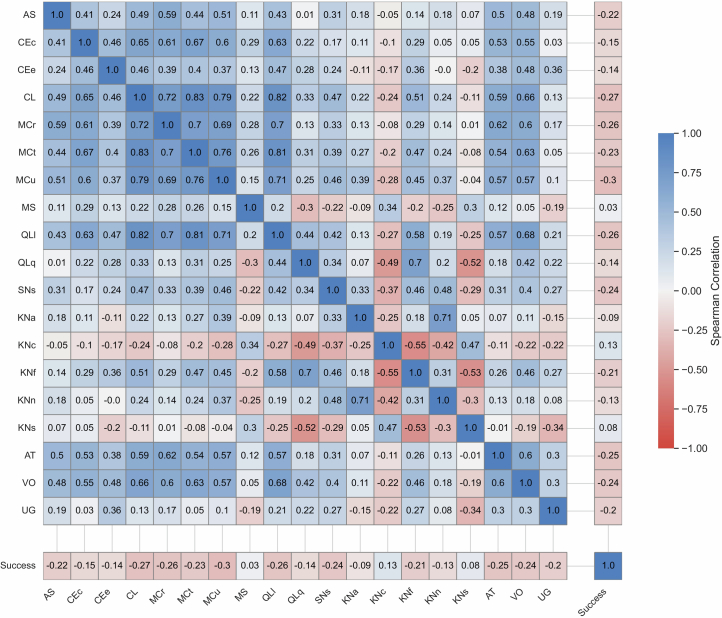
Correlations of the demand level using all of the items in the ADeLe battery for all pairs of the 18 demands and the special dimension UG (Unguessability). It also includes the success (that is, correctness at the instance level) of all of the subject LLMs considered in the experiments. Source data

Thus, with our methodology, we unlock the following possibilities, beyond the reach of previous approaches:

1. We can carve the space of capabilities into a hierarchical catalogue of general scales. The DeLeAn rubrics v1.0 (see Supplementary Information Section 2 for the dimensions in Extended Data Table 5) are applied systematically to the 16,108 instances of the ADeLe battery v1.0 (Supplementary Table 28), yielding 289,944 annotations across 18 general scales. The clarity of the rubrics is validated by the agreement between human and LLM annotations. The existence of instances that differ on any pair of capabilities and the moderate demand correlations between the 19 dimensions (Extended Data Fig. 1) suggest that the set of scales maps potentially distinctive capabilities, not dependent on present systems, likely remaining informative for future AI systems.
2. We can explain what common benchmarks truly measure. We discover the presence of demands in extraneous dimensions such as atypicality (from common to unique), volume (from small to large) and unguessability (from multiple-choice to open-ended), indicating contamination (overestimation because similar data were seen during training^33^), amalgamation (underestimation because examples are made more difficult by agglomerating more things to the task^34^) and funnelling (underestimation or overestimation by changing the difficulty of a task by reducing or increasing options or distractors^35^), respectively (Fig. 2 shows the levels of these demands and Supplementary Table 2 shows how predictive these dimensions are). Beyond these effects, many benchmarks lack either sensitivity or specificity: they do not contain instances of all demand levels for the dimensions their designers claimed to measure or they include non-zero demands on other dimensions they should not be measuring (Fig. 2). Identifying what each instance really measures paves the way for interoperability of benchmarks and AI evaluation with construct validity.
3. We can explain the general strengths and limits of AI systems through commensurate scales. In our experiments with three families of LLMs, we find that the ability scores at knowledge dimensions are mostly determined by model size, whereas quantitative and logical reasoning, learning and abstraction and (perhaps surprisingly) mind modelling and social capabilities are boosted in chain-of-thought, inference-heavy models such as OpenAI’s o1 and DeepSeek-R1-Distilled (Figs. 3 and 4). Because the dependent variable is not a relative percentage on a benchmark but a level on commensurate ratio scales that do not saturate, we have been able to clarify conflicting evaluation results (Supplementary Information Section 1.12) and demonstrate diminishing returns in scaling laws (Supplementary Information Section 1.4).
4. We can robustly predict AI performance for instances from new tasks and benchmarks. High predictive power at the instance level is possible, superior to black-box assessor baselines based on embeddings or fine-tuning, especially in out-of-distribution settings (new tasks and benchmarks), supporting both internal and external validity of the scales. These are also superior to domain-based^36^ or learning-levels taxonomies^37^ (Supplementary Information Section 1.9). This opens up a range of applications, such as better routing methods to choose what model to use^38^, safety operating areas in which assurance is guaranteed^7^ and anticipatory reject rules when harm or cost is anticipated^39,40^. See Extended Data Tables 2, 3 and 4 and Supplementary Fig. 8.

These processes are fully automated through open-source pipelines that can be easily customized by AI researchers, policymakers and regulators by extending the scales to other capabilities, traits or propensities (for example, affecting safety or fairness) and to agents with affordances (see Extended Data Fig. 5 and full explanation of the collaborative platform in Methods section ‘Pipeline and guidelines for applications and extensions’). This endeavour is seminal in creating a measurement standard for AI, mimicking the measurement efforts that have been pivotal in other sciences^41–43^.

The key element for our overhauling of AI evaluation is the configuration of scales that are understandable, general and well-grounded in measurement theory. We work with a catalogue of 18 scales, following a hierarchical structure (Supplementary Information Section 2), chosen by following a set of criteria fully explained in Methods section ‘General scales’. We refer to the first 11 as ‘elemental’, capturing general capabilities such as verbal expression and metacognition. The second group includes five ‘knowledge’ dimensions measuring the expertise in different broad areas of science. There are also three ‘extraneous’ dimensions (two are proper scales and the third is a control variable for funnelling), AT (Atypicality), VO (Volume) and UG (Unguessability), which do not directly capture cognitive demands but, rather, reflect those elements making items more difficult in other ways. The full scale rubrics can be found in Supplementary Information Section 2. We also explore alternative ablations with subsets of the catalogue as well as other taxonomies^36,37^ in Supplementary Information Sections 1.7 and 1.9, with none of them coming close to what the DeLeAn catalogue achieves in predictive or explanatory power.

In Methods sections ‘Ratio scales’ and ‘Dissecting the demand-ability space’, we explain how the scales are defined using rubrics that serve as measurement instruments for the instance demands and then build the methodology around them; this is applicable to whatever catalogue we use, be it DeLeAn v1.0, its extension or others. Our main goal with these scales is to achieve AI evaluation with both explanatory and predictive power. We now demonstrate that this is indeed the case with four specific research questions, comparing our approach with standard practice or best baselines in AI evaluation.

## Annotation scales distinguish levels and dimensions

First we address the following research question: can humans distinguish the levels in the rubrics and the dimensions? The scales will only serve for explanatory purposes if they can be understood. In Methods section ‘LLM annotators and inter-rater analysis’, we describe how a group of five humans were selected, how the rubrics were presented and to what sample of data. The inter-rater agreement (*r*_WG_ index) of these five humans for the 18 demands ranges between 0.70 and 0.91 (with an average of 0.83). After applying the Delphi method, we have a consensus annotation, which we compare against GPT-4o, the LLM annotator, resulting in high agreement rates (*r*_WG_ scores between 0.75 and 0.94, averaging 0.86). These agreement rates show common understanding between humans and with the automated annotations performed by GPT-4o. Another source of necessary support for a rubric would be whether it leads to high predictive power, which we will explore in the section ‘Predictive power analysis: anticipating performance with assessors’, while still representing the construct in an understandable way.

The dimensions could be understandable by humans but conceptually redundant, in the sense that we could not conceive an instance for which one dimension level is high and the other is low. If such an instance does not exist, humans will find it hard to distinguish the dimensions. The dimensions can still be correlated in a particular benchmark (for example, because the design or selection bias always makes one increase along with the other), but if the correlation is not near-maximal, we could conclude that there must be instances with very different levels. In Extended Data Fig. 1, we show the Spearman correlations of the demand levels for all of the dimensions in the ADeLe battery, a representative sample selected mainly from AI benchmarks in 2024. The generally low or moderate correlations indicate that most dimensions seem to carve different parts of the intelligence space, still allowing for cases in which the level for one dimension is low and the level for the other dimension is high. These examples do not abound but are not impossible. Only two correlations are greater than 0.8 and they fall on CL (Conceptualisation, Learning, and Abstraction), which looks slightly central in the manifold, given its strong correlation with MC (Metacognition and Critical Thinking) and with QLl (Quantitative and Logical Reasoning). We also see that the correlations for the extraneous dimensions are high with other demands (except for UG). In general, these positive or negative correlations can have several interpretations, as they are contingent to our choice of benchmarks.

The overall conclusion is that the annotations by GPT-4o seem understandable for humans across all dimensions, and the dimensions can be well distinguished. This is valuable, as other rubrics in AI evaluation practice tend to be specific, rarely quantitative and only occasionally meant to be explanatory^44,45^, despite the recognition that this understanding is a key factor in AI adoption^27^. Also, the correlations between dimensions do not seem to suggest that some combinations of demand levels are impossible, but simply infrequent in the present ADeLe battery v1.0. In this paper, our choice of instances and benchmarks was meant to be representative of the landscape of AI benchmarks, rather than a cherry-picked selection to minimize correlations. This was conditioned by our interest to explore what the benchmarks measure, as we study next.

## Explanatory power through benchmark demand profiles

The research question we address in this section is: what is the sensitivity and specificity of ADeLe and its constituent benchmarks? We can first look at the demand profiles per benchmark (Fig. 2). This is informative to understand what the benchmarks actually measure and whether they measure what their designers claim to measure.

Overall, the profiles are considerably distinct, so apparently they measure different things. Benchmarks that focus on specialized topics (for example, ChemLLMBench, OmniMath, MedCalcBench and SciBench) show high demands in their respective domains (KNa (Applied Sciences), KNn (Natural Sciences) and KNf (Formal Sciences)), whereas benchmarks such as TempReason and TruthQuest, which target a single domain, often peak in further dimensions. Other benchmarks—such as Date Arithmetic, GRE & GMAT, MCTACO, TimeDial and TimeQA—have uniformly low demands. By contrast, broader assessments such as Civil Service Examination, LSAT and MMLU-Pro show mixed profiles.

To determine whether they measure what they claim to measure, we must compare Fig. 2 with the list of capabilities or domains these benchmarks are said to be measuring (Supplementary Table 28). To better illustrate the issues of construct validity, we systematize sensitivity and specificity thresholds through two criteria:

- The sensitivity criterion: if a new benchmark claims to measure *X*, we should expect to see a wide distribution of levels for the demands related to *X* in that benchmark: we characterize this by having mean ≥ 2 and standard deviation (s.d.) ≥ 1.0 in dimension *X*.
- The specificity criterion: moreover, we should expect to see low levels for all dimensions that are not related to what the benchmark claims to measure: we characterize this with mean < 2.0 for all other ‘confounding’ dimensions.

Table 2 quantitatively shows a list of benchmarks and whether they meet these specificity and sensitivity criteria. In a few particular cases, there is some overlap between what a benchmark claims to measure and what capabilities it is sensitive to. However, this occurs for less than half of the capabilities that the benchmark claims to measure and does not happen for most benchmarks and dimensions (that is, aggregates have little sensitivity and specificity). For instance, benchmarks such as SAT are saturated for different reasons (low atypicality, that is, high contamination), whereas MedCalcBench actually measures whether the LLM has sufficient attention and scanning capability to process the given information, rather than purely measuring medical calculation capabilities. Further, in Supplementary Information Section 1.12, we reconcile common myths in LLM ‘reasoning’, observing the same issue of lacking either sensitivity or specificity for a batch of 20 ‘reasoning’ benchmarks.

**Table 2:** Sensitivity and specificity analysis of a subset of 20 benchmarks in ADeLe

Benchmark	Claims to measure (their terminology)	Claims to measure (our terminology)	Sensitive to…	Mean	s.d.	Accuracy
ChemLLMBench	• Generation of descriptions for molecules	CEc^•^, CEe˚, KNf^•^, KNn˚	AS˚	3.2	1.0	27.2
	• Generation of new molecules		CEc^•^	2.6	1.2	
	• Chemical name understanding		KNf^•^	4.0	1.2	
	• Chemical reaction products prediction		MCr˚	3.1	1.1	
	• Identification of target molecules		QLq˚	2.2	1.5	
			SNs˚	3.2	1.3	
Civil Service Examination	• Logical reasoning	QLI˚	KNa˚	2.1	1.8	73.5
			KNs˚	2.0	1.8	
Data Analysis	• Data analysis	CL˚, KNf˚, QLq˚	KNc˚	2.2	1.0	69.7
LSAT	• Analytical reasoning	CEc˚, CL˚, MC˚, QLI˚	KNc˚	2.9	1.1	81.6
	• Logical reasoning		KNs˚	2.1	1.8	
	• Reading comprehension					
MMLU-Pro	• Knowledge	KNa^•^, KNc˚, KNf˚, KNn˚, KNs˚, QL˚	KNa^•^	2.9	1.6	87.6
	• Reasoning					
Math	• Mathematics	CL˚, KNf˚, QLI^•^, QLq˚	AT˚	2.4	1.1	59.0
			MCu˚	2.4	1.0	
			QLI^•^	2.7	1.2	
MedCalcBench	• Medical calculation knowledge	KNf˚, KNn˚, QLq˚	AS˚	2.0	1.2	88.0
	• Patient attributes extraction					
	• Final results arithmetic					
MenatQA	• Event temporal reasoning	QL˚	KNc˚	2.8	1.0	72.8
OmniMath	• Mathematical reasoning at Olympiad level	CL^•^, KNf˚, QLI^•^, QLq˚	AS˚	2.3	1.3	34.4
			CL^•^	3.1	1.2	
			MCr˚	2.6	1.1	
			QLI^•^	3.4	1.0	
Reasoning	• Spatial Reasoning	QLI˚, SN˚	CL˚	2.6	1.1	48.2
	• Logical Reasoning		MCr˚	2.8	1.1	
SAT	• Critical thinking	MC˚	AT˚	2.2	1.1	98.3
	• Problem-solving					
	• Analytical skills					
SciBench	• Scientific problem-solving	KNa^•^, KNf˚, KNn^•^, KNs˚, MC˚	KNa^•^	3.0	1.6	83.7
			KNn^•^	2.8	1.7	
TempReason	• Event temporal reasoning	QL˚	KNc˚	2.8	1.1	71.2
TimeQA	• Event temporal reasoning	QL˚	KNc˚	2.4	1.1	89.0

Benchmarks are chosen such that at least one dimension that satisfies our two sensitivity criteria (s.d. ≥ 1 and mean ≥ 2). The second column shows what they claim to measure using their own terminology (sources are described in Supplementary Table 28). The third column shows the dimensions that the benchmarks claim to measure, expressed in our terminology. The fourth column shows what the benchmarks are actually measuring or sensitive to. For every dimension to which the benchmark is sensitive, we provide the mean and s.d. of the levels for that dimension (fifth and sixth columns, respectively). The seventh (last) column shows the average accuracy of GPT-4o per benchmark as a reference. A superscript ∘ in the third and fourth columns indicates missed dimension (lack of sensitivity) or extra dimension (lack of specificity), respectively. A superscript • indicates the dimensions that are claimed to be measured and are actually measured (high sensitivity). The other six benchmarks from the ADeLe battery that do not follow the two aforementioned sensitivity criteria are Date Arithmetic, GRE & GMAT, Language, MCTACO, TimeDial and TruthQuest (with the accuracies of GPT-4o being 98.9, 95.6, 72.4, 95.1, 98.8 and 43.0, respectively). Takeaway: no benchmarks have high sensitivity and specificity, indicating a clear lack of construct validity.

**Extended Data Fig. 2:**
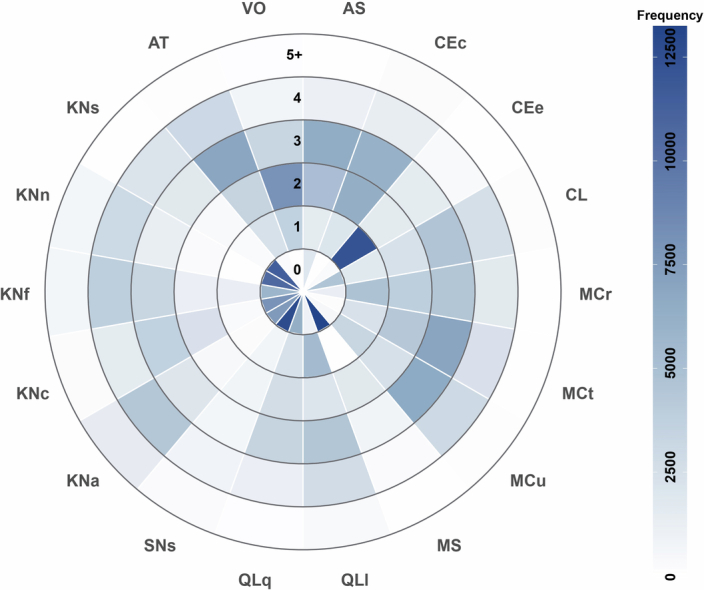
Distribution of level frequencies for the 18 demands using all of the 16,108 instances in the ADeLe battery v1.0. Dimensions such as CEe (Verbal Expression), MS (Mind Modelling and Social Cognition) and SNs (Spatial Reasoning and Navigation - Spatial) have low proportion of items of high level, but this is in accordance with the focus of LLM evaluation on factual questions with no navigation or full social interaction. Future versions of the battery for agents or multimodal scenarios can increase the number and breadth of the dimensions. Source data

Taking all of this into account, the specificity and sensitivity of common benchmarks are poor and variable. These results indicate that, by assigning one or more benchmarks to one ‘capability’ and aggregating their accuracy (as is the present standard practice), different demand levels and dimensions are averaged, leading to highly confounded results. If this is the baseline for common AI evaluation practice, it is simply insufficient to detect problems of specificity and sensitivity^10,27^. This issue becomes even more pronounced when integrating numerous benchmarks, such as BIG-bench^46^ and other mega-benchmarks. Even if sensitivity may be increased by this integration (as we see for the whole of ADeLe; Extended Data Fig. 2), specificity is lost if aggregate scores are used. Instead, with our scales, we can compare mixed subsets of items from different benchmarks whose demand levels now become commensurate, create recombinations of instances to test specific capabilities and systematically select or discard benchmarks altogether based on their profile quality, before even using them.

## Explanatory power through LLM ability profiles

Another research question about explanatory power moves the focus to the AI systems: can we understand the capabilities of models and their evolution in non-saturating plots? To answer this question, we selected 15 LLMs (Extended Data Table 1) and ran them on the ADeLe battery. As will be explained in more detail in Methods section ‘Subject characteristic curves’, we use a dominant slice procedure: for each demand level *l* along a dimension, we aggregate the results of only those task instances for which the demands in all remaining dimensions do not exceed *l*. We apply a logistic fit to these points, yielding 18 per-dimension characteristic curves that capture how model success rates decline with increasing demand (Fig. 3). For example, the curves of certain dimensions are steep and with low variability across models, such as AS (Attention and Scan) and MCu (Calibrating Knowns and Unknowns). They explain success very well for instances in the low range (success for demands between 1 and 2) and the high range (failure for demand 5 or higher). By contrast, curves of other dimensions are flatter, such as KNs (Knowledge of Social Sciences), in which the discrimination (between success and failure) is the lowest. Notably, several dimensions show particularly distinct behaviours. The characteristic curves for MCr (Identifying Relevant Information) and MS (Mind Modelling and Social Cognition) clearly distinguish the performance of reasoning models (whether distilled or not) from non-reasoning ones. All subject characteristic curves, in independent plots, can be found in Supplementary Information Section 1.14.

We use the area under the subject characteristic curve to estimate ability, as explained in Methods section ‘Subject characteristic curves’. Note that an ability of 4 does not mean that the model can solve all or most of the items at level 4; it actually means that it can solve half of those at exactly level 4 in expectation. Figure 4 shows the ability profiles of the 15 LLMs, arranged into families. It is now more evident that those dimensions related to knowledge are high for larger models and reduced for small and distilled models. The reasoning models (such as OpenAI’s o1 and DeepSeek-R1-Distilled) have clear improvements on the two kinds of QL (Quantitative and Logical Reasoning) but also on MCr (Identifying Relevant Information) and MS (Mind Modelling and Social Cognition) (even down to 7B in the distilled models).

Finally, the increase of model abilities based on the number of parameters seems to be marginal for the two largest LLMs in the LLaMA or DeepSeek-R1-Distilled-Qwen families; this is further confirmed in Supplementary Information Section 1.4, in which we introduce the very first scaling laws of the actual abilities of LLMs. The use of open ratio scales commensurate to the demand levels is in opposition to the traditional scaling laws using performance, which easily saturate close to 100% accuracy and fluctuate heavily depending on the demand-level distributions of the selected benchmarks. Aggregation, even if sliced by benchmarks, domains or some tags^46–48^, leads to values in each dimension that are not commensurate, hard to explain and volatile to the distribution of difficulties. For instance, 70% aggregate accuracy in all logical reasoning benchmarks does not mean more capability than 50% aggregate accuracy in all metacognition benchmarks, not even more capability than 50% aggregate accuracy in another set of logical reasoning benchmarks. Reflecting to what we saw in Fig. 1, a moderate increase in demands can be associated with a big drop in performance (as seen in the two versions of OlymMATH). Making differences commensurate is one of the advantages of having demands and capabilities in the same scale. By looking at standardized scales on several dimensions, we can explain many conflicting claims made in the literature, such as LLMs being considered capable of ‘complex reasoning’^49^ in 2022, to claims of LLMs ‘not capable of the non-trivial reasoning’^50^ 3 years later, which seems inconsistent with the substantial progress in chain-of-thought and reasoning models in the past few years. These contradictory statements about reasoning are explored and clarified with our scales in Supplementary Information Section 1.12.

In general, through our approach, we can investigate the capabilities of models and their evolution in a comprehensive and granular way, with characteristic curves explaining why each model succeeds or fails in different regions, depending on the demand profile of the instance. This explanation originates from the information collected from the AI system under observation only: unlike IRT and other latent variable approaches (factor analysis or principal component analysis) derived from the results of many systems and instances, the abilities and explanations we get for one LLM with our methodology are not affected by the results or the choice of the other 14 LLMs.

## Predictive power through assessors anticipating performance

The last research question is, can we predict AI performance on unseen instances, both in distribution and out of distribution? As shown in the bottom row of Extended Data Fig. 1, most dimensions are negatively correlated with success, suggesting that, in aggregate, higher demands tend to reduce performance. This is promising about their predictive power when used in a multivariate way. To quantify this predictive power precisely, we trained three types of instance-level probabilistic classifiers, known as assessors: a random forest (RF) that maps the 19-dimensional demand annotation vector directly into a predicted probability of success, another RF model that relies on precomputed GloVe embeddings extracted from the raw text of each question and a fine-tuned LLaMA model trained end-to-end on the question text to predict success. Further details are provided in Methods section ‘Assessors and metrics’.

In-distribution results (Extended Data Table 2) show that, despite the large imbalance in some of the subject models’ own accuracies (from 0.102 for Babbage-002 to 0.843 for OpenAI o1), the demand-based RF achieves high discrimination (between success and failure), as measured by the area under the receiver operating characteristic curve (AUROC), and near-perfect calibration, as quantified by the expected calibration error (ECE). In terms of discrimination, we see that the best result is achieved for GPT-4o (0.882 in AUROC), being the most predictable LLM for the three assessors, whereas small models are less predictable. Averaged across all 15 LLMs, the demand-based RF produces an accuracy-weighted average AUROC of approximately 0.84, which is on par with the performance of the fine-tuned LLaMA assessor, whereas its average ECE (0.01) is much lower than that of the other approaches (0.03 for the GloVe-based model and 0.04 for the fine-tuned LLaMA model). Calibration plots demonstrating these results are provided in Supplementary Information Section 1.5. This strong in-distribution performance supports the internal validity of the methodology.

For the analysis of external validity, we further evaluated predictive performance under out-of-distribution conditions, by withholding entire tasks (task out of distribution) or entire benchmarks (benchmark out of distribution) from training (Extended Data Tables 3 and 4, respectively). In the task out of distribution set-up, the predictive power of the demand-based assessor remains robust (weighted AUROC = 0.81, ECE = 0.02), only slightly lower than in-distribution, and outperforms the rest of the assessors, whose performance considerably decreases (achieving weighted AUROC values of 0.79 for the LLaMA-based and 0.74 for the GloVe-based assessors). In the more challenging benchmark out of distribution, the performance of the demand-based assessor decreases a slightly more (weighted AUROC = 0.75 and ECE = 0.04). By contrast, the predictive power of the other two assessors suffers a much greater decrease. This suggests that the demand-based predictor is less prone to overfitting with spurious features compared with its counterparts. In Supplementary Information Section 1.9, we also demonstrate that the predictive power of our demand-based assessor is superior to two domain-based and learning-levels taxonomies.

Although many traditional IRT methods can explain performance on seen items, they cannot be used to predict performance for new instances (except linear logistic test models; see Supplementary Information Section 1.1). IRT requires the item in question to be included in the pool of items that are used to extract the parameters and multidimensional IRT extracts the difficulty dimensions that matter for that pool only. In our case, any new instance, coming from any new benchmark or batch of examples, can be annotated automatically to obtain its vector of demands, which is independent of population, and from which we can predict performance.

Predictive power is paramount in a deployment setting in which the goal is to anticipate whether AI will perform well in unseen scenarios, rather than merely grading subjects in a testing environment. The natural baseline in AI evaluation practice is just average accuracy. This can extrapolate to an extent for system selection but, in the case of instance prediction, it leads to no discriminative power at all (an AUROC of 0.5) and calibration that is only good for in distribution. Uncertainty estimation from the LLM itself, on the other hand, requires running the model or, in many cases, a white-box or grey-box access, whose results are not better than external assessors^20^, as we have used here. Overall, the supremacy in predictive power observed for the demand-based assessors is clear. They are based on interpretable demands, in comparison with the two much larger and uninterpretable baselines. This is strongly encouraging, shedding light on a promising future for the reliable deployment of AI.

## Discussion

So far, AI evaluation is not meeting the needs of a fast-changing and increasingly diverse AI ecosystem. Understanding and anticipating performance has become an urgent requirement for many general-purpose AI systems. By building and exploiting absolute demand scales for annotating thousands of instances by means of automated rubrics, we have set a promising new direction for AI evaluation. The methodology we have presented and illustrated is comprehensive, scalable and standardizing, addressing many of the issues of conventional AI evaluation practice: a lack of explanatory and predictive power, as well as saturation and overfitting to specific populations of benchmarks and AI systems, respectively. With the pace and penetration of general-purpose AI, a rigorous, scalable and pipelined evaluation had been urgently demanded by researchers, companies, third-party evaluators, policymakers and regulators. It is paradoxical that powerful LLMs as annotators have made this new methodology possible and scalable. The explanatory value of LLM annotations has been independently validated by humans through inter-rater analysis and the Delphi method, and their predictive power stands through task diversity.

Nonetheless, our work is not without limitations. First, the DeLeAn v1.0 rubrics do not fully cover certain dimensions, such as navigation, and excludes capabilities in other modalities and paradigms in AI, such as multimodal systems and robotics, given that we limited our analysis to LLMs. We encourage other researchers to extend the set of rubrics to further dimensions (including propensities, values and other elements that are specifically conceived for safety or fairness and evaluate other kinds of AI systems with them). Second, there are very few high-quality level 5+ items in our present battery. Given the pace of progress in AI, the present scales (up to 5+) will need to be extended in a way that remains backward compatible with existing scales. Third, we could increase the predictive power in and, most importantly, out of distribution, especially if we introduce more benchmarks with ‘purer’ items only loaded on a few demands, and as LLMs improve as annotators. Fourth, we used LLM judges for grading model outputs, with excellent results compared with human graders. However, some more open-ended or agential tasks in the future may require more advanced automated grading.

Overall, the new methodology showcases the successful development of the construct-oriented paradigm in AI evaluation^8^, integrating perspectives from different disciplines. A streamlined collaborative platform (https://kinds-of-intelligence-cfi.github.io/ADELE/), and associated catalogue of rubrics, will grow in the years to come, ready to explain and predict the performance and safety of AI systems. In a moment when AI evaluation is at the crux of research and regulations, and the science of evaluation had not yet digested the pace of general-purpose AI, our work takes crucial steps to make AI evaluation fit for purpose.

## Methods

### General scales

For more than a century, psychology has introduced many constructs with explanatory and predictive power about human behaviour, from conscientiousness to metacognition. On the basis of experimental data and theories of human cognition, these constructs are usually organized into hierarchical taxonomies, such as the Cattell–Horn–Carroll structure of human cognitive abilities^51^ or the Big Five personality traits^52^. In principle, we could build a similar taxonomy for artificial cognition, based on theory and experiments about machine behaviour^4^. However, as the base population of machines is much more arbitrary and changing than those of humans, it makes more sense to devise a taxonomy that could encompass any kind of natural and artificial intelligence, by considering capabilities that are meaningful for more general theories of cognition^53^. Under this paradigm and by integrating and generalizing taxonomies from human psychology, comparative cognition and AI^53^, a general taxonomy of 14 capabilities was designed^54^ and later extended with corresponding 14 rubrics by Tolan et al.^55^ for the study of AI and human capabilities in the workplace. These rubrics assigned the presence or absence of the need for each capability in generic tasks extracted from worker surveys, occupational databases and AI benchmarks.

This taxonomy serves as a basis to construct a catalogue of capabilities following these four criteria: (1) the capabilities are general rather than specific, enabling the characterization of a wide range of tasks usually present in human activities; (2) the capabilities represent concepts that are understandable to humans (and LLMs), enabling their levels to be expressed through rubrics in plain natural language; (3) there is no a priori assumption of correlation or orthogonality in these capabilities as observed in humans or LLMs, to accommodate various present and future AI paradigms (rather than overfitting to a specific state of the art of AI); and (4) two capabilities are considered distinct as long as many tasks could conceivably require a high level of one but not the other. Following criteria (1) and (2), we use capabilities that are familiar in human and non-human cognition and AI practice (see Supplementary Information Section 1.1 for a coverage of taxonomies in humans and AI). Despite these inspirations, we follow (3) to ensure that the catalogue does not replicate human intelligence hierarchies or taxonomies derived from populational methods. But we do not look for a middle ground either: we do not assume that humans and AI systems share a common capability structure. Finally, ensuring (4), we consider two dimensions to be different (for example, metacognition and logical reasoning) if it is possible to conceive tasks that require one but not the other, independently of whether they are correlated in human or AI populations. Indeed, we include dimensions that may not be the most discriminative ones for the population of benchmarks or LLMs we use in this paper but can be useful to detect emergent properties in the future. This population independence is especially critical in the present era in which benchmarks and models get replaced every few months: for instance, for models without chain-of-thought, dominant until 2024, the set of reasoning capabilities we use may not have been very discriminative; however, with the advent of models with reinforcement learning and integrated chain-of-thought in 2025, reasoning capabilities become more informative. If our catalogue had not included them, we would have been unable to detect this shift, and the same applies for capabilities that may not be discriminative now but can be conceived of as different from others and may be informative in the future. As we may nevertheless miss some capabilities that will become relevant, the catalogue is expected to expand to include new dimensions in the future, provided they are understandable to humans.

As mentioned, our work builds on that of Tolan et al.^55^. First, we extend the taxonomy by including both knowledge and extraneous dimensions. Second, we develop new scales and rubrics in a quantitative range between 0 and 5+, with 0 representing absence of demand, values 1–4 representing increasing demand levels of the capability and 5+ representing 5 or above. For instance, the famous Sally–Anne false-belief task assesses understanding of an individual’s false belief about the properties of an object if those properties change while they are not looking (Sally will look for her marble in the basket where she left it, even though Anne moved it to the box when Sally was away). This may be level 4 for dimension MS (Mind Modelling and Social Cognition) but may be level 0 for dimension QLq (Quantitative Reasoning). Similarly, the question “if all A are B, some B are C, no C are D, and all D are E, what can be inferred about the relationship between A and E?” may be level 4 for QLl (Logical Reasoning) but level 0 for MS (Mind Modelling and Social Cognition).

Extended Data Table 5 shows the set of dimensions we have included in the first version of the DeLeAn rubric set (DeLeAn v1.0). We adapt seven broad capabilities from Tolan et al.^55^, applicable to LLMs (for example, ‘auditory processing’ was discarded), and refine a subset of them hierarchically with subdimensions, making them a group of 11 ‘proper’ cognitive capabilities that we call ‘elemental’; by ‘elemental’, we mean that these capabilities are not derived from others, as opposed to the knowledge dimensions, which are more acquisitive. These ‘elemental’ subdimensions were included after several rounds of discussions about whether some of the original seven broad subdimensions could be carved into finer, but still general, subdimensions that are conceptually distinct. Beyond the capabilities, we also include new dimensions accounting for domain ‘knowledge’, separated into five subdimensions (KNn, KNs, KNa, KNf, KNc) covering large branches of human knowledge, and three ‘extraneous’ ones, AT (Atypicality), VO (Volume) and UG (Unguessability), to account for elements that make the task more challenging independently of elemental capabilities or knowledge demands.

In particular, Atypicality deals with contamination^56,57^ and other familiarization effects leading to capability overestimation because similar data were seen during training. An AI system may simply succeed because it has memorized the instance. This dimension can be used to explain and predict performance, by identifying AT as a confounder with the other demands. The second extraneous dimension, Volume, represents the use of ‘collages’ to make instances more difficult. For instance, if we put ten simple additions in an exercise and we score whether all of them are correct, then we have increased the difficulty greatly, but the quantitative reasoning demand is the same. We call this phenomenon amalgamation and it is a recurrent trick to make instances more difficult, either in benchmarks of increasing hardness^46,58,59^ or in adversarial testing^60^. There is a correlation between the size of the questions (and the answers) and the difficulty you can achieve with it^46^ (Figs. 3 and 4). In the end, amalgamation produces an underestimation of the capabilities, because the subjects fail at tasks that are incorporating many simple things. The chances of error accumulate, even if the cognitive load is not necessarily increased^61,62^. Finally, Unguessability captures the very usual funnelling effect to make a question more amenable for scoring but, at the same time, reducing its difficulty. The obvious case is the use of multiple-choice questions, which have become predominant in most AI benchmarks, despite its issues^63^. Reducing or increasing the number of options has been a common practice to change the ‘difficulty’ of a task without modifying its cognitive demands^35^. In general, these three extraneous dimensions will account for an important proportion of the predictability in LLM success and including them helps clarify these confounding effects.

Although we have 19 dimensions in total, only the first 18 correspond to proper capability demands (11 elemental, five knowledge and two extraneous) that may be met by the subject or not, with Unguessability being a special extraneous dimension reflecting the funnelling in the item design (for example, multiple-choice questions). Because of that, it is the only dimension expressed between 0 (the correct answer is trivially determined by the question) and 100 (unguessable, that is, a good open-ended question). Each of the other 18 demand rubrics includes a general description of the construct to be annotated, followed by a description of each of the levels, from 0 to 5+, with three ‘anchor’ instances each. By following Supplementary Information Section 2, we can better understand the trade-offs in the construction of the rubrics.

It is important to highlight that the catalogue is not definitive and is meant to be extended in the future using the same criteria of dimensions being general and conceptually distinct. We use the term ‘catalogue’ instead of ‘taxonomy’ to better emphasize its non-definitive nature. This is also why we call the rubrics and battery DeLeAn v1.0 and ADeLe v1.0, respectively, with the vision of incorporating new capabilities and propensities in the future. This will also include considering safety, fairness and values^64,65^ and not only performance (correctness) as the variable to predict.

### Ratio scales

We deliberately design the demand scales as ‘ratio’ scales^66^, with an absolute zero(no demand) and differences that are comparable across the scale. In the social sciences, a common interest lies in understanding differences, as no human has zero capabilities, and an ‘interval’ scale with negative capabilities makes sense (as in IRT) or as percentiles of a normal distribution (as IQ scores). We argue that for AI, we should aim for the top level in Stevens’s topology of measurement^67^: the ratio scales. Ratio scales have all of the properties of the previous scales: intervals and differences are meaningful but so too are ratios. Given the flexibility with which we can regulate compute and time use in AI, it makes more sense to set an absolute zero (no compute) on the demands and build the scales in such a way that ratios are meaningful. We wish to say that instance *x*_*i*_ at level 6 doubles the demand of an instance *x*_*j*_ at level 3. Taking into account that we fit logistic functions, this can be understood in terms of the log odds of being correct halving when moving 2*x* in the scale and doubling when moving *x*/2 in the scale^68^.

For this first version of the scales, we decided to choose levels (0, 5) of the full range (0, *∞*) for practical reasons. With a single rubric, it is hard for humans and LLMs to refine beyond five ordinal values—this is why Likert scales are so popular. Note that the rubrics only show cases in an ordinal scale between 0 and 5 and the annotations are discrete, never generating non-integer values. This is convenient for avoiding the need of binning for the curves and the demand histograms, but the values become fully continuous when estimating the abilities. In any case, it is usual to consider originally ordinal scales as interval or ratio scales when the number of levels is 5 or more^69^. Indeed, the magnitudes between 0 and 5 should not be interpreted as a mere rank. The way the scale increases depends on what the demand represents, but the pace of increase, the actual scale, is chosen in such a way that all scales are commensurate. For instance, for knowledge dimensions (applied sciences, customary everyday knowledge, formal sciences, natural sciences and social sciences and humanities) we thought of levels corresponding roughly to elementary, middle, high, undergraduate and graduate education. By looking at the attainment rates of some statistical data of education level rates (for example, Organisation for Economic Co-operation and Development (OECD) data^70^) and the specialization of domains as the educational level increases, we noticed that the questions of level *l* were usually sufficiently advanced to have roughly one person in 10^*l*−1^ solving it correctly. Then we extend this criterion as a rule of thumb for all scales, although future work could perform a proper calibration and see that the base of each dimension corresponds with the correct proportions. By using the same base, we achieve ratio scale consistency and commensurate scales across dimensions. In general, an item is at level *l* if *l* is the highest number such that, in at least 95% of samples of *n* = 10^*l*^ individuals, there is at least one correct response. The levels we have defined are 0 (None), 1 (Very low), 2 (Low), 3 (Intermediate), 4 (High) and 5+ (Very high), with *n* going from 1 to 100,000.

We could have calibrated some dimensions using procedurally generated examples. For instance, in reasoning, we could have increased the components of reasoning processes^71^ to see whether the levels increase accordingly, but each of these ‘scales’ would have been incommensurate with each other and not sufficiently general.

**Extended Data Fig. 3:**
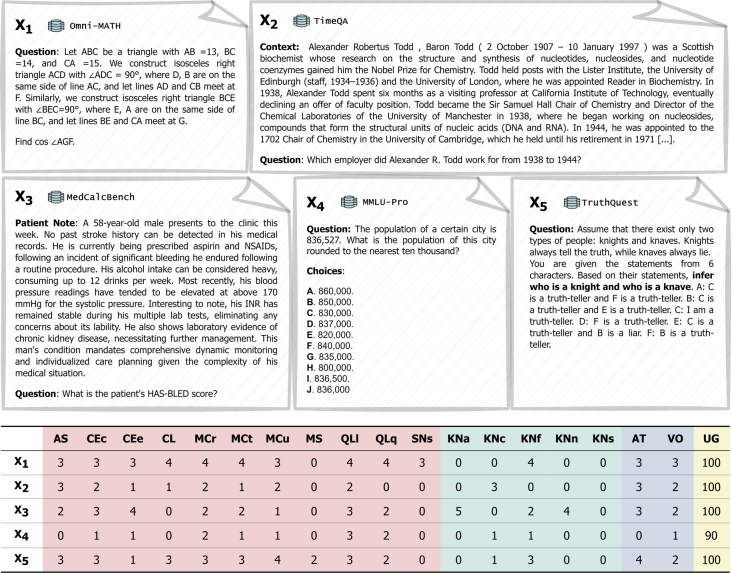
Level annotations of five items (from benchmarks OmniMath, TimeQA, MedCalcBench, MMLU-Pro and TruthQuest) using the DeLeAn rubric set by GPT-4o. The listed demands in the table (from left to right) follow the same order shown in Extended Data Table 5 (from top to bottom): 11 being elemental, five knowledge and three extraneous.

The 18 rubrics were crafted following the above criteria, using several iterations while testing with human and AI annotators. The final rubrics can be found in Supplementary Information Section 2. Once the rubrics were settled, we conducted the experiments, annotating tens of thousands of instances using a LLM, scalably and rapidly. Five annotation examples are illustrated in Extended Data Fig. 3.

### Dissecting the demand-ability space

Annotating instances using these general scales allows us to compare what makes them easy or hard and provides the same lens of analysis independently of where the instance comes from: human test, AI benchmark or new item design. We can discard or combine instances to build a specific test profile. Although this is not new in psychology or AI^72^, the scales can be applied to any task, test or collection of benchmarks; DeLeAn v1.0 is instantiated to consider only textual modality for now and to be extended in the future. By using the same scales in a standardized way, the comparison of the vast space of tests and benchmarks becomes possible for the first time.

For instance, in this paper, we applied DeLeAn to 16,108 instances from 63 tasks from 20 benchmarks, curated from the 2024 proceedings of six AI conferences and other venues, while ensuring both data quality and diversity (details in the section ‘Benchmark battery: instance selection and curation’). This is unprecedented, as all of these tasks are now represented within the same 19-dimensional space of 18 general cognitive demands (plus unguessability). After the annotation, these 16,108 instances constitute the ADeLe battery (Supplementary Table 28). We can observe the distribution of the demand levels for each dimension, the demand profile, represented as a polar histogram. Exploring this for each benchmark in ADeLe (Fig. 2) helps answer the question of whether each benchmark actually measures what their developers claimed to measure, as we explored in the main text.

Once instances are annotated, we can do more insightful analyses than just calculating one average for a whole dataset. When we run a LLM on an annotated benchmark such as the ADeLe battery, we can analyse each dimension separately using a subject characteristic curve^73^ to show the performance of an AI system as a function of demand levels, offering a comprehensive and robust delineation of the model’s ability on that dimension. The curve can be summarized using the area under the curve, referred to as the ability score, as described in the section ‘Subject characteristic curves’.

With this procedure on the characteristic curves, we can derive ability profiles as 18-dimensional vectors containing the estimated abilities. The usual way of representing a score profile with many dimensions is a radial plot. This is common in the behavioural sciences and more recently in AI as well. However, if we look at these plots in AI papers (for example, refs. ^47,48^), we see that what they represent in each dimension is the average accuracy of a selection of instances that belong to a particular domain or dataset, not an actual ability. The plots based on performance scores will change as the difficulty of the selected instances varies, whereas an ability profile is invariant to these changes. Overall, our notion of ability using the general scales is very different from the common yet inaccurate use of the term in AI as a synonym of performance. This includes the use of the term ‘capability’ in the area of safety evaluations: even if informally the concept may be associated with levels^74^, these levels were never defined or scaled.

By comparing the ability profile of an AI system with the demand profile of a task instance or a benchmark, we can explain the observed performance. Moreover, using the differences between abilities and demands, we can use interpretable algebraic models to anticipate performance for new instances (Supplementary Information Section 1.7.7). Notably, there is potential for other options as well. For example, the 18 values that are annotated for each single instance on the scale 0 to 5+ and unguessability constitute a 19-dimensional vector **x**, which can be used as predictor variables for a probabilistic classification model, an assessor, outputting the (estimated) performance of an AI system on that instance. Each assessor can be trained specifically for each LLM, without relying on the features of the LLM. As shown in the main text, we can compare this with many other powerful ways of predicting performance, such as assessors with embeddings and fine-tuned LLMs (there are more details on how we build distinct assessors in the section ‘Assessors and metrics’). Notably, despite the much smaller computation cost (apart from annotating the battery, which only needs to be done once), the predictive power is substantially better for the demands-based assessor than the best baseline, especially out of distribution, and evidently much better than average accuracy, which is only well-calibrated in distribution. This is because our general scales provide predictive features over a wide variety of tasks while limiting overfitting on features that become spurious when switching tasks and benchmarks. Finally, just as ability profiles are non-populational, the assessors we derive for each system are inferred exclusively from the results of that system, rather than from population-level parameters such as those used in scaling laws for aggregate performance prediction^75^.

### LLM annotators and inter-rater analysis

With the rubric set in hand, we annotate any new instance along each dimension using a LLM to replace human annotations, to scalably and rapidly annotate thousands of items. Although there may be some discordances between LLM and human scores, scalability is critical for widespread deployment of the new evaluation methodology. This can be seen as a trade-off but also as an opportunity to have stable and fully reproducible annotations using LLMs, which can be improved as LLMs get better or are more aligned with human interpretation. In fact, the three instance anchors per level were very instrumental for the LLMs to perform good ratings (in a few-shot inference fashion) but also for human understanding. In our case, we performed the annotations with GPT-4o, with which we found high agreement rate. The use of comprehensive rubrics in natural language that can be applied automatically is a substantial advancement in making the explanatory power of the scale a reality, especially if humans could interact with the LLM to explain their annotations.

Specifically, we prompt GPT-4o (‘gpt-4o-0513’ checkpoint)^76^ to annotate task demands levels (on a discrete scale from 0 to 5) instance by instance for all individual rubrics (see DeLeAn Rubric Set v1.0 in Supplementary Information Section 2). We use the Azure AI application programming interface (API) with chain-of-thought prompting (Supplementary Table 23) at temperature set to 0 with a maximum output token length of 1,000, to ensure that answers can be long enough for nearly all instances while substantially reducing the cost. The stopping condition and the rest of the parameters are left by default.

To assess the agreement rate between humans and GPT-4o, for each demand, we randomly sampled 50 instances while ensuring each level had at least a sample size of 3 to avoid minority levels getting neglected in our inter-rater analysis. This led to 900 instances to be annotated, which were distributed to five humans (authors of this paper, corresponding to Y.H., Y.M.-D., L.Z., Q.Z. and S.Z.), for which each instance was annotated by exactly three humans. The annotation process consisted of two steps. First, each annotator independently assigned a difficulty level (using the 0 to 5+ scale) to each instance using the rubrics. Next, the annotators met for a Delphi^77^ consensus meeting. During this meeting, instances for which the minimum and maximum ratings of the three annotators differed by two or more points were discussed in detail until a consensus was reached. For cases with differences of less than two points, a simple majority vote determined the final annotation. To check the inter-rater agreement rates, we use the *r*_WG_ index^78,79^ with the default rectangular null distribution; a score greater than 0.7 is generally considered as a good agreement rate.

The result is shown in Supplementary Table 22, in which we observe satisfactory *r*_WG_ scores (average = 0.86) between Delphi consensus and GPT-4o, consistently greater than 0.80, except for one dimension with a score of 0.75. However, the *r*_WG_ scores between humans before the Delphi consensus meeting were slightly lower for certain dimensions. These initial disagreements are because of several reasons, identified during our Delphi consensus meetings: occasional misinterpretations of certain words or terminologies, mainly for those humans whose primary language for daily use is not English; knowledge gaps in annotating certain particularly challenging task instances beyond the expertise of annotators; cultural variations affecting annotations, especially within some knowledge dimensions; and several inconsistent ratings for which annotators could not explain their own numerical assignments in hindsight, possibly caused by tiredness in annotating a large amount of instances; the reported time in annotating 50 instances on only one single rubric usually ranges between 30 and 60 min. The Delphi method proved useful to mitigate the individual biases and inconsistencies from human annotations caused by the miscellaneous reasons listed above, among others.

In Supplementary Information Section 1.8, we also explore two alternative LLM annotators. One is DeepSeek-V3, which is similarly powerful but open-weight: keeping all other things equal, it exhibits a similarly high agreement rate with the Delphi consensus (an average *r*_WG_ of 0.83; slightly worse than that for GPT-4o of 0.86) and it unlocks similarly high predictive power, comparing with the section ‘Predictive power analysis: anticipating performance with assessors’. The other LLM is LLaMA-3.1-8B-Instruct, which is open-source but much smaller. We find that it achieves a reasonably good agreement rate with the Delphi consensus (an average *r*_WG_ of 0.74; noticeably worse than that for GPT-4o of 0.86) and it exhibits moderately worse predictive power, comparing with the section ‘Predictive power analysis: anticipating performance with assessors’. This is to be expected, as older and smaller models are relatively less powerful in terms of obtaining reliable annotations.

Looking to the future, despite good agreement between humans and GPT-4o as annotator, higher agreements may be possible as the capabilities of LLMs progress, including their potential for explaining their annotations to humans.

### Benchmark battery: instance selection and curation

We constructed our benchmark battery by reviewing papers published in the 2024 proceedings from top-tier machine learning conferences (ICML, NeurIPS, ICLR) and natural language processing venues (ACL, EMNLP, NAACL). In our search, we first identified papers with ‘bench’ in the title and then supplemented the collection with further benchmark sets found at other reputable venues. Before including any benchmark (or subset thereof), we applied a rigorous quality check to ensure that the source meets the following selection criteria:

- The benchmark set must be sufficiently difficult to avoid an overabundance of trivial instances. A benchmark is discarded if state-of-the-art LLMs such as GPT-4 achieve more than 75% overall accuracy.
- The expected outputs must be amenable to automatic verification by LLM-based graders. Tasks requiring lengthy passages or those with several valid answers are excluded to maintain grading reliability.
- Benchmarks must not contain AI-generated content, when explicitly noted in the source paper.
- Tasks must be formulated as either open-ended or multiple-choice questions with at least four options to minimize the effect of stochastic ‘guessing’.
- Licensing requirements for the selected benchmarks shall be compatible and allow for free redistributions.
- The collection of benchmark(s) introduced by a paper must be publicly available at the time of our curation effort (that is, as of 26 December 2024).
- The task must have an objective ground truth that can be used to unambiguously categorize performance as either success or failure.
- The quality of ground-truth labelling must be near-perfect, if reported. For those benchmarks that do not report any quality scores of their ground truth, we apply further quality filters, described both at the end of this subsection and in the section ‘Subject LLMs and grading’.

This eventually resulted in a total of 20 benchmarks from nine papers, comprising 63 tasks for our analysis (Supplementary Table 28). For efficiency reasons, we randomly sampled up to 500 instances per task to strike a balance between data diversity and size. This led to an original battery of 21,996 instances.

Last, we prompted GPT-4o to annotate three quality indicators: (1) the accuracy of ground-truth labels; (2) the objectivity; and (3) the unambiguity, for all instances, graded with a Likert scale from 1 to 5 (Supplementary Tables 24, 25 and 26). We inspected the annotations of 50 randomly sampled instances with a score of 1 for each quality indicator, in which a human judge (a researcher with a background in computer science) reviewed these annotations and labelled them as ‘agree’, ‘disagree’ and ‘uncertain’. For the accuracy of ground-truth labels, the agreement, disagreement and uncertainty rates were 32%, 6% and 62%, respectively. For objectivity, the agreement, disagreement and uncertainty rates were 68%, 10% and 22%, respectively. For unambiguity, the agreement, disagreement and uncertainty rates were 70%, 22% and 8%, respectively.

Given this observation, we removed those instances with a score of 1 in any of the three aforementioned indicators, which accounts for 16% of instances in the initial battery, reducing the battery at this stage to 18,462 instances. Also, we discarded 0.9% of instances in which the LLM annotator did not offer an annotation (for example, flagged by OpenAI’s moderation filters) or did not yield demand annotations in an expected and easily processable format, resulting in 18,291 instances remaining.

This is a satisfactory result, as we removed many problematic instances at the cost of eliminating a small proportion of seemingly good ones. This cleaning is critical to reduce noise when deriving the ability profiles of models and evaluating the predictive power of assessors.

### Subject LLMs and grading

The pool of analysed subjects includes 15 LLMs in total (Extended Data Table 1), six proprietary models from OpenAI, five open-weight models from Meta and four open-weight models from DeepSeek:

- GPT/o1: we use six models from the GPT and o1 families (OpenAI)^80,81^. The four GPT models, Babbage-002, Davinci-002, GPT-3.5-Turbo (built as ‘gpt-35-turbo-0613’) and GPT-4o (built as ‘gpt-4o-0513’), are the original instruction-tuned models in the GPT family, in which the last two are also shaped up by fine-tuning with human feedback and further include a moderation post-filtering mechanism^82^. By contrast, OpenAI o1-mini (built as ‘o1-mini-2024-09-12’) and OpenAI o1 (built as ‘o1-2024-12-17’ with the reasoning effort parameter set to ‘low’) belong to a family of ‘reasoning’ models, designed to take extra time to generate and refine a chain-of-thought before producing a final answer. All of these models were accessed through the public API offered by Azure AI Foundry.
- LLaMA: we use five different scales of the latest LLaMA saga (LLaMA-3 family^83^): 1B, 3B, 11B, 90B and 405B, all of which have been instruction-tuned. Note that we refer to them consistently with the suffix ‘-Instruct’ as in the original names of the 1B, 3B and 405B variants. This also applies to the 11B and 90B variants, although they are originally named with the suffix ‘-Vision’ instead of ‘-Instruct’, as these are multimodal. To avoid any possible confusion, we replace the suffix ‘-Vision’ with ‘-Instruct’, as we focus on evaluating text modality in this work. All of the inferences were run through the Hugging Face API.
- DeepSeek: we locally run the four different scales (1.5B, 7B, 14B and 32B) of the DeepSeek-R1-Distilled-Qwen suite^13^, a set of ‘reasoning’ models (based on the Qwen-2.5 model family^84^) that distilled knowledge from a much more powerful LLM (DeepSeek-R1).

For inference, all subject models were queried with the temperature parameter set to 0 and no system prompt, with the exceptions of OpenAI’s o1 models, which can only be queried with temperature equal to 1, and the DeepSeek-R1-Distilled-Qwen models, which were queried with a temperature of 0.6 and a top-p of 0.95 as recommended by the original paper^13^. Similarly, we use chain-of-thought prompting for all models except for the ‘reasoning’ models (OpenAI’s o1 models and DeepSeek-R1-Distilled-Qwen models), which were already shaped up to perform chain-of-thought by default by their developers. In terms of maximum output token length, we use 2,000 tokens for all models, except for OpenAI’s o1 models and the DeepSeek-R1-Distilled models, which use 16,384 tokens instead. We used the default values for the stopping condition and the rest of the parameters.

Most grading of instances in present AI evaluation practice is performed with LLMs as a judge^85^, because manual grading for a large number of instances and models would be infeasible. We follow that practice but we do not want to consider instances that are wrongly graded, because that would portray a misleading account of the explanatory and predictive power of the methodology we present in this paper. We then prefer to discard those instances for which the LLMs (as a judge) are not robust. This means that we exclude some instances and this may introduce some bias selection in ADeLe. We believe instances that are hard to grade or verify do not necessarily mean that they are easier or harder to solve. In either extreme, they would increase predictability but not the separability metrics such as AUROC. Consequently, we perform the following procedure. We automatically grade the responses of these models on a discrete scale between 1 (surely incorrect) and 5 to (surely correct) using two LLMs, GPT-4o and Claude 3.5 Sonnet (‘claude-3.5-sonnet-1022’ checkpoint), prompted with temperature set to 0 while the rest follows the default configurations. The prompt contains both the input, the response of the subject and the ground truth (Supplementary Table 27) for a sample prompt template. To spot instances that are ‘hard to verify’ (for example, owing to inherent subjectivity or erroneous ground truth), which can introduce noise into the analysis, we remove approximately 12% of instances in which both LLM graders did not agree through simultaneously outputting either correctness scores ≥4 (both graders think the answer is a success with some confidence) or correctness scores ≤2 (both graders think the answer is a failure with some confidence) when verifying GPT-4o as a subject; this forms the final ADeLe battery v1.0, with 16,108 instances. We finally labelled input–output pairs graded with a mean score less than 3 as failure pairs and success otherwise (scores of 3 were filtered in the previous step anyway). We randomly sampled 100 instances from all of the gradings and manually found that 98% of input–output pairs are correctly verified.

### Assessors and metrics

An assessor is an external metamodel designed to predict the performance of a subject system (for example, a LLM) on individual task instances by taking features of those individual task instances as input^19,21,22,39^. These features can range from the raw representation (full text or image) to metafeatures representing cognitive demands and linguistic characteristics, as well as more structured representations such as average (word) embeddings of each task instance. When performance is defined as a binary success score (correct versus incorrect), an assessor can be built by using any standard binary classifier, including statistical models (for example, RF) and fine-tuned language models (for example, fine-tuned LLaMA-3.1-8B). Such models are trained to anticipate the success probabilities of a given subject on task instances without executing that subject and can be either tailored to predict the performance of a single AI system or designed to generalize across systems. In this work, we train and compare three types of assessor:

- Demand-based: this assessor is a RF^86^ classifier that takes the vector of 18 demands and the special UG (Unguessability) dimension as input to predict a subject LLM’s performance. The in-distribution data are used to optimally select the minimum number of samples required to split an internal node, chosen as 2, 50 or 200.
- Embeddings-based: in this model, each item instance is represented by the average of its GloVe word embeddings^87^, fed to train a RF classifier. As with the demand-based assessor, we tuned the minimum-samples-per-split hyperparameter of the RF (choosing from 2, 50 and 200) using the in-distribution data.
- Fine-tuned LLaMA: this is a fine-tuned LLaMA-3.1-8B (ref. ^83^) with a linear classification head. This model is trained end-to-end using the original input text for each task instance. We use the in-distribution data to select the optimal learning rate between 1 × 10^−4^ and 2 × 10^−^^5^. To improve training efficiency, we used the NF4 quantization scheme and bfloat16 for computation, along with low-rank adaptation (LoRA) for efficient training. Training was performed with a batch size of 16 for three epochs and a weight decay of 0.01.

For implementation, the RF models were trained using the scikit-learn library^88^, whereas the fine-tuned LLaMA-3.1-8B was trained on the Transformers library^89^ using the PyTorch backend running on Python 3.11. All unspecified hyperparameters were left at their default values.

In terms of computational cost, the on-demand assessor was extremely efficient. On an M3 Pro CPU, the data of each subject were processed by means of tenfold cross-validation in about 4 s. By contrast, the embedding-based assessor took about 40 times as long owing to the higher computational overhead of processing dense vector representations. The fine-tuned LLaMA assessor was by far the most expensive, taking around 300 GPU hours on a single V100 GPU to converge (that is, around six orders of magnitude longer than the demand-based approach).

To quantify the predictive quality of these assessors, we used AUROC and the ECE with ten equal-width bins, as these two metrics capture two key aspects of predictive power (discrimination and calibration) and each of them is commensurate when comparing the predictive power of distinct assessor–subject pairs.

We compute the statistical significance between the demand-based assessor and the strongest baseline. We apply the Wilcoxon signed-rank test based on the win–loss outcomes using paired comparisons of each fold between two assessors (across ten folds with ten repetitions each based on distinct seeds).

Although the use of demand annotations substantially outperforms the other baseline approaches as seen in Extended Data Tables 2, 3 and 4, two key factors explain why the discrimination power declines in out-of-distribution settings. First, because our analysis includes only 63 tasks from 20 benchmarks—many of which (for example, ChemLLMBench) have non-overlapping demand distributions— the training data do not fully capture the multidimensional demand space. We suggest that the predictive power of the demand-based assessor for any arbitrary new tasks or new benchmarks can be boosted to the level of in-distribution by ensuring that the demand distribution of the training data efficiently covers the multivariate demand space.

Second, there is a paucity of extremely difficult instances to challenge the high-performance models (for example, OpenAI o1-mini, OpenAI o1, DeepSeek-R1-Distilled-Qwen-32B). As shown in Fig. 3, even at level 5 (for which instance coverage is low), the best models maintain success probabilities well above zero and the estimated abilities can go beyond 5, just by extrapolation. In Supplementary Information Section 1.6, we further discuss these factors and potential improvements on instance selection and automated grading.

### Subject characteristic curves

**Extended Data Fig. 4:**
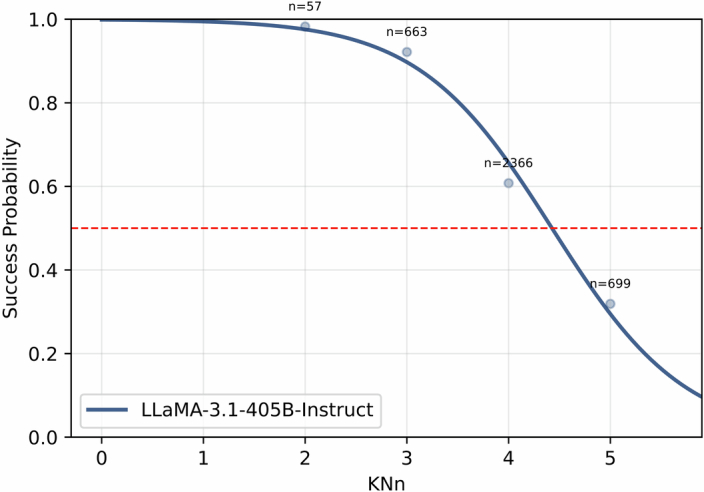
The characteristic curve of Llama-3.1-405B-Instruct for dimension KNn (Knowledge of Natural Sciences) on the ADeLe battery. The *x*-axis shows the demand levels from 0 to 5 for KNn and the *y*-axis is the average performance for that level (probability of success). As usual, level 0 has no points left (0 never dominates), but in this case, we see a situation with no point for 1 either. The curve is a logistic fit in the output range (0, 1). Source data

Extended Data Fig. 4 shows a subject characteristic curve for the results of Llama-3.1-405B-Instruct on 16,108 instances of the ADeLe battery, sorted and binned by the levels on the dimension KNn (Knowledge of Natural Sciences). As further elaborated in Supplementary Information Section 1.2, for each bin *b* for that dimension, we exclude all points for which the level of any other dimension is greater. In other words, we want the represented dimension to dominate on the instances we are showing (in this case, only 3,785 out of 16,108).

On this plot, we can then fit a logistic function and look for the *x*-axis value at which the probability of the subject to succeed is 0.5. In Extended Data Fig. 4, this leads to an estimated ability of 4.3. Ability can then be interpreted as the level of demand at which the probability of the subject to succeed is 0.5, assuming all other demand levels are lower, which is in accordance with psychometric tradition (ref. ^90^, p. 249) and will be followed for the rest of the paper. Note that an ability of 4.3 does not necessarily mean that the subject solves all tasks instances of level 4.3 or less but that it has 50% chance of succeeding at level 4.3, higher at level 3, much higher at level 2 and so on, and evidently lower at level 5 and above, in a sigmoidal way, as we see in the figure. The exact estimation of the ability (as the usually equivalent area under the curve) is further explained in Supplementary Information Section 1.2.

The advantages of these curves and this manner of interpreting ability are reinforced by the fact that the scale on the *x*-axis is absolute rather than relative. It is robust to changes of demand distribution in the data. For instance, with the 3,786 instances in Extended Data Fig. 4, we get an average accuracy of 62%. However, if we chose the *n* = 699 instances of level 5 and repeated them 500 times in the dataset, the average accuracy of the LLM would decrease substantially (below 40%), as we would be adding more difficult examples. This is what adversarial testing does^60^, especially when benchmarks saturate. By contrast, the average accuracy for the instances at bin 5 would remain the same and the characteristic curve would not be affected at all. The ability would not alter, remaining at level 4.3. This case neatly represents the difference between performance, which is a measure of a pair subject and task distribution (so changing from 62% to 40% when the task distribution changes), and ability, which is an inferred property of a subject that is invariant to the task distribution. Although all of this is strongly inspired by IRT, and the linear logistic test model in particular^91^, it is important to clarify that, unlike these and other latent factors approaches—those in AI included^16,17,75^—we only use the information of a single LLM for the estimation of its abilities.

With the demand-based scales and the ability-estimation method introduced in this paper, the demands and abilities for tasks and AI systems get values that are completely independent of other tasks and AI systems, now or in the future. We have used the term ‘non-populational’ to refer to an indicator or measurement that does not depend on the rest of the population, only on the individual. For the first time, there is a non-populational measurement paradigm for evaluating the cognitive and intellectual capabilities of general-purpose systems. This is in contrast to common non-inferential techniques, such as benchmark aggregates, which are affected by the distribution of difficulty in the benchmark. Similarly, standard inferential techniques such as IRT, principal component analysis and factor analysis are also populational. They usually work well with human populations because samples are sufficiently stable over time but lead to different results as soon as the AI system ‘population’ is modified, whenever a new set of LLMs is added to the inferential pool. For instance, the factors that were discovered for LLMs in ref. ^16^ differ from those found in ref. ^17^, even if the two studies collected representative samples of LLMs, used the same factor analysis methodology and took place in only a few months time difference. This volatility does not happen with our approach. Our abilities are not relative to a population of subjects and the scale is absolute. Even if the evaluation battery were extended with instances of levels 7 or 8 to account for more powerful future AI systems, the logistic curve for the old systems would probably have low values on these instances, thus not affecting the original estimates. This forward-looking extensibility and backward compatibility is crucial for measurement. In sum, there is an open opportunity for the new scales, battery and procedure presented in this paper to be the genesis of a standardization initiative for the robust measurement of present and future AI capabilities.

### Pipeline and guidelines for applications and extensions

There is a consensus within the AI community that there is a need for a new science of AI evaluation^92,93^. However, there is also resistance to moving beyond the present benchmarking paradigm^8^. Although some have proposed using the potential of the behavioural sciences, such as psychology and psychometrics, for AI evaluation, this is generally understood to mean populational approaches, such as factor analysis, principal component analysis or IRT^16–18^, whose findings may soon lose value owing to the fast-evolving set of AI systems. Our paper demonstrates that a possible answer for a scientific approach to AI evaluation comes from behavioural inference at the instance level. These inferences are made from features that are not derived from a population of subjects. This approach was not previously possible for human evaluation because it requires tens of thousands of instance-level results for each subject—yet this scale is possible for AI evaluation. Furthermore, the annotation of this number of items with a wide range of dimensions is only unlocked now by the ability to automate good-quality annotation with LLMs. Nevertheless, to move beyond the present paradigm (based on benchmark aggregates or the use of latent factors), the methodology must be made accessible, modular and customizable.

**Extended Data Fig. 5:**
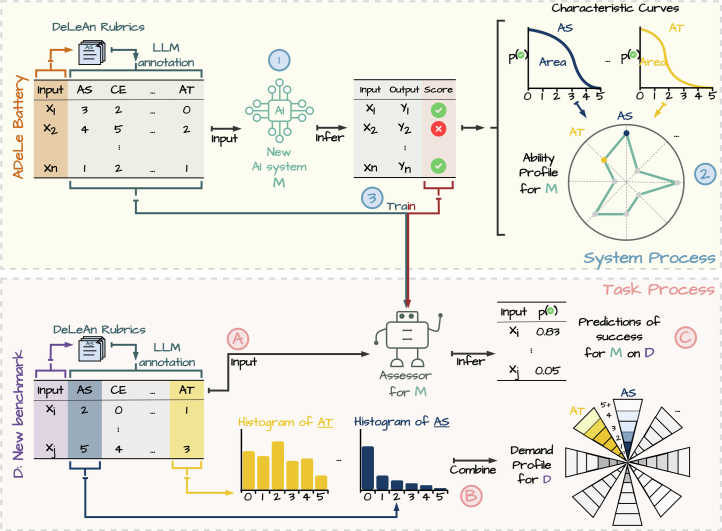
Pipelines to explain and predict performance for new systems and benchmarks. Top, ‘System Process’ – steps for each new AI system: (1) run the new system on the ADeLe battery; (2) plot characteristic curves for all dimensions and extract the ability profile for the system; and, optionally, (3) train a simple assessor using the annotated levels as inputs and the score as output. Bottom, ‘Task Process’ – steps for each new task or benchmark: (A) apply the DeLeAn rubrics to the new tasks using a standard LLM; (B) get demand histograms and demand profiles that explain what demands the tasks require; and, optionally, (C) predict performance for the new tasks for any system that has built an assessor after the ‘System Process’. Assessors based on the demand profile have especially higher predictive power in out-of-distribution settings than other baseline assessors, anticipating validity in new situations.

Extended Data Fig. 5 illustrates a pipeline for our methodology, with two processes that can be followed independently. The ‘System Process’ (top) can be applied to any new AI system we want to explain or predict about and consists of running the model on the ADeLe battery, plotting characteristic curves (see, for example, Fig. 3 and Extended Data Fig. 4) and summarizing the profile of abilities with a radial plot, as in Fig. 4. The ‘Task Process’ (bottom) ensures that the methodology can be extended and kept up-to-date by using the DeLeAn rubrics to automatically obtain a demand profile for new task instances or benchmarks. This is especially useful to mitigate challenges such as data contamination and benchmark saturation while still keeping everything in the same measurement space. This can be compared with the system capability profile for any AI system that has previously gone through the ‘System Process’ to identify specific areas of strength and weakness relative to the task demands and intuitively predict performance. Moreover, we can also train powerful assessors that automatically decide whether it is sensible to use the AI system in a given situation.

In Table 1, we enumerated a series of applications. Here we extend how they are implemented using the pipelines (system or task process) in Extended Data Fig. 5.

- Resolution of apparently inconsistent results (system and task processes): dual profiling of tasks and systems enables us to reconcile seemingly contradictory evaluation outcomes^94,95^. If two benchmarks in the same domain produce different rankings or success rates for a model, the discrepancy can be explained by differences in their demand profiles. For example, several tasks labelled ‘mathematical reasoning’ can require disparate levels of reasoning versus knowledge demands, resulting in inconsistent outcomes that our method can explain. We illustrate this in Supplementary Information Section 1.12.
- Better benchmarks with construct validity by design (task process): designing benchmarks using demand-level rubrics ensures that each task covers the intended range of abilities without extraneous factors, thereby improving construct validity^10^. By selecting instances that span all relevant demand levels, new benchmarks can be aligned with their target constructs by design. In practice, this means that a benchmark will be sensitive (including items of all difficulty levels relevant to the intended skills) and specific (excluding demands relating to unintended skills), resulting in more meaningful evaluation results.
- Benchmarks interoperability and instance reuse into new batteries (task process): items from different benchmarks can be easily integrated into new evaluation batteries (as equating procedures in psychometrics^96^) by placing tasks on the same general demand scales. This interoperability allows us to reuse instances across benchmarks, covering each other’s blind spots and ensuring broader coverage of the capability space. In other words, complementary benchmarks can be merged or linked through their demand annotations to create composite tests that fill gaps (for example, by adding missing high-level reasoning items from one source to another).
- Meaningful scaling laws (system process only, if reusing ADeLe): using the absolute demand/ability scales provides a clearer picture of how model performance scales with size or training. Traditional scaling analyses based on aggregate accuracy often saturate or yield ambiguous trends and indeed there is evidence that naive ‘scaling laws’ can be misleading, break down under certain conditions or do not scale universally^97^. By contrast, evaluating models on our general scales reveals genuine diminishing returns and emergent phenomena, owing to some specific capabilities.
- Measurements robust to changing populations (system process only, if reusing ADeLe: usually, benchmarks are replaced whenever a relevant part of the population of AI systems to which they are applicable achieves accuracy equally close to the maximum (termed ‘saturation’^98^). At the same time, populational methods that infer difficulty levels^18,98,99^ or (the number of) latent factors describing capabilities^16,17^ depend on the considered population of models; thus, the extracted factors or difficulties may lose relevance when the population evolves. With our measurement scales, we can define indicators of progress spanning years or even decades.
- Capability catalogue accommodating AI progress (system and task process): upcoming AI systems may be better described by further dimensions that are not included in the present set, such as having access to affordances that unlock dimensions in the visual domain. The evolution of the catalogue can be used as a mirror of the trends of the discipline and a way of making use of standardized rubrics for new capabilities that appear as AI advances, and can be used for regulation purposes^100^.
- Capability profiles bringing explanatory power (system process): summarizing the performance of an AI system as an ability profile (a vector of scores for each demand dimension) provides valuable insights that go beyond a single aggregate score and offer actionable insights for selection and deployment. These profiles highlight the specific strengths and weaknesses of a model, thus adding an interpretable layer to performance evaluation (for example, they can reveal whether a model’s strong knowledge base comes at the expense of its logical reasoning ability or how training strategies such as chain-of-thought prompting can boost certain capabilities more than others). Recent work evaluating LLMs shows the value of such multidimensional evaluation and analysis^101,102^.
- Model diagnosis and task counterfactuals (system and task process): the demand-ability framework enables fine-grained diagnosis of model failures and ‘what-if’ analyses. When a model fails a particular challenge, we can identify the demand dimension that was high for that item, thus pinpointing the capability shortfall. Recent work decomposes counterfactual reasoning into sub-skills and demonstrates that present LLMs struggle with such tasks^103^, for which prompting is typically used for diagnostic capabilities of LLMs^104^. With profiles, we can adjust task demands or system abilities in a controlled manner, conducting counterfactual experiments to explain how LLMs would behave under modified conditions, lower or higher demands and abilities.
- Routing instance to best system (task process, for LLMs already profiled): a new task instance can be annotated ‘on the fly’ and its demands compared with the capability profiles of AI systems to ‘route’ the task instance to the most appropriate LLM^38,105^. Routers can make use of existing system-specific assessors^21,22,39^. Moreover, routers can also combine performance with considerations such as cost, speed or uptime, whose importance depends on the considered application^38,105^. Given the high out-of-distribution performance of the assessors trained on the ADeLe annotations (see the ‘Predictive power analysis: anticipating performance with assessors’ section), it is conceivable that routers using these annotations will perform similarly well in such scenarios.
- Monitoring LLMs and rejecting queries (task process if LLM already profiled): demand profiles allow for proactive safety monitoring and query rejection^106^ when appropriate. If an incoming query is estimated to require capabilities beyond the reliable scope of a given model, the system can either refrain from answering or delegate to a human operator. Previous studies have shown that a smaller assessor model can be trained to predict the performance of a larger model on individual instances, enabling a ‘reject before you run’ mechanism^39^. This type of anticipatory rejection or deferral contributes to reliability by avoiding situations in which the model is pushed beyond its capabilities^40^.
- Guiding red teaming (task process, annotation only, if LLM already profiled and assessor already built): red-teaming efforts^107^ can be informed by highlighting where an AI system is most vulnerable^108^. For example, if the profile of a model indicates lower ability in metacognition or abstract reasoning, the red team can create prompts that heavily tax these abilities. Also, by inverting inputs and outputs in the assessor, we can test on areas in which the model is weak, ensuring that potential failure modes are covered more thoroughly. This uncovers critical vulnerabilities before malicious actors do^109^ and provides concrete feedback for model improvement, as any weakness discovered is immediately contextualized by the demand that elicits it.

Other applications related to policy, such as safety auditing or regulatory review, require a comparison of LLM and task profiles, with the two processes involved.

All of these applications can use and extend the collaborative platform at https://kinds-of-intelligence-cfi.github.io/ADELE. Predicted extensions will mostly be led by future applications and the evolution of AI. Clearly, more capabilities will be added to the catalogue (for specific domains or to cover multimodal or agentic systems), more levels for some of the capabilities may be needed as AI become more powerful and more annotations of benchmarks, extending or complementing ADeLe for different purposes. This will lead to an evolution of the catalogue and, if necessary, revision of rubrics and their taxonomic relations, provided there is transparency about backward compatibility. This should be the seed of a collective consensus and standardization effort of measurement scales for AI, as has happened in other scientific disciplines.

### Inclusion and ethics

We used LLMs that are trained on very different sources of data and may have important ethical consequences, such as failing in ways users cannot understand or anticipate. This has been the main motivation for this research. The domains we use in our experiments and the examples included in the manuscript do not generate any specific ethical issue. We only use examples and prompts in the English language. The rubric is also only in English but could be adapted to other languages. We did not conduct any human study directly other than a subset of the authors applying the rubrics. More details about the costs of this research (compute), safety implications and other ethical issues can be found in Supplementary Information Section 1.15.

### Reporting summary

Further information on research design is available in the Nature Portfolio Reporting Summary linked to this article.

## Online content

Any methods, additional references, Nature Portfolio reporting summaries, source data, extended data, supplementary information, acknowledgements, peer review information; details of author contributions and competing interests; and statements of data and code availability are available at 10.1038/s41586-026-10303-2.

## Supplementary information

Supplementary Information Supplementary Methods, Supplementary Figures and Supplementary Tables

Reporting Summary

## Source data

Source Data Fig. 1

Source Data Fig. 2

Source Data Fig. 3

Source Data Fig. 4

Source Data Extended Data Fig. 1

Source Data Extended Data Fig. 2

Source Data Extended Data Fig. 4

## Figures and Tables

**Extended Data Table 1:**
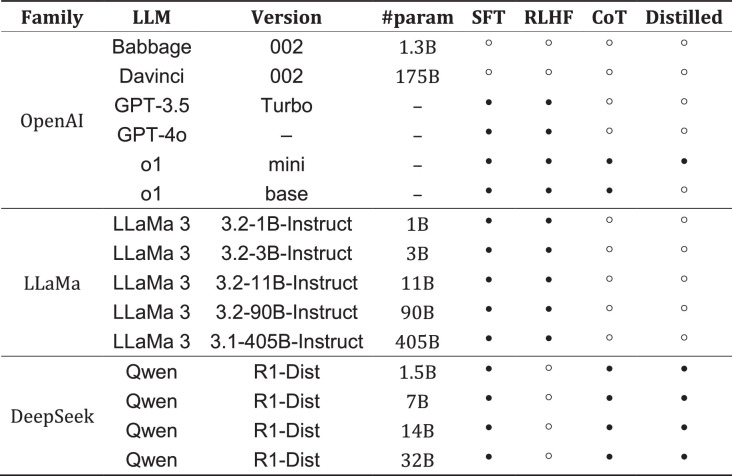
Characteristics of the 15 language models evaluated in this paper

SFT, supervised fine-tuning; RLHF, reinforcement learning from human feedback; CoT, chain-of-thought.

**Extended Data Table 2:**
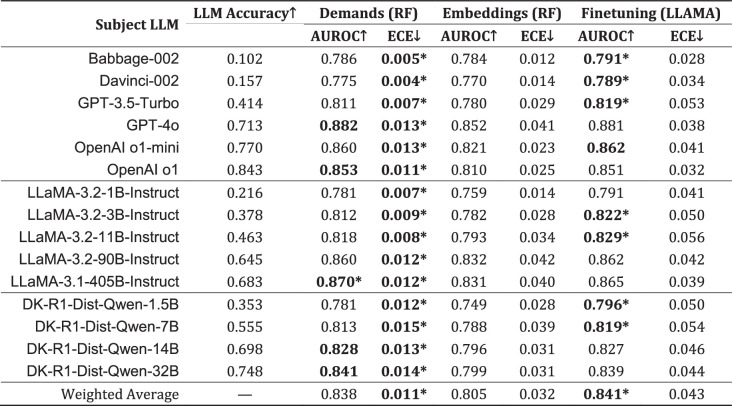
In-distribution predictability results of 15 LLMs for the ADeLe battery using tenfold cross-validation, averaged across ten seeds

The first two columns show names of subject LLMs and the overall accuracy of subject LLMs on the ADeLe battery. The remaining three pairs of columns show the AUROC and ECE of three different assessors (RF using demands, RF using average GloVe embeddings and fine-tuning LLaMA-3.1-8B). For a single LLM subject, the training time is 4 s and 160 s for the demand-based and embeddings-based assessors, respectively, on a M3 Pro CPU, whereas the fine-tuned LLAMA assessor costs 300 h on a single V100 GPU. The weighted average is only indicative for easy comparison, and uses the normalized LLM accuracy as a weight in the mean, giving more relevance to more powerful models, which are more representative now and in the near future. The asterisks indicate statistical difference (*α* = 0.05) between the demand-based assessor and the strongest baseline (fine-tuned LLAMA), using the Wilcoxon signed-rank. The RF assessor’s s.d. across the ten seeds range between 0.0004 and 0.001 for AUROC and between 0.0006 and 0.002 for ECE among subject LLMs. Given these low s.d. scores, we do not show confidence intervals (which are very narrow) for the sake of clarity.

**Extended Data Table 3:**
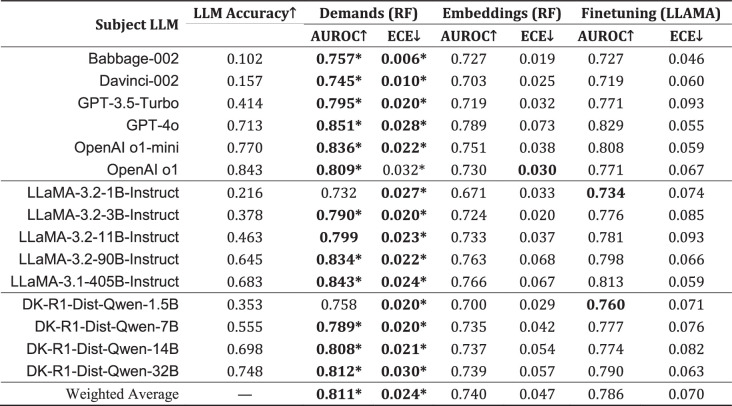
Task out-of-distribution predictability results

15 LLMs for the ADeLe battery using tenfold cross-validation, averaged across ten seeds (all other things equal to Extended Data Table 2 except for the RF assessor’s s.d. across the ten seeds ranging between 0.001 and 0.007 for AUROC and between 0.001 and 0.005 for ECE among subject LLMs).

**Extended Data Table 4:**
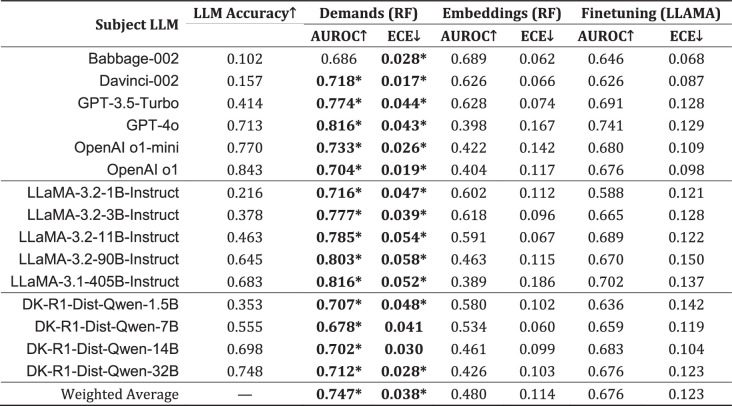
Benchmark out-of-distribution predictability results

15 LLMs for the ADeLe battery using tenfold cross-validation, averaged across ten seeds (all other things equal to Extended Data Table 2 except for the RF assessor’s s.d. across the ten seeds are now ranging between 0.004 and 0.02 for AUROC and between 0.003 and 0.03 for ECE among subject LLMs).

**Extended Data Table 5:**
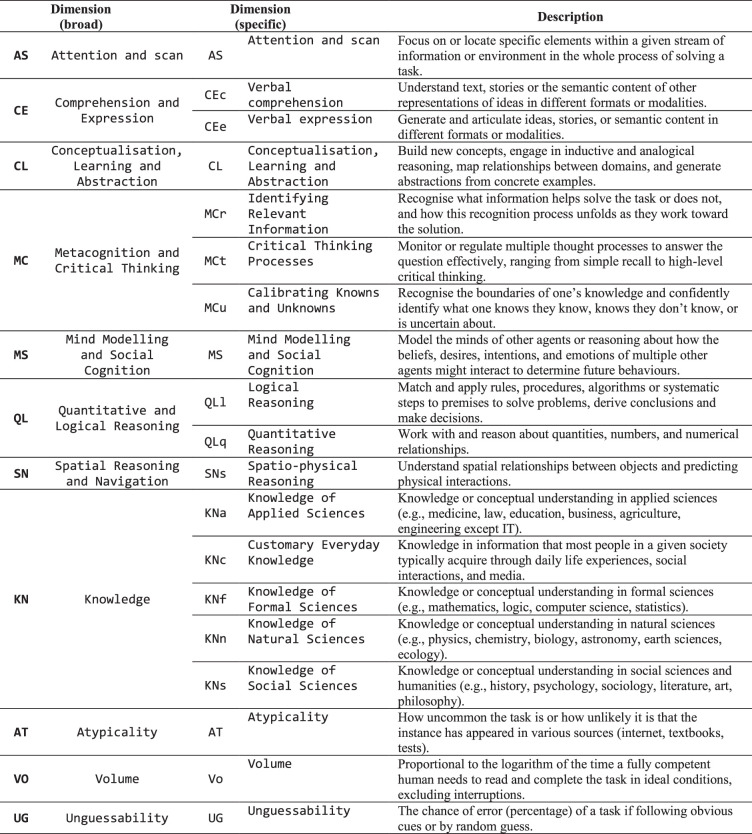
Dimensions and subdimensions in the DeLeAn rubric set v1.0

The first 18 (grouped into 11 ‘elemental’, five ‘knowledge’ and two ‘extraneous’ in a sequential order) are demand scales in the range (0, 5), whereas UG (Unguessability) is not a demand: it is another extraneous dimension representing the (1 minus the) probability of success by random guess or a naive method. For instance, a multiple-choice question with four options would have value 75%. Full rubrics are in Supplementary Information Section 2.

## Data Availability

The associated data, code and instance-level results are available in an independent public platform: https://kinds-of-intelligence-cfi.github.io/ADELE. In compliance with the recommendations of ref. ^9^ about the reporting of evaluation results in AI, we include the results at the instance level. Source data are provided with this paper.

## Code availability

The associated data, code and instance-level results are available in an independent public platform: https://kinds-of-intelligence-cfi.github.io/ADELE. In compliance with the recommendations of ref. ^9^ about reporting of evaluation results in AI, we include the results at the instance level.
