# Supplementary material for: General scales unlock AI evaluation with explanatory and predictive power
Source: Nature. 2026 Apr 1;652(8108):58–67. doi: 10.1038/s41586-026-10303-2 (PMC13043289; doi:10.1038/s41586-026-10303-2)
Supplement: Supplementary file 1 — Supplementary Methods, Supplementary Figures and Supplementary Tables [file 41586_2026_10303_MOESM1_ESM.pdf]

nature portfolio

https://doi.org/10.1038/s41586-026-10303-2

Supplementary information

General scales unlock AI evaluation with explanatory and predictive power

In the format provided by the authors and unedited

General Scales Unlock AI Evaluation with Explanatory and Predictive Power

Lexin Zhou[1,2,3,4,†] **Lorenzo Pacchiardi**[2] **Fernando Martínez-Plumed**[4] **Katherine M. Collins**[5]
Yael Moros-Daval[4] **Seraphina Zhang**[2,6] **Qinlin Zhao**[3] **Yitian Huang**[3] **Luning Sun**[7]
Jonathan E. Prunty[2] **Zongqian Li**[8] **Pablo Sánchez-García**[9] **Kexin Jiang-Chen**[4]
Pablo A. M. Casares[4] **Jiyun Zu**[10] **John Burden**[2] **Behzad Mehrbakhsh**[4] **David Stillwell**[7]
Manuel Cebrian[11] **Jindong Wang**[12] **Peter Henderson**[1] **Sherry Tongshuang Wu**[13]
Patrick C. Kyllonen[10] **Lucy Cheke**[2,6] **Xing Xie**[3,†] **José Hernández-Orallo**[2,4,†]

Abstract

Supplementary Information for the paper 'General Scales Unlock AI Evaluation with Explanatory and Predictive Power'.

Collaborative platform: https://kinds-of-intelligence-cfi.github.io/ADELE.

[†]Correspondence: lz5066@princeton.edu; josephorallo@gmail.com; xing.xie@microsoft.com
[1]Princeton University, US
[2]Leverhulme Centre for the Future of Intelligence, University of Cambridge, UK
[3]Microsoft Research Asia
[4]Valencian Research Institute for Artificial Intelligence (VRAIN), Universitat Politècnica de València, Spain
[5]Department of Engineering, University of Cambridge, UK
[6]Department of Psychology, University of Cambridge, UK
[7]The Psychometrics Centre, University of Cambridge, UK
[8]Department of Theoretical and Applied Linguistics, University of Cambridge, UK
[9]KU Leuven, Belgium
[10]Educational Testing Service, US
[11]Center for Automation and Robotics (CAR), Spanish National Research Council (CSIC-UPM), Madrid, Spain
[12]William & Mary, US
[13]Carnegie Mellon University, US

Contents

1 Main Supplementary Information **3**
1.1 Related Work . . . 3
1.2 Details about the Slicing Method for Characteristic Curves . 10
1.3 ADeLe-Light . . . 11
1.4 Scaling Curves of Model Abilities . . . 12
1.5 Calibration of Assessors . . . 13
1.6 Sources of Unpredictability . . . 14
1.7 Ablations and Other Predictive Models . . . 17
1.7.1 Feature Importance . . . 17
1.7.2 Assessor Only with AT, UG, and VO . . . 17
1.7.3 Assessor without AT, UG, and VO . . . 18
1.7.4 Feature Grouping: Assessor with 11 Broad Dimensions . . . 18
1.7.5 Demand-based Assessor with Logistic Regression 19
1.7.6 A Universal Assessor . . . 19
1.7.7 Algebraic Assessor . . . 20
1.7.8 Further Ablations for Univariate Predictive Power 21
1.8 DeepSeek-V3 and LLaMA-3.1-8b-instruct as Annotators . . 25
1.9 Predictive Power of Alternative Taxonomies . . . 26
1.10 Qualitative Analysis of Demand Annotations . . . 28
1.11 Annotation Perturbation Analysis . . . 32
1.12 From 'can LLMs reason?' to 'how much do LLMs reason?' 33
1.12.1 Toward Reconciling Common Myths on LLM "Reasoning" . . . 33
1.12.2 Negative Claims . . . 33
1.12.3 Positive Claims . . . 35
1.12.4 Two Extra "LLM Reasoning" Papers Confounded by Volume and Chain-of-Thought Effects 37
1.12.5 Summarising the Evidence and Analyses of LLM "Reasoning" . . . 39
1.13 Confidence-interval Width of SCCs by Dimension and Level 41
1.14 SCCs for all models . . . 41
1.15 Inter-rater Agreement for Humans and GPT-4O . . . 57
1.16 Prompt templates . . . 57
1.17 Benchmarks in ADeLe v.1.0 . . . 59
1.18 Cost, Ethical and Safety Implications . . . 60
1.19 Glossary . . . 61
1.20 References in Supplementary Information . . . 64

2 DeLeAn Rubric Set v.1.0 **76**
2.1 Elemental . . . 76
2.2 Knowledge . . . 88
2.3 Extraneous . . . 94

3 Rubrics for Baseline Taxonomies **98**
3.1 Miller & Tang . . . 98
3.2 BLOOM . . . 100
3.3 Generic Difficulty . . . 101

1. Main Supplementary Information

This section includes a full coverage of the related work, more details on the slicing method for building the characteristic curves, a data efficient version of ADeLe known as ADeLe-Light, an analysis of scaling curves using abilities instead of performance, the full calibration plots for the assessors used to predict performance, a study of the sources of unpredictability in the data (aleatoric uncertainty), the results for other predictive models (feature importance, ablations, etc.), the use of other LLM annotators beyond GPT-4o, the predictive power of alternative taxonomies as baselines, qualitative analysis of demand annotations between humans and the LLM annotator, a detailed analysis reconciling common myths on LLM "reasoning" through 20 extra so-called "reasoning" benchmarks, quantification of confidence interval widths of subject characteristic curves by dimension and level, the subject characteristic curves for all LLMs, a full account of cost, ethical and safety implications, and a glossary of terms.

1.1. Related Work

This paper builds on many disciplines inside AI, such as natural language processing and machine learning, and outside AI, such as psychometrics, cognitive psychology and measurement theory. We will cover the literature which we deem more directly related to this paper in a non-historical order.

Meta-feature extraction and annotation in linguistics and NLP. A crucial element of this work is the annotation of testing instances, tasks, or problems. Feature extraction is a traditional approach in many areas of machine learning, especially in Natural Language Processing (NLP). However, traditional NLP generally focused on this for the purpose of building a good tabular representation for training the subject models solving the task. Here, we focus on meta-features extracted to understand the type of question or problem and its complexity, rather than as predictor variables for the original problem (e.g., answering a question). (Computational) Linguistics and NLP developed metrics of vocabulary and syntax complexity of sentences or paragraphs based on readability assessments (for humans), from the Flesch Reading Ease Score [1] to modern approaches integrating NLP itself and machine learning for the extraction of text characteristics meta-features, such as Coh-Metrix [2] and others [3]. The use of LLMs as automated annotators is becoming a viable alternative to some of these metrics and is replacing many manually intensive annotation processes [4–7], even if requiring some level of human verification [8].

Taxonomies of capabilities and rubrics. Beyond linguistics, and when dealing with cognition more generally, a primary question is how to carve the space of capabilities, skills and knowledge. Several theories of cognition and intelligence have been developed, informed by conceptual frameworks, experimental data or a hybrid of both. For instance, the Cattell-Horn-Carroll hierarchical structure of human cognitive abilities [9] is a popular general taxonomy in psychology, but many others exist, both describing cognition at a general level or focusing on specific areas of behaviour [10]. Our elemental dimensions were adapted from [11, 12], that in turn based them on figures 3.1 and 3.2 (human psychometrics, from Thurstone's primary mental abilities [13] and Cattell-Horn-Carroll hierarchical model), table 4.1 (animal cognition research, from Wasserman and Zentall's book [14]) and tables 5.2, 5.3 and figure 5.3 (AI, AGI and benchmarks, from AI Journal and [15]) of [16]. There is a tradition of creating rubrics for the annotation of assessment items [17–19] based on a taxonomy, independently of whether that comes from experimental human data, theories of cognition, or subfields of AI. For example, the CRAS scales of demands [20] feature a five-level scale composed of five meta-level dimensions: complexity, resources, abstractness, task strategy, and response strategy. These dimensions bear a resemblance to aspects of our `Metacognition` and `Volume` dimensions. Moreover, many additional dimensions are employed across various fields in psychology and the behavioural sciences. In the paper, we include Miller & Tand's taxonomy [21], covering six categories related to generative tasks (summarisation, technical assistance, reviewing work, data structuring, generation and information retrieval), and Bloom's taxonomy [22, 23], which rates the instructional goals of a given task item into six levels (knowledge, comprehension, application, analysis, synthesis, and evaluation). Finally, it is important to acknowledge that all taxonomies are culture-laden. This is not only because the concept behind a capability may have (more) validity in some subpopulations or cultures (e.g., 'executive functions', [24]), but also because we want them to be understandable by humans.

Annotating LLM benchmarks for cognitive demands. Since the possibility of using LLMs for annotation is quite recent, there is a disconnect with some of the rubrics used in educational settings and those used to annotate NLP benchmarks, including LLMs themselves. Out of the large amount of LLM benchmarks [25], only a few are designed around specific dimensions and annotated according to numerical demands on those dimensions [26, 27]. Other studies instead build controlled evaluation experiments varying various factors, but these factors are qualitative rather than quantitative [28–31]. A few works explored automatic generation of variations of benchmarks, which could be encoded in annotations. For instance, Wang et al. [32] assessed how performance is impacted by noise and textual perturbations, modifications that are quantifiable but much more specific than the domains we consider. Instead, Cao et al. [33] probed LLMs' performance by extracting "atomic test objectives" from questions and crafting new ones to test each element of Bloom's taxonomy [22, 23] of cognitive levels (Remembering, Understanding, Applying, Analyzing, Evaluating, Creating); these levels are more generic than the domains we consider—moreover, our method obtains multidimensional quantitative annotations for task instances of any type, while their modification only tests a single level at a time and can only be applied to questions with a single atomic test objective. Finally, Zhu and colleagues [34] transformed problems by paraphrasing them or adding extra context or choices; while these transformations are akin to increasing the demands for our `Verbal Comprehension` and `Attention and Scan` dimensions and multiple transformations can be applied at once, those are only 2 out of the 19 dimensions we consider (excluding the extraneous dimensions). Moreover, all of the above works require testing LLMs on a large set of novel prompts to obtain a comprehensive profile, while our approach obtains it from existing evaluation results, after the annotation has been performed once. Moros et al. [35] developed a similar methodology for annotating cognitive demands to what we present here, but only provided examples of the levels rather than detailed rubrics, did not include dimensions about `Knowledge`, and conducted smaller-scale experiments. Relatedly, Zhou et al. [36] showed that automatically annotating linguistic features using an LLM at scale can enable the construction of interpretable classifiers or regressors for dialogue constructiveness assessment and outcompete various strong neural black-box baselines in terms of prediction quality. Our work can be seen as scaling up these approaches.

Generic critiques of the state of AI evaluation. There are issues in AI evaluation that apply to a broad range of AI paradigms [37], such as issues with measurement scales [38], reproducibility [39, 40], statistical rigour [41], representativeness and fairness [42, 43], and other ethical aspects [44, 43]. Some issues that are closely related to some of the problems we address in this paper are the effect of `Volume` (such as, for LLMs, adding tokens to the question definition [45] and the "needle in the haystack" phenomenon [46]) or `Atypicality` (such as dataset contamination or over-optimisation [47–51] or more general "training on the test set" [52]) or `Unguessability` (such as the effect of different multiple-choice format questions [53, 54] for LLMs). The problem of saturation is also recurrent, and has been mentioned multiple times since the era of AI acceleration began and benchmarks were broken more often [55, 56]. Finally, [57] surveyed inadequacies related to validity (discrepancies between what an instrument is intended to measure and what it actually measures), security issues, and the failure to capture cultural diversity, and claimed that ecologically-valid evaluation needs to pay more attention to the real world [58, 59, 39, 60] or to situations where the AI systems do not achieve their solutions independently, but in interactive settings with people [61, 62].

Issues with benchmarks. Numerous studies have examined challenges associated with benchmarking [63, 64], the prevailing paradigm for AI evaluation [37], most often involving aggregate measures of model behaviour (usually performance) on a fixed set of instances related to a task or a few tasks. For example, in the context of traditional supervised Machine Learning systems, Liao et al. [65] provided an overview of issues within the benchmarking paradigm, while Bowman et al. [66] proposed essential criteria that NLP benchmarks should fulfil. Still considering NLP, Subramonian et al. [67] conducted a survey of AI researchers to develop a taxonomy of issues related to the "validity" of benchmark measurements—specifically, discrepancies between what a benchmark is intended to measure and what they claim they measure, usually referred to as test validity [68, 69]. For instance, Siska et al. [70] found the correlation between the performance of multiple LLMs across benchmark test instances to be non-random and that "accounting for [this] can change model rankings on major benchmarks", with common failure points partly explaining the correlation; here, our instance-level annotation would allow to clarify the factors causing these correlations in performance. Moreover, our extracted model capabilities robustly rank models along different dimensions independently of the distribution of instances that composes the benchmark. At a different level, Ren et al. [71] found the performance on different benchmarks to be related, focusing on "safety" and "capability" benchmarks.

While our paper is purely focused on capabilities, the influence on safety is important, and how our approach could be extended to include "safety" demands is a promising direction for future work.

Building better benchmarks. Finally, there have been many suggestions on how to build better benchmarks. For instance, Momennejad et al. [31] provided guidance on building evaluation frameworks to measure whether LLMs possess capabilities that are robust to various perturbations and experimental conditions, thereby improving validity. Relatedly, Liu et al. [72] proposed a protocol for designing benchmarks around capabilities (constructs) and collecting items that best elicit evidence of the targeted capability. They view "benchmarking as the process of gathering capability evidence from objects of evaluation [...] —i.e., evidence about whether or to what degree those objects have some capabilities of interest", aligning with our goal of measuring LLMs' underlying capabilities rather than superficial benchmark performance. Their analysis of existing benchmarks reveals poorly conceptualised constructs and unclear instance-capability relationships, echoing our finding that benchmarks often include unintended demands. At the same time, BetterBench [73] identified 46 best practices for building benchmarks, mostly related to reproducibility, documentation and statistical significance, despite mentioning construct validity as an open problem. These criteria are therefore complementary to the above approaches focusing on validity, and the analysis of construct validity our approach affords can be combined with their criteria.

Ability-oriented AI evaluation and AI predictability. LLMs are an example of General-Purpose AI (GPAI) models: AI models that can be applied to an extremely large variety of tasks[1]. The evaluation approach discussed above, benchmarking, is exported from traditional supervised machine learning practice, where a bespoke ML model was trained for a specific task; there, evaluating a model in terms of its accuracy on a test set was sufficient to gain information on how the model would perform if the real-world data distribution was represented in the test set (which could be subject to minor distribution shift with respect to the training set). However, due to their generality, a fixed benchmark cannot represent the full distribution of applications an LLM is used in. To partially address this problem, an LLM's performance on a collection of benchmarks is usually reported. However, such a *task-oriented* evaluation does not fully characterise a model's behaviour for two reasons: 1) benchmarks represent a specific distribution of instances of a task, but the difficulty of problems of the same domain encountered in the real world may be different [70]; 2) it is impossible to assess the performance on the complete set of tasks to which a GPAI model can be possibly applied.

Therefore, it has been advocated [75, 76] that GPAI evaluation must become *ability-oriented*, namely focusing on inferring a set of abstract latent capabilities (for instance, through the use of construct-oriented evaluation tools [37] that functionally connect observed behaviour to latent variables) that comprehensively characterise a model's behaviour. Starting from the obtained capability profile, the model's performance on specific task instances could be predicted from the "demands" they pose on each capability, even if the model was not previously evaluated on task instances with that specific combination of demands. Therefore, ability-oriented evaluation provides an interpretable description of a model's characteristics and fosters the goal of building predictable AI [77], which is important to anticipate and mitigate model errors and thus contribute to safety.

Our work is an example of ability-oriented evaluation: we extract capabilities of models related to the dimensions we define, which comprehensively characterise a model and apply across various tasks and domains. Further, the inferred capability levels could be combined with the annotated demands of each task instance to predict the model's performance on new individual task instances (for instance, with an approach similar to that in Burden et al. [78]) or the accuracy on a benchmark with a domain profile, rather than building separate assessors as we did here. It is important to note that using radial plots in AI evaluations—even when labelled with the term "capability"—does not inherently imply an ability-oriented assessment. Often, these plots merely represent aggregate performance, broken down by benchmarks or their subdomains [79–82]. They are not in the form of commensurate quantitative scales for the set of capabilities, and then end up being reported as percentages. For a score to genuinely qualify as a capability, the evaluation must incorporate either binary demands or difficulties. Also, only when capabilities are defined as counterparts to demands or difficulties, can we properly talk about notions of generality as opposed to capability [83] or as a derived metric from a range of capabilities [84]. The profile we extract with our approach could be used for this purpose.

[1]For instance, the EU AI Act [74, Article 3(63)] defines a model as GPAI if it "displays significant generality and is capable of competently performing a wide range of distinct tasks [...] and that can be integrated into a variety of downstream systems or applications".

Extracting profiles of latent features explaining the behaviour of LLMs. Through the annotation of instances for the demands they pose on various dimensions and instance-level analysis of results, our approach can explain performance through a "profile" of model capabilities, in the spirit of ability-oriented evaluation (see the above paragraph). A few other works extracted latent dimensions characterising LLM behaviour. For example, both Burnell et al. [85] and Ilic et al. [86] explained the variance of aggregate performance on different benchmarks for a population of models via a set of latent factors inferred through factor analysis. In particular, Burnell et al. [85] found 3 latent factors to explain a large part of the variance and, by manual inspection, realised that these partially align with an *a priori* classification of the cognitive skills needed to solve the benchmarks (then interpreting the factors as *reasoning, comprehension and language modelling*). Ilic et al. [86], on the contrary, identified one general factor only. Similarly, Ruan et al. [87] introduced "observational scaling laws" that connect performance on complex downstream tasks with hypothesised latent capabilities. These latent values are estimated by decomposing the performance of a population of LLMs across different benchmarks into components that follow a log-linear relationship with the compute measures used in LLM training. All of these methods rely on a population of models and explain aggregate performance; in contrast, by using instance-level annotations and performance of a single LLM, the capability profile we obtain is independent of the choice of an LLM population and of their compute footprint. Moreover, while their inferred latent factors have to be identified a posteriori, our capabilities are relative to a pre-defined demand, facilitating interpretation. At the same time, the assessors we train allow us to predict instance-level performance on an instance on which models were not previously tested; in contrast, the observational scaling laws in Ruan et al. [87] can predict aggregate performance on a benchmark which was previously processed in their framework, although they can do that for new models of a family that was used in their analysis, based on compute measures. Instead, Burnell et al. [85] and Ilic et al. [86] offer no predictive power for new models or new tasks beyond a qualitative estimate based on what abilities are presumed to be useful for a task.

Uncertainty estimation and calibration. There are two main categories of approaches for modelling uncertainty in large language models. White-box methods require access to the model's internal parameters or output probabilities. For example, Kuhn et al. [88] and Duan et al. [89] use semantic entropy as a way to quantify uncertainty, while Cohen et al. [90] introduce a special token [IDK] to explicitly model uncertainty during training. The problem with these methods is that, since they require full access to the models, they are not always feasible to apply, and even when they are, many are computationally expensive. Furthermore, when evaluating already existing models, any method that involves this access is excluded. By contrast, when full access to the model is unavailable, black-box methods are employed. Kadavath et al. [91] propose making the language model itself estimate the probability that its answer is correct ($p(True)$). Along these lines, Xiong et al. [92] explore asking the model directly about its confidence in its answer using different types of prompts. Additionally, they also propose querying the model multiple times and inferring its confidence based on the consistency of its responses, which is more properly a calibration method. Furthermore, Shrivastava et al. [93] suggest using an auxiliary model to assist in estimating the uncertainty of the primary model, but not independently from it. Limitations of these methods include the need of several runs of the model or complex prompts that may lead to different answers to those the model would answer in normal circumstances. On top of these limitations, uncertainty quantification does not serve the purpose of evaluation, as it does not give insights of why the LLM is failing, does not characterise tasks and LLMs in terms of capabilities, and as said above, is beaten by external assessors, as we survey next.

Instance-level performance prediction of AI models. Zhou et al. [77] emphasised the importance of instance-level success predictions for AI models, coining the term "predictable AI". They argue that in high-stakes applications, prioritising predictability is a more valuable goal than pursuing unpredictable improvements in average performance. As a precursor of this idea, Hernandez-Orallo et al. [94] introduced the concept of an *assessor*—a model trained to predict an ML system's performance on individual instances based on its evaluation results on test data (i.e., data not used for training the ML system). Several studies have explored variations of this approach. Zhou et al. [95] demonstrated that a smaller LLM could be trained to predict the performance of a larger LLM on individual instances without direct access to them. This assessor successfully rejected nearly half of the failure cases, leading to significant computational savings. Schellaert et al. [96] accurately predicted LLM performance across more than 100 BIG-bench tasks [79], surpassing the confidence of the models themselves while maintaining predictability across different model sizes, suggesting scalability. Drapal et al. [97] extracted explainable meta-rules from trained assessors to identify regions where

performance is predictable. Instead, Pacchiardi et al. [98] trained assessors that leveraged shared information across multiple LLMs, reducing the number of instances each LLM needed to process for assessor training. In general, assessors are based on features that are difficult to interpret, and extrapolate poorly out of distribution [96, 98–101]. In this work, we place ourselves in this strand of literature by training assessors using our annotated demands on the various dimensions, which are interpretable and, due to their generality, extrapolate well out of distribution.

Beyond the assessor framework, alternative techniques from other fields have been adapted for instance-level performance prediction. For example, Drapal et al. [102] combined novelty detection with meta-learning to filter out instances where an ML system is likely to fail (additional similar approaches are discussed in Section 4 of Hendrickx et al. [103]). Another related approach, inspired by intrinsic uncertainty quantification [104], is Kadavath et al. [91], which trained LLMs to estimate their probability of success on a question without reference to a specific answer. This was achieved either through natural language responses or by adding an additional "head" to the model.[2] Additionally, the approach in Burden et al. [78] explicitly modelled the probability of an AI system succeeding on an instance using a set of system's latent capabilities and instance-level demands, and inferred a posterior for the capabilities using Bayesian inference starting from annotated demands. A prediction on an instance with assigned demands can then be obtained in terms of the "posterior predictive" distribution. Finally, Item Response Theory (IRT) [105] can be used to predict performance of AI models, but only on previously processed instances; this was done in previous work [106], despite not being the main focus. We discuss connections of our work with IRT more extensively in the subsequent paragraphs. Finally, Pacchiardi et al. [107] introduced *Predictaboard*, a standardised benchmarking framework that jointly evaluates an LLM's performance and the ability of a performance prediction method, facilitating comparisons across assessors (including possibly our own) and other approaches, such as the ones discussed above.

How human users predict and understand LLM performance. In parallel with developing a score predictor model, an important question arises: How effectively can humans predict where AI might fail? Several works address this question. For instance, Carlini [108] found that human predictions are only marginally better than chance at predicting GPT-4's performance. Relatedly, Vafa et al. [109] demonstrated that humans tend to overestimate the future performance of LLMs based on prior interactions, particularly with larger models in high-stakes contexts. They argue that "the best LLM is the one that allows humans to make the most reliable inferences about where it will succeed". Zhou et al. [110] showed that as AI systems become more capable, human predictions of their performance become increasingly unreliable. Furthermore, Steyvers et al. [111] found that LLM-generated explanations supporting a statement do not enable humans to reliably determine whether that statement is correct, even when the LLM's token-level probabilities are well-calibrated. Additionally, longer explanations were observed to increase user confidence, regardless of their accuracy. Our approach provides an interpretable profile of the capabilities of LLMs, which may help human users to more reliably predict where LLMs will perform successfully.

Item Response Theory. Item Response Theory (IRT) [105, 112] originally developed as an alternative approach to classical test theory in psychometrics where the notion of difficulty was chosen to play a central stage [10]. One of the key observations in the evaluation of cognition and intelligence is that correlation between tests appears when the range of difficulties of the items in a test generates enough variance in the population. Not controlling for the difficulty of the items may lead to inefficient testing and the wrong information about whether several latent factors correlate, or simply missing an important construct because the range of difficulties was insufficient to generate enough variance in the population. As a reaction, IRT contrasts difficulty and ability from the start, and sets a common scale for them. Given a matrix of results of subjects and items, IRT can estimate the ability of the subjects and the difficulty of items at the same time. Many traditional IRT models represent the probability of success as a logistic function of the difference between ability and difficulty. The simplest of these models, the 1-PL or Rasch model, only estimates ability and difficulty, assuming the logistic curve has slope 1. The 2-PL model extends this by allowing a second parameter, the slope of the logistic curve (termed *discrimination*) to be inferred from the data. The 3-PL model includes a *guess* parameter, which is the minimum expected value of the response, which is usually appropriate for multiple-choice questions where distractors are obvious and the baseline goes above the statistical chance. In general,

[2] Many other studies apply intrinsic uncertainty quantification to assess whether a model perceives a given statement as correct or incorrect after generating an answer. However, this differs from predicting performance *before* an answer is produced. See Shorinwa et al. [104] for a broader discussion.

the guess parameter not being 0 is useful when there is a chance of success even without ability. This has inspired the `Unguessability` dimension in this paper. The logistic curves are called item characteristic curves, with ability on the x-axis and response on the y-axis for a population of subjects. The dual curve is called the person characteristic curve [113, 114], indicated in this paper as subject characteristic curve, which maps response on the y-axis for a population of items as a function of the ability of one subject on the x-axis. An important thing to clarify is that IRT cannot predict performance for items that have not been characterised during the estimation phase, and the parameters depend on the population of subjects. If the subjects or the items are changed (very different groups of humans), all the parameters may change. IRT has been adapted to machine learning and NLP [115–117, 106, 118, 119]. Of the many works adapting IRT to AI evaluation, a few applied it specifically to analyse LLM performance. For instance, Fang et al. [120] started from a test which has been validated on humans and converted it into prompts that are suitable for LLMs. It used human performance to obtain item difficulty and then used them to analyse LLM performance and get capability scores, using IRT-like models. Very similarly, Zhuang et al. [121] extracted difficulty of different instances in a benchmark and models' abilities from a population of models. Instead, Tang et al. [122] introduced a dataset annotated using 5 levels of difficulty obtained from human performance scores, while Lei et al. [123] considered a dataset annotated with difficulty scores by human experts and employed a simple psychometric model to finding differences in how success for humans and LLMs correlates with the annotated difficulty. Finally, Federiakin et al. [124] applied IRT to improve rankings on the HuggingFace leaderboard. Ding et al. [125] apply IRT and Glicko-2 models (another approach to derive latent difficulty, especially in situations zero-sum two-player games, as the data is obtained from performance data from humans, LLMs but also preference leaderboards). Other works instead used IRT to improve the efficiency of benchmarking: Polo et al. [106] and Kipnis et al. [117] used IRT to select informative subsets of a benchmark and estimates the performance of a new LLM on the whole benchmark by evaluating it only on those instances.

Multidimensional Item Response Theory. On many occasions, one single ability-difficulty pair is not sufficient to account for the variability of responses; rather, more than one dimension is needed. Multidimensional IRT [126, 127] replaces the notion of item characteristic curve with an item characteristic surface, where two or more dimensions of ability-difficulty are represented. Estimating this surface will depend on the assumption of independence of the dimensions. Two abilities are considered compensatory if the lack of one can be compensated by the other. For instance, for recognising a person, we can use face recognition ability and voice recognition ability. In a situation when one ability is affected or the demand is too high (e.g., noise in the image or the audio), then the other can compensate. Conversely, two abilities are considered non-compensatory when the lack of one cannot be compensated by the other. One important question from multidimensional IRT is how to extract individual dimensions from the multidimensional space that are calibrated [128]. This depends on a series of assumptions, such as the level of compensatoriness. Some multidimensional IRT models have been applied to machine learning as well: Liu et al. [129] considered an extension of multidimensional IRT using a set of subjects, their instance-level performance on a dataset and a binary annotation matrix encoding the "skills" required by each instance to infer levels of skill-specific ability for the different subjects and obtain an overarching difficulty (and discrimination) factor for each instance. These inferred quantities depend however on the considered population of learners, in contrast to our approach, which is non-populational. Further, our approach uses numerical levels of demands over different dimensions, while they only considered binary indicators for whether a specific skill is needed in an instance, without quantifying skill-specific demand.

Other extensions of IRT. Linear logistic test models (LLTM) [130] consider a vector of binary demands for each item, expressing if a given demand is needed or not for a particular item (they can also be greater than one, in an ordinal or quantitative scale). The matrix of all items and demands is called the Q-matrix. LLTM considers one single difficulty as a linear function of the demands, with the parameters of that function being estimated from data, and being the same for all subjects and items. Similarly to traditional IRT, the item difficulty is opposed to the ability. Despite having several demands, the model assumes one single ability per subject, and it cannot generate ability profiles. Also, the parameters are estimated from the whole population of items and subjects, and these parameters can change if the subject population changes. However, one great advantage of LLTMs is the much smaller number of parameters to estimate, as difficulty is derived a function of the demands. Because of this, it can be applied to new instances. LLTM has been generalised by the area known as 'explanatory item response models' [131] where the approach LLTM applies to item parameters is extended to person parameters or both person and item parameters (known as

doubly explanatory models), or in the multidimensional case [132]. Cognitive diagnostic models [133–135] are a related approach and also use a Q-matrix, but can generate 'multidimensional skill profiles'. Lately, machine learning methods are being used to estimate these models. Wang et al. [136] provide a history of the entire field, encompassing both the traditional approaches and the new ones based on machine learning. Finally, the bifactor scoring model [137], separates a general factor, accounting for communality among all items (or items from domains that are highly correlated), and group factors (the capability dimensions), accounting for domain-wise variances. Unfortunately, most of these advanced models have not been applied in AI, despite the recent calls to do so [138].

Situation of this paper in the space of psychometric methods. Our work adapts and integrates many ideas from psychometrics, in particular from IRT. Our approach is similar to LLTM in the use of demands (the ADeLe battery can be seen as a Q-matrix) and especially to explanatory item response models and cognitive diagnostic models. However, we do *not* consider demands as being determined by the difficulty for a fixed population. The analogy with the bifactor scoring models can shed light on our observations of our subjects not having probability of success 1 at demand level 0 (as can be seen in the subject characteristic curves): that is because the subject needs to understand the question and know that they need to give an answer. To account for this, some IRT approaches introduce a parameter called *inattention*. Our approach can be seen as starting with a multidimensional model, assuming independence, and then slicing it using the 'dominant' approach. In general, slicing multidimensional spaces into unidimensional spaces implies important assumptions and depends on the data [139]. Our use of the 'dominant' slicing approach induces a dependence on the correlations between dimensions in the battery we consider, which may lead to poor calibration the less pure the items are. The major difference between all of the above psychometric models and our methodology originates from the nature of the subject: humans for psychometrics and machines for AI. For adult humans, the notion of population is meaningful and is generally stable. However, the capabilities of AI models and their similarity are changing quickly, so any result that depends on the variance of the population of benchmarks would need to be reconsidered every year when a new generation of models appear. For instance, the factor analysis studies changed conclusions between [85] and [86], most likely because of a different sample of models, despite the studies being conducted just a few month apart. In principle, we can produce a score for single individuals by looking at the individual's responses to items and generating a score from the model parameters [140]. However, psychometrics rarely builds a model from a single human with a "cold start" (i.e., not adapting models informed by a population), because it makes sense to use the information of many other humans, and collecting a sufficient amount of data from a single individual is costly. In the case of our battery ADeLe, we have more than 16,000 items per LLM, from which we can make strong inferences (including high predictability). The other difference is that very few dimensions are usually enough for humans, because most capabilities and traits show high correlations. Moreover, the smaller number of items in human studies with respect to what is possible with AI systems lead to stronger effect of noise, which reduces the number of parameters that can be accurately estimated: with 50 items, a single odd item may produce noise and estimate error, but a high number of dimensions is possible with 16,000 items. As such, with more data, there is greater potential to uncover regularities beyond a limited set of dimensions, thereby revealing processes that might otherwise remain undetected.

Item design, procedural generation and adaptive testing. In our paper, we have not explored exploiting the rubrics and annotations for better item design, procedural generation [50] and adaptive testing. These approaches are common in assessment, especially with the use of difficulty levels from IRT, but have also been adopted to AI. For instance, Zhuang et al. [121] used IRT to extract difficulty of different instances in a benchmark and relied on these to perform adaptive testing by selecting items whose difficulty is more informative of a model's ability level. A similar approach could be taken with our annotated demands, although their multidimensional nature should be taken into account. Instead,Guinet et al. [141] used IRT to improve the quality of a benchmark for Retrieval Augmented Generation (RAG) LLMs by eliminating the questions that are not sufficiently informative about a model's ability. Again, we could analogously rely on our extracted demand levels to ensure that a benchmark comprehensively tests LLMs in a range of situations. Finally, Zhang et al. [142] performs adaptive labelling and procedural generation simultaneously. Interestingly, their categories are organised in a taxonomy of binary dimensions.

Measurement theory and AI scales. Two important concepts in measurement theory [143] are the type of scale and the measurement units. Steven's typology of measurement [144] includes 'nominal', 'ordinal', 'interval' and 'ratio'

scales, and many other topologies have appeared since then. For quantitative measurement, interval scales make the comparisons of differences meaningful, and calculating a mean is well justified. Ratio scales set an absolute value, and hence ratios become meaningful. For a latent factor, the choice of a scale is arbitrary. In IRT, it is somewhat controversial (e.g., [145, 146]). Although some researchers advocate for a ratio or interval-scale basis for test scores (e.g., 'the odds (or log odds) of student 1 answering a class of items correctly are twice the odds (or log odds) of student 2 doing so' is a meaningful assertion [147]), others point out that a latent trait scale of a fitted IRT model is convenient but not inherently meaningful because any monotonic transformation of the estimated latent trait scores produces an equally valid model [146, 148]. In contrast to this, some magnitudes are associated with cognition have a clear scale, such as size or time. For instance, for the dimension `Attention and Scan`, the length (e.g., in words) could be relevant, and for the `Volume` dimension we have used time (and we saw correlation between the size of a question and the time to solve it, [149]). Time is isolated in the person-month metric, used everywhere in human resources, project management, or software engineering, which can be mapped to educational levels, taking into account the effort in days that several levels require (e.g., OECD data [150]). Morris et al. [151] uses percentiles of the human population for their Emerging, Competent, Expert, Virtuoso and Superhuman levels of AGI. Here, we do not base the levels on outperforming percentiles, but on the probability of finding a sample of 10^l humans with more than 95% chance of at least one being correct.

1.2. Details about the Slicing Method for Characteristic Curves

The 19 dimensions, with values between 0 and 5 each (except `LG`), constitute a multidimensional space. For a particular subject (AI system) and each instance $\mathbf{x}$ in the `ADeLe` battery having the same values for the vector, we can calculate the percentage of correct responses, to get an extra 20th dimension for performance. This will create a surface in this 20-dimensional space representing the capability footprint of an AI system, in the same way multidimensional IRT does [126, 127]. However, having at least one instance for each combination of levels and dimensions would require 6^{19} instances, which is not only a big number, but also hard to achieve, as finding items for all combinations can be very challenging. Actually, the correlation matrix between dimensions (Extended Data Figure 1) suggests that it is rare to find some particular combinations of levels, so densely populating this space would be a big challenge.

Performing a dimensionality reduction can alleviate this problem but it will reappear as more relevant demands are added to the rubrics in the future (multimodal AI, robotics, etc.) and the scales are extended beyond level 5 (more powerful AI), as we set in the design criteria for this methodology. Instead, we propose to explore unidimensional distillations of that original space, having a characteristic curve for each dimension, rather than a surface for all of them. These curves are called person characteristic curves [113, 114], whose name we generalise here to subject characteristic curves.

One way of doing this would be to find task instances that are pure, i.e., having a level above 1 for one dimension and 0 for all the others. This would be the slice of the space for that 0-hyperplane for all other dimensions. However, again, it is difficult to find 'pure' instances, only having a non-zero level for one dimension. Even if this were feasible, most of the items in the battery would be discarded in the process, unless we impose draconian requirements on item design. Therefore, when trying to represent how a subject performs for one single dimension i, it is unreasonable to select an instance only if its demand vector $\mathbf{x}$ has a profile like $(0, ..., 0, x_i, 0, ..., 0)$, with $x_i \in [0, 5]$. Instead, what we can do is to derive the characteristic curve for dimension i such that for level l we only consider the instances for which $x_i = l$ and all other $x_{j\neq i} \leq l$ (or similarly, $\max_j x_j = l$). We call this approach *'dominant' slices*. For instance, for dimension 5 (`MCr`) and level 2, we have three items in Figure 5 with `MCr`=2: the vector corresponding to the population question $(0, 1, 1, 0, 2, 1, 1, 0, 1, 1, 0, 0, 1, 1, 0, 0, 0, 1)$ would be kept since $\max_j x_j$ equals 2 but the vectors corresponding to the Baron Todd question and the 58-year-old male question $(3, 2, 1, 1, 2, 1, 2, 0, 2, 0, 0, 0, 3, 0, 0, 0, 3, 2)$ and $(2, 3, 1, 0, 2, 2, 1, 0, 3, 2, 0, 5, 0, 2, 4, 0, 3, 2)$ would not be used for this dimension, since $\max_j x_j$ equals 3 and 5 respectively, both greater than 2. This has the advantage of finding instances meeting the condition for all levels, while rejecting those for which another demand is playing a more significant role in explaining the response. In other words, we keep instances for dimension d at level l if no other dimension dominates. The only level for which we will not get instances is level 0, since there are no 'control' instances with all dimensions set to 0. This way of 'slicing' is both pragmatic and conceptually aligned with established psychometric practices for isolating dimension-specific response functions [152–154], also allowing use to use most of the instances in the battery across dimensions. It does not consider the correlations (which will vary if we change the composition of the battery) and does not assume any level of compensatoriness on the demands [127].

Following this 'dominant' slices approach, for each subject (an AI system) we get five aggregated points (1 to 5) in a one-dimensional space such as those represented in Figure 6 and Supplementary Figures 34 and 22, where the x-axis is the demand level and the y-axis is the percentage correct. However, we want to fit a curve out of these points. For this, we use a two-parametric logistic fit (with $y \in [0, 1]$), making all bins weight the same in the fit as the largest one (except those bins with less than 100 instances, which uses a proportional weight for robustness), with an anchor[3] of 0 at imaginary level 20 (we assume performance decreases to 0 at that level and beyond, which is plausible taking into account the meaning of levels in our rubric). This leads to monotonically decreasing curves. Note that the starting point of these curves at level 0 is not necessarily 100% accuracy, and in some cases it is much lower than that (e.g., this happens frequently for Supplementary Figure 34 and less so for Supplementary Figure 22). This makes sense as even if this would represent instances with all demands being zero (which we do not have in the battery), for weak subjects (early LLMs), there is not enough instructability to answer correctly on these simplest questions having no demands. The LLMs are not good enough. In a way, the value at 0 could be considered as some kind of base reliability of the system, independent of the demands.

Finally, once we have estimated the curve, we have the slope and the position as the two estimated parameters, as in any other IRT 2-PL psychometric model[4] [152, 157]. The slope indicates how predictive the dimension is for the performance of the items represented in the curve (only those for which that dimension dominates). A maximum (vertical) slope would mean that the dimension is very predictive and would sharply distinguish between a high probability of success at the beginning of the curve and a low probability of success from that inflection point. In general, we see some cases with moderate slopes, and some other cases with flatter curves. Finally, the position of the curve is the value on the x-axis of this maximum-slope point. The more to the right the curve descends, the higher the ability of the subject. Then, using common psychometric practice, we call this position 'ability', the point of maximum slope of the logistic curve, which is also the point where the probability of success equals 0.5 [158]. This is important to remember, because an ability of 4 does not mean the subject succeeds at most of the items of difficult 4, but only on half of them on expectation. Finally, given a logistic curve starting at $x_i = 0$ and a value of y close to 100% it is easy to see that the area under the curve (from $x = 0$ onwards) is equal to the ability. Because of this, and to make the definition more extensible in the future for situations where we do not want to estimate a parametric curve, we simply define ability as the area under the curve. Also, this avoids having negative abilities, which is nonsensical in a ratio scale.

1.3. ADeLe-Light

To streamline our analyses while preserving the representativeness of the demand space, we perform a redundancy reduction on the entire ADeLe battery. To identify clusters of highly similar instances, we apply k-nearest neighbours for each instance x_i to find k=10 neighbours and calculate the Average Squared Distance (ASD) between x_i and its 10 neighbours. The ASD serves as a proxy for local density in the multidimensional demand space: a lower ASD indicates that an instance is largely redundant with its neighbours. In our procedure, we mark an instance as redundant if its ASD falls below a threshold of 0.21. For these low-variability instances, we randomly remove 90% of the data, which reduces approximately 62% of redundant data, resulting in a sample of 6,179 instances, forming the ADeLe-Light battery v.1.0. The distributions of demands in the entire ADeLe-Light battery (Supplementary Figure 2) and the inferred ability profiles of all subject LLMs (Supplementary Figure 1) are similar to those obtained from the full ADeLe battery.

In general, ADeLe-Light can be used as a starting point when evaluating very complex (and costly) AI systems or many models, and especially for making engineering decisions when profiling different stages of development. For the final results, we always recommend to use of the full ADeLe battery.

[3]We assign it a weight that is equivalent to the 50% of the total weight shared between all bins and the anchor.

[4]The 2-PL model is usually applied to the items, and not to the subjects, although some models exist [155, 156]. Note that we do not include a guess parameter. We have an `Unguessability` dimension instead.

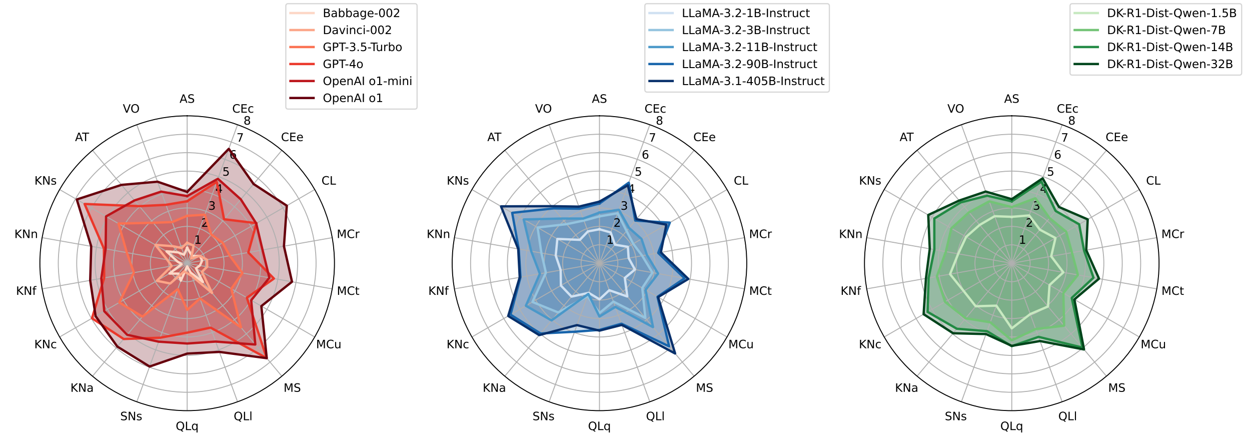

Supplementary Figure 1: Ability profiles of the 15 LLMs estimated using all the 6,179 instances in the ADeLe-Light battery v.1.0. Interpretation as in Figure 4.

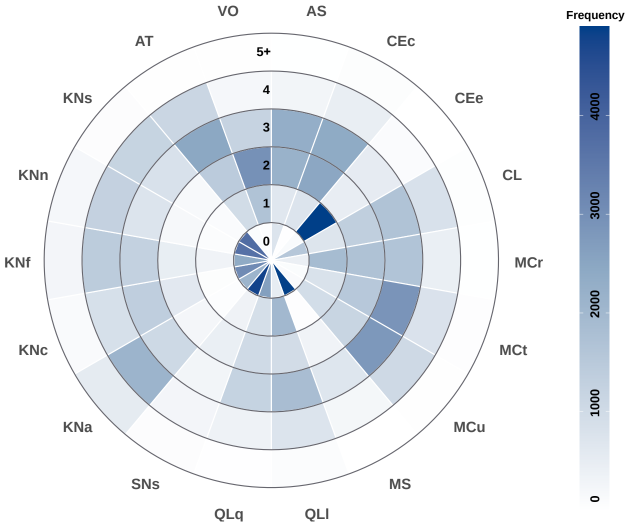

Supplementary Figure 2: Histograms of level frequencies for the 18 demands using all the 6179 instances in the ADeLe-Light battery v.1.0. Comparing with the distribution of the full ADeLe (Extended Data Figure 2), the distributions are similar.

1.4. Scaling Curves of Model Abilities

Supplementary Figures 3 and 4 show the scaling curves of model abilities and performance, respectively, as a function of model size, for both LLaMA and DK-R1-Distilled-Qwen families.

Traditional performance scaling analyses, such as those shown in Figure 4, which aggregates results across 20 benchmarks, are susceptible to saturation effects, not only because the scale on y-axis is bounded by 1 but also because there may be some abstruse or even wrongly labelled questions that make the percentages never reach 100%. For the most powerful models, the composite performance scores flatten across many benchmarks, making it difficult to interpret incremental improvements as model size increases. This saturation can mask subtle but important gains in specific cognitive abilities. In contrast, our ability scaling curves based on ratio scales remain sensitive and informative: they avoid benchmark saturation and show clear trends even for the largest models. This discloses insights, but the most notable one is the clear diminishing return from the second largest to the largest model in both LLaMA and DK-R1-Distilled-Qwen families, consistent in nearly all dimensions. In other words, while performance generally increases with model size, our method reveals that the magnitude of skill improvement tapers off beyond a certain size.

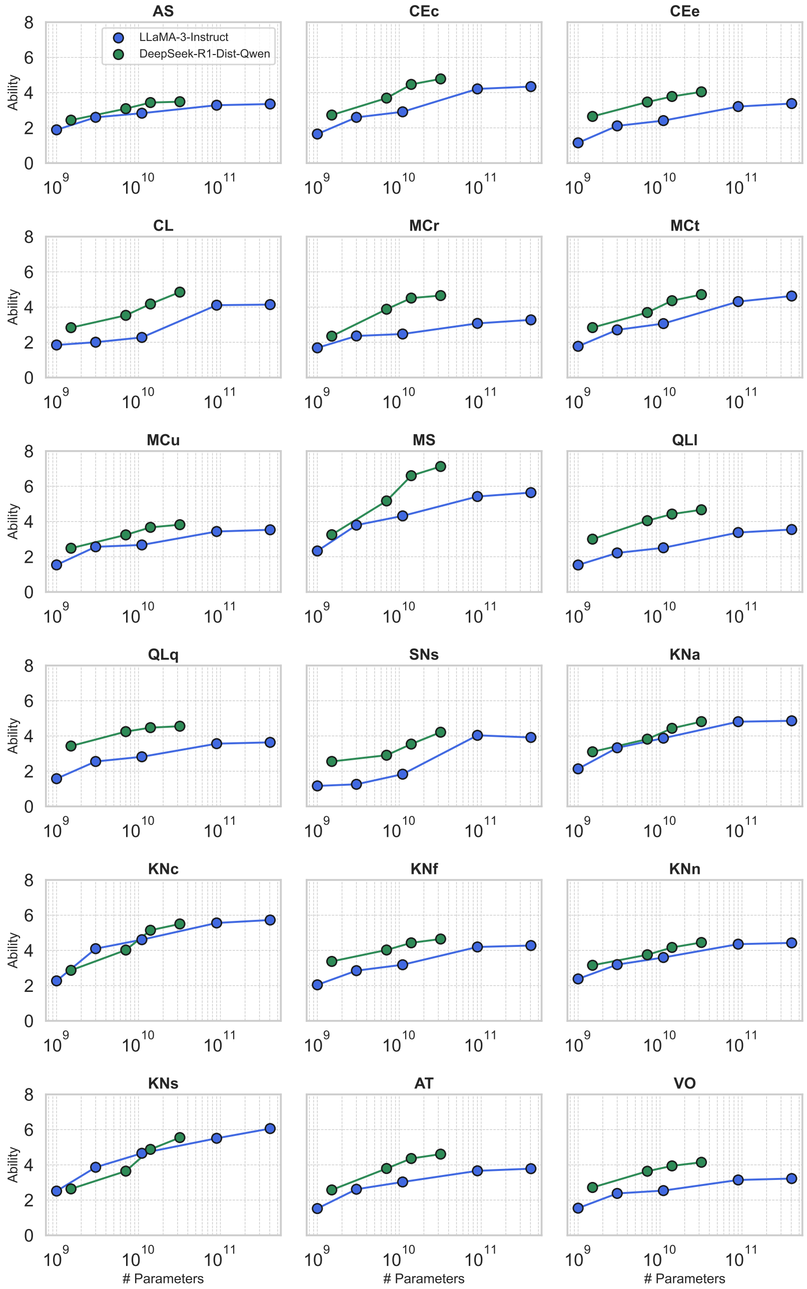

Supplementary Figure 3: The scaling curves of actual abilities for LLaMA and DK-R1-Distilled-Qwen families across all 18 demands.

1.5. Calibration of Assessors

Supplementary Figure 5, 6, 7 show the calibration of our demand-based, GLOVE and LLAMA assessors (from Extended Data Table 2) on the in-distribution setup. This confirms our observation in the Results section: the demand-based assessor achieves nearly perfect calibration, while the two black-box assessors are noticeably worse.

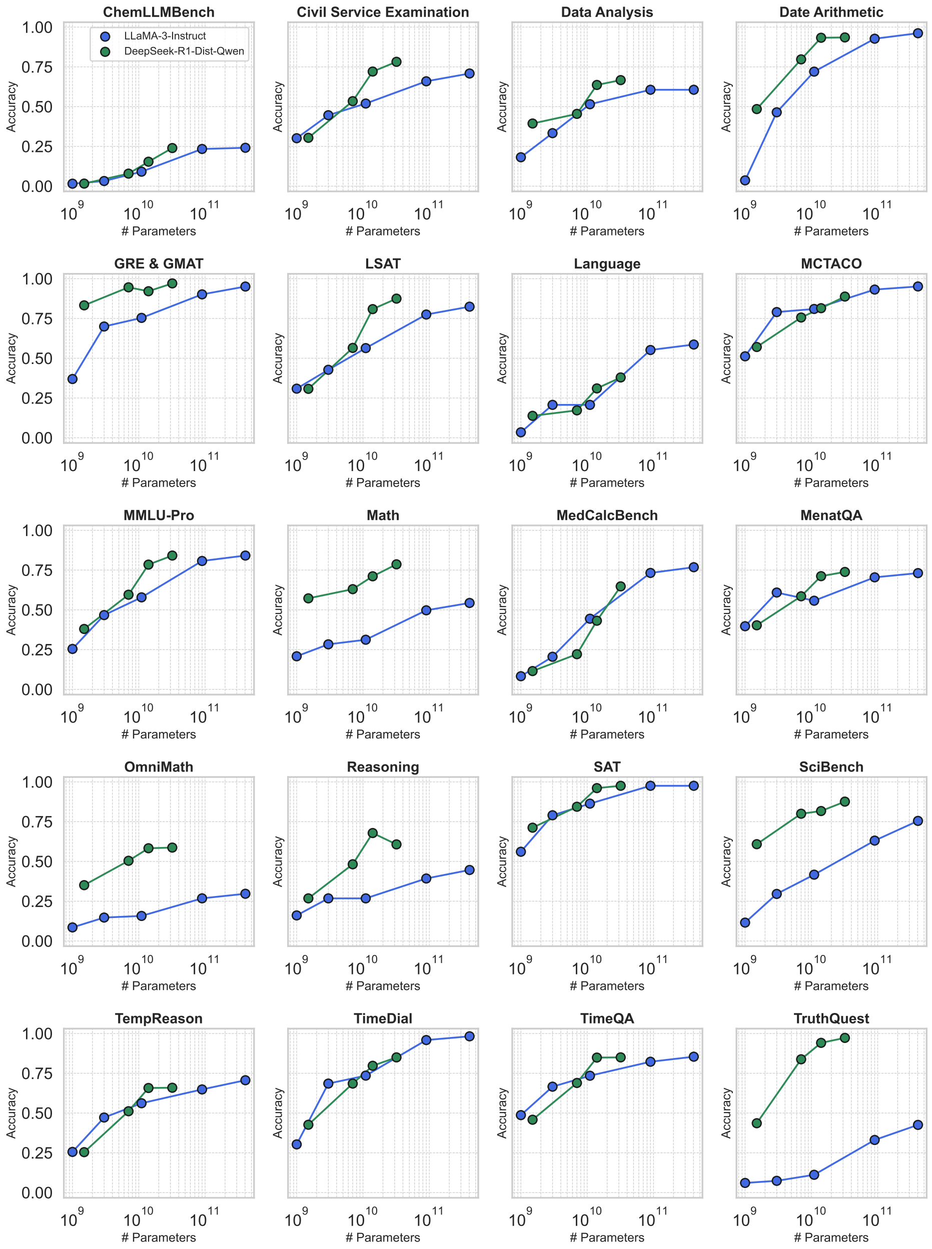

Supplementary Figure 4: The scaling curves of performance for LLaMA and DK-R1-Distilled-Qwen families across all 20 benchmarks.

1.6. Sources of Unpredictability

There are various sources that hinder perfect predictability more broadly. This includes both epistemic uncertainty, which can be reduced by additionally finding relevant predictive features (e.g., including new features or extending the demand level of 5+ in our DeLeAn rubrics to level 6 and beyond), and aleatoric uncertainty, which is inherently irreducible [159]. The latter may come from, but not limited to, chance guess rate in multiple-choice questions, memorisation of test instances, inherent stochasticity of reasoning models or models based on the Mixture-of-Experts architecture, prompt sensitivity, among others.

The first two sources of aleatoric uncertainty listed above, chance guess rate and memorisation, stem from data. The impact of these seems quite large, as supported by the analysis in Supplementary Information 1.7.2, in which

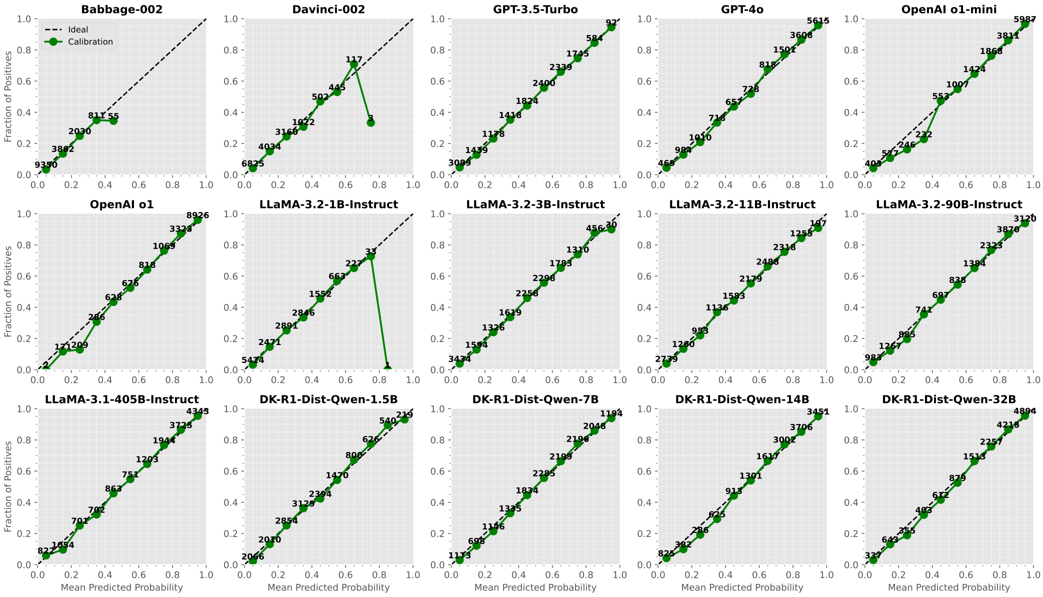

Supplementary Figure 5: Calibration of the RF demand-based assessor for the 15 subject LLMs, in-distribution, from Extended Data Table 2. The *x*-axis corresponds to the estimated probability of success, while the *y*-axis corresponds to the empirical performance. The number of instances in each bin are shown.

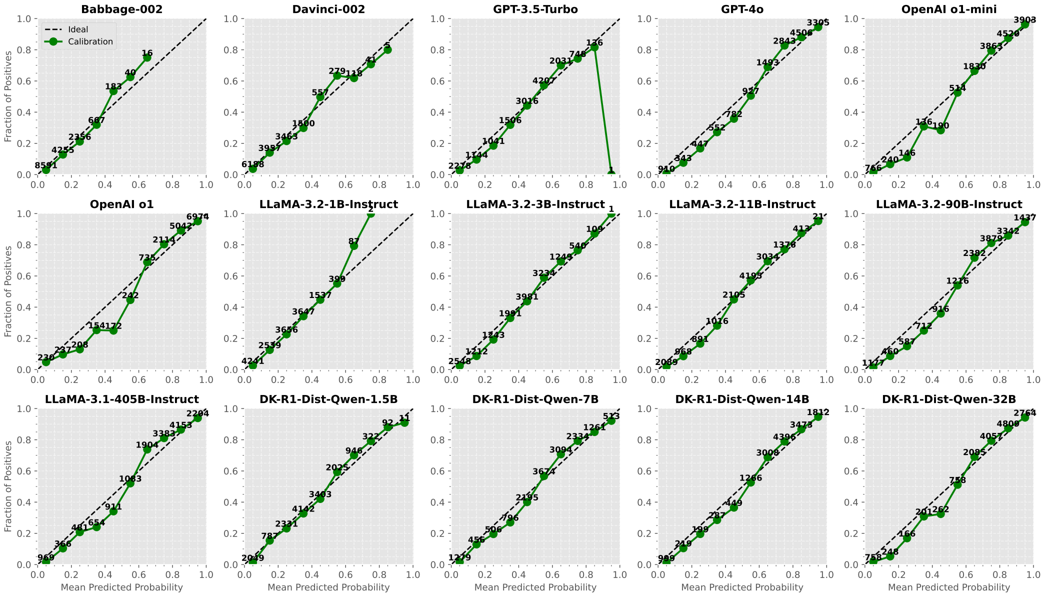

Supplementary Figure 6: Calibration of the GloVe assessor for the 15 subject LLMs, in-distribution, from Extended Data Table 2. The *x*-axis corresponds to the estimated probability of success, while the *y*-axis corresponds to the empirical performance. The number of instances in each bin are shown.

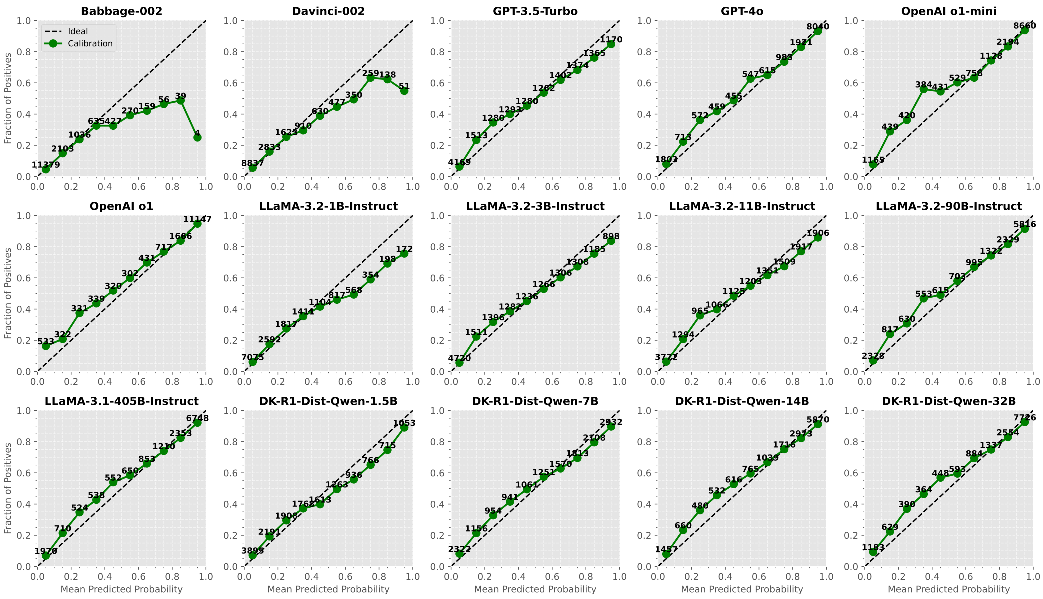

Supplementary Figure 7: Calibration of the LLAMA assessor for the 15 subject LLMs, in-distribution, from Extended Data Table 2. The x-axis corresponds to the estimated probability of success, while the y-axis corresponds to the empirical performance. The number of instances in each bin are shown.

we build a demand-based assessor by only using `AT` (`Atypicality`), `VO` (`Volume`) and `UG` (`Unguessability`); future research should seek solutions for controlling and minimising these sources of unpredictability coming from the data. The other two sources of aleatoric uncertainty come from the high-quality but imperfect input-response graders (see Methods) and demand annotators (see Methods). In other words, the predictive power of the demand-based assessors shown in this paper is in fact underrated, as the imperfect verification and demand annotations introduce noise and thus decrease the upper bound of predictive power that can be accomplished. This is unfortunate but encouraging, as it simultaneously implies that the predictive power of assessors based on our methodology will increase as future LLMs improve their capabilities.

In contrast, the other sources of unpredictability stem from the design of AI systems. For instance, many LLMs today are not deterministic, even at temperature 0, and chain-of-thought LLMs usually use a temperature well above 0). Prompt sensitivity has been reduced in recent models but it is still a source of unpredictability, as two very similar phrasings of a question that lead to different answers would typically be annotated with the same levels. future efforts should look for pathways that enable the minimisation of inherent unpredictability of models, as previously suggested by Zhou et al. [77], alongside a discussion of various possible future pathways for striving toward this goal.

1.7. Ablations and Other Predictive Models

1.7.1. Feature Importance

Supplementary Figure 8 shows the feature importance[5] of the 19 dimensions for all the demand-based assessors we have trained for Extended Data Table 2. We see that no demand has a feature importance value below 0.02, suggesting all demands are relevant to a greater or lesser extent. Based on the elbow method, we can select the six most relevant dimensions: `MCu` (`Calibrating Known Unknowns`), `UG` (`Unguessability`), `KNf` (`Knowledge of Formal Sciences`), `CL` (`Conceptualisation, Learning and Abstraction`), `QLl` (`Logical Reasoning`), and `MCr` (`Identifying Relevant Information`). This ranking is strongly correlated (Spearman Corr. = 0.718) with the ranking of correlation magnitudes between demands and success, as observed in Extended Data Figure 1.

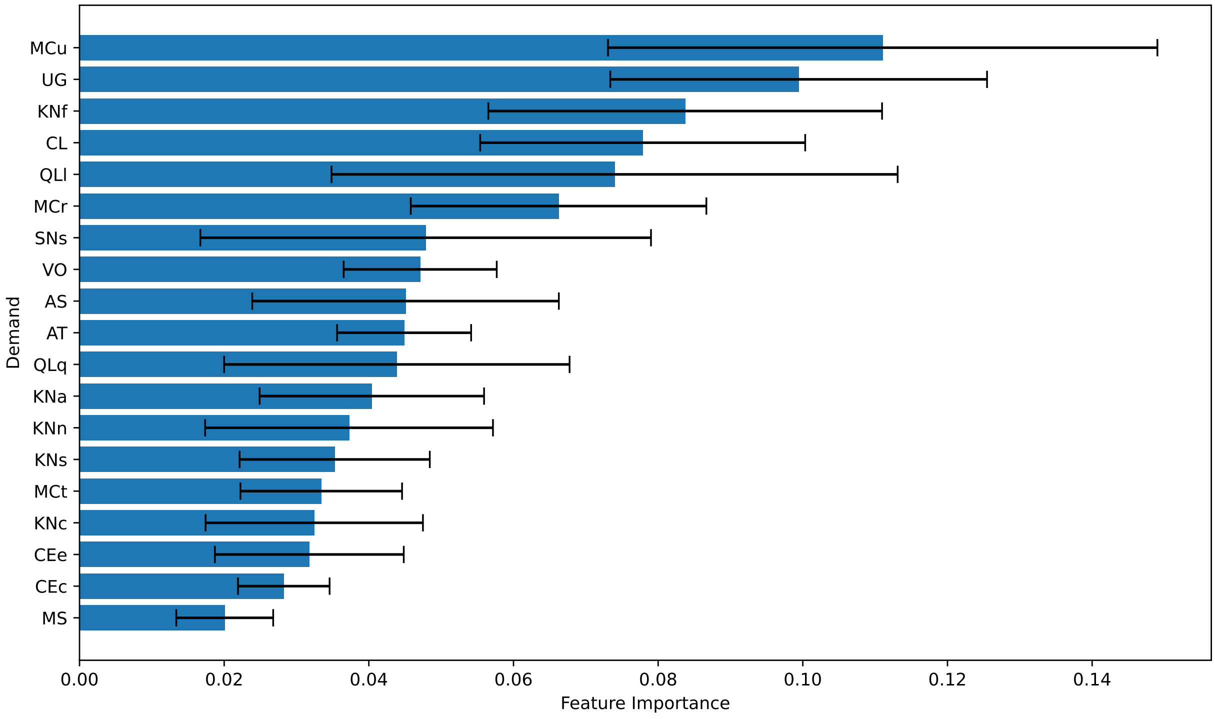

Supplementary Figure 8: Feature importance of all dimensions averaged across all demand-based assessors for different subject LLMs.

To explore whether it is possible to achieve high predictive power with a smaller number of dimensions, we feed the selection from the elbow method as input to the demand-based assessor using the same algorithm (Random Forest). We observe that predictive power in general decreases between 0.01 and 0.03 on average, but still fairly high (Supplementary Table 1) . This is promising as it implies that high predictive power may be achieved through a small number of dimensions.

1.7.2. Assessor Only with `AT`*,* `UG`*, and* `VO`

Supplementary Table 2 shows that a demand-based assessor fed only with `AT` (`Atypicality`), `UG` (`Unguessability`), `VO` (`Volume`) can achieve fairly good predictive power. This demonstrates that existing benchmarks incorporate extraneous demands in many task instances, identifying contamination, amalgamation and funnelling, respectively, that are predictive of a large proportion of LLM success. These are extraneous interventions in popular AI benchmarks that make them not measure what they claim they measure and should be controlled in the design of future benchmarks to ensure construct validity.

[5]We use the 'permutation_importance' in https://scikit-learn.org/stable/modules/generated/sklearn.inspection.html. It assesses each feature's importance by shuffling the feature's values and measuring the model's performance change.

Supplementary Table 1: Predictive power of the demand-based assessor by using only `MCu` (`Calibrating Known Unknowns`), `UG` (`Unguessability`), `KNf` (`Knowledge of Formal Sciences`), `CL` (`Conceptualisation, Learning and Abstraction`), `QLl` (`Logical Reasoning`), and `MCr` (`Identifying Relevant Information`) for in-distribution, task out-of-distribution, and benchmark out-of-distribution.

Subject LLM	LLM Accuracy↑	ID		Task OOD		Benchmark OOD	
		AUROC↑	ECE↓	AUROC↑	ECE↓	AUROC↑	ECE↓
Babbage-002	0.102	0.761	0.009	0.726	0.019	0.714	0.024
Davinci-002	0.157	0.751	0.007	0.724	0.019	0.696	0.039
GPT-3.5-turbo	0.414	0.792	0.012	0.773	0.026	0.767	0.024
GPT-4o	0.713	0.858	0.013	0.827	0.039	0.814	0.059
OpenAI o1-mini	0.770	0.830	0.012	0.800	0.032	0.734	0.051
OpenAI o1	0.843	0.815	0.010	0.777	0.032	0.737	0.043
LLaMA-3.2-1B-Instruct	0.216	0.768	0.011	0.728	0.034	0.711	0.048
LLaMA-3.2-3B-Instruct	0.378	0.794	0.009	0.779	0.027	0.757	0.042
LLaMA-3.2-11B-Instruct	0.463	0.801	0.010	0.780	0.041	0.768	0.036
LLaMA-3.2-90B-Instruct	0.645	0.837	0.015	0.807	0.033	0.795	0.072
LLaMA-3.1-405B-Instruct	0.683	0.846	0.010	0.814	0.037	0.797	0.077
DK-R1-Dist-Qwen-1.5B	0.353	0.753	0.010	0.733	0.023	0.681	0.053
DK-R1-Dist-Qwen-7B	0.555	0.781	0.013	0.762	0.017	0.666	0.053
DK-R1-Dist-Qwen-14B	0.698	0.797	0.012	0.774	0.030	0.691	0.071
DK-R1-Dist-Qwen-32B	0.748	0.809	0.013	0.785	0.036	0.714	0.075
Weighted Average	—	0.811	0.012	0.784	0.031	0.742	0.056

Supplementary Table 2: Predictive power of the demand-based assessor by using only `AT` (`Atypicality`), `UG` (`Unguessability`) and `VO` (`Volume`) for in-distribution, task out-of-distribution, and benchmark out-of-distribution.

Subject LLM	LLM Accuracy↑	ID		Task OOD		Benchmark OOD	
		AUROC↑	ECE↓	AUROC↑	ECE↓	AUROC↑	ECE↓
Babbage-002	0.102	0.676	0.007	0.590	0.043	0.548	0.070
Davinci-002	0.157	0.669	0.006	0.615	0.039	0.584	0.078
GPT-3.5-Turbo	0.414	0.748	0.009	0.720	0.041	0.670	0.096
GPT-4o	0.713	0.809	0.004	0.773	0.050	0.741	0.048
OpenAI o1-mini	0.770	0.738	0.005	0.693	0.038	0.620	0.082
OpenAI o1	0.843	0.727	0.004	0.672	0.028	0.598	0.086
LLaMA-3.2-1B-Instruct	0.216	0.707	0.007	0.652	0.052	0.618	0.101
LLaMA-3.2-3B-Instruct	0.378	0.738	0.008	0.706	0.030	0.650	0.130
LLaMA-3.2-11B-Instruction	0.463	0.748	0.006	0.717	0.053	0.682	0.076
LLaMA-3.2-90B-Instruct	0.645	0.792	0.007	0.758	0.041	0.727	0.070
LLaMA-3.1-405B-Instruct	0.683	0.799	0.008	0.763	0.065	0.739	0.069
DK-R1-Dist-Qwen-1.5B	0.353	0.685	0.007	0.657	0.033	0.569	0.122
DK-R1-Dist-Qwen-7B	0.555	0.686	0.007	0.645	0.054	0.568	0.120
DK-R1-Dist-Qwen-14B	0.698	0.713	0.006	0.664	0.063	0.584	0.114
DK-R1-Dist-Qwen-32B	0.748	0.730	0.007	0.692	0.039	0.608	0.101
Weighted Average	—	0.742	0.006	0.701	0.045	0.644	0.089

1.7.3. Assessor without `AT`*,* `UG`*, and* `VO`

Supplementary Table 3 demonstrates that the predictive power of a demand-based assessor fed with all the dimensions except for the extraneous ones, can achieve satisfactory results, though worse to a small extent than the demand-based assessor fed with all the dimensions, as shown previously in Extended Data Table 2, 3 and 4. This means that some other variables can compensate for this, including `unguessability`. This suggests that fully controlling for these variables could be done with some hierarchical assessors or, possibly, by performing a multivariate analysis.

1.7.4. Feature Grouping: Assessor with 11 Broad Dimensions

Supplementary Table 4 shows the predictive power of our demand-based assessor using the 11 broad dimensions listed in Table 7 instead of the 19 specific dimensions. Overall, this is slightly worse than using all 19 demands in general, again confirming that that one can predict model performance very well by aggregating some dimensions. Whether this compensates for robustness (having three scales for metacognition aggregated into one single scale) instead of one single scale for metacognition is something to explore in future work.

Supplementary Table 3: Predictive power of the demand-based assessor using all the demands expect for `AT` (`Atypicality`), `UG` (`Unguessability`) and `VO` (`Volume`), for in-distribution, task out-of-distribution, and benchmark out-of-distribution.

Subject LLM	LLM Accuracy↑	ID		Task OOD		Benchmark OOD	
		AUROC↑	ECE↓	AUROC↑	ECE↓	AUROC↑	ECE↓
Babbage-002	0.102	0.775	0.004	0.737	0.010	0.664	0.033
Davinci-002	0.157	0.763	0.008	0.732	0.011	0.715	0.015
GPT-3.5-turbo	0.414	0.802	0.005	0.783	0.019	0.753	0.053
GPT-4o	0.713	0.871	0.013	0.841	0.022	0.790	0.041
OpenAI o1-mini	0.770	0.848	0.007	0.827	0.017	0.736	0.027
OpenAI o1	0.843	0.839	0.009	0.796	0.026	0.705	0.031
LLaMA-3.2-1B-Instruct	0.216	0.771	0.004	0.722	0.031	0.698	0.053
LLaMA-3.2-3B-Instruct	0.378	0.804	0.008	0.780	0.022	0.752	0.050
LLaMA-3.2-11B-Instruct	0.463	0.810	0.008	0.788	0.028	0.757	0.051
LLaMA-3.2-90B-Instruct	0.645	0.848	0.014	0.816	0.027	0.759	0.055
LLaMA-3.1-405B-Instruct	0.683	0.859	0.011	0.828	0.024	0.770	0.050
DK-R1-Dist-Qwen-1.5B	0.353	0.772	0.009	0.745	0.028	0.702	0.043
DK-R1-Dist-Qwen-7B	0.555	0.802	0.013	0.778	0.020	0.689	0.043
DK-R1-Dist-Qwen-14B	0.698	0.815	0.012	0.792	0.014	0.681	0.033
DK-R1-Dist-Qwen-32B	0.748	0.827	0.010	0.795	0.029	0.709	0.046
Weighted Average	—	0.827	0.010	0.798	0.022	0.731	0.042

Supplementary Table 4: Predictive power of the demand-based assessor by using the 11 broad dimensions for in-distribution, task out-of-distribution, and benchmark out-of-distribution. If a broad dimension has more than one subdimensions, we take the maximum value.

Subject LLM	LLM Accuracy↑	ID		Task OOD		Benchmark OOD	
		AUROC↑	ECE↓	AUROC↑	ECE↓	AUROC↑	ECE↓
Babbage-002	0.102	0.768	0.004	0.731	0.014	0.686	0.020
Davinci-002	0.157	0.763	0.007	0.727	0.011	0.712	0.021
GPT-35-Turbo	0.414	0.801	0.007	0.784	0.021	0.768	0.032
GPT-4o	0.713	0.870	0.013	0.839	0.025	0.816	0.048
OpenAI o1-mini	0.770	0.845	0.013	0.821	0.020	0.745	0.029
OpenAI o1	0.843	0.835	0.011	0.793	0.031	0.725	0.039
LLaMA-3.2-1B-Instruct	0.216	0.769	0.010	0.718	0.034	0.692	0.035
LLaMA-3.2-3B-Instruct	0.378	0.803	0.010	0.782	0.026	0.768	0.032
LLaMA-3.2-11B-Instruct	0.463	0.808	0.008	0.786	0.033	0.773	0.045
LLaMA-3.2-90B-Instruct	0.645	0.849	0.014	0.819	0.030	0.795	0.061
LLaMA-3.1-405B-Instruct	0.683	0.858	0.011	0.830	0.029	0.810	0.049
DK-R1-Dist-Qwen-1.5B	0.353	0.768	0.007	0.746	0.018	0.691	0.053
DK-R1-Dist-Qwen-7B	0.555	0.799	0.013	0.775	0.022	0.682	0.031
DK-R1-Dist-Qwen-14B	0.698	0.815	0.012	0.793	0.024	0.701	0.038
DK-R1-Dist-Qwen-32B	0.748	0.825	0.011	0.798	0.028	0.704	0.053
Weighted Average	—	0.825	0.011	0.797	0.026	0.745	0.042

1.7.5. Demand-based Assessor with Logistic Regression

Supplementary Table 5 shows the predictive power of an demand-based assessor based on logistic regression, using all the 19 demands, without any hyperparameters tuning. In comparison with our main demand-based assessor in Extended Data Table 2, this assessor is moderately worse (e.g. dropping from 0.881 to 0.852 in terms AUROC when predicting GPT-4o in the in-distribution setup), which is expected since this logistic regression model is expressed in the simplest form—using only raw features and does not capture any nonlinear (or interaction) terms into the feature set, leading its decision boundary to remain linear, unlike random forest.

1.7.6. A Universal Assessor

Supplementary Table 6 shows the predictive power of a demand-based assessor trained with all the 19 dimensions plus one extra variable with the identifier of the LLM subjects. Even if the dataset is 15 times larger and it can smooth the aleatoric uncertainty specific to some models, the results are very similar to the standard configuration with one assessor per model. Things could improve with a characterisation of the LLM or some other techniques, but we leave this as future work.

Supplementary Table 5: Predictive power of a demand-based assessor trained with a basic logistic regression by using all the 19 specific dimensions for in-distribution, task out-of-distribution, and benchmark out-of-distribution.

Subject LLM	LLM Accuracy↑	ID		Task OOD		Benchmark OOD	
		AUROC↑	ECE↓	AUROC↑	ECE↓	AUROC↑	ECE↓
Babbage-002	0.102	0.749	0.010	0.725	0.016	0.705	0.018
Davinci-002	0.157	0.740	0.005	0.717	0.014	0.709	0.023
GPT-3.5-Turbo	0.414	0.793	0.020	0.784	0.030	0.776	0.032
GPT-4o	0.713	0.852	0.018	0.833	0.033	0.814	0.032
OpenAI o1-mini	0.770	0.820	0.013	0.798	0.017	0.695	0.069
OpenAI o1	0.843	0.796	0.020	0.760	0.029	0.607	0.081
LLaMA-3.2-1B-Instruct	0.216	0.748	0.016	0.722	0.039	0.716	0.041
LLaMA-3.2-3B-Instruct	0.378	0.788	0.025	0.778	0.033	0.768	0.031
LLaMA-3.2-11B-Instruction	0.463	0.799	0.026	0.789	0.038	0.782	0.036
LLaMA-3.2-90B-Instruct	0.645	0.828	0.022	0.808	0.035	0.788	0.033
LLaMA-3.1-405B-Instruct	0.683	0.840	0.018	0.823	0.031	0.801	0.032
DK-R1-Dist-Qwen-1.5B	0.353	0.746	0.019	0.733	0.029	0.694	0.039
DK-R1-Dist-Qwen-7B	0.555	0.767	0.020	0.747	0.031	0.640	0.087
DK-R1-Dist-Qwen-14B	0.698	0.781	0.025	0.761	0.043	0.612	0.114
DK-R1-Dist-Qwen-32B	0.748	0.797	0.026	0.776	0.042	0.628	0.099
Weighted Average	—	0.801	0.020	0.781	0.032	0.710	0.059

Supplementary Table 6: Predictive power of the demand-based assessor by using only `MCu` (`Calibrating Known Unknowns`), `UG` (`Unguessability`), `KNf` (`Knowledge of Formal Sciences`), `CL` (`Conceptualisation, Learning and Abstraction`), `QLl` (`Logical Reasoning`), and `MCr` (`Identifying Relevant Information`) for in-distribution, task out-of-distribution, and benchmark out-of-distribution.

Subject LLM	LLM Accuracy↑	ID		Task OOD		Benchmark OOD	
		AUROC↑	ECE↓	AUROC↑	ECE↓	AUROC↑	ECE↓
Babbage-002	0.102	0.785	0.013	0.750	0.015	0.715	0.031
Davinci-002	0.157	0.772	0.013	0.737	0.020	0.698	0.032
GPT-3.5-turbo	0.414	0.812	0.019	0.796	0.027	0.774	0.044
GPT-4o	0.713	0.883	0.019	0.857	0.027	0.799	0.045
OpenAI o1-mini	0.770	0.860	0.018	0.839	0.029	0.720	0.037
OpenAI o1	0.843	0.851	0.018	0.810	0.040	0.671	0.043
LLaMA-3.2-1B-Instruct	0.216	0.786	0.018	0.732	0.036	0.714	0.058
LLaMA-3.2-3B-Instruct	0.378	0.813	0.021	0.790	0.032	0.774	0.043
LLaMA-3.2-11B-Instruct	0.463	0.820	0.018	0.802	0.021	0.781	0.055
LLaMA-3.2-90B-Instruct	0.645	0.861	0.025	0.837	0.025	0.781	0.060
LLaMA-3.1-405B-Instruct	0.683	0.870	0.020	0.846	0.029	0.792	0.056
DK-R1-Dist-Qwen-1.5B	0.353	0.783	0.030	0.755	0.037	0.707	0.069
DK-R1-Dist-Qwen-7B	0.555	0.812	0.027	0.785	0.023	0.691	0.028
DK-R1-Dist-Qwen-14B	0.698	0.828	0.020	0.808	0.025	0.693	0.061
DK-R1-Dist-Qwen-32B	0.748	0.841	0.019	0.815	0.031	0.701	0.057
Weighted Average	—	0.838	0.020	0.812	0.029	0.735	0.049

1.7.7. Algebraic Assessor

Comparing the demand profile of a task instance and the ability profile of an AI system gives immediate insights on the expectation of success. Actually, the ability comes from the interpretation of a characteristic curve, which plots probability of success as a function of the demand level. Can we use this without the need of training any model? This is what we refer to as an algebraic or formulaic assessor. There are many possible ways of doing this, but the main issue is to reconstruct the multidimensional characteristic surface out of the unidimensional curves.

We will illustrate one approach that is straightforward and hence interpretable, but many other options could be explored. To build this algebraic assessor, we first compute the differences between the subject's ability, a_i, and the task demand, d_i for all 18 demands. While we could apply the original two-parametric logistic function (slope and position), for simplicity we assume we only have access to the ability (the area or position of the curve) and we apply the standard logistic function. This gives us a univariate probability per each demand, i.e., we apply

$$\sigma(x) = \frac{1}{1 + \exp(-x)},$$

to each $a_i - d_i$, which maps the values into $[0, 1]$. In addition, we add `UG` (`Unguessability`), which is normalised as

$(100 - UG)/100$, ensuring that it is also within [0, 1]. The prediction of LLM success is computed by combining these 19 values using a generalised mean:

$$S = \left(\frac{1}{19} \left(\sum_{i=1}^{18} [\sigma(a_i - d_i)]^r + \left(\frac{100 - UG}{100} \right)^r \right) \right)^{1/r}, \quad (1)$$

where r is a parameter that controls the type of mean used. Extreme negative values of r tend to approximate the minimum of all components, while extreme positive values tend to approximate the maximum of all components. The predictive power of this algebraic assessor is shown in Supplementary Table 7, using different values of r, including $r = 0$ (geometric mean), $r = 0.25$ and $r = 1$ (arithmetic mean). Overall, it seems that a value of $r = 0.25$ is the optimal, reaching satisfactory AUROC scores, especially for large models. The advantage of $r = 0.25$ and $r = 0$ (geometric mean) over arithmetic mean ($r = 1$) is sensible because the most informative probability scores in the generalised mean equation come from those dimensions i with more negative values in $a_i - d_i$ (e.g., if a_i is much smaller than d_i for one or a few dimensions, it is highly likely that the model will fail, even if the model's abilities on other dimensions are well above the demands). When comparing $r = 0.25$ and $r = 0$, we see that the geometric mean ($r = 0$) is also fairly good as it is often the best in terms of calibration, though clearly worse in AUROC than $r = 0.25$, indicating that the geometric mean (or a value of r below 0) may over-focus on one or very few dimensions that exhibit the most negative values in $a_i - d_i$. This also has an interpretation in terms of compensatoriness of the dimensions in the sense of multidimensional IRT (see related work, section 1.1).

Supplementary Table 7: Predictive power of the demand-based *algebraic* assessor for different values of r using the ADeLe demands. Bold numbers indicate the best value for each LLM (largest AUROC and lowest ECE) across the three r settings.

Subject LLM	LLM Accuracy↑	$r = 0$		$r = 0.25$		$r = 1$	
		AUROC↑	ECE↓	AUROC↑	ECE↓	AUROC↑	ECE↓
Babbage-002	0.102	0.607	**0.068**	**0.614**	0.138	0.581	0.230
Davinci-002	0.157	0.590	**0.105**	**0.652**	0.135	0.638	0.226
GPT-3.5-Turbo	0.414	0.744	**0.098**	**0.771**	0.122	0.752	0.203
GPT-4o	0.713	0.812	0.238	**0.826**	0.129	0.803	**0.105**
OpenAI o1-mini	0.770	0.739	0.267	**0.749**	**0.065**	0.746	0.079
OpenAI o1	0.843	0.733	0.312	**0.734**	0.071	0.706	**0.040**
LLaMA-3.2-1B-Instruct	0.216	0.693	**0.111**	**0.715**	0.177	0.696	0.266
LLaMA-3.2-3B-Instruct	0.378	0.739	**0.109**	**0.775**	0.152	0.756	0.235
LLaMA-3.2-11B-Instruct	0.463	0.749	**0.105**	**0.780**	0.112	0.762	0.193
LLaMA-3.2-90B-Instruct	0.645	0.791	0.188	**0.805**	0.127	0.780	**0.124**
LLaMA-3.1-405B-Instruct	0.683	0.803	0.216	**0.817**	0.128	0.793	**0.104**
DK-R1-Dist-Qwen-1.5B	0.353	0.655	**0.179**	**0.697**	0.199	0.691	0.283
DK-R1-Dist-Qwen-7B	0.555	0.665	0.210	0.699	**0.122**	**0.702**	0.203
DK-R1-Dist-Qwen-14B	0.698	0.720	0.213	**0.733**	**0.050**	0.730	0.114
DK-R1-Dist-Qwen-32B	0.748	0.739	0.247	**0.747**	**0.061**	0.734	0.088
Weighted Average	—	0.738	0.206	**0.757**	**0.106**	0.741	0.137

In general, this algebraic assessor, especially because the results are good for $r = 0$, suggests a simple mechanism to go from unidimensional abilities and demands to an integrated prediction that is based on all the multidimensional information of an instance: instance performance can be seen as the geometric mean of the expected performance for each dimension. Evidently, using some trained models, results can be better (especially in calibration) but Eq. 1 is a very formulaic, interpretable way of understanding how abilities and demands affect performance.

We also show the predictive power of two algebraic assessors trained using the annotations based on the 'Miller & Tang' and 'BLOOM' taxonomies [21, 22] which are defined in two distinct rubrics in Appendices 3.1 and 3.2 (Supplementary Tables 8 and 9). Here we observe that the predictive performance is significantly inferior to that of the algebraic assessor using our ADeLe framework (as shown in Supplementary Table 7).

1.7.8. Further Ablations for Univariate Predictive Power

Above, we presented several ablation results using meaningful selections of 3 (Supplementary Information 1.7.2), 6 (Supplementary Information 1.7.3) or 11 capabilities (Supplementary Information 1.7.4), following either the hierarchy or other elements of the catalogue. This subsection contains complementary ablation studies considering the full set of dimensions and several feature-selection criteria.

Supplementary Table 8: Predictive power of the demand-based *algebraic* assessor for different values of r on the Miller & Tang baseline. Bold numbers indicate the best value for each LLM (largest AUROC and lowest ECE) across the three r settings. The numbers here are comparable with Supplementary Table 7.

Subject LLM	LLM Accuracy↑	r = 0		r = 0.25		r = 1	
		AUROC↑	ECE↓	AUROC↑	ECE↓	AUROC↑	ECE↓
Babbage-002	0.102	**0.558**	**0.175**	0.552	0.241	0.458	0.333
Davinci-002	0.157	**0.500**	0.195	0.499	**0.194**	0.428	0.288
GPT-3.5-Turbo	0.414	0.648	0.179	**0.649**	**0.063**	0.572	0.076
GPT-4o	0.713	**0.700**	0.450	**0.700**	0.280	0.620	**0.159**
OpenAI o1-mini	0.770	**0.695**	0.505	**0.695**	0.332	0.691	**0.210**
OpenAI o1	0.843	**0.697**	0.570	**0.697**	0.389	0.669	**0.262**
LLaMA-3.2-1B-Instruct	0.216	**0.624**	**0.135**	0.623	0.139	0.549	0.235
LLaMA-3.2-3B-Instruct	0.378	0.646	0.149	**0.647**	**0.050**	0.570	0.106
LLaMA-3.2-11B-Instruct	0.463	0.648	0.223	**0.649**	**0.096**	0.563	0.037
LLaMA-3.2-90B-Instruct	0.645	**0.685**	0.389	**0.685**	0.222	0.605	**0.105**
LLaMA-3.1-405B-Instruct	0.683	**0.697**	0.423	**0.697**	0.254	0.616	**0.135**
DK-R1-Dist-Qwen-1.5B	0.353	0.621	0.164	**0.624**	**0.060**	0.646	0.117
DK-R1-Dist-Qwen-7B	0.555	0.634	0.311	0.634	0.156	**0.655**	**0.103**
DK-R1-Dist-Qwen-14B	0.699	**0.683**	0.441	**0.683**	0.274	0.680	**0.166**
DK-R1-Dist-Qwen-32B	0.748	**0.697**	0.485	**0.697**	0.314	0.682	**0.193**
Weighted Average	—	**0.671**	0.380	**0.671**	0.234	0.629	**0.159**

Supplementary Table 9: Predictive power of the demand-based *algebraic* assessor for different values of r on the BLOOM baseline. Bold numbers indicate the best value for each LLM (largest AUROC and lowest ECE) across the three r settings. The numbers here are comparable with Supplementary Table 7.

Subject LLM	LLM Accuracy↑	r = 0		r = 0.25		r = 1	
		AUROC↑	ECE↓	AUROC↑	ECE↓	AUROC↑	ECE↓
Babbage-002	0.102	0.611	0.068	0.616	0.060	**0.687**	**0.030**
Davinci-002	0.157	0.585	0.098	0.587	0.092	**0.677**	**0.049**
GPT-3.5-Turbo	0.414	**0.712**	0.322	**0.712**	0.308	0.702	**0.228**
GPT-4o	0.713	0.779	0.581	**0.780**	0.554	0.728	**0.406**
OpenAI o1-mini	0.770	**0.689**	0.633	0.688	0.603	0.650	**0.444**
OpenAI o1	0.843	**0.687**	0.697	0.686	0.663	0.613	**0.482**
LLaMA-3.2-1B-Instruct	0.216	0.676	0.148	0.676	0.139	**0.709**	**0.090**
LLaMA-3.2-3B-Instruct	0.378	0.716	0.290	0.718	0.276	**0.733**	**0.203**
LLaMA-3.2-11B-Instruct	0.463	**0.721**	0.363	**0.721**	0.347	0.717	**0.258**
LLaMA-3.2-90B-Instruct	0.645	0.759	0.521	**0.762**	0.497	0.722	**0.366**
LLaMA-3.1-405B-Instruct	0.683	0.770	0.554	**0.772**	0.529	0.725	**0.388**
DK-R1-Dist-Qwen-1.5B	0.353	0.599	0.272	**0.601**	0.261	0.593	**0.197**
DK-R1-Dist-Qwen-7B	0.555	**0.610**	0.446	0.607	0.427	0.604	**0.323**
DK-R1-Dist-Qwen-14B	0.698	**0.671**	0.569	**0.671**	0.543	0.644	**0.400**
DK-R1-Dist-Qwen-32B	0.748	**0.689**	0.613	**0.689**	0.584	0.640	**0.427**
Weighted Average	—	**0.700**	0.500	**0.700**	0.477	0.672	**0.350**

First, we use random forward feature inclusion, i.e., selecting one random dimension, then two random dimensions (not necessarily including the previous one), and so on, repeating this process across 10 seeds and calculating the average. The results can be seen in Supplementary Figures 9, 10 and 11 (one graph per family: OpenAI, Meta and DeepSeek). We see that, while the contribution of each new variable decreases as more are introduced, selecting a small number of features will result in a loss of predictive power.

An alternative experiment involves instead progressively selecting the most relevant features first. This is what we show in Supplementary Figures 12, 13 and 14 using best-feature selection[6] rather than random-feature selection. In this case, we see saturation of the performance, especially in OOD, beyond 8 features. This may be due to overfitting, and saturation could happen later with a larger training dataset or more powerful techniques. Also, as the saturation happens especially for OOD, it shows that the best features for some benchmarks may be different from those that would be required for other benchmarks.

Still, in the previously shown curves, we only see the number, but not whether the features are different according

[6]We use the 'permutation_importance' in https://scikit-learn.org/stable/modules/generated/sklearn.inspection.html. It assesses each feature's importance by shuffling the feature's values and measuring the model's performance change.

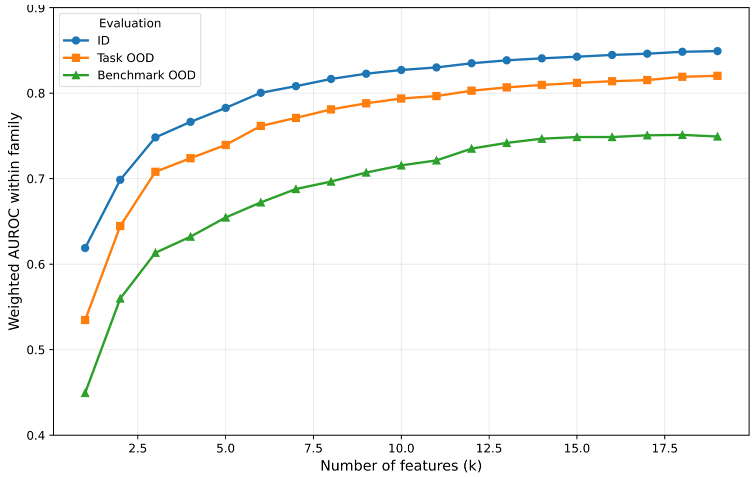

Supplementary Figure 9: Evolution of the weighted average of AUROC (weighted by subject accuracy) of the random forest assessor in predicting subject LLMs from the OpenAI family, as the number of input features (i.e., annotated dimensions) increases. Features are sampled randomly from the full set.

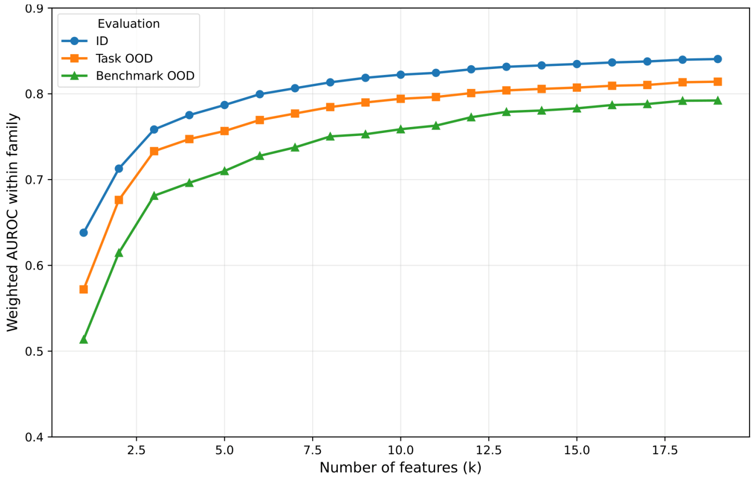

Supplementary Figure 10: Evolution of the weighted average of AUROC (weighted by subject accuracy) of the random forest assessor in predicting subject LLMs from the LLaMA family, as the number of input features (i.e., annotated dimensions) increases. Features are sampled randomly from the full set.

to the model family. This is what we see in Supplementary Table 10, where we build a random forest assessor per model family and list the top-10 most important features.

For instance, there are a few dimensions that are shared across families, `UG`, `MCu`, and `CL`. This reflects the importance of multiple-choice questions in the 2024 benchmarks, dealing with the metacognition of uncertainty (`UG` and `MCu`) and abstract reasoning and learning (`CL`). However, we see that some dimensions such as `QLI` or `VO` are relevant for some models but not for others. In fact, we see a significantly different list for DeepSeek-R1-Distilled. This supports our claim that selecting features based on a selection of benchmarks or models is likely a poor choice for other benchmarks or models, especially as there is no reaso to believe that such a selection would have predictive power, according to the previous plots (the only exception being OOD).

All of the above are ablations for the multivariate influence of the dimensions, but can we analyse the univariate effects? We have already included the correlations between the dimensions and the dependent success variable in Extended Data Figure 1 (the separate bar out of the matrix). This is not totally univariate, as many examples have medium or high values of several dimensions, due to the cross-influence of the variables in the `ADeLe` battery.

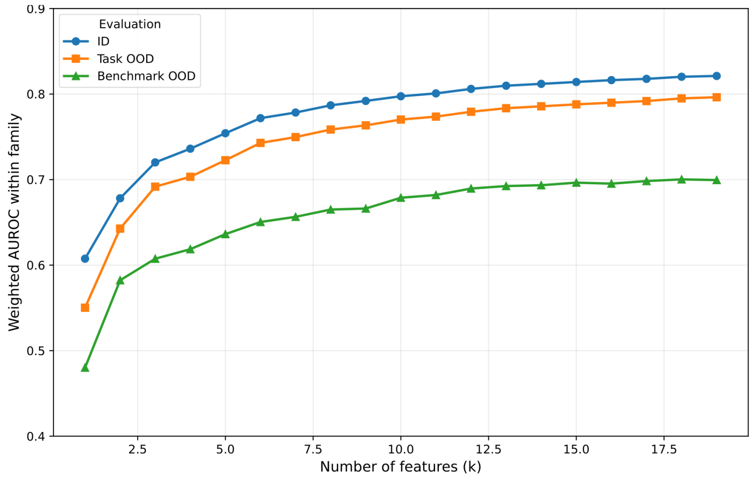

Supplementary Figure 11: Evolution of the weighted average of AUROC (weighted by subject accuracy) of the random forest assessor in predicting subject LLMs from the DeepSeek-R1-Distilled family, as the number of input features (i.e., annotated dimensions) increases. Features are sampled randomly from the full set.

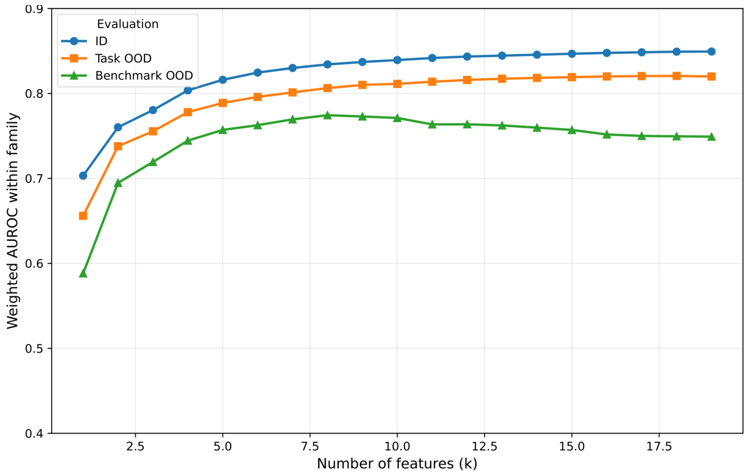

Supplementary Figure 12: Evolution of the weighted average of AUROC (weighted by subject accuracy) of the random forest assessor in predicting subject LLMs from the OpenAI family, as the number of input features (i.e., annotated dimensions) increases. Features are selected based on their feature importance – the one with the highest feature importance is chosen first whenever adding a new dimension.

Supplementary Table 10: Top-10 most important features for the random forest assessor across the three model families.

**Importance Rank**	**OpenAI**		**LLAMA**		**DeepSeek-R1-Distilled**	
	**Feature**	**Importance**	**Feature**	**Importance**	**Feature**	**Importance**
1	MCu	0.107	UG	0.128	MCu	0.143
2	UG	0.100	QLI	0.111	UG	0.082
3	CL	0.083	MCu	0.085	CL	0.073
4	KNf	0.077	CL	0.084	KNn	0.065
5	QLI	0.067	KNf	0.080	SNs	0.061
6	MCr	0.064	MCr	0.079	KNf	0.060
7	SNs	0.062	VO	0.050	AS	0.060
8	AT	0.051	AT	0.045	MCr	0.057
9	AS	0.046	CEe	0.039	KNa	0.056
10	VO	0.043	QLq	0.037	AT	0.052

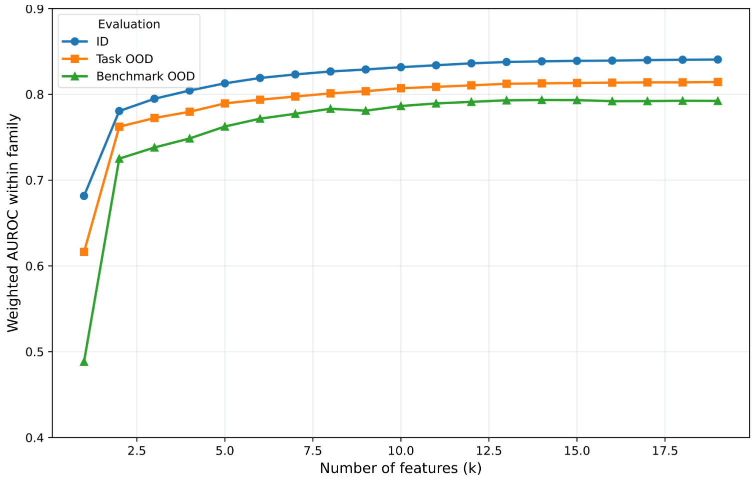

Supplementary Figure 13: Evolution of the weighted average of AUROC (weighted by subject accuracy) of the random forest assessor in predicting subject LLMs from the LLaMA family, as the number of input features (i.e., annotated dimensions) increases. Features are selected based on their feature importance – the one with the highest feature importance is chosen first whenever adding a new dimension.

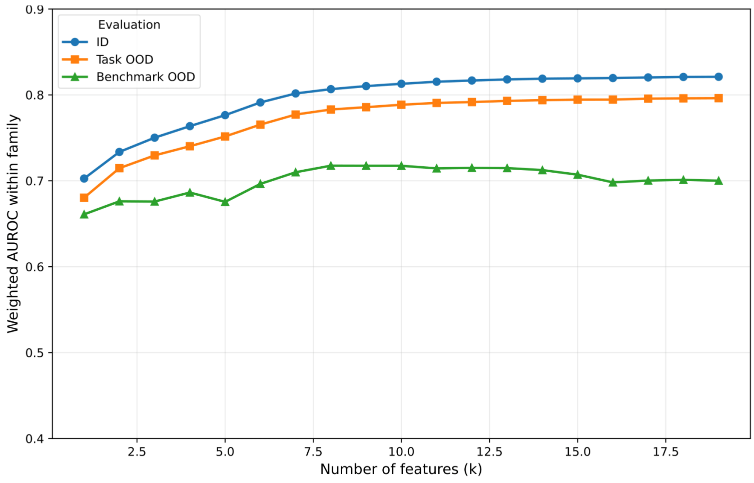

Supplementary Figure 14: Evolution of the weighted average of AUROC (weighted by subject accuracy) of the random forest assessor in predicting subject LLMs from the DeepSeek-R1-Distilled family, as the number of input features (i.e., annotated dimensions) increases. Features are selected based on their feature importance – the one with the highest feature importance is chosen first whenever adding a new dimension.

To provide a clearer analysis of each dimension, we now select those items where that dimension is dominant, i.e., the dimension's demand level of that dimension is no lower than that of any other dimension (see Supplementary Figure 15). Here, we see that all dimensions except for `SNs` have negative correlations, with 14 dimensions having an absolute value greater than 0.25. The dimensions with low correlations are spatio-physical reasoning (`SNs`), social science and humanities knowledge (`KNs`), mind modelling and social cognition (`MS`) and customary everyday knowledge (`KNc`). Nevertheless, this should not be a reason for eliminating them, as this is explained by the collected benchmarks being less loaded on these dimensions–these are among the top five dimensions with more zeros (see Extended Data Figure 2), and also being less represented by the number of instances shown in the SCCs in Figure 3.

1.8. DeepSeek-V3 and LLaMA-3.1-8b-instruct as Annotators

Instead of GPT-4o, we explore the use of DeepSeek-V3 and LLaMA-3.1-8b-instruct as annotators for demands, keeping all other things equal. Supplementary Table 11 shows their agreements with the Delphi consensus of the human annotators, where we observe an average r_{WG} of 0.83 for DeepSeek-V3 (only slightly worse than GPT-4o's

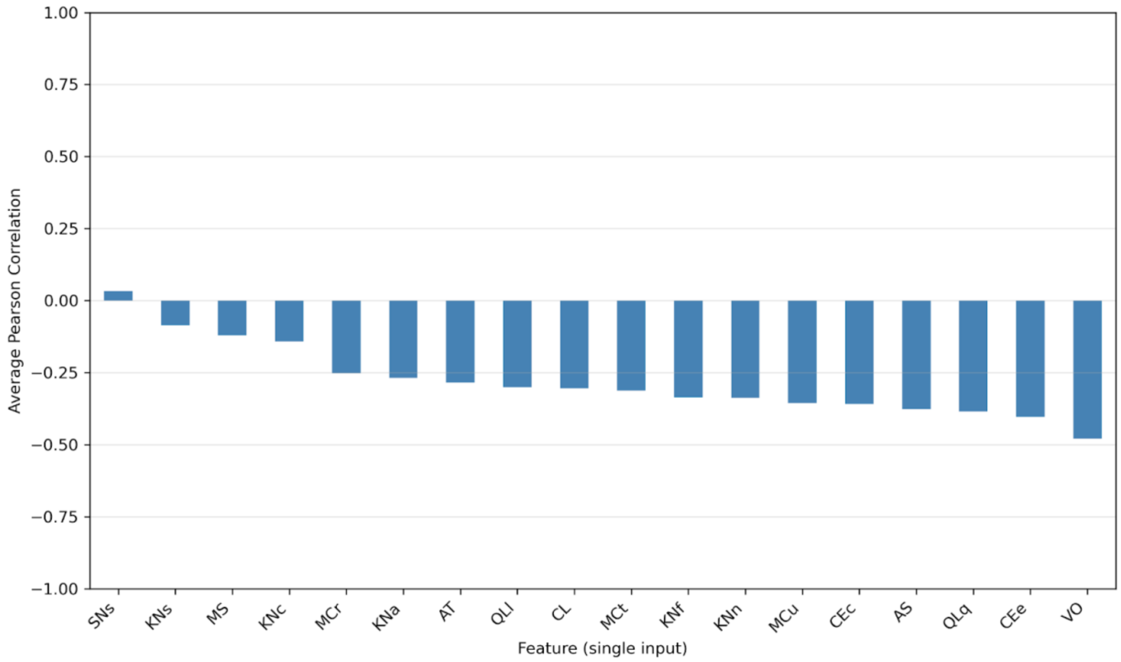

Supplementary Figure 15: Spearman Correlation of each dimension with task success when we only select those instances for which the dimension being examined is dominant.

0.86) and an average r_{WG} of 0.74 for LLaMA-3.1-8b-instruct, representing high inter-rater agreement for DeepSeek-V3 and moderately high agreement for LLaMA-3.1-8b-instruct. Similarly high predictive power is achieved via the annotations of both DeepSeek-V3 (Supplementary Table 12) and moderately worse predictive power is achieved via LLaMA-3.1-8b-instruct (Supplementary Table 13), in comparison with using GPT-4o's annotations. The moderate reduction in predictive power (but still fairly high) from LLaMA-3.1-8b-instruct is expected, as reflected by its worse inter-rater agreement with Delphi consensus and it being two order of magnitude smaller in #parameters than the other two LLM annotators. Overall, these findings indicate the model-agnosticity of our annotation pipeline, with better results as the LLMs become more powerful.

Supplementary Table 11: Agreement of ratings (r_{WG}) between Delphi consensus and DeepSeek-V3 and LLaMA-3.1-8b-instruct. Comparable to GPT-4o results (Table 9).

	AS	AT	CEc	CEe	CL	KNa	KNc	KNf	KNn	KNs	MCr	MCt	MCu	MS	QLl	QLq	SNs	VO	**Average**
**Delphi & DeepSeek-V3**	0.80	0.77	0.76	0.94	0.78	0.88	0.83	0.67	0.96	0.90	0.82	0.89	0.85	0.78	0.89	0.84	0.74	0.90	**0.83**
**Delphi & LLaMA-3.1-8b-instruct**	0.79	0.85	0.71	0.77	0.78	0.75	0.69	0.54	0.88	0.72	0.82	0.88	0.80	0.69	0.91	0.78	0.64	0.87	**0.74**

1.9. Predictive Power of Alternative Taxonomies

We include four baseline taxonomies from Miller & Tang [21] and BLOOM [22]. These are defined in two separate rubrics in sections 3.1 and 3.2 of this Supplementary Information. In addition, we derive an additional baseline called 'Generic Difficulty' (GD), which considers all the demands collectively, and derive two versions of it, where one is simply defined by prompting the LLM (GPT-4o) annotator with '*How difficult do you think this question (task instance) is on a scale from 1 to 5?*' (mimicking the self-perceived difficulty scale by the LLM annotator) and another model-agnostic version, which is defined in Supplementary Information 3.3.

We obtain the annotations defined by these rubrics using GPT-4o again and show the predictive power of each of these baseline taxonomies (and some combinations thereof) in Supplementary Tables 14, 15, and 16. Overall, the predictive power achieved through using our ADeLe framework substantially exceeds that of all these baselines (and their combinations) by a large margin, especially compared to Miller & Tang and BLOOM, which perform much

Supplementary Table 12: Full predictability results using DeepSeek-V3 (averaged across 10 seeds) with a random forest classifier. Each cell lists *AUROC, ECE*. The most predictable model is still GPT-4o, even when using DeepSeek-V3 as the annotator. Predictive power is similar to using GPT-4o as the annotator (see the columns of 'Demands (RF)' in tables Ex- tended Data Figure 2, Extended Data Figure 3 and Extended Data Figure 4).

Subject LLM	LLM Accuracy↑	ID		Task OOD		Benchmark OOD	
		AUROC↑	ECE↓	AUROC↑	ECE↓	AUROC↑	ECE↓
Babbage-002	0.102	0.784	0.004	0.746	0.008	0.649	0.041
Davinci-002	0.157	0.771	0.004	0.743	0.011	0.706	0.016
GPT-3.5-Turbo	0.414	0.814	0.008	0.801	0.017	0.777	0.050
GPT-4o	0.713	0.876	0.016	0.849	0.020	0.802	0.032
OpenAI o1-mini	0.770	0.859	0.013	0.839	0.018	0.711	0.053
OpenAI o1	0.843	0.844	0.011	0.804	0.023	0.673	0.038
LLaMA-3.2-1B	0.216	0.779	0.006	0.727	0.026	0.684	0.047
LLaMA-3.2-3B	0.378	0.812	0.010	0.789	0.021	0.768	0.049
LLaMA-3.2-11B	0.463	0.817	0.008	0.798	0.017	0.779	0.055
LLaMA-3.2-90B	0.645	0.858	0.016	0.834	0.017	0.802	0.048
LLaMA-3.2-405B	0.683	0.864	0.014	0.841	0.017	0.810	0.051
DK-R1-Dist-Qwen-1.5B	0.353	0.783	0.011	0.758	0.018	0.704	0.048
DK-R1-Dist-Qwen-7B	0.555	0.810	0.015	0.786	0.018	0.695	0.032
DK-R1-Dist-Qwen-14B	0.698	0.826	0.013	0.807	0.016	0.713	0.035
DK-R1-Dist-Qwen-32B	0.748	0.834	0.013	0.810	0.027	0.709	0.034
**Weighted Average**	—	**0.835**	**0.012**	**0.809**	**0.019**	**0.739**	**0.042**

Supplementary Table 13: Full predictability results using LLaMA-3.1-8B-Instruct (averaged across 10 seeds) with a random forest classifier. Each cell lists *AUROC, ECE*. The most predictable model is still GPT-4o, even when using llama3 as the annotator. Predictive power is slightly worse than using GPT-4o as the annotator (see the columns of 'Demands (RF)' in Extended Data Tables 2, 3 and 4 or DeepSeek (Supplementary Table 12). Results are formatted identically to enable side-by-side comparison with the DeepSeek-V3 table.

Subject LLM	LLM Accuracy↑	ID		Task OOD		Benchmark OOD	
		AUROC↑	ECE↓	AUROC↑	ECE↓	AUROC↑	ECE↓
Babbage-002	0.102	0.769	0.005	0.727	0.011	0.669	0.020
Davinci-002	0.157	0.757	0.006	0.722	0.008	0.696	0.021
GPT-3.5-Turbo	0.414	0.777	0.015	0.747	0.029	0.741	0.043
GPT-4o	0.713	0.847	0.020	0.807	0.041	0.788	0.046
OpenAI o1-mini	0.770	0.821	0.016	0.775	0.017	0.685	0.047
OpenAI o1	0.843	0.812	0.018	0.744	0.023	0.676	0.024
LLaMA-3.2-1B	0.216	0.762	0.012	0.708	0.022	0.684	0.035
LLaMA-3.2-3B	0.378	0.780	0.014	0.757	0.015	0.748	0.033
LLaMA-3.2-11B	0.463	0.781	0.014	0.755	0.026	0.753	0.042
LLaMA-3.2-90B	0.645	0.830	0.021	0.791	0.033	0.778	0.046
LLaMA-3.2-405B	0.683	0.836	0.019	0.799	0.035	0.787	0.042
DK-R1-Dist-Qwen-1.5B	0.353	0.744	0.014	0.712	0.017	0.651	0.057
DK-R1-Dist-Qwen-7B	0.555	0.776	0.024	0.744	0.020	0.643	0.061
DK-R1-Dist-Qwen-14B	0.698	0.790	0.018	0.758	0.022	0.665	0.040
DK-R1-Dist-Qwen-32B	0.748	0.803	0.013	0.768	0.022	0.683	0.042
**Weighted Average**	—	**0.803**	**0.017**	**0.764**	**0.025**	**0.714**	**0.042**

worse than the simpler GD. It is also important to note that the Miller & Tang and BLOOM baselines offer limited explanatory power (unlike ADeLe) as they are either domain-specific or learning levels that cannot adequately capture the capability space.

Supplementary Table 14: Predictive power (AUC, ECE) using a random forest classifier (assessor) for each of the extra four baseline 'taxonomies' (and some combinations thereof) in the in-distribution scenario. The numbers here are comparable with Extended Data Table 2, where our ADeLe catalogue achieves a much higher predictive power in AUROC (0.838) than all baselines here (AUROC=0.659-0.765).

Subject LLM	LLM Accuracy↑	GD		GD with rubric		Miller & Tang		Miller & Tang + GD		BLOOM		BLOOM + GD	
		AUROC↑	ECE↓	AUROC↑	ECE↓	AUROC↑	ECE↓	AUROC↑	ECE↓	AUROC↑	ECE↓	AUROC↑	ECE↓
Babbage-002	0.102	0.626	0.001	0.585	< 0.001	0.673	0.001	0.699	0.003	0.664	0.001	0.700	0.001
Davinci-002	0.157	0.660	0.004	0.632	< 0.001	0.678	0.001	0.708	0.005	0.686	< 0.001	0.719	0.003
GPT-3.5-Turbo	0.414	0.726	0.001	0.720	< 0.001	0.632	0.004	0.751	0.006	0.674	< 0.001	0.755	0.002
GPT-4o	0.713	0.768	< 0.001	0.753	0.004	0.673	0.002	0.795	0.006	0.711	0.003	0.807	0.001
OpenAI o1-mini	0.770	0.741	< 0.001	0.714	< 0.001	0.675	0.003	0.775	0.003	0.658	< 0.001	0.789	0.004
OpenAI o1	0.843	0.706	< 0.001	0.683	0.006	0.650	0.001	0.753	0.007	0.619	0.002	0.764	0.004
LLaMA-3.2-1B	0.216	0.693	0.001	0.676	0.001	0.643	0.030	0.707	0.004	0.658	< 0.001	0.721	0.006
LLaMA-3.2-3B	0.378	0.735	0.004	0.718	< 0.001	0.656	0.001	0.749	0.004	0.691	0.002	0.762	0.006
LLaMA-3.2-11B	0.463	0.738	0.004	0.722	< 0.001	0.656	0.004	0.758	0.003	0.683	0.005	0.764	0.005
LLaMA-3.2-90B	0.645	0.751	0.001	0.734	< 0.001	0.660	0.002	0.781	0.006	0.695	< 0.001	0.784	0.004
LLaMA-3.2-405B	0.683	0.766	0.003	0.746	0.001	0.664	0.004	0.789	0.003	0.701	0.004	0.798	0.004
DK-R1-Dist-Qwen-1.5B	0.353	0.678	0.001	0.638	< 0.001	0.645	0.001	0.709	0.005	0.619	0.003	0.710	0.005
DK-R1-Dist-Qwen-7B	0.555	0.695	< 0.001	0.658	< 0.001	0.660	0.003	0.729	0.006	0.610	0.002	0.727	0.004
DK-R1-Dist-Qwen-14B	0.698	0.726	0.004	0.692	< 0.001	0.663	0.002	0.744	0.005	0.624	< 0.001	0.749	0.006
DK-R1-Dist-Qwen-32B	0.748	0.733	< 0.001	0.697	< 0.001	0.658	0.004	0.751	0.003	0.625	< 0.001	0.763	0.009
Weighted Average	—	0.729	0.001	0.704	0.001	0.659	0.003	0.757	0.005	0.659	0.002	0.765	0.004

Supplementary Table 15: Predictive power (AUC, ECE) using a random forest classifier (assessor) for each of the extra four baseline 'taxonomies' (and some combinations thereof) in the OOD benchmark scenario. The numbers here are comparable with Extended Data Table 3, where our ADeLe catalogue achieves a much higher predictive power (AUROC=0.811, ECE=0.024) than all baselines here (AUROC=0.594-0.748, ECE=0.036-0.052).

Subject LLM	GD		GD with rubric		Miller & Tang		Miller & Tang + GD		BLOOM		BLOOM + GD	
	AUROC↑	ECE↓	AUROC↑	ECE↓	AUROC↑	ECE↓	AUROC↑	ECE↓	AUROC↑	ECE↓	AUROC↑	ECE↓
Babbage-002	0.553	0.010	0.477	0.027	0.623	0.012	0.652	0.008	0.627	0.016	0.662	0.014
Davinci-002	0.638	0.028	0.591	0.026	0.648	0.023	0.686	0.024	0.665	0.024	0.698	0.018
GPT-3.5-Turbo	0.699	0.001	0.682	0.015	0.593	0.034	0.728	0.064	0.652	0.049	0.744	0.019
GPT-4o	0.736	0.086	0.708	0.049	0.587	0.045	0.748	0.077	0.678	0.068	0.787	0.056
OpenAI o1-mini	0.719	0.036	0.685	0.037	0.581	0.048	0.741	0.056	0.599	0.067	0.769	0.045
OpenAI o1	0.680	0.028	0.635	0.060	0.547	0.047	0.705	0.036	0.560	0.052	0.739	0.038
LLaMA-3.2-1B	0.672	0.001	0.642	0.020	0.604	0.033	0.677	0.018	0.637	0.004	0.704	0.017
LLaMA-3.2-3B	0.711	0.042	0.683	0.005	0.615	0.044	0.726	0.045	0.670	0.033	0.752	0.020
LLaMA-3.2-11B	0.710	0.087	0.683	0.046	0.614	0.053	0.732	0.053	0.662	0.072	0.753	0.040
LLaMA-3.2-90B	0.719	0.058	0.690	0.019	0.590	0.051	0.743	0.084	0.672	0.071	0.765	0.059
LLaMA-3.2-405B	0.734	0.057	0.702	0.097	0.593	0.077	0.752	0.046	0.674	0.060	0.780	0.041
DK-R1-Dist-Qwen-1.5B	0.668	0.024	0.617	0.004	0.613	0.038	0.688	0.017	0.597	0.016	0.703	0.016
DK-R1-Dist-Qwen-7B	0.677	0.054	0.626	0.043	0.615	0.054	0.703	0.031	0.562	0.047	0.713	0.013
DK-R1-Dist-Qwen-14B	0.708	0.034	0.666	0.038	0.607	0.057	0.717	0.034	0.562	0.018	0.737	0.036
DK-R1-Dist-Qwen-32B	0.715	0.023	0.672	0.027	0.595	0.055	0.718	0.055	0.562	0.078	0.747	0.037
Weighted Average	0.704	0.043	0.667	0.039	0.594	0.050	0.723	0.049	0.618	0.052	0.748	0.036

1.10. Qualitative Analysis of Demand Annotations

We have shown the results and agreements with humans are agnostic to the choice of LLM used as annotator (provided the LLM reaches a minimum quality standard); we only saw minor variations when we changed from GPT-4o to DeepSeek-V3 (section 1.8). We also found high agreement of annotations between humans and LLMs. While humans are not the ground truth in the annotation, we can explore whether those (rare) cases where there is disagreement between the humans (using the consensus through Delphi method) and the LLM, to see if this has some influence on the (predictability of the) errors of the model. This question is especially recurrent when the same LLM is used as annotator and subject. In order to explore this, we will perform some quantitative analysis (in the form of correlations) as well as some binning and qualitative analysis.

We first analyse whether annotation 'uncertainty' (discrepancies with humans) is correlated with failures of the LLM when used as evaluated subject. Supplementary Table 17 reports the Spearman correlations between Delphi–GPT-4o annotation discrepancies and GPT-4o question failures; the coefficients are generally small, indicating little to no association.

Supplementary Figure 16 delves deeper by showing 18 confusion matrices in terms of the agreement between Delphi and LLM for all scales, with each cell showing two numbers: (1) the count of correctly solved instances and (2) the count of failed instances by GPT-4o. These matrices can show whether the variance comes mostly because of an annotation problem or the innate uncertainty of the level (if model has a capability close to the annotated

Supplementary Table 16: Predictive power (AUC, ECE) using a random forest classifier (assessor) for each of the extra four baseline 'taxonomies' (and some combinations thereof) in the OOD task scenario. The numbers here are comparable with Extended Data Table 4, where our ADeLe catalogue achieves a much higher predictive power (AUROC=0.747, ECE=0.038) than all baselines here (AUROC=0.558-0.711, ECE=0.061-0.090).

Subject LLM	GD		GD with rubric		Miller & Tang		Miller & Tang + GD		BLOOM		BLOOM + GD	
	AUROC↑	ECE↓	AUROC↑	ECE↓	AUROC↑	ECE↓	AUROC↑	ECE↓	AUROC↑	ECE↓	AUROC↑	ECE↓
Babbage-002	0.550	0.024	0.470	0.028	0.607	0.009	0.616	0.021	0.618	0.033	0.634	0.030
Davinci-002	0.639	0.039	0.599	0.025	0.646	0.026	0.678	0.033	0.666	0.027	0.689	0.035
GPT-3.5-Turbo	0.699	0.063	0.691	0.066	0.589	0.104	0.725	0.062	0.647	0.066	0.714	0.050
GPT-4o	0.733	0.144	0.711	0.107	0.536	0.111	0.747	0.093	0.659	0.086	0.747	0.073
OpenAI o1-mini	0.713	0.089	0.666	0.078	0.531	0.077	0.706	0.061	0.568	0.101	0.730	0.071
OpenAI o1	0.672	0.058	0.610	0.079	0.494	0.086	0.678	0.057	0.502	0.072	0.683	0.052
LLaMA-3.2-1B	0.673	0.020	0.633	0.043	0.599	0.066	0.668	0.024	0.611	0.034	0.683	0.034
LLaMA-3.2-3B	0.708	0.112	0.684	0.059	0.605	0.102	0.725	0.075	0.660	0.077	0.727	0.060
LLaMA-3.2-11B	0.712	0.113	0.693	0.087	0.600	0.110	0.737	0.038	0.656	0.118	0.721	0.056
LLaMA-3.2-90B	0.716	0.145	0.692	0.105	0.565	0.085	0.732	0.071	0.641	0.102	0.723	0.109
LLaMA-3.2-405B	0.731	0.089	0.705	0.133	0.534	0.136	0.749	0.079	0.644	0.072	0.743	0.058
DK-R1-Dist-Qwen-1.5B	0.658	0.052	0.609	0.045	0.596	0.058	0.675	0.025	0.577	0.078	0.679	0.043
DK-R1-Dist-Qwen-7B	0.667	0.080	0.616	0.093	0.582	0.084	0.685	0.072	0.499	0.129	0.668	0.079
DK-R1-Dist-Qwen-14B	0.703	0.098	0.650	0.066	0.560	0.107	0.703	0.068	0.493	0.142	0.670	0.064
DK-R1-Dist-Qwen-32B	0.710	0.076	0.659	0.070	0.555	0.053	0.712	0.044	0.497	0.132	0.695	0.081
Weighted Average	0.699	0.090	0.660	0.082	0.558	0.089	0.711	0.061	0.582	0.095	0.706	0.066

Supplementary Table 17: Spearman correlation between GPT-4o's annotation discrepancy (w.r.t. humans) and its failure on the question. Values near zero indicate weak or no monotonic association, suggesting the two are mostly uncorrelated.

	AS	AT	CEc	CEe	CL	KNa	KNc	KNf	KNn	KNs	MCr	MCt	MCu	MS	QLl	QLq	SNs	VO	**Average**
**Spearman** ρ	−0.35	0.02	−0.10	−0.16	0.06	0.38	−0.05	−0.26	0.27	0.29	−0.10	0.06	−0.19	−0.34	−0.05	−0.10	−0.29	−0.25	**-0.06**

demand). For instance, if we observe 5/5 in the (3,3) cell, then the maximum uncertainty (the variance) comes because they are examples of medium difficulty (level 3) but are equally identified as level 3 by humans and GPT-4 alike. The variance (low predictability) comes because the model's capability and the demand are close, not because of annotation variability. However, if we obtain 5/5 in the (2,4) cell, then we do not know if the variance comes because the model's capability and the demand are close, or possibly because the annotator is noisy. This a different kind of variance. As most of our cells are around the diagonal, we can conclude that the variance that we find is of the first kind, inherent because of the uncertainty of the subject, not the annotator.

To have even more insight in how annotations and subject errors relate, Supplementary Table 18 shows qualitative examples across eight cases depending on whether humans (Delphi) and GPT-4o agree or disagree, whether the demands are of low or high levels and whether the subject model (GPT-4o) succeeds or fails; we show up to 3 examples per case:

- **S1**: Delphi and GPT-4o both assign a very high level (=5); model succeeds.
- **F1**: Delphi and GPT-4o both assign a very high level (=5); model fails.
- **S2**: Delphi and GPT-4o both assign low or moderate levels (≤ 3); model succeeds.
- **F2**: Delphi and GPT-4o both assign low or moderate levels (≤ 3); model fails.
- **S3**: Delphi level lower than GPT-4o by ≥ 2 levels ('Delphi low vs. GPT-4o high'); model succeeds.
- **F3**: Delphi level lower than GPT-4o by ≥ 2 levels ('Delphi low vs. GPT-4o high'); model fails.
- **S4**: Delphi level higher than GPT-4o by ≥ 2 levels ('Delphi high vs. GPT-4o low'); model succeeds.
- **F4**: Delphi level higher than GPT-4o by ≥ 2 levels ('Delphi high vs. GPT-4o low'); model fails.

We see that S1 is a strange situation, with the LLM agreeing with humans as annotator for a very difficult item, but then succeeding at that item. This either shows both annotators are wrong or the instance is affected by some uncertainty not captured by our dimensions. F1 is a natural and expected case, the LLM consistently predicts the instance is hard, and fails on it. S2 is even more natural and expected, the LLM consistently predict it is an easy one, and succeeds at it. F2 is more surprising though, but this may be affected by other dimensions being not dominating but close or above the capability level of the model. Finally the examples that we have for S3 talk better about the LLM as annotator than the human, as the LLM annotates them as easier, and succeeds at them. For F3, it is the opposite, humans are annotating this higher and the LLM is failing, so these are genuine cases of wrong annotation

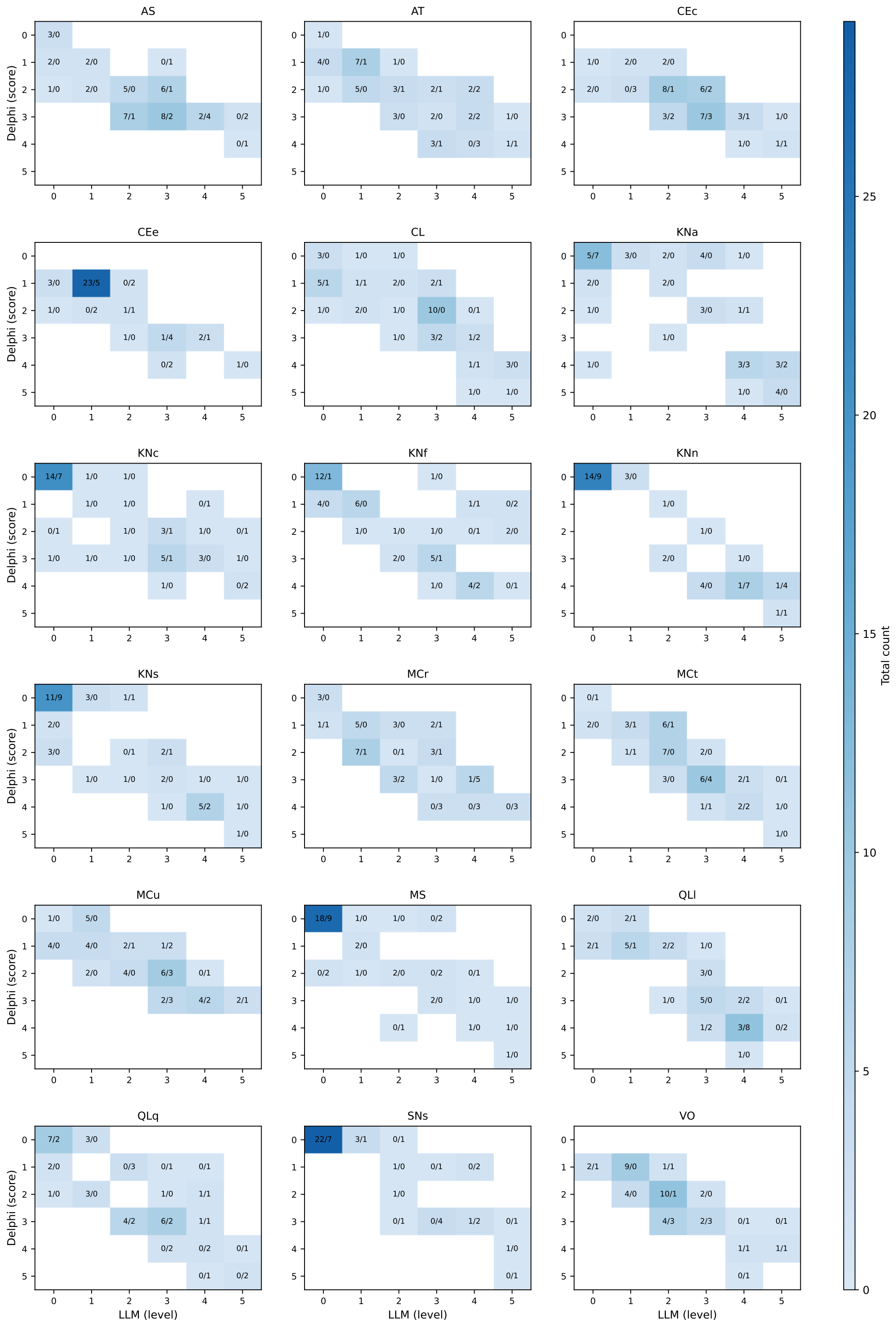

Supplementary Figure 16: Confusion matrices delineating the agreement between Delphi and LLM for all scales, with each cell's count disaggregated into two numbers: (1) the count of correctly solved instances and (2) the count of failed instances by GPT-4o. As expected, we see higher variance in the top-left corner or centre of the matrices (more balance in correct vs incorrect) because this is around the level of performance of GPT-4o, but this is affected by many examples having medium levels for several dimensions.

from the LLM. Noteworthy, we did not find any instances from S4 and F4 (two problematic scenarios where Delphi and GPT-4o annotators disagree) across all the 900 instances annotated by the Delphi group (see Methods). This was expected given the high annotation quality.

Supplementary Table 18: Qualitative samples across eight scenarios describing the cases where humans (Delphi) and GPT-4o agree or disagree depending on whether the demands are of low or high levels and whether the subject model (GPT-4o) succeeds or fails; we show up to 3 examples per case. The description of the cases can be found in the text. The column 'Dom. Dim' denotes the dimension with highest value for the instance. The column 'Delphi Level' denotes the Delphi-annotated level 'GPT-4o Level' denotes the GPT-4o–annotated level for the dominant dimension of a given question; Δ = level(GPT-4o) − level(Delphi). Remarkably, we did not find any instances from S4 and F4 (two problematic scenarios where Delphi and GPT-4o annotators disagree) across all the 900 instances annotated by the Delphi group (see Methods).

Case	Dom. Dim.	Delphi Level	GPT-4o Level	Question
S1	KNa	5	5	**Question**: A 68-year-old female has a history of breast cancer that has recurred twice in the past 5 years. She has undergone both radiation and chemotherapy for metastatic carcinoma to the bone. She presents to the office today to ask for help in dealing with midthoracic back pain to enable her to use less analgesic agents. The most appropriate osteopathic manipulative technique to treat this patient's spine is\n Choices:\n A. direct soft tissue technique\n B. high velocity, low amplitude\n C. high velocity, high amplitude\n D. indirect myofascial release\n E. low velocity, low amplitude\n F. isotonic exercise therapy\n G. mid velocity, mid amplitude\n H. static stretching technique\n I. direct myofascial release\n J. isotonic resistance therapy
S1	KNs	5	5	**Question**: A university student, a citizen of State A, believes that her university violated federal law when it eliminated funding for the women's varsity volleyball team. She has sued the university, a nonprofit corporation located and incorporated in State B, and the university's athletic director, a citizen of State B, in federal court in State B. What is the best method of serving the defendants?\n Choices:\n A. Service by publication in a newspaper of general circulation in State B.\n B. Service as required by State B's rules of civil procedure.\n C. Service by emailing copies of the summons and complaint to the athletic director and the university president.\n D. Service by faxing copies of the summons and complaint to the athletic director and the university president.\n E. Service by the student herself on the athletic director and the university president.\n F. Service by a process server's leaving copies of the summons and complaint with copies of the summons and complaint with secretaries in the respective offices of the athletic director and the university president.\n G. Service by the student's attorney on the athletic director and the university president.\n H. Service by sending copies of the summons and complaint through the university's internal mail system.\n I. Service by leaving copies of the summons and complaint at the university's main entrance.
S1	MS	5	5	The media has recently been filled with gossip news about a celebrity, which puts the celebrity in an awkward situation? if she does not come forward to clarify and refute, those rumors will be believed by the public; if she comes forward to clarify and refute, This will attract more people's attention and make those gossip news spread faster and wider.This may be the price that celebrities have to pay.\n **Question**: If the statement in the stem is true, which of the following must be true?\n Choices:\n A. The celebrity can't actually stop the gossip news from damaging her personal reputation.\n B. The reputation of a celebrity will not be affected by gossip news in the media.\n C. When faced with gossip news, the best strategy the celebrity can take is to clarify the truth.\n D. Some friends of the celebrity came forward to praise her, which would have a counter-effect.
F1	SNs	5	5	**Question**: (Self-Isogonal Cubics) Let ABC be a triangle with $AB = 2, AC = 3, BC = 4$. The isogonal conjugate of a point P, denoted P^*, is the point obtained by intersecting the reflection of lines PA, PB, PC across the angle bisectors of $\angle A$, $\angle B$, and $\angle C$, respectively. Given a point Q, let $\mathfrak{R}(Q)$ denote the unique cubic plane curve which passes through all points P such that line PP^* contains Q. Consider: (a) the M'Cay cubic $\mathfrak{R}(O)$, where O is the circumcenter of $\triangle ABC$, (b) the Thomson cubic $\mathfrak{R}(G)$, where G is the centroid of $\triangle ABC$, (c) the Napoleon-Feurerbach cubic $\mathfrak{R}(N)$, where N is the nine-point center of $\triangle ABC$, (d) the Darboux cubic $\mathfrak{R}(L)$, where L is the de Longchamps point (the reflection of the orthocenter across point O), (e) the Neuberg cubic $\mathfrak{R}(X_{30})$, where X_{30} is the point at infinity along line OG, (f) the nine-point circle of $\triangle ABC$, (g) the incircle of $\triangle ABC$, and (h) the circumcircle of $\triangle ABC$. Estimate N, the number of points lying on at least two of these eight curves.
F1	QLq	5	5	**Question**: Determine if there exists a (three-variable) polynomial $P(x, y, z)$ with integer coefficients satisfying the following property: a positive integer n is *not* a perfect square if and only if there is a triple (x, y, z) of positive integers such that $P(x, y, z) = n$.
F1	QLq	5	5	**Question**: We say that a subset of $\mathbb{R}^n$ is k-almost contained by a hyperplane if there are fewer than k points in that set which do not belong to the hyperplane. We call a finite set of points k-generic if there is no hyperplane that k-almost contains the set. For each pair of positive integers k and n, find the minimal number $d(k, n)$ such that every finite k-generic set in $\mathbb{R}^n$ contains a k-generic subset with at most $d(k, n)$ elements.
S2	VO	2	2	**Question**: $b = 2.35 + 0.25x,\ c = 1.75 + 0.40x$\n In the equations above, b and c represent the price per pound, in dollars, of beef and chicken, respectively, x weeks after July 1 during last summer. What was the price per pound of beef when it was equal to the price per pound of chicken? \n Choices:\n A. \$2.60\n B. \$2.85\n C. \$2.95\n D. \$3.35
S2	KNf	2	2	**Question**: Miranda enlarged a picture proportionally. Her original picture is 4 cm wide and 6 cm long. If the new, larger picture is 10 cm wide, what is its length?\n Choices:\n A. 22 cm\n B. 15 cm\n C. 16 cm\n D. 10 cm\n E. 12 cm\n F. 24 cm\n G. 8 cm\n H. 18 cm\n I. 20 cm\n J. 30 cm
S2	MCt	2	2	**Question**: A rectangle has width x and length y. The rectangle is cut along the horizontal and vertical dotted lines to produce four smaller rectangles. The sum of the perimeters of these four rectangles is 24. What is the value of $x + y$?
F2	KNc	3	3	**Context**: Siim Kallas was an Estonian politician who served as European Commissioner for Transport between 2010 and 2014. Before that, he was European Commissioner for Administrative Affairs, Audit and Anti-Fraud between 2004 and 2009. Kallas has also served as Prime Minister of Estonia, Estonian Minister of Finance, and Estonian Minister of Foreign Affairs.\n **Question**: Which position did Siim Kallas hold after Minister of Foreign Affairs?\n Answer: European Commissioner for Administrative Affairs, Audit and Anti-Fraud.

Continued on next page

Case	Dom. Dim.	Delphi Level	GPT-4o Level	Question
F2	QLq	3	3	**Question**: Let ABC be a triangle with $AB = 9, BC = 10$, and $CA = 17$. Let B' be the reflection of the point B over the line CA. Let G be the centroid of triangle ABC, and let G' be the centroid of triangle $AB'C$. Determine the length of segment GG'.
S3	KNa	0	4	**Question**: What are the results of fertilization?\n Choices:\n A. Creation of multiple cells, apoptosis, gene silencing, and evolutionary adaptation\n B. Fusion of diploid cells, onset of embryonic development, gender differentiation, and genetic stabilization\n C. Formation of a diploid cell, mitosis, meiosis, and genetic diversity\n D. Replication of somatic cells, induction of pluripotency, chromosome pairing, and alteration of genetic code\n E. Fusion of haploid sex cells, initiation of zygote cleavage, sex determination, and species variation\n F. Cell division, gene expression, DNA replication, and genetic variation\n G. Synthesis of new proteins, activation of growth factors, chromosomal crossing-over, and phenotype establishment\n H. Formation of a haploid cell, cytokinesis, tissue differentiation, and mutation introduction\n I. Cell differentiation, organogenesis, morphogenesis, and genetic recombination\n J. Combination of genetic material, activation of embryonic genes, cellular specialization, and creation of genetic clones
S3	AT	3	5	**Context**: Charlotte Lotte Reiniger (2 June 1899 – 19 June 1981) was a German film director and the foremost pioneer of silhouette animation. She is best known for *The Adventures of Prince Achmed* (1926) and *Papageno* (1935). Reiniger made more than 40 films using her invention of the first multiplane camera and developed a distinct animation style focused on gestures, metamorphosis, and expressionism.\n **Question**: What was the residence of Lotte Reiniger from 1980 to 1981?
S3	KNs	3	5	**Question**: Congress has recently enacted a statute requiring all males between the ages of 18 and 30 to take a physical examination each year. The results of the exam are sent to a government data information center for the purpose of keeping information about men who may be drafted into the military. A 25-year-old law school graduate has herpes. He has recently sent resumes to many governmental agencies. Fearful that the information about his herpes condition will become available, he seeks a declaratory judgment that would forbid the government from requiring him to take a physical examination. Which of the following is the best constitutional basis in support of the federal law?\n Choices:\n A. The full faith and credit clause.\n B. The dormant commerce clause.\n C. The necessary and proper clause.\n D. The equal protection clause.\n E. The commerce clause.\n F. The supremacy clause.\n G. To raise and support an army and navy.\n H. The privileges and immunities clause.\n I. To provide for the general welfare.\n J. The due process clause.
F3	MCu	2	4	**Question**: Consider a ball rolling in a horizontal circular path on the inside surface of a cone. The normal force on the ball\n Choices:\n A. may be greater or less than mg\n B. is less than mg always\n C. is greater than mg always\n D. is dependent on the radius of the circular path\n E. is equal to the gravitational force on the ball\n F. is less than the weight of the ball\n G. is mg\n H. is independent of the mass of the ball\n I. is independent of the surface material of the cone\n J. is dependent on the velocity of the ball
F3	KNa	2	4	**Question**: The following data show how the standard molar constant-pressure heat capacity of sulfur dioxide varies with temperature. By how much does the standard molar enthalpy of $SO_2(g)$ increase when the temperature is raised from 298.15 K to 1500 K?
F3	VO	3	4	**Question**: For each positive integer n, denote by $\omega(n)$ the number of distinct prime divisors of n (for example, $\omega(1) = 0$ and $\omega(12) = 2$). Find all polynomials $P(x)$ with integer coefficients such that whenever n is a positive integer satisfying $\omega(n) > 2023^{2023}$, then $P(n)$ is also a positive integer with\n $\omega(n) \geq \omega(P(n))$.\n Greece (Minos Margaritis – Iasonas Prodromidis)

1.11. Annotation Perturbation Analysis

Supplementary Table 19 shows the agreement rates between the original demand annotations of GPT-4o and the annotations of GPT-4o after reshuffling the choice order for a randomly extracted set of 200 multiple choice questions in ADeLe. Overall, we see a near perfect agreement, a r_{WG} index of 0.916 after averaging the scores in all 18 scales.

Supplementary Table 19: r_{WG} index delineating the agreement rates, across scales, between the original demand annotations of GPT-4o and the annotations of GPT-4o after reshuffling the choice order for a randomly extracted set of 200 multiple choice questions in ADeLe.

	AS	AT	CEc	CEe	CL	KNa	KNc	KNf	KNn	KNs	MCr	MCt	MCu	MS	QLl	QLq	SNs	VO	**Average**
r_{WG}	0.93	0.93	0.95	0.90	0.91	0.92	0.74	0.95	0.93	0.92	0.92	0.95	0.90	0.85	0.95	0.92	0.94	0.97	**0.92**

1.12. *From 'can LLMs reason?' to 'how much do LLMs reason?'*

Reasoning has become ubiquitous when making capability claims about LLMs: model cards, post-training papers, and several surveys now foreground "reasoning" as a primary objective and evidence of progress. Gains on a handful of datasets are routinely interpreted as "LLMs can reason" [160] while low scores on newer, harder batteries are read as evidence of the opposite [161]. This subsection replaces that subjective binary perspective with an objective quantitative question: how much do current systems reason, at which levels, and under which non-reasoning demands? Our measurement is not based on mixing incommensurate results on several benchmarks—that is the reason for inconsistencies—but on the use of the same scales on the reasoning dimensions. That allows us to resolve contradictory claims in the literature of AI evaluation.

1.12.1. *Toward Reconciling Common Myths on LLM "Reasoning"*

As we have previously shown, our general scales do not saturate as average performance does; they therefore support construct-valid analysis of "reasoning" benchmarks, placing their reasoning demands (and other non-reasoning ones) on the same scale. To analyse the claims on reasoning, we annotate the demand profiles of many so-called "reasoning" benchmarks, putting emphasis on reasoning dimensions while tracking non-reasoning demands and extraneous factors. These benchmark profiles explain conflicting results, e.g., clarifying when a high aggregate performance score genuinely reflects reasoning capabilities versus when a low score is caused by high demands for other non-reasoning capabilities. In this sense, we revisit common faulty inferences or myths in relation to measuring and interpreting reasoning:

(i) Benchmarks measure reasoning if their creators say they measure reasoning;
(ii) High accuracy on a dataset labelled as "reasoning" implies strong reasoning;
(iii) Low scores on a dataset labelled as "reasoning" show a lack of reasoning;
(iv) Longer chains-of-thought or "thinking time" demonstrate reasoning;
(v) Raising difficulty automatically raises reasoning demand and no other non-reasoning demands;
(vi) Performance improvements on mixed-demand benchmarks generalise to other reasoning problems.

Figure 1 in the introduction already illustrates how applying these inferences for DK-R1-Dist-Qwen-14B leads to counter-intuitive results: the same model shows near-ceiling average performance on GSM8K, middling on OlymMATH Easy, worse on GPQA, and low on OlymMATH Hard. If we only look at accuracies separately, one could boldly argue both that this model "can" and "cannot" reason at the same time. However, looking at the DeLeAn profiles solves this conflict. We give a more detailed analysis below.

Here we operationalise this analysis for twelve widely cited "reasoning" benchmarks (see Supplementary Figure 17) highlighting recurrent construct-validity pitfalls in the literature. First, sensitivity: many datasets labelled "reasoning" rarely populate upper levels of reasoning dimensions (so they cannot detect gains or deficits of systems there) and always focus on a narrow range of levels of reasoning demands. Second, specificity: authors of these "reasoning" benchmarks often raise "difficulty" via volume, atypicality or non-reasoning demands, rarely via genuine reasoning difficulty. Using DeLeAn demand profiles, we quantify and separate these effects, and assess each considered benchmark (in the order of Supplementary Figure 17, where the first five yield negative claims and the last seven positive claims): what it claims to measure, whether it truly does so (per DeLeAn), and how to interpret its high/low accuracy. We end by synthesising a set of "reasoning myths" refuted via the evidence we have discovered.

1.12.2. *Negative Claims*

We start with some benchmarks that claim that LLMs cannot reason. All of them were published in 2025.

***USAMO 2025** [161] – Released in September 2025: Negative Claim.* The dataset design intention is highly specific and reasonably narrow: measuring "*rigorous proof-based reasoning*" and "*full solution reasoning*", evaluated via free-form solutions, and human partial credit that rewards justified chains of thought. The authors argue that USAMO requires detailed proof and explanation. They report that all tested models performed poorly (with only one model above 20%), concluding that current systems are inadequate for rigorous proof-based reasoning.

The benchmark consists of six uncontaminated USAMO 2025 problems; solutions were anonymised and double graded by four expert judges using USAMO-style rubrics with partial credit derived from verified AoPS (Art of Problem Solving) solutions. Open-ended proofs (no funnelling) and partial credit reduce the chance that low scores

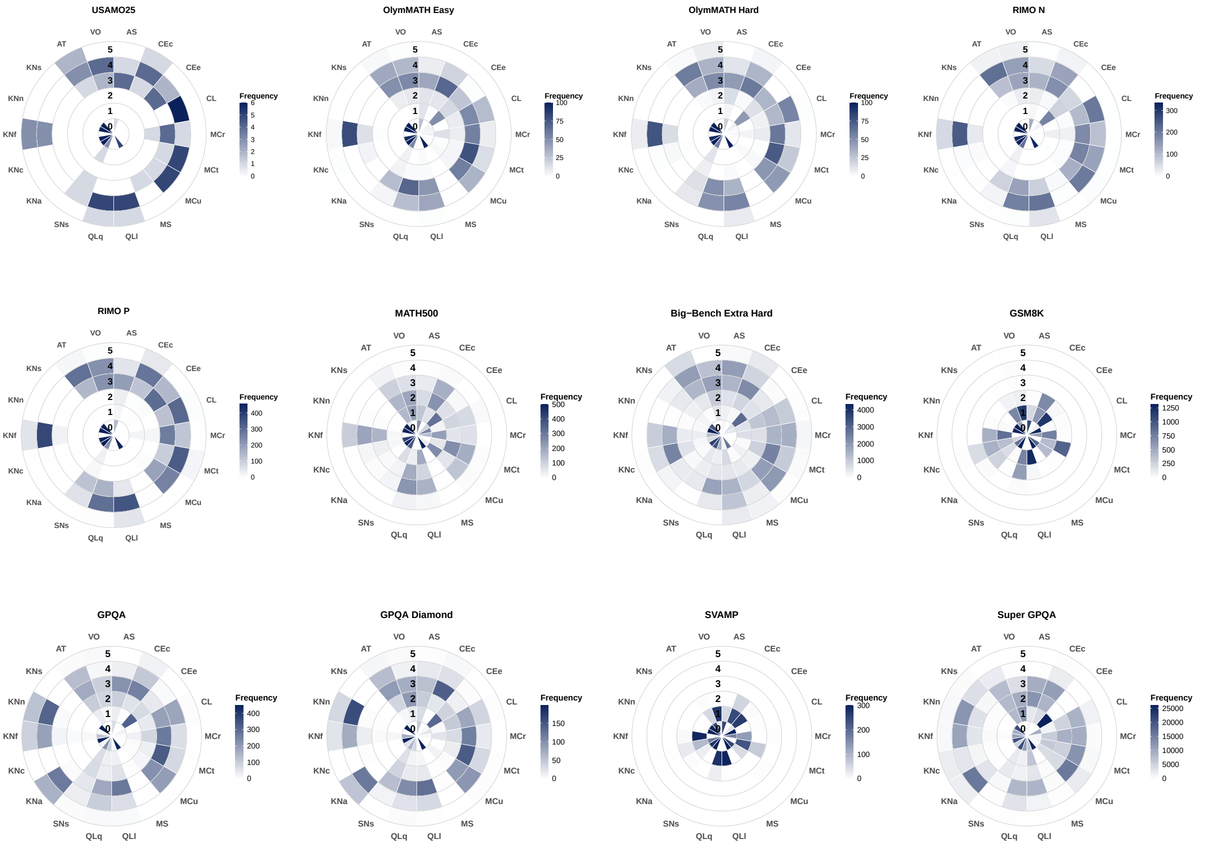

Supplementary Figure 17: Distribution of level frequencies for the 18 demands (i.e., demand profiles) of 12 "reasoning" benchmarks analysed in Supplementary Information 1.12. Some of them, with low demands on the reasoning capabilities (`CL`, `QLq` and `QLl`), have been used to claim that LLMs can reason, while others, with high demands on non-reasoning capabilities such as `KNn`, `KNa` or `MS`, have been used to claim that LLMs cannot reason. Namely, the first five have been associated with negative claims and the last seven with positive claims. Other non-reasoning dimensions, especially `VO`, have been used to make instances more complicated without really increasing the reasoning demands.

are an artefact of answer formatting. The small number of problems is countered by (i) high reliability of USAMO items as proof tasks and (ii) fine-grained, human partial-credit scoring.

This benchmark's demand profile (Supplementary Figure 17) shows that reasoning demands are concentrated in the top levels (`QLl` ~4–5, `QLq` ~4-5, `CL` ~4), with metacognitive demands (`MCr`, `MCt`, `MCu`) generally around level 4. The demand for `KNf` (knowledge of formal sciences) is also high (~4–5). Volume (`VO`) is high (sustained proof search). `AT` is high (novel, non-templated instances), which reduces contamination and funnelling at the cost of difficulty. Indeed, judge comments most often cite logical gaps in the solutions, unjustified assumptions and lack of creativity/strategy choice, rather than routine algebraic mistakes; this aligns with the high reasoning and metacognitive demands from the profile.

Low performance scores (and the negative claim about 'LLM cannot reason') reported by the original authors can be explained by the high-level demands in reasoning-related dimensions. Most items inhabit the top levels in the reasoning-related dimensions; while non-reasoning dimensions such as `VO`, `AT` and `KNf` are high, moderate values for `CEe` are also present. Those properties are intrinsic to proof-writing and are controlled by the grading scheme used, although there may be an uncontrolled effect for `CEc`, which could explain some failures as being caused by poor understanding of the question.

OlymMATH *[162] – Released in March 2025: Negative Claim.* With respect to the construct the authors intend to measure and the outcomes they observed with this battery, we find claims like: "*OlymMATH [is] designed to rigorously test the complex reasoning capabilities of LLMs*", "*All tested models exhibit relatively poor performance, [a high level of which] demands stronger reasoning abilities and a deeper understanding of mathematical knowledge*". The OlymMATH benchmark consists of olympiad-style maths problems drawn from algebra, geometry, number theory, and combinatorics. The authors developed an EASY and a HARD split, and observed very big drops in performance when increasing difficulty.

With the `DeLeAn` profiles (Supplementary Figure 17), the separation between EASY and HARD splits is clear. EASY concentrates `QLq` and `QLl` around 3 with a meaningful share at 4; HARD shifts a larger fraction to level 4 (with a small presence at 5). `CL` rises from mainly 3–4 in EASY to predominantly 4 in HARD, and metacognitive dimensions (`MCr`/`MCt`/`MCu`) also increase moderately. `KNf` is consistently high across both splits; other non-reasoning ability dimensions generally stay low or moderate. This indicates that the HARD subset contains a denser concentration of tasks nearer the top levels of our reasoning scales, rather than being simply similar items that are more difficult in arbitrary ways. Still, extraneous demands remain non-trivial: `AT` is moderate–high (curation from non-templated sources), and `VO` is moderate–high with multi-step solutions; both increase slightly in HARD, coherently with the authors' contamination-avoidance and "exhaustive case" design, but they do not seem determinant in the shift of difficulty.

Looking at the accuracies in both splits in the original OlymMATH paper [162, Table 4] and reported on Figure 1 for DS-R1-Distill-Qwen (14B), going from 61.80% to 13.30%, we see large drops in performance between EASY and HARD subsets. Such gaps can be explained via the `DeLeAn` demand profiles; there's a clear uplift in reasoning demands—more items at level 4 in reasoning dimensions (e.g., `QL`, `CL`), with some even reaching level 5 in the HARD subset. For DS-R1-Distill-Qwen (14B), this happens around the ability level of the model, where it has the highest slope, explaining the big decline in performance. For other models evaluated in the same paper, whose capabilities are much lower or much higher, the drop in performance may even be less than 10 points. So-called 'emergent' capabilities just depend on the location of most of the examples in the dataset in the logistic capability curve of the model.

RIMO *(-N and -P splits) [163] – Released in September 2025: Negative Claim.* RIMO targets "*advanced mathematical reasoning*" and specifically "*actual Olympiad-level reasoning*" through two complementary tracks: RIMO-N and RIMO-P. In RIMO-N the problems are classical olympiad-style (geometry, number theory, algebra, combinatorics) remapped to unique numeric outputs. The intent is to preserve peak olympiad difficulty while avoiding ambiguity about partial credit or expression. In RIMO-P the same genre of problems is presented as concise proof steps that must be correct consecutively; an LLM judge (Deepseek-R1) enforces strictness, halting at the first flawed step.

The DeLeAn profile for RIMO-N (Supplementary Figure 17) shows a dominant mass of instances at level 4 with few items touching 5; most items lie at level 4 for reasoning dimensions (`QLq`, `QLl`, `CL`). Metacognitive demands are moderate-high, with most of the instances at levels 3-4. `KNf` is high as expected for the International Mathematical Olympiad (IMO). Extraneous dimensions are also substantial; volume (`VO`) is high; atypicality (`AT`) is high because of the use of curated, non-contaminated items. In the case of RIMO-P, the distribution is similar to RIMO-N in the reasoning dimensions, only with moderately more instances at level 4 and less at level 3. The increase is more notable in metacognitive dimensions such as `MCt` and `MCr`, whose distribution concentrates higher in the scale. Demand level for the comprehension ability and especially expression ability (`CEe`) are also notably increased, which is expected considering the need to write proofs for RIMO-P. Atypicality (`AT`), volume (`VO`) and other non-reasoning dimensions remain similar to the -N split.

The authors treated the performance drop in RIMO-P as purely a deficit in mathematical reasoning. Nevertheless, this overlooks the more notable increase in comprehension (`CEc`) and metacognition (`MC-`), and most especially expression (`CEe`), also with a different grading method. Looking at the `DeLeAn` profiles, we can explain the difference being caused by many other factors other than an increase in reasoning demands.

1.12.3. Positive Claims

Now we look at another group of benchmarks that were usually introduced or used to claim that LLMs *can* reason. Surprisingly, there is a much higher variability in the year of the claim, going from 2021 to 2025.

MATH500 *("most difficult" subset of MATH [164]) – Released in March 2021: Positive Claim.* The full MATH dataset is presented as a set of mathematical problem-solving tasks: analyse the problem, choose useful heuristics, and chain them together (explicitly contrasted with "plug-and-chug" problems, where a single formula has to be identified and applied). The 500-item slice (MATH500) has become ubiquitous in "reasoning" sections of model reports and post-training papers.

The `DeLeAn` profile (Supplementary Figure 17) places most items at levels 2-3 for quantitative and logical reasoning (`QL`) and metacognition (`MC`) dimensions. Knowledge in formal mathematics (`KNf`) is the most salient secondary dimension; volume is low, and atypicality is low to moderate, despite some items being located at level 4. This mix means many items rely on routine reasoning plus formal-maths knowledge and familiar heuristics, rather than sustained high-level reasoning.

Consequently, strong MATH500 performance demonstrates mid-level reasoning and `KNf` rather than advanced reasoning. Using MATH500 as a proof of "reasoning achieved" (e.g., [165, 166]) is very likely an overconfident claim that predicates on model performance only on a narrow region of reasoning scales. This clarifies two misunderstandings that recur across the literature: high accuracy on a benchmark with a "reasoning" label is insufficient as evidence of strong reasoning if the benchmark items concentrate on low or mid levels on 'reasoning', and improvements measured in a given narrow region of low-mid reasoning levels do not transfer to much more demanding reasoning problems.

Big-Bench Extra Hard (BBEH) *[149] – Released in in February 2025: Positive Claim.* The designers intended BBEH to "*push the boundaries of reasoning*," reduce funnelling and broaden coverage beyond maths or coding to multi-hop, error finding and needle-in-a-haystack tasks. Difficulty is increased deliberately with longer inputs and adversarial construction of instances.

The `DeLeAn` profile (Supplementary Figure 17) shows reasoning demands predominantly in the middle band (`QLl`/`QLq` and `CL` are more populated in levels 3 and 4. Metacognitive demands have a similar distribution, but level 4 instances are more frequent for these dimensions. Volume (`VO`) and atypicality (`AT`) rise significantly by design; several instances also lean on knowledge (often `KNf` and `KNc`).

This means BBEH certainly makes tasks harder, but mainly via longer responses, novelty and breadth rather than consistently lifting reasoning demands of items to higher levels. High accuracy on this benchmark is therefore a sign of robustness to `VO` or `AT` and moderate reasoning, not necessarily a mastery of high-level reasoning. Despite this, some prior work [167] still inappropriately uses BBEH as the benchmark to measure "complex reasoning". Our analysis thus clarifies a common misunderstanding: more "thinking time" or longer chains for correct results do not by themselves imply better reasoning unless reasoning demands are held high and constant. Some easy reasoning problems may require long reasoning chains.

GSM8K *[168] – Released October 2021: Positive Claim.* GSM8K is the most well-known grade-school maths problem set and was used to demonstrate that chain-of-thought prompting lifts performance [169]. It is still common to see GSM8K being used as a headline to argue that models can reason [170, 171].

The `DeLeAn` profile (Supplementary Figure 17) places GSM8K items at the lower end of our reasoning scales (`QLl`, `QLq` and `CL`). A similar observation extends to metacognitive dimensions. Comprehension (`CEc`) is modest; knowledge required is elementary (all instances below 3 for these dimensions); and both volume (`VO`) and atypicality (`AT`) low.

Near-ceiling results for this benchmark are very common in 2025, as this benchmark is saturated, but this does not deter researchers from employing this battery. These almost perfect scores indicate mastery of simple mapping from text to operation and basic multi-step arithmetic. They definitely do not indicate capability in mid- or high-level reasoning. This is precisely why the same model can be near-ceiling on GSM8K while performing poorly on the challenging benchmarks like the ones we described previously (Supplementary Information 1.12.2). Interpreting GSM8K as proof of general reasoning [171] conflates saturation at low levels with breadth of the capabilities (no specificity), reinforcing the misunderstanding that improvements on low-demanding instances transfer to highly demanding reasoning tasks.

GPQA (main) *and* ***GPQA-Diamond*** *[172] – Released in November 2023: Positive Claim.* GPQA's declared aim is to be "Google-proof" expert-level multiple-choice questions for scalable oversight in biology, physics and chemistry rather than a dedicated reasoning probe. Nonetheless, it is frequently misused downstream as "reasoning" evidence [173–175], even in frontier system cards like Claude-Sonnet-4.5's [176].

The DeLeAn profiles (Supplementary Figure 17) match the intent of the authors of the benchmark but not the community's use. We see knowledge dimensions dominate at the top levels for both splits (Main and Diamond), with moderate comprehension and reasoning centred around level 3, with small tails to level 4The Diamond subset has mainly non-reasoning dimensions proportionally more populated at higher levels, which makes this split harder, but not due to increased reasoning demands. Moreover, the only dimension from the "metacognition and critical thinking" category that reaches higher levels is confidence calibration (MCu); in contrast, multi-step derivation, which could be more closely linked to reasoning, stays roughly constant/ In fact, switching from GPQA to GPQA-Diamond (the harder subset), we see some decline from level 4 to level 3 for reasoning dimensions (QLq, QLl, CL).

Accordingly, high accuracy scores in the GPQA-diamond indicate higher levels of expert knowledge and better confidence calibration, rather than sustained high-level reasoning. This clarifies the recurrent misunderstanding that "harder" questions or "frontier" topics necessarily target reasoning: here, most of the performance change is knowledge-driven.

***SuperGPQA** [177] – Released in March 2025: Positive Claim.* This benchmark is framed as a long-tail, graduate-level test across hundreds of disciplines. However, it often appears in "reasoning" model cards as if strong SuperGPQA performance substantiated high-level reasoning [178, 179].

The DeLeAn profile (Supplementary Figure 17) shows a very different picture of what drives variance: the reasoning dimensions sit mostly in the middle level (QLq/QLl $\approx$3, with a modest tail to 4 for QLq and less noticeable for QLl); the metacognition dimensions (MCt/MCu/MCr) cluster around 3; comprehension is moderate; and the dominant source of difficulty is knowledge, especially KNn, KNa and KNf, which consistently reach higher levels. Volume (VO) is low to moderate (short items) and atypicality (AT) is moderate (due to curated, expert-authored items). In short, most items do not inhabit the upper levels of the reasoning scales; they inhabit mid-level QL over deep, long-tail knowledge.

Read this way, SuperGPQA is best understood as a domain-coverage challenge rather than a dedicated test of sustained high-level reasoning (though certain prior work, e.g., [180], treated it as a reasoning test). Strong accuracy results are compatible with broader knowledge coverage and better elimination of distractors. This clarifies that "harder" does not necessarily mean "requiring more reasoning" (here, hardness mainly comes from knowledge breadth).

***SVAMP** [181] – Released in March 2021: Positive Claim.* SVAMP was explicitly created to expose heuristics in elementary 'one-unknown' maths problems by perturbing question sensitivity to simple rewrites. It succeeded in showing that early systems were over-counting success on templated instances; however, SVAMP often appeared alongside GSM8K as evidence of "reasoning achieved." [182–185].

The DeLeAn profile (Supplementary Figure 17) shows most dimensions are generally populated around level 1 (reasoning and metacognitive are among them), with many instances being located at level 0 in many dimensions. This again explains the apparent timeline contradiction (the "LLMs can reason" opinion represented in 2022-2023 by influential works from Kojima et al. [170] and Wei et al. [169], versus other results in 2025 stating "LLMs cannot reason"): early high performance results were obtained with very low levels of reasoning scales; later "reasoning" batteries, as shown above, started moving demands (both reasoning and non-reasoning ones) towards higher levels. Our analysis of SVAMP using DeLeAn therefore clarifies two persistent misunderstandings: that high accuracy on an elementary reasoning dataset implies general reasoning, and that improvements on such sets imply transfer to other "reasoning" tasks.

1.12.4. Two Extra "LLM Reasoning" Papers Confounded by Volume and Chain-of-Thought Effects

Lastly, before exploring all the myths together, we analyse two widely cited papers that challenge the ability of LLMs to reason, but whose conclusions are confounded by problem size (VO) and related non-reasoning demands that interact with task difficulty. We show that, once non-reasoning demands and difficulty are accounted for, the reported successes or failures do not cleanly reflect higher-level reasoning or lack thereof. The two papers are "The illusion of thinking" [186] (whose collection of benchmarks we will call 'PUZZLE'), and OMEGA [187].

***The Illusion of Thinking** [186].* This work has been extremely popular on social media and in the news due to its authors' confident claim of "LLM cannot reason". It introduces four 'PUZZLE' families (Towers of Hanoi, Checker Jumping, Blocks World, River Crossing) whose instances are parameterised by size N (e.g., number of discs, pieces or

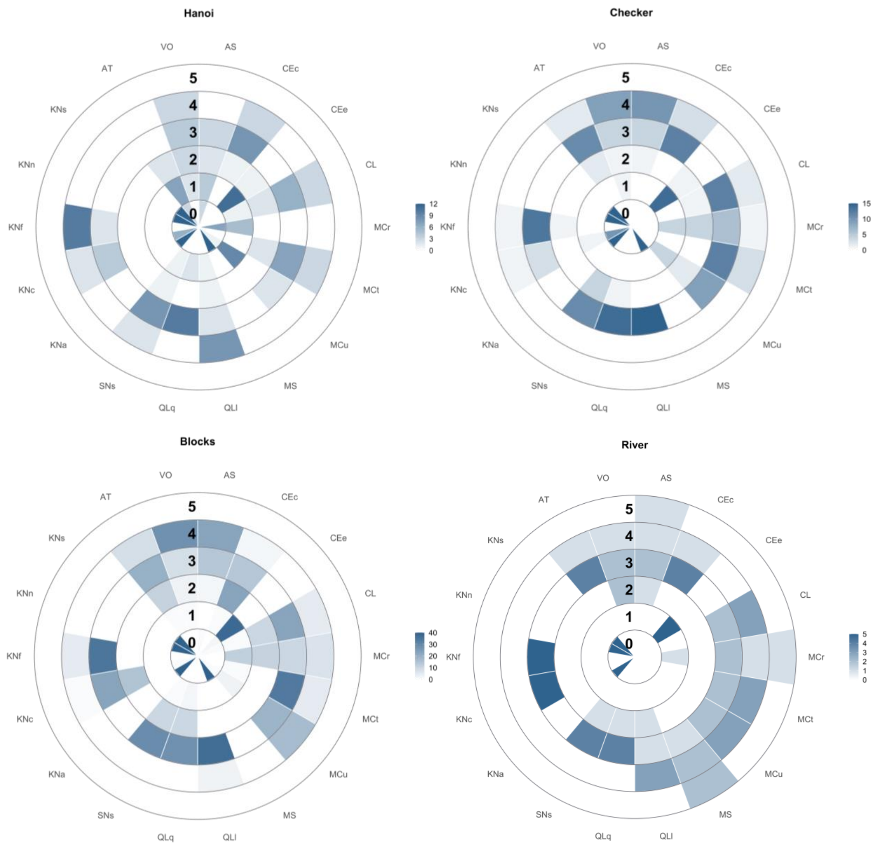

Supplementary Figure 18: Benchmark profiles for the 'PUZZLE' datasets: Towers of Hanoi, Checker Jumping, Blocks World and River Crossing from Shojaee et al. (2025) [186]. While we see reasoning (QL and CL), as well as KNf, having levels around 3 and 4, there is little sensitivity, since the range is short. Also, the specificity of the test is compromised by wide ranges in AT, VO, AS, and CEc, which are not about reasoning and could explain the difficulty of the items more than reasoning. Also, some of them require common knowledge (KNc) and spatial reasoning (SNs), but River Crossing is also loaded on mind modelling and social cognition (to understand what each character is compatible with).

people to move, etc.). Higher levels of N lead to greater difficulty. The authors of the paper compare the performance and the number of tokens used as a function of N, and they find that Language Reasoning Models (LRMs) are comparable to traditional LLMs for low values of N, while LRMs are better than LLM for slightly higher values of N, before falling down for some even higher values. Authors conclude that LRMs (and classical LLMs) cannot reason for these tasks, precisely because of this fall, which we instead explain as a natural logistic-like decrease of performance as we increase the Volume dimension (VO), which plays the role of N in our setting.

By using DeLeAn across these items, (see the Supplementary Figure 18) we see that the majority of tasks land in mid-to-high levels on non-reasoning dimensions, Volume (VO), Atypicality (AT), and Metacognition (MC), while the reasoning dimensions (QL and CL) remain around mid levels (roughly 2–4). In other words, the difficulty rise is driven more by N (i.e., Volume) than by an ascent into higher reasoning levels. They also explore the length of chain-of-thought reasoning traces, showing that their length increases up to this point of 'rupture', which is interpreted by the authors as evidence of LRMs not really 'thinking'.

This work by Shojaee et al. [186] has received many criticisms and has been interpreted in many different ways[7]). With DeLeAn we easily see that the drop of LRMs models at some difficulty levels (the rupture point) is mainly because of an increase in Volume, not in the reasoning dimensions, clarifying these differing interpretations.

[7] See this discussion of mistakes and misunderstandings: https://aievaluation.substack.com/p/2025-june-ai-evaluation-digest

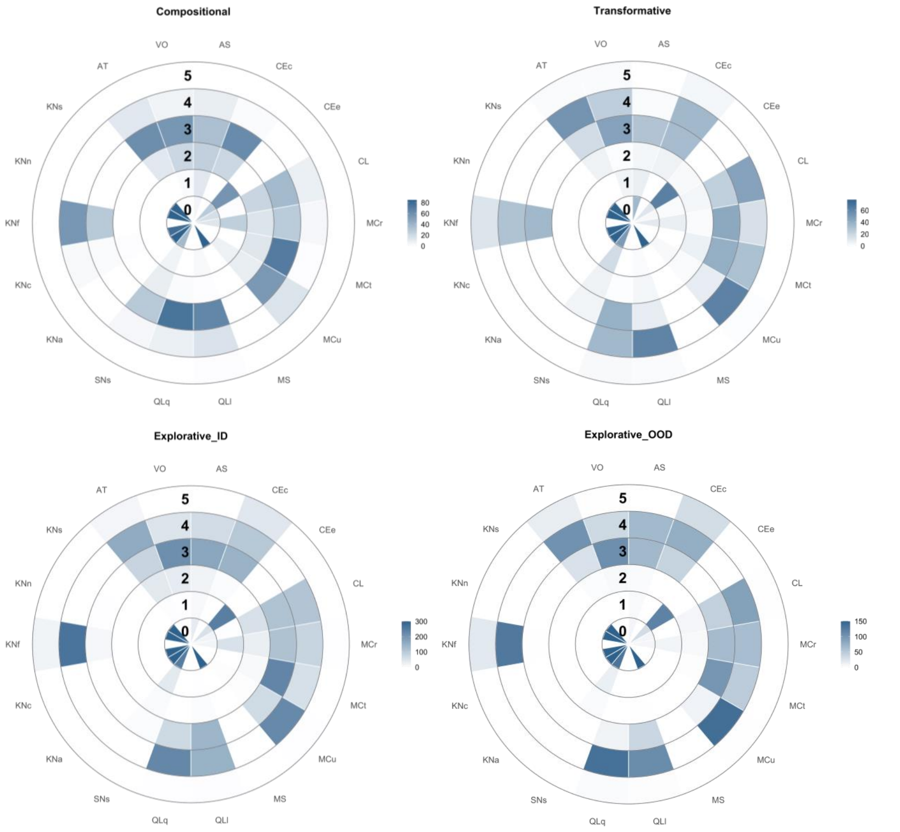

Supplementary Figure 19: Benchmark profiles for mathematical problems in OMEGA [187] including arithmetic and algebra, combinatorics and number-theory, logic&puzzles and geometry, arranged by compositional, explorative (in-distribution and OOD datasets) and transformative character. Their demands are narrowly concentrated around levels 3-4 in reasoning dimensions and formal knowledge. These are not confounded by SNs or MS. However, CEc (comprehension) plays a very important role in the Explorative split. The difference between ID and OOD for Explorative can be traced to the atypicality (AT) change, explaining the difference in performance for OOD problems, all the other things remaining almost equal.

OMEGA *[187].* OMEGA is a large suite of mathematical problems (arithmetic, algebra, combinatorics, number theory, logic and geometry) categorised into compositional, explorative, and transformative groups, with difficulty tagging and ID/OD (in-distribution / out-of-distribution) splits. Supplementary Figure 19 shows the demand profiles when merging the problems into the three categories (keeping ID and OOD separately).

Again, we see the same phenomenon of concentration in the reasoning dimensions, but with fewer confounders than in the previous benchmarks. The level and range of CEc is noteworthy in this case. The demand levels of these benchmarks are not very different from Math and Omni-math, shown in Figure 2, but some dimensions about reasoning are higher, yet more narrow, making the detection of different levels of reasoning capabilities harder, and leading to conflicting interpretations.

The use of difficulty levels is very positive, but for many tasks, an "intrinsic difficulty" is hard to extract, and is only unidimensional, with different scales and conceptualisation across benchmarks. We give a more complete account in the related work section (1.1). Unlike IRT or intrinsic difficulty, with the DeLeAn annotation scales we can flexibly deal with any task and put it into commensurate difficulty levels for all the dimensions in the taxonomy.

1.12.5. Summarising the Evidence and Analyses of LLM "Reasoning"

DeLeAn profiles for reasoning benchmarks (Supplementary Figures 17, 18 and 19) show that positive claims of reasoning frequently arise under low or mid-level reasoning demands. With that in mind, the analysis with DeLeAn debunks the frequent claim that "high score" on a reasoning dataset means high-level reasoning. For negative claims of

reasoning, high-level reasoning demands may be present, although in many cases, non-reasoning dimensions dominate variance and/or difficulty. The profiles also explain why some systems can do well on these batteries while struggling on other datasets *assumed* to be measuring the same thing.

Moreover, across these benchmarks, we detect a consistent pattern: the distribution of reasoning demands is narrow, implying that sensitivity to reasoning is low, even when specificity for this dimension is good (as observed in USAMO or OlymMATH). As a result, reasoning ability estimates based on these benchmarks extrapolate poorly with predictions about reasoning performance in harder or easier instances.

Across this collection of so-called "reasoning" benchmarks, the apparent contradictions collapse into various recurring misunderstandings or "myths". Some benchmarks indeed evaluate upper-level reasoning with grading aligned with response and chain-of-thought quality (e.g., USAMO25); others make the benchmark "hard" mainly through knowledge breadth, novelty (atypicality) or volume (e.g., SuperGPQA, BBEH and PUZZLE); and many widely cited "reasoning" benchmarks saturate because they focus only on the low or mid levels of our reasoning scales (e.g., GSM8K, SVAMP and MATH500 among others). Supplementary Table 20 summarises these patterns into six myths, states what our framework actually reveals, and lists representative benchmarks that show the pitfalls in each concrete case.

Supplementary Table 20: Myths about "reasoning" evaluation, evidence provided by the DeLeAn profiles, and benchmarks that illustrate each myth.

**Myth**	**What the demand profiles show (Reality)**	**Benchmarks illustrating the Myth**
Benchmarks measure reasoning if their creators say they measure reasoning	Sensitivity gaps (narrow reasoning ranges) and specificity failures (extra unintended dimensions) are common even in benchmarks focussed on "reasoning", which are actually driven by non-reasoning dimensions, rather than systematically varied reasoning demands.	GPQA; GPQA-Diamond; SuperGPQA; BBEH (labels from the community or authors claim "reasoning" while profiles show KN/VO/AT demands)
High accuracy on a dataset labelled as "reasoning" implies strong reasoning	Many "reasoning" batteries show low levels on reasoning dimensions, so high accuracy alone (which usually happens with low levels of many other dimensions, except knowledge, where LLMs are usually good) is not evidence of strong reasoning.	GSM8K; SVAMP; MATH500; GPQA; GPQA-Diamond; SuperGPQA (low or mid reasoning levels, with the variance dominated by knowledge)
Low scores on a dataset labelled as "reasoning" show a lack of reasoning	"Hardness" is frequently increased via extraneous factors (AT or VO), metacognition (MC), broader knowledge (KN), or other non-reasoning dimensions rather than higher reasoning levels alone. Low scores can arise from these confounders, not necessarily from lack of reasoning.	RIMO-P AND BBEH (difficulty raised via MC/KN/VO/AT)
Longer chains-of-thought or "thinking time" demonstrate reasoning	Volume (VO), an extraneous dimension, is responsible for this rather than reasoning. Longer prompts or solutions may correlate with difficulty due to extraneous demands, which does not necessarily lead to higher levels of reasoning, as seen in Supplementary Information 1.12.4.	BBEH; RIMO-P; OlymMATH-Hard; PUZZLE; OMEGA (length of the response or "thinking time" high VO/CE/MC without guaranteeing higher QL)
Raising difficulty automatically raises reasoning demand and no other non-reasoning demands	Difficulty is often raised by increasing knowledge breadth, atypicality/familiarity, or volume, not by placing items at reasoning levels 4–5 while controlling confounders. True reasoning difficulty requires targeted design with high reasoning demands and low non-reasoning loads.	SuperGPQA; OlymMATH EASY → HARD; RIMO-N → RIMO-P; BBEH; PUZZLE; OMEGA (hardness often partly comes from non-reasoning dimensions or extraneous factors)
Performance improvements on mixed demand benchmarks generalise to other reasoning problems	Improvements on datasets with low reasoning sensitivity or high non-reasoning demands typically reflect familiarity, knowledge, or option-elimination heuristics, among other abilities. Such gains do not reliably transfer to advanced reasoning.	GSM8K; SVAMP; MATH500 (improvements consistent with elementary reasoning only, knowledge, or option-elimination, not high-level reasoning)

Designing "clean" instances with reasoning demands at level 4 or 5 without simultaneously raising non-reasoning or extraneous demands is challenging, but we find a few examples scattered through the ADeLe battery, and proportionally more in ADeLe-Light. This is precisely why non-saturating, commensurate, general scales are needed: to detect and quantify what we can attribute to actual reasoning capabilities, and separate increases in those capabilities from other capabilities. Instead of asking whether models reason, we can now ask "how much" and where, and under what non-reasoning demands. The same lens that reconciles all these current contradictory claims also provides a forward path: build new items by targeting uncovered reasoning dimensions levels while holding extraneous factors flat and evaluating improvements as shifts in ability curves instead of leveraging aggregate performances.

1.13. Confidence-interval Width of SCCs by Dimension and Level

Supplementary Table 21 shows the width of CIs by dimension and level pairs (averaged across subject models) for the SCCs in Figure 3. As can be seen, these numbers are generally small. Still, one should be aware of that the logistic model is fitted with all the points, not the mean of each level, so these CI are less relevant, as we are not using the empirical curves level-wise but a parametric fit across all levels.

Supplementary Table 21: Mean 95% confidence-interval width by level and dimension (averaged across models). Level 1 is omitted as the number of instances with all demand dimensions below 2 is negligible.

**Level**	`AS`	`CEc`	`CEe`	`CL`	`MCr`	`MCt`	`MCu`	`MS`	`QLl`	`QLq`	`SNs`	`KNa`	`KNc`	`KNf`	`KNn`	`KNs`	`AT`	`VO`
2	0.031	0.039	0.117	0.058	0.105	0.028	0.030	0.090	0.038	0.036	0.171	0.013	0.021	0.016	0.025	0.038	0.025	0.055
3	0.034	0.033	0.109	0.036	0.053	0.026	0.029	0.076	0.028	0.038	0.100	0.014	0.019	0.016	0.025	0.030	0.022	0.053
4	0.034	0.034	0.125	0.027	0.048	0.027	0.035	0.091	0.022	0.051	0.060	0.015	0.020	0.018	0.023	0.024	0.032	0.058
5	0.017	0.042	0.138	0.028	0.068	0.033	0.030	0.174	0.026	0.063	0.054	0.021	0.030	0.021	0.024	0.032	0.040	0.054

1.14. SCCs for all models

From Supplementary Figure 20 to Supplementary Figure 34, we show the individual characteristic curves for all 15 subject LLMs, starting from OpenAI's o1 and GPT models, followed by Meta's LLaMA-3 saga, and ending up with the DeepSeek-R1-Distilled-Qwen family. Overall, the logistic fits are quite good for most dimensions, with the only exception of `SNs` (`Spatial Reasoning and Navigation - Spatial`) for various models, which can be attributed to the small number of instances in level 1 and 2; this can be improved in `ADeLe` v2.0. Between all subjects, the fits of Davinci-002 and Babbage-002 are comparably worse. This is expected, given the lack of instruction-tuning for these two models (Extended Data Table 1), meaning that they frequently repeat the prompts instead of solving the problems specified in the prompts, in an seemingly elusive and arbitrary way.

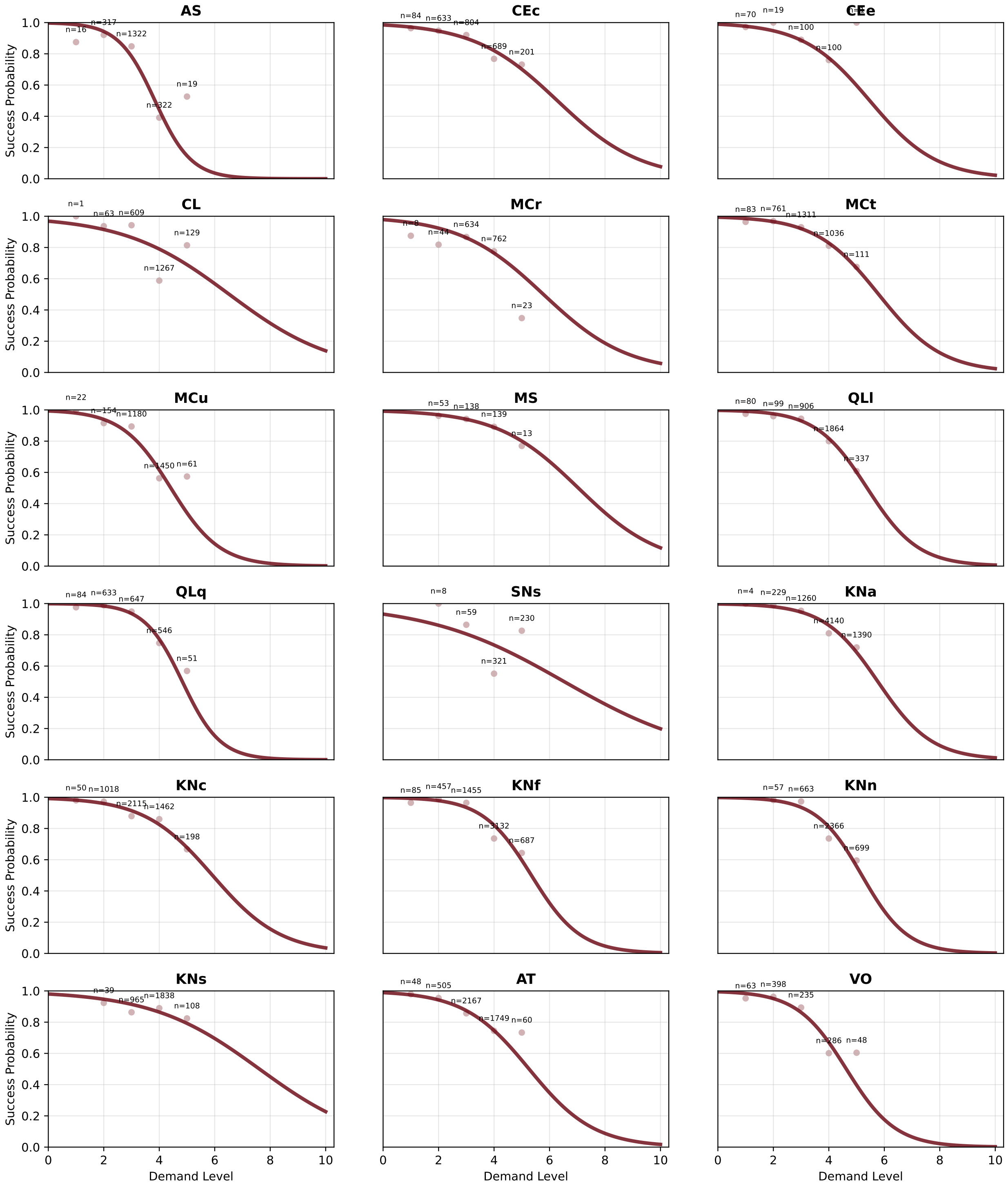

Supplementary Figure 20: Characteristic curves for the 18 demands for OpenAI's o1 (all other things equal to Figure 3).

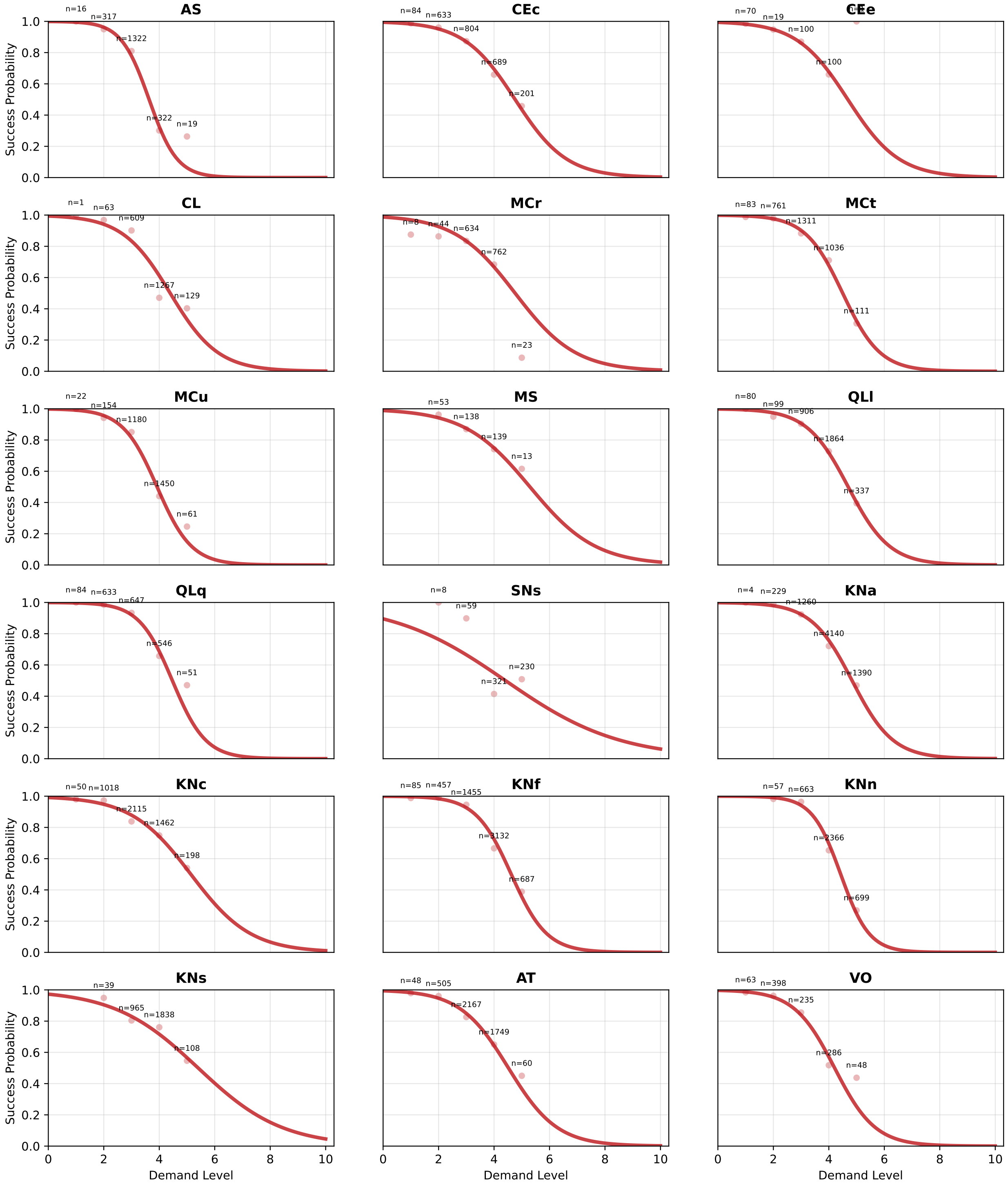

Supplementary Figure 21: Characteristic curves for the 18 demands for OpenAI's o1-mini (all other things equal to Figure 3).

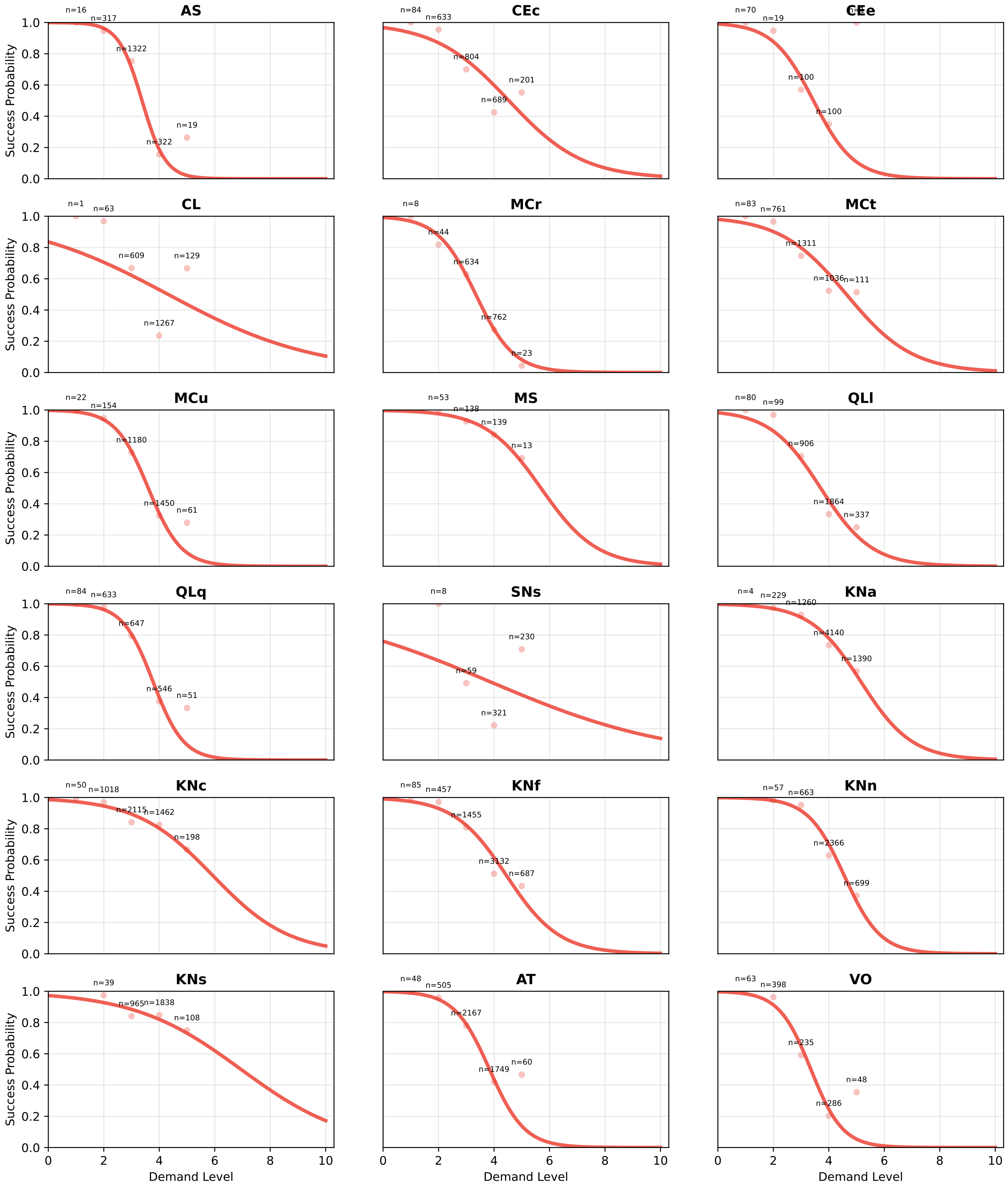

Supplementary Figure 22: Characteristic curves for the 18 demands for OpenAI's GPT-4o (all other things equal to Figure 3).

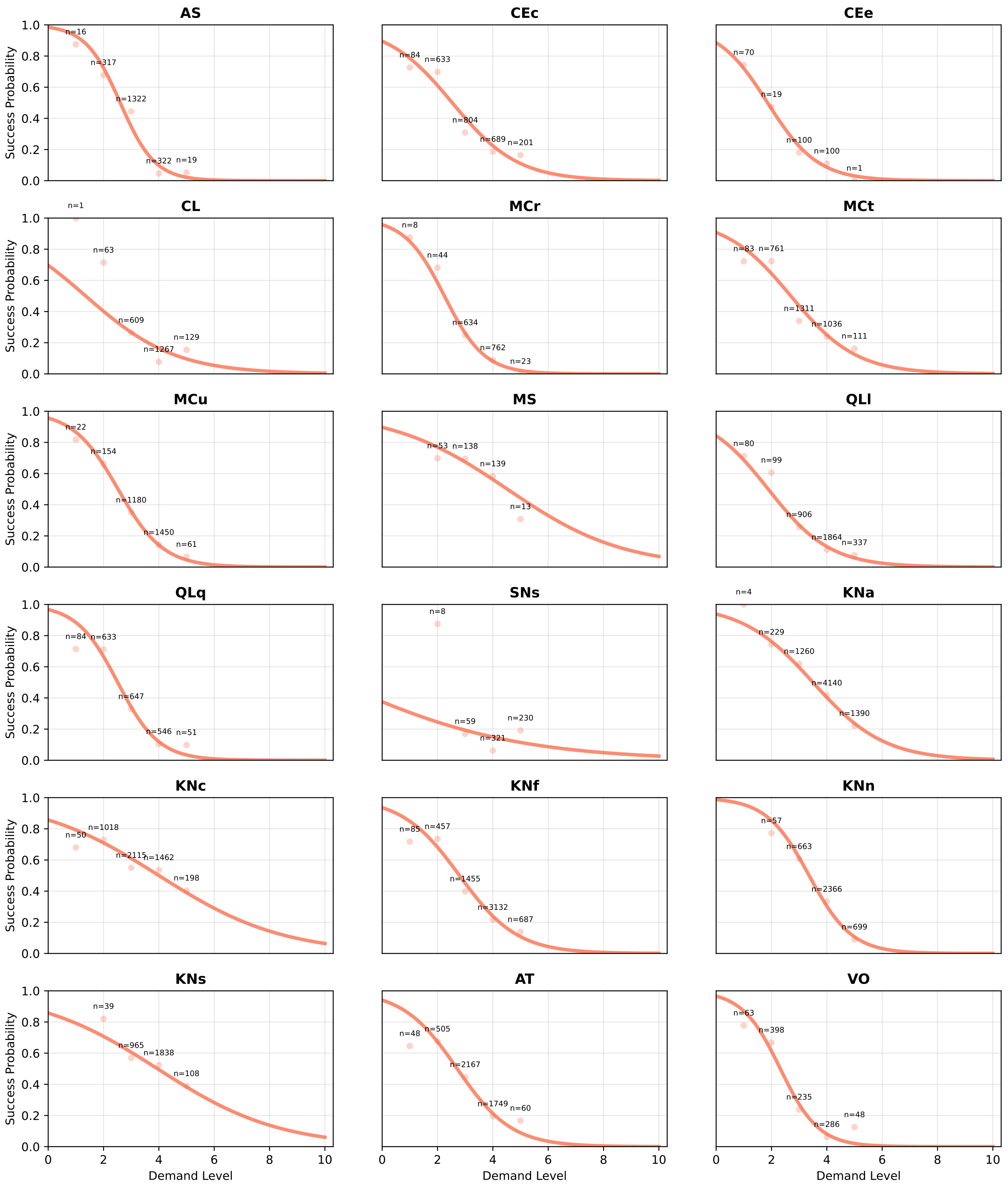

Supplementary Figure 23: Characteristic curves for the 18 demands for OpenAI's GPT-3.5-Turbo (all other things equal to Figure 3).

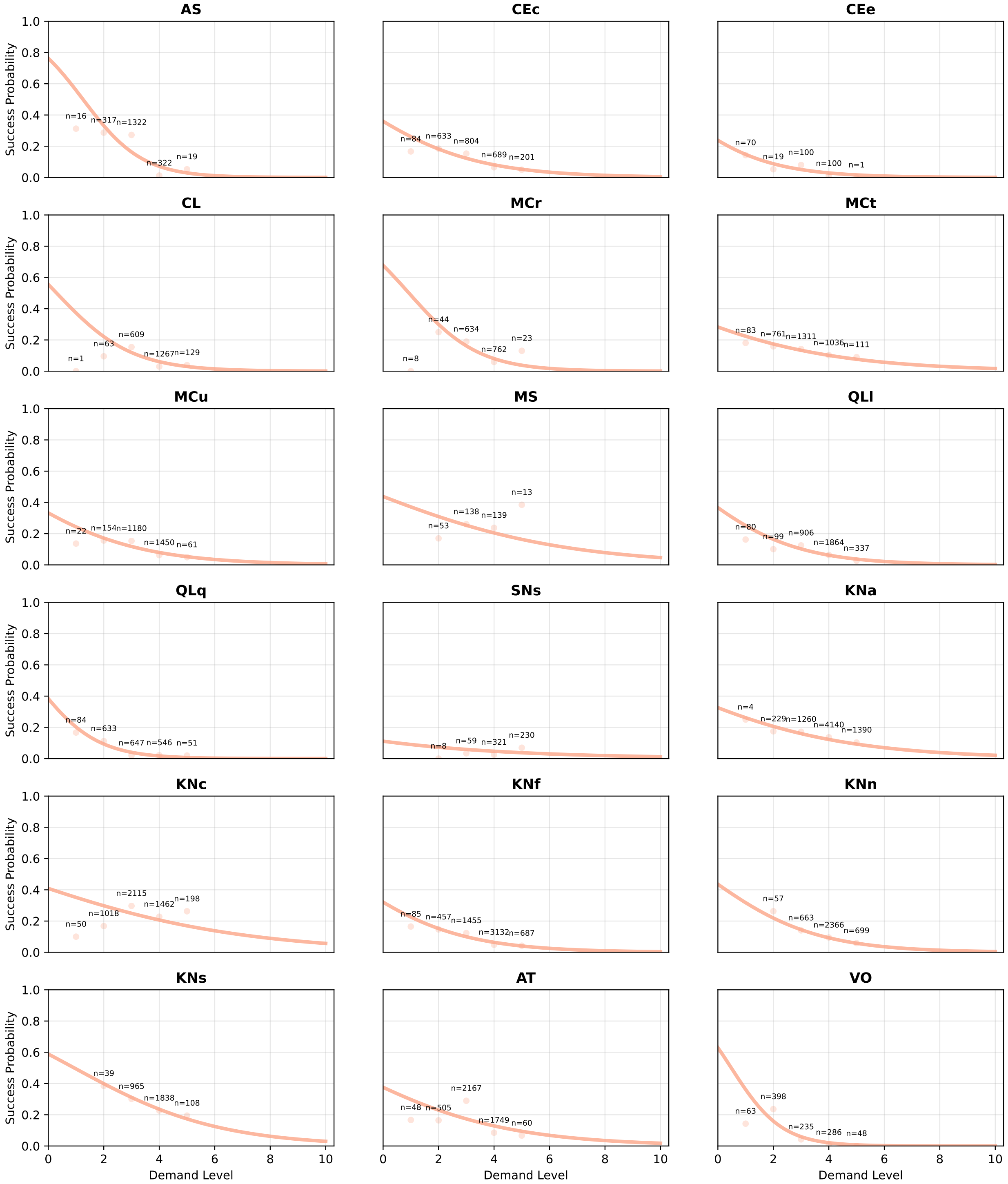

Supplementary Figure 24: Characteristic curves for the 18 demands for OpenAI's Davinci-002 (all other things equal to Figure 3).

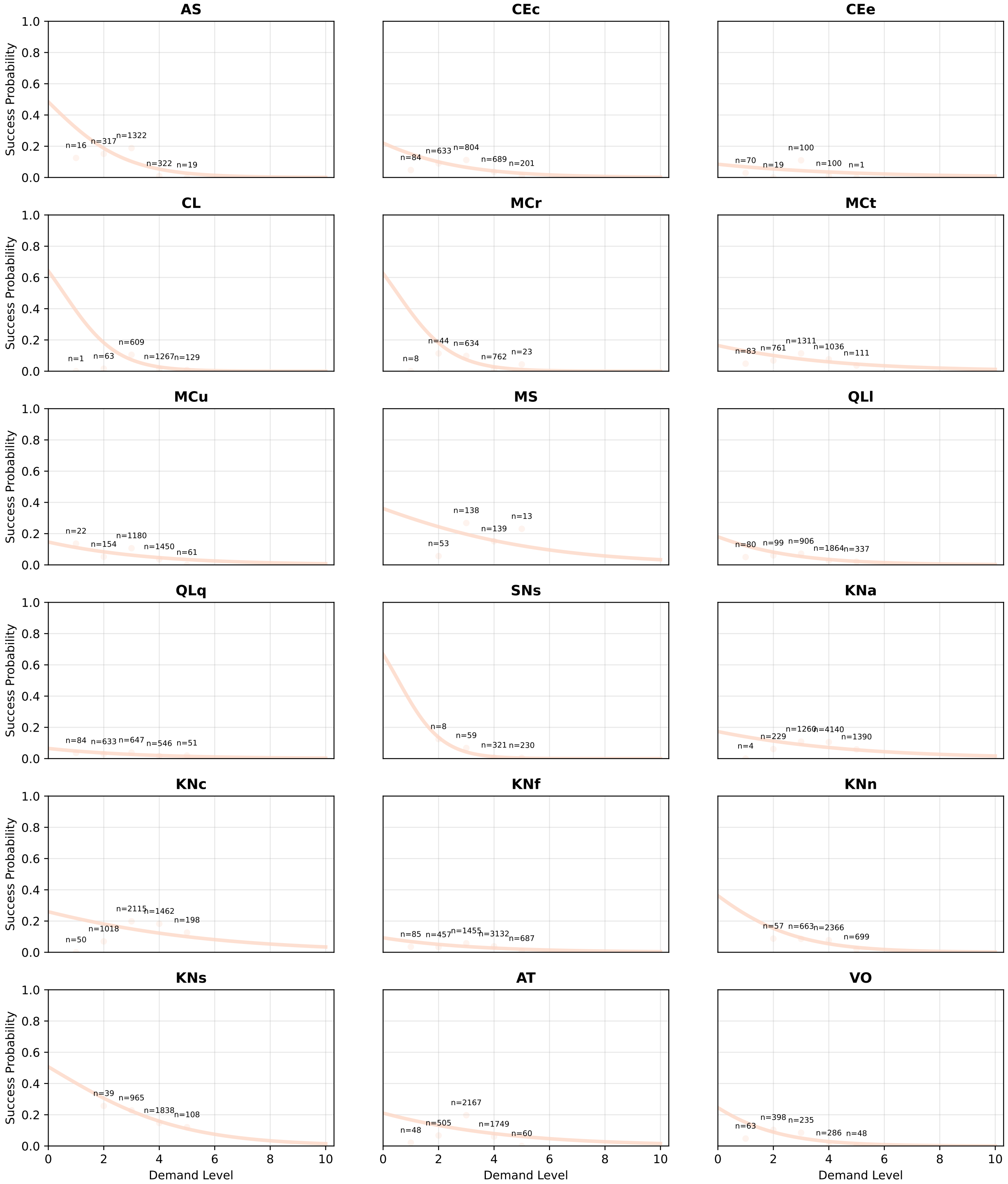

Supplementary Figure 25: Characteristic curves for the 18 demands for OpenAI's Babbage-002 (all other things equal to Figure 3).

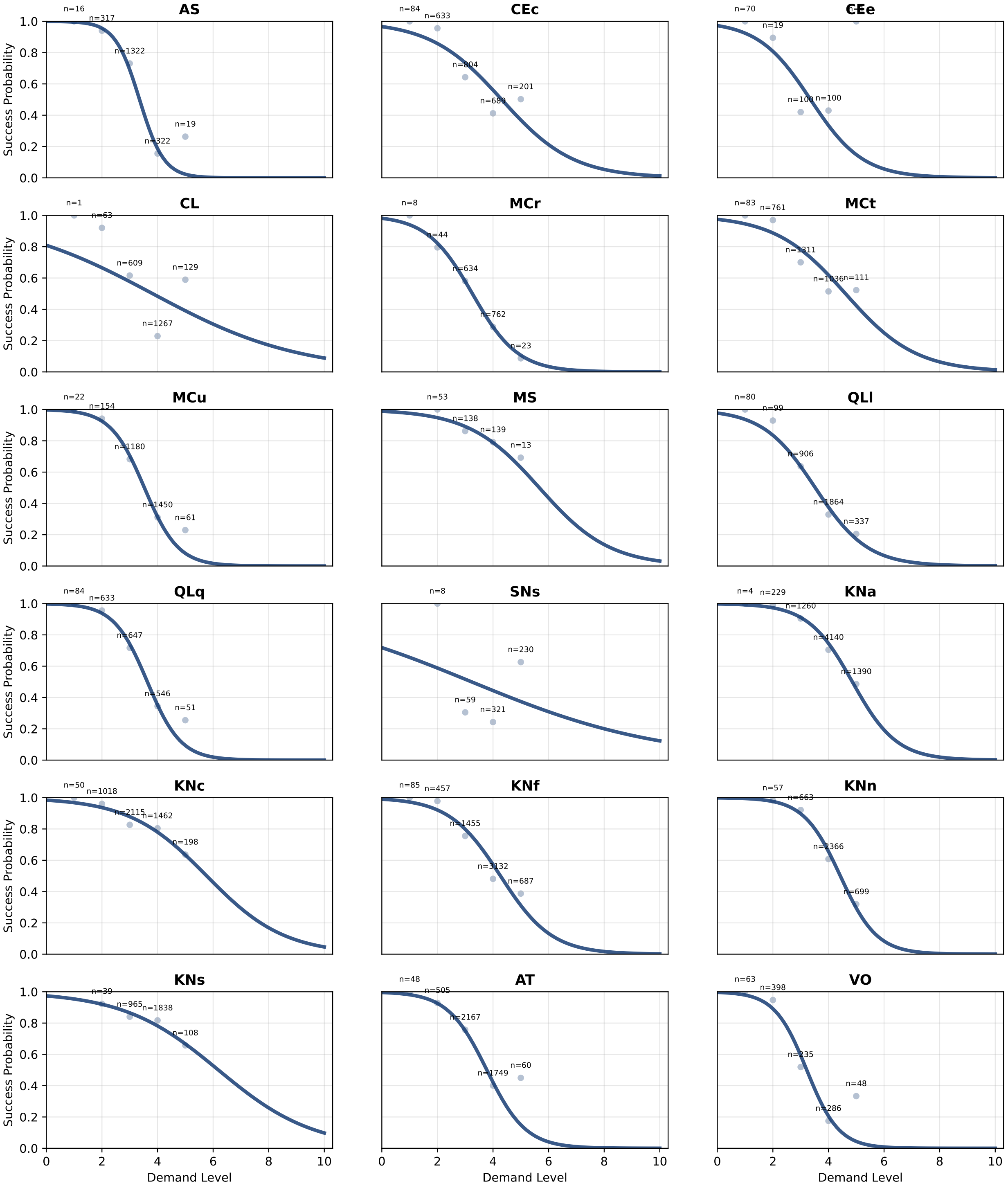

Supplementary Figure 26: Characteristic curves for the 18 demands for LLaMa 3.1-405B-Instruct (all other things equal to Figure 3).

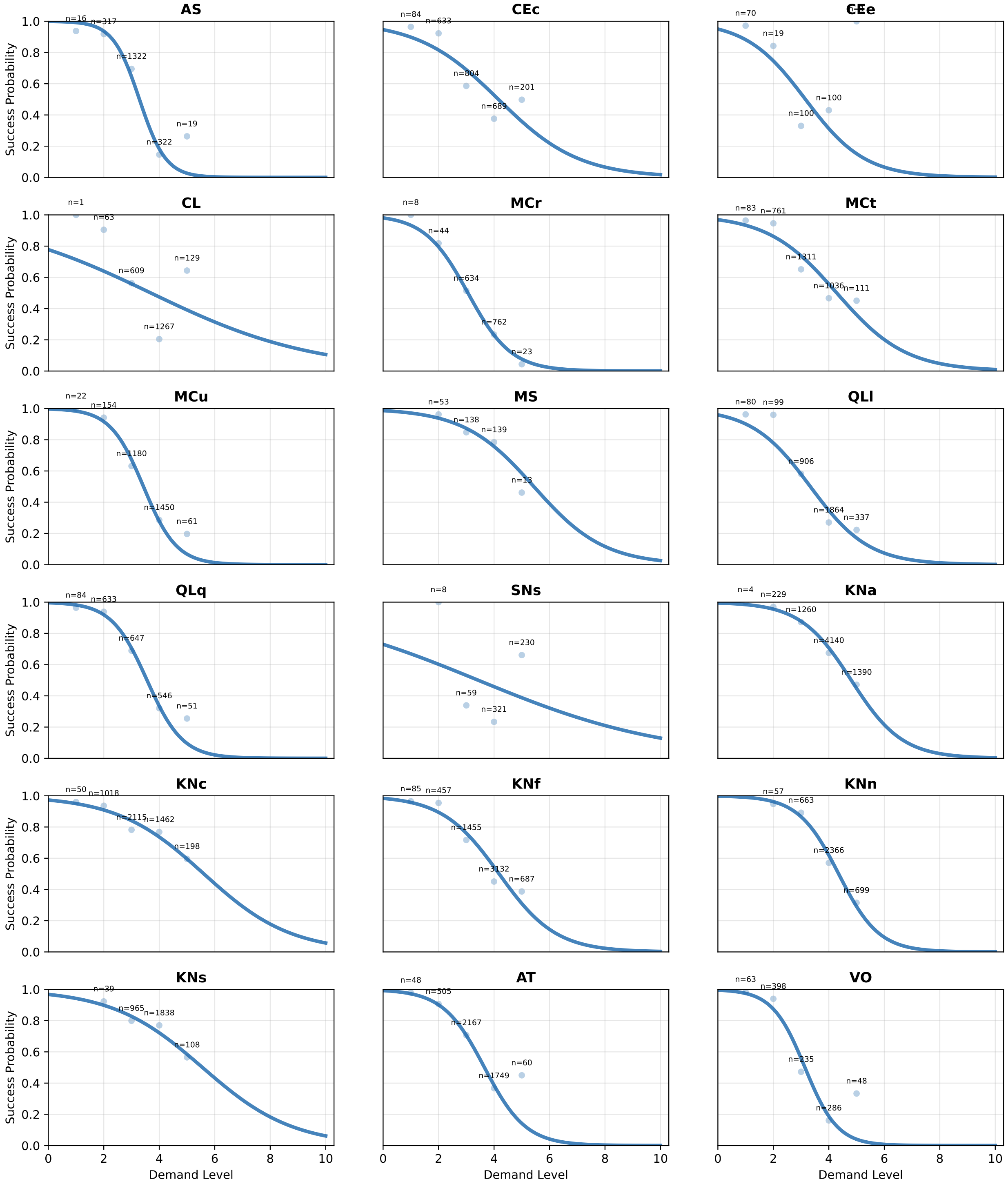

Supplementary Figure 27: Characteristic curves for the 18 demands for LLaMa 3.2-90B-Instruct (all other things equal to Figure 3).

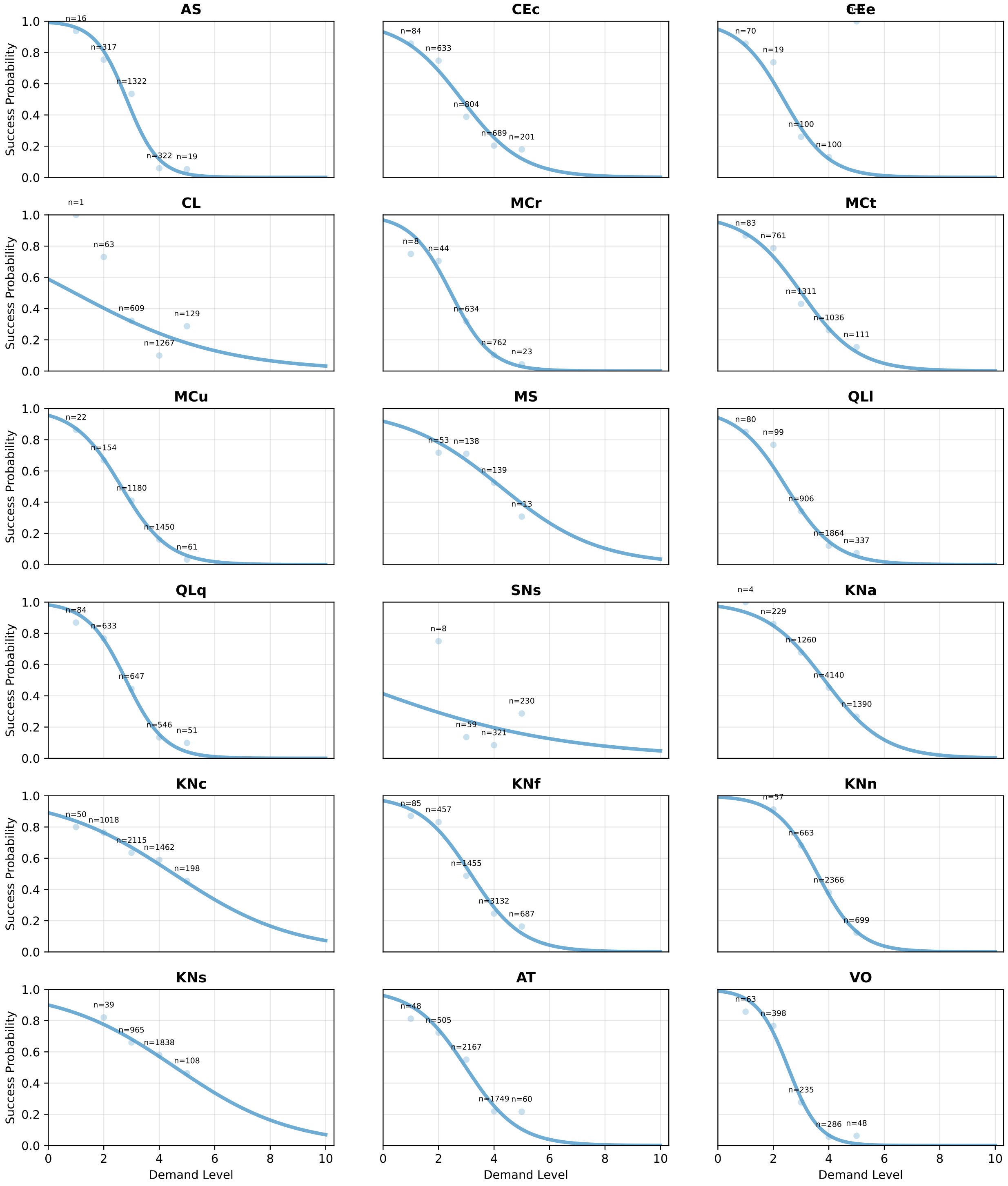

Supplementary Figure 28: Characteristic curves for the 18 demands for LLaMa 3.2-11B-Instruct (all other things equal to Figure 3).

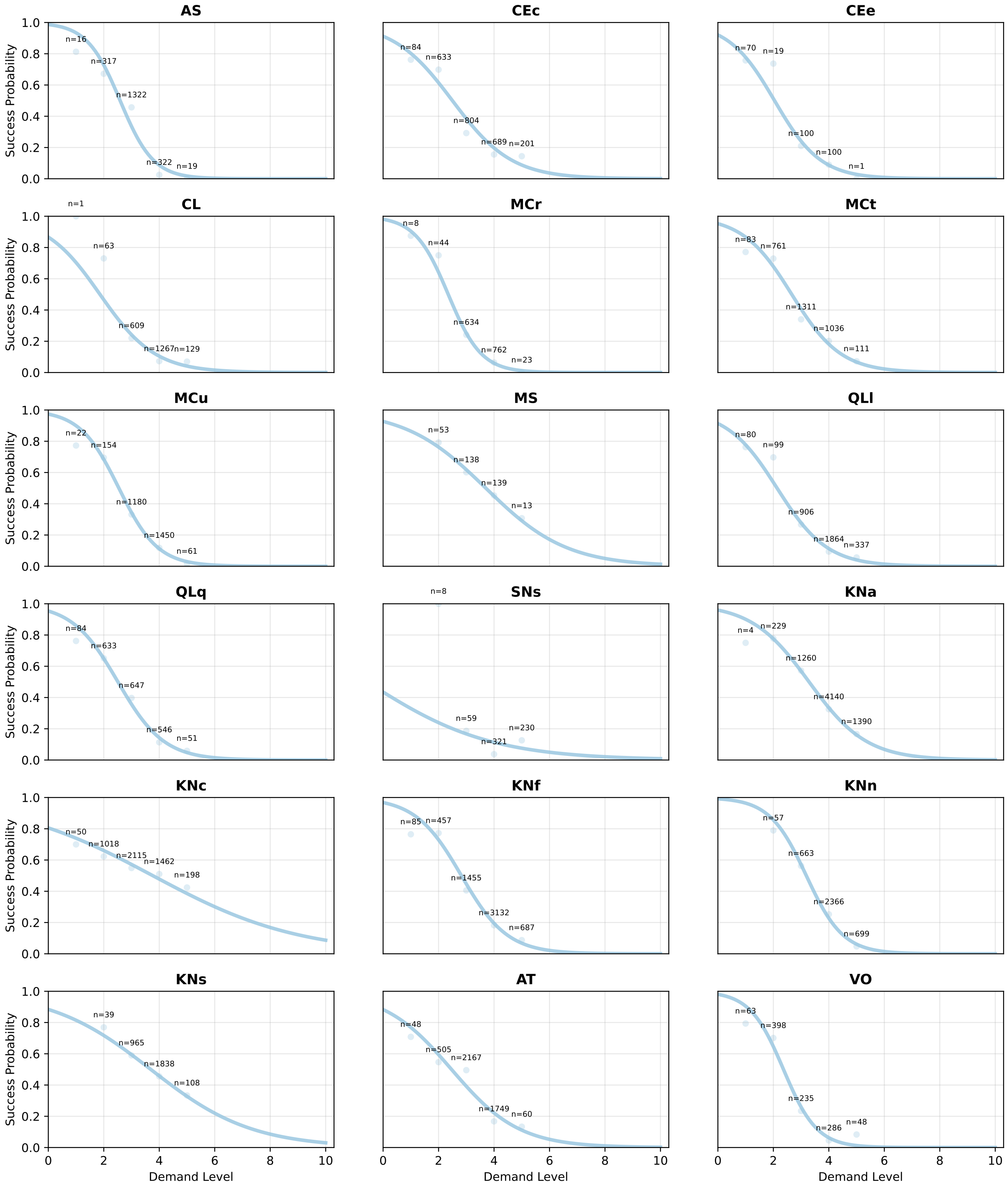

Supplementary Figure 29: Characteristic curves for the 18 demands for LLaMa 3.2-3B-Instruct (all other things equal to Figure 3).

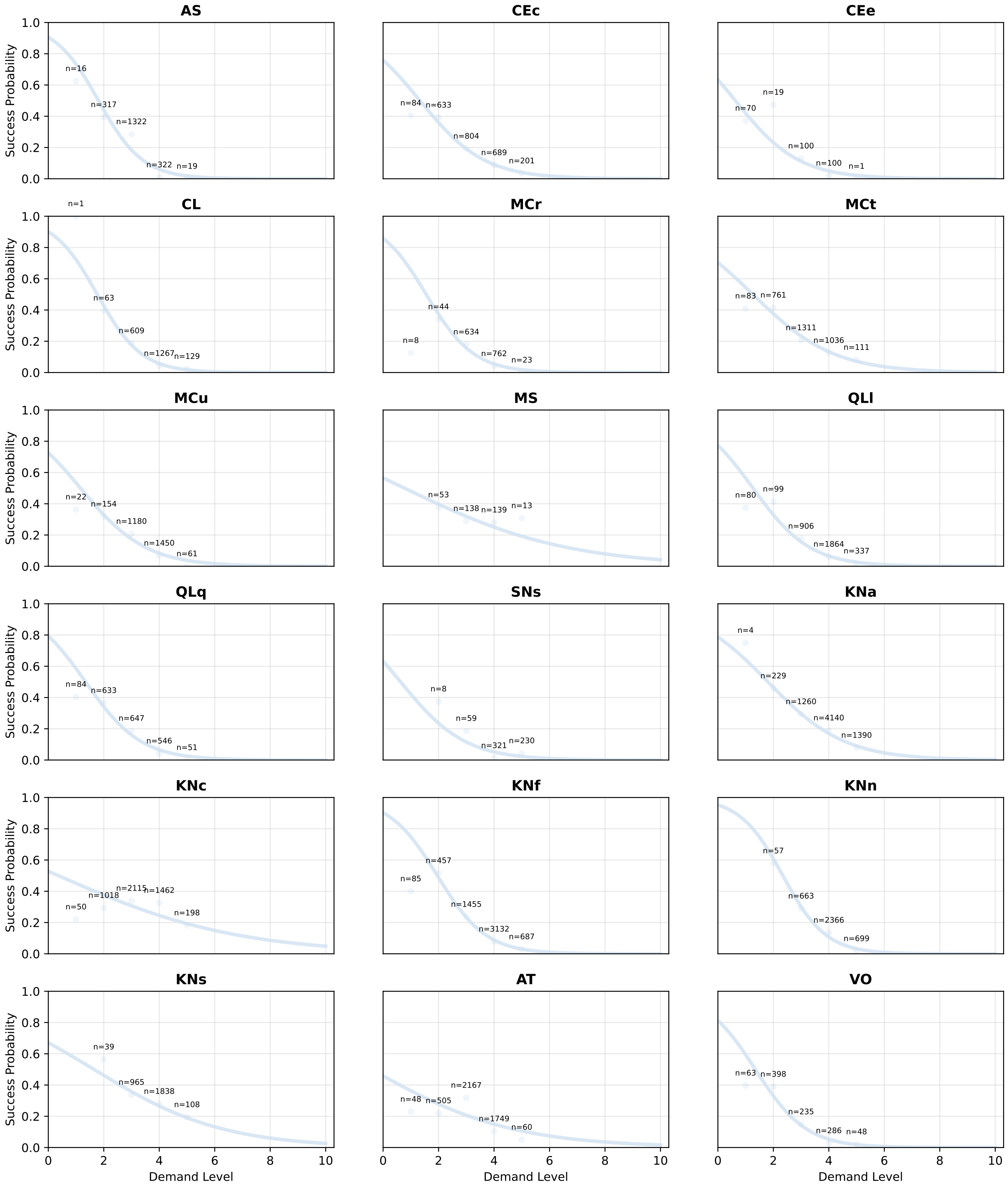

Supplementary Figure 30: Characteristic curves for the 18 demands for LLaMa 3.2-1B-Instruct (all other things equal to Figure 3).

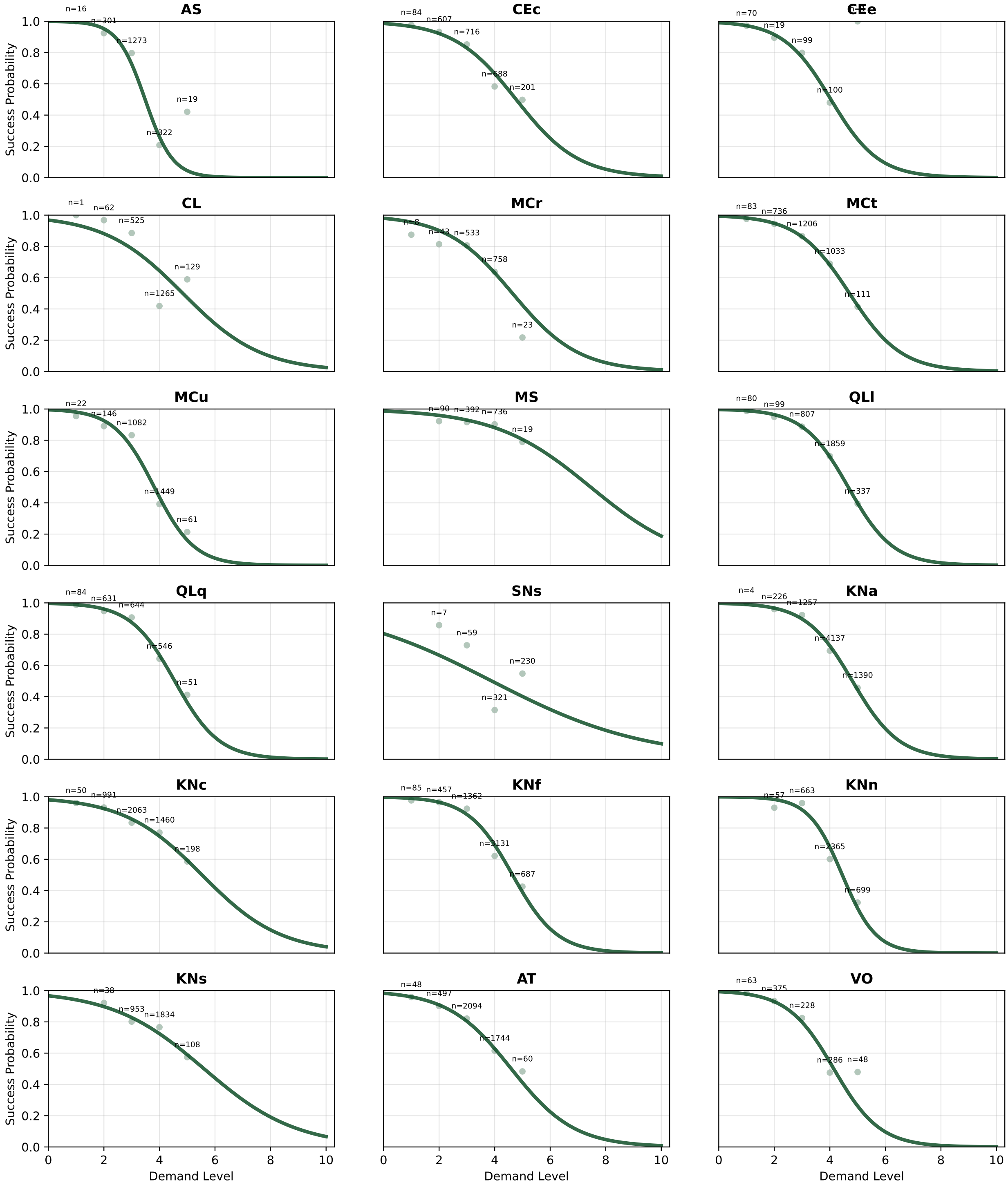

Supplementary Figure 31: Characteristic curves for the 18 demands for DeepSeek's R1-Dist-Qwen-32B (all other things equal to Figure 3).

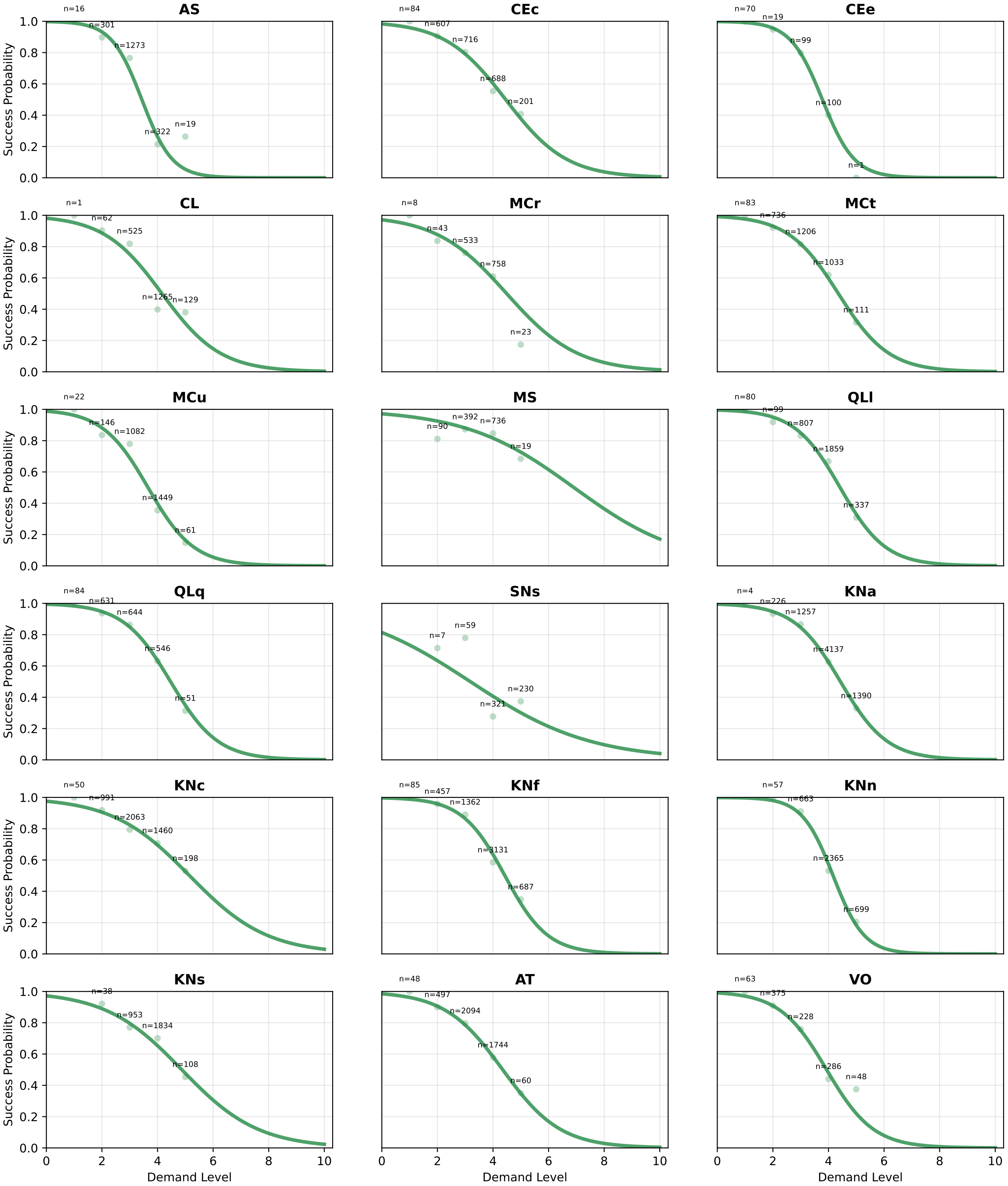

Supplementary Figure 32: Characteristic curves for the 18 demands for DeepSeek's R1-Dist-Qwen-14B (all other things equal to Figure 3).

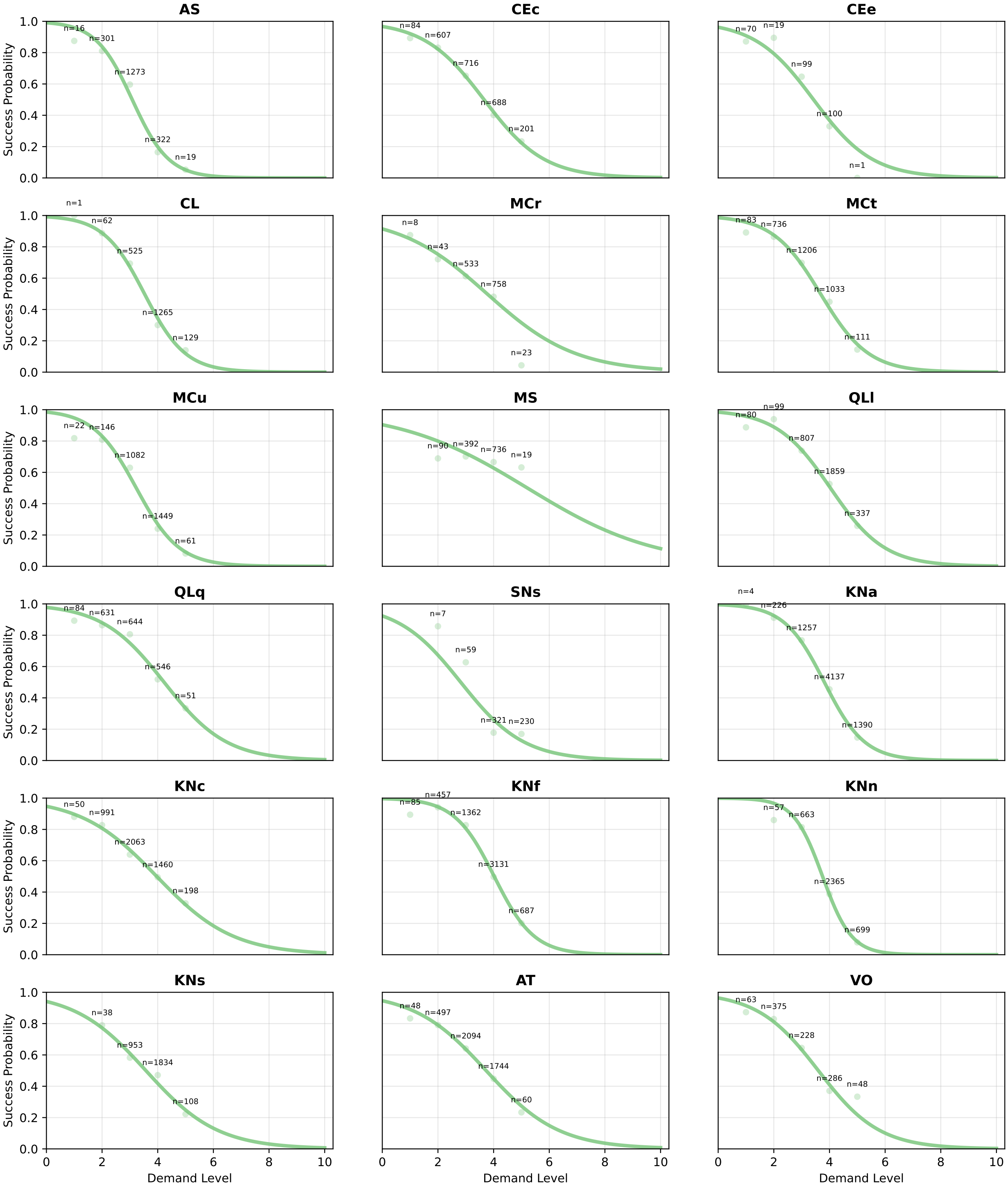

Supplementary Figure 33: Characteristic curves for the 18 demands for DeepSeek's R1-Dist-Qwen-7B (all other things equal to Figure 3).

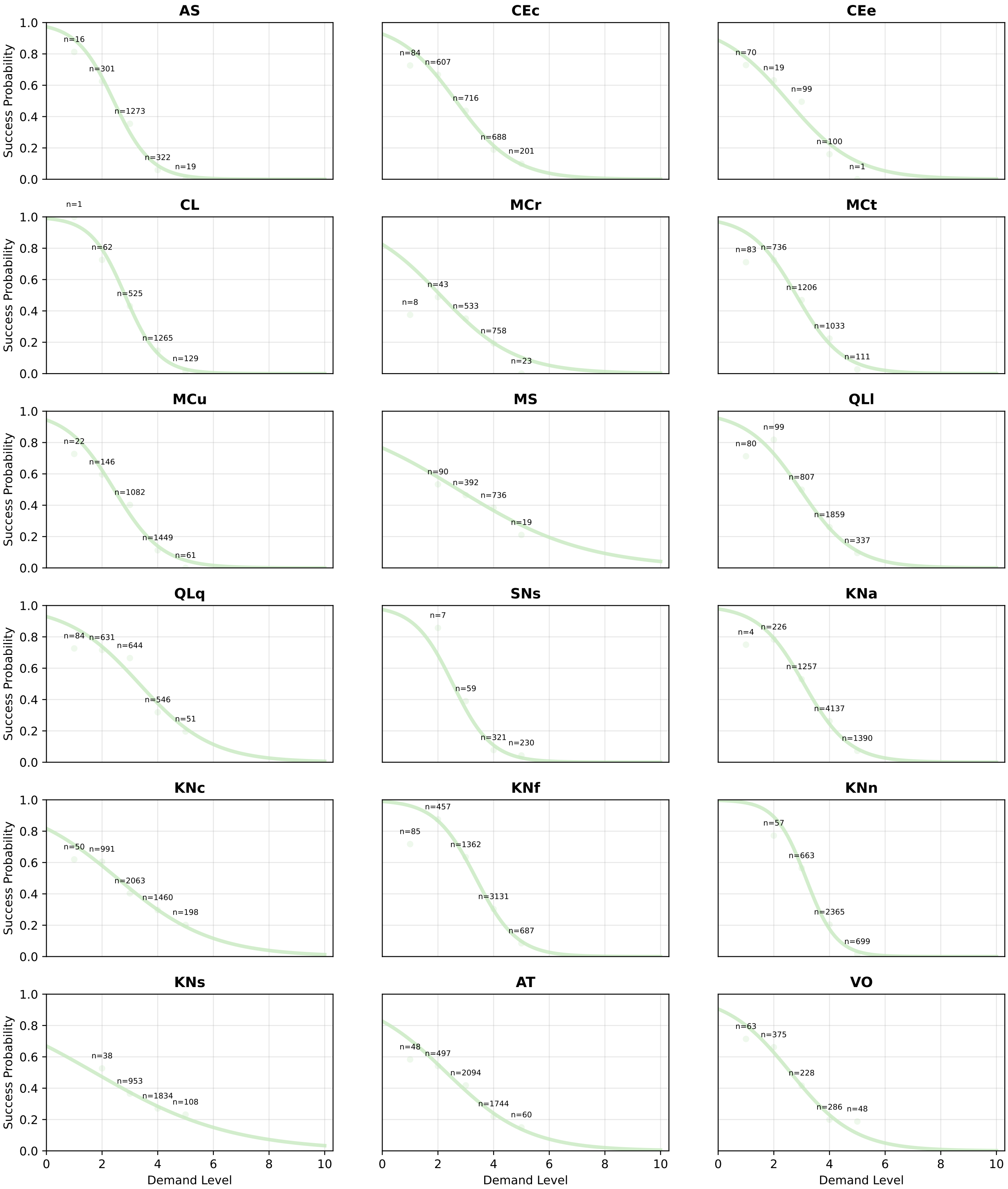

Supplementary Figure 34: Characteristic curves for the 18 demands for DeepSeek's R1-Dist-Qwen-1.5B (all other things equal to Figure 3).

1.15. Inter-rater Agreement for Humans and GPT-4O

Here we show the inter-rater agreements for humans and GPT-4o, our main annotator.

Supplementary Table 22: Row "Humans" shows the agreement of ratings (r_{WG} scores) obtained between humans prior to Delphi consensus, while "Delphi & GPT-4o" reports the agreement between Delphi consensus and GPT-4o.

	AS	CEc	CEe	CL	MCr	MCt	MCu	MS	QLl	QLq	SNs	KNa	KNc	KNf	KNn	KNs	AT	VO	**Average**
**Humans**	0.91	0.91	0.90	0.78	0.79	0.88	0.80	0.77	0.85	0.84	0.87	0.73	0.86	0.86	0.91	0.70	0.80	0.84	**0.83**
**Delphi & GPT-4o**	0.86	0.87	0.94	0.82	0.84	0.91	0.81	0.86	0.89	0.84	0.89	0.75	0.83	0.81	0.94	0.86	0.83	0.91	**0.86**

1.16. Prompt templates

This section includes the prompt templates using for annotations, accuracy, objectivity, unambiguity and for the graders to verify the outputs of the subject LLMs.

Supplementary Table 23: Prompt template for deriving demand annotations with GPT-4o.

```
QUERY: The following rubric describes six distinct levels of *{$dimension$}*
required by different tasks:
{$rubric$}

TASK INSTANCE: {$instance$}

INSTRUCTION: Score the level of *{$dimension$}* demanded by the given
TASK INSTANCE using a discrete value from 0 to 5. Use CHAIN-OF-THOUGHTS
REASONING to reason step by step before assigning the score. After the
CHAIN-OF-THOUGHTS REASONING STEPS, conclude your assessment with the
statement: "Thus, the level of *{$dimension$}* demanded by the given TASK
INSTANCE is: SCORE", where 'SCORE' is the integer score you have determined.
```

Supplementary Table 24: Prompt template for evaluating the factual accuracy of the ground truth labels recorded with task items.

```
QUERY: {$query$}

ANSWER: {$answer$}

INSTRUCTION: Given the query and an answer above, evaluate the accuracy or
factuality of this answer. Please rate it using a Likert scale from 1 to 5,
where 5 means surely accurate while 1 means surely inaccurate. You may employ
any reasoning strategy before providing your final answer. However, you must
conclude your assessment with the statement: 'Thus, the accuracy of the given
answer is: X', where 'X' is the score you've determined.
```

Supplementary Table 25: Prompt template for evaluating the objectivity of task items.

```
QUERY: {$query$}

INSTRUCTION: Given the query above, evaluate its objectivity. That is,
assess whether this query can be answered objectively - would different
qualified individuals arrive at the same answer regardless of their personal
views or preferences? Please rate it using a Likert scale from 1 to 5, where
5 means surely objective while 1 means surely subjective. You may employ
any reasoning strategy before providing your final answer. However, you must
conclude your assessment with the statement: 'Thus, the objectivity of the
given query is: X', where 'X' is the score you've determined.
```

Supplementary Table 26: Prompt template for evaluating the unambiguity of task items.

```
QUERY: {$query$}

INSTRUCTION: Given the query or question above, evaluate the ambiguity of
this query, independently of the potential difficulty of answering it. That
is, assess whether the query has a single clear interpretation with all
necessary details provided, or if it lacks critical information making it
difficult to answer confidently. Please rate it using a Likert scale from
1 to 5, where 5 means surely unambiguous (very clear) while 1 means surely
ambiguous (very unclear). You may employ any reasoning strategy before
providing your final answer. However, you must conclude your assessment with
the statement: 'Thus, the ambiguity of the given query is: X', where 'X' is
the score you've determined.
```

Supplementary Table 27: Prompt template used for LLM graders to verify the outputs of subject LLMs, scaled from 1 to 5.

```
TASK: {$prompt$}

MODEL RESPONSE: {$model_response$}

GROUND TRUTH: {$ground truth$}

INSTRUCTION: You are an evaluation system that compares a MODEL
RESPONSE to a GRound truth of a given TASK (as shown above), and rates
the response on a scale of 1 to 5, where 5 means surely correct and 1
means surely incorrect. Your response must be concise, responding with
exactly one integer score between 1 and 5 and nothing else that may be
redundant.
```

1.17. Benchmarks in ADeLe v.1.0

Supplementary Table 28 shows the 20 benchmarks included in ADeLe v.1.0.

Supplementary Table 28: Overview of the selected sources, benchmarks and tasks in the ADeLe battery. Total number of instances: 16,108. We also indicate what they claim to measure according to the original source in Table 2 to determine their sensitivity and specificity.

Source	Benchmark	Task	Claiming to Measure	#Instances
AGIEval [188]	Civil Service Examination	LogiQA-en	Logical Reasoning	408
	GRE & GMAT	AQuA-RAT	Mathematics	203
	LSAT	LSAT-AR	Analytical Reasoning	187
		LSAT-LR	Logical Reasoning	470
		LSAT-RC	Reading Comprehension	253
	SAT	SAT-En	Critical thinking, problem-solving and analytical skills	196
		SAT-Math		214
ChemLLMBench [189]	ChemLLMBench	Molecule Captioning	Generation of descriptions for molecules	160
		Molecule Design	Generation of new molecules given a description	295
		Name Prediction	Chemical name understanding	476
		Reaction Prediction	Chemical reaction products prediction	412
		Retrosynthesis	Identification of efficient synthetic pathways for target molecules	380
LiveBench [190]	Data Analysis	CTA	Data Analysis	33
	Language	Connections	Language Comprehension	29
	Math	AMPS Hard	Mathematics	69
		Math Competition		78
		Olympiad		26
	Reasoning	Spatial	Spatial Reasoning	34
		Zebra Puzzle	Logical Reasoning	22
MMLU-Pro [191]	MMLU-Pro	Biology	Knowledge and Reasoning	447
		Business		410
		Chemistry		368
		Computer Science		345
		Economics		428
		Engineering		296
		Health		411
		History		304
		Law		362
		Math		425
		Other		429
		Philosophy		402
		Physics		377
		Psychology		427
MedCalcBench [192]	MedCalcBench	Date	Recall of medical calculation knowledge<br>Extraction of relevant patient attributes<br>Arithmetic computation of final results	27
		Diagnosis		14
		Dosage		20
		Lab		180
		Physical		214
		Risk		84
		Severity		17
OmniMath [193]	OmniMath	Algebra	Mathematical reasoning at Olympiad level	337
		Applied Mathematics		302
		Calculus		30
		Discrete Mathematics		314
		Geometry		329
		Number Theory		322
		Precalculus		30
SciBench [194]	SciBench	Chemistry	Scientific problem-solving	142
		Math		105
		Physics		108
TimeBench [195]	Date Arithmetic	Date Arithmetic	Symbolic temporal reasoning	493
	MCTACO	MCTACO	Commonsense temporal reasoning	205
	MenatQA	MenatQA-Counterfactual	Event temporal reasoning	130
		MenatQA-Order		157
		MenatQA-Scope		393
	TempReason	TempReason-L2	Event temporal reasoning	318
		TempReason-L3		339
	TimeDial	TimeDial	Commonsense temporal reasoning	340
	TimeQA	TimeQA-explicit	Event temporal reasoning	379
		TimeQA-implicit		348
TruthQuest [196]	TruthQuest	E	Suppositional reasoning	344
		I		371
		S		340

1.18. Cost, Ethical and Safety Implications

Our methodology is designed to be scalable, cost-effective and adaptive, with its primary costs divided between two core processes: (1) the *System Process*, for evaluating a new AI system; and (2) the *Task Process*, for evaluating a new task or benchmark (see Figure 7).

The *System Process* is performed once per AI system. Its dominant cost lies in running the AI model on the 16,108 instances of the ADeLe battery (Step 1), which can range from a few GPU-hours for smaller models to hundreds or thousands of GPU-hours (or equivalent cloud API costs) for large language models like GPT-4. In practice, this can be reduced to about a third with the ADeLe-Light version of the battery, while resulting in a slight loss in precision. This is substantially lightweight in comparison with the typical evaluations taking place at major AI labs and regulatory bodies. Subsequent steps, such as grading outputs with the provided scorer and generating characteristic curves (Step 2), are computationally inexpensive, requiring only a few CPU-hours or less. In addition, the optional step of training a simple assessor (Step 3) using traditional machine learning algorithms incurs minimal CPU cost (in our case 4 seconds in a M3 Pro CPU). For our experiments, only as part of the set of baselines, we fine-tuned LLaMA, which is, by far, much more expensive than our proposed methodology, taking thousands of GPU hours (in our case 300 GPU hours on a single V100 GPU for one run). Human effort in the System Process is minimal, primarily involving setup and monitoring of automated processes.

The *Task Process* is performed once for each new task or benchmark. Its computational cost is primarily driven by the annotation of task instances using an LLM together with the DeLeAn rubrics (Step A). Although the cost depends on the model used (e.g., GPT-4o vs. DeepSeek), the rapid decrease in inference costs in recent years mitigates this cost. After LLM-based annotation, the subsequent data preparation and analysis processes require negligible computational resources. In contrast, human effort for the task process is largely focused on the prompt design for the subject LLM and data preparation (which has to be done for any evaluation) as well as the analysis and interpretation of the demand profiles (Step B). Although this can involve anywhere from various hours to a few days of expert time, these are one-off investments that benefit from future reuse and further automation. Predicting performance using an assessor (Step C) is computationally very cheap. The methodology is designed for high automation to minimise recurring human intervention, and focus the expert time on the design of rubrics for new dimensions (e.g., propensities) and the interpretation of results.

Developing robust rubrics, as we have done here, requires a significant initial investment of time and expert effort, involving interdisciplinary collaboration as well as iterative testing and validation to ensure that they accurately capture the important dimensions of task difficulty. However, once these rubrics have been established and validated, their use is highly efficient and cost-effective. New instances can be quickly annotated using automated pipelines that incorporate the rubrics. We highly recommend to capitalise on our battery ADeLe as a standard, which can be extended collaboratively to share the effort.

We should also recognise that our dimensional approach reduces complex behaviour to a fixed set of measurable constructs. This reduction is an inherent limitation in any evaluation framework, and can introduce biases, especially in the knowledge domains. These are not all the dimensions that affect behaviour. For example, in risk or safety sensitive applications, additional dimensions such as context-specific propensities may need to be included. In such cases, the scales are not necessarily monotonic (as in personality, where the extremes are generally bad) and may need to be recalibrated. We therefore emphasise that our framework is designed to evolve, while new rubrics require up-front effort to develop and validate.

The public access to the scales and the methodology could attract specialised sandbagging [197]: a malicious provider could take a model with high capabilities and use the rubric to identify instances it should answer correctly and incorrectly to create a believable error profile. Conversely, this could be used for good: limiting capabilities (rejecting or failing on questions for which the demands are higher than some thresholds) as a better approach than unlearning [198].

Validity and reliability in evaluation are of utmost importance in stake-high applications. Measuring the wrong construct or measuring it wrong leads to poor decisions, entailing ethical concerns, various risks and possible harm to people. Overestimation of abilities (and predicted performance) can contribute to hype and use of AI in contexts when it is not ready. Underestimation is problematic for regulators, especially for safety. This paper makes progress in all these fronts but it is not the definitive solution in this space. We advise a careful and critical use of our methodology, report its limitations and collectively work on its improvement.

1.19. Glossary

ability In this paper, a very precise term referring to a property of a subject (AI system or human) defined as the demand level of those items for which there is a 0.5 chance of being correct.

ability profile A vector with the specific ability levels in each of the scales for a set of dimensions.

amalgamation Agglomeration of elements in a question for the only purpose of making it harder, usually leading to under-estimation in capabilities or attainment.

attainment Positive property of a system, usually an acquired construct representing knowledge or specialised skills, that allows us to predict or explain performance.

AI system "A machine-based system that is designed to operate with varying levels of autonomy and that may exhibit adaptiveness after deployment, and that, for explicit or implicit objectives, infers, from the input it receives, how to generate outputs such as predictions, content, recommendations, or decisions that can influence physical or virtual environments" (Article 3, EU AI Act).

AI model An operative abstraction of a parcel of the world, parameterised or not, which is usually trained from data. The better the model represents the world and captures its patterns, the more it can be used to make predictions, give explanations or perform simulations about the world.

battery of tests A set of tests designed to be complementary or extensive about a certain domain or set of domains.

benchmark A set of tasks used to compare performance of several systems, models or components.

capability Positive property of a system, usually an innate construct, that allows us to predict or explain performance.

compensatory Two properties are said to compensate if not having enough of one can be compensated by having a great amount of the other.

construct A latent trait rather than an observable variable that tries to capture a property with high predictive and explanatory power. For instance, "agreeableness" is a construct that predicts and explains how people behave in human interactions.

contamination Use of data for testing that appeared during training or development, usually leading to over-estimation in capabilities or attainment. In machine learning this is a kind of over-fitting to the training data.

demand An observable property of a task that indicates one aspect of its difficulty (e.g., the number of digits in an arithmetic operation). In this paper, each demand originally leads to a dimension, but several demands can be grouped into one dimension.

dimension A scale along which the constructs affecting subject behaviour over items are carved. In other words, a feature in this space.

difficulty A generic demand of a task that is usually inversely correlated with performance.

discrimination power The quality of an item to distinguish between individuals, typically because its difficulty or demand lies between the abilities of the individuals.

evaluation A procedure to determine the value and qualities (capabilities, risks, etc.) of a system, model or component.

**foundation model**	A model of a parcel of the world that is meant to be used for downstream applications.
**funnelling**	Guiding a respondent toward the right answer by limiting or reducing the options [191], making distractors obvious or using hints or cues.
**generality**	Regularity and sharpness in the capability levels that a system exhibits across a range of domains, as opposed to having high levels in some areas but low levels in others, or flat characteristic curves in many of them.
**instance**	A particular example or instantiation of a task. In the context of LLMs, this is usually a specific prompt (e.g., "What's the capital of France?").
**item**	An instance.
**large language model (LLM)**	A model that captures the distribution of one or several languages, whether natural or artificial (e.g., English and Python), usually expressed as a stochastic model assigning probabilities to the next word or token. These probabilities can be used to generate text.
**modality**	A particular way in which inputs and outputs can be represented, such as text, audio, video, or through other sensor/actuator forms.
**model**	See AI model.
**out-of-distribution (OOD)**	Refers to data points or regions of a problem space that differ from those seen in the original (typically training) distribution.
**performance**	The observed value for a metric that measures the degree to which a goal is met on a task, dataset, or benchmark. The EU AI Act, for example, mentions "the ability of an AI system to achieve its intended purpose" – actual performance is a measurable result that shows the intended purpose is met [199].
**personality**	A property of a system (usually a construct) that helps predict or explain its behavior, though it is not necessarily monotonic or directly linked to problem solving like capability or attainment.
**populational**	Said of a metric or measurement that depends on the entire population of subjects or instances being evaluated rather than on only individuals or specific batches.
**propensity**	A property (often a construct) of a system that makes a particular behaviour more likely.
**reinforcement learning from human feedback (RLHF)**	A common mechanism—especially in large language models—by which generated content is modified to make the model more instructable, agreeable, safe, or palatable using human feedback.
**ratio scales**	The highest level in Stevens' typology [144] of measurement (which includes nominal, ordinal, interval and ratio scales), where both differences and ratios are meaningful. For example, length in metres, time in seconds, or Kelvin temperature are ratio scales; in contrast, Celsius or Fahrenheit are interval scales because they lack an absolute zero.
**reliability (of a test or evaluation)**	The degree to which a testing procedure yields stable and consistent results across multiple administrations.
**scale**	An ordering of qualitative or quantitative levels along which we can arrange demands and abilities for comparison.
**sensitivity**	The extent to which a metric or model captures all the relevant aspects of what it is supposed to measure.

**specificity**	The extent to which a metric or model captures only the relevant aspects—excluding what it should not capture.
**subject**	The system, model, or human being evaluated. (Note: In the EU AI Act, "subject" refers only to humans: "for the purpose of real-world testing, means a natural person who participates in testing in real-world conditions" (Article 3, EU AI Act).)
**subject characteristic curve**	A plot that shows performance as a function of difficulty, usually binning by ranges of difficulty or demand, and averaging performance per bin.
**superhuman performance**	Performance that exceeds the average or even the best human performance on a particular task.
**system**	See AI system.
**task**	A structured problem paired with a metric that quantifies the quality of goal completion. A task represents a quantum of work that may be delegated, abstracted from a usual situation, or conceptualised as a challenge demanding certain capabilities or skills. (For example, "addition" is a task, of which "3+5" is an instance.)
**testing**	"Assessment of the fitness of a product to achieve its stated goals" [199]. Testing is typically aimed at exposing failures in a system or component, or certifying the parts that function as intended.
**validation (of a system, model or component)**	The process of establishing that the intended use of the system, model, or component is met.
**validity (of a test)**	The degree to which a test measures what it is designed to measure.
**verification (of a system, model or component)**	The process of establishing that a system, model, or component meets its specifications.

1.20. References in Supplementary Information

[1] R. Flesch, "Marks of readable style; a study in adult education.," *Teachers College Contributions to Education*, 1943.

[2] A. C. Graesser, D. S. McNamara, and J. M. Kulikowich, "Coh-metrix: Providing multilevel analyses of text characteristics," *Educational researcher*, vol. 40, no. 5, pp. 223–234, 2011.

[3] T. François and E. Miltsakaki, "Do nlp and machine learning improve traditional readability formulas?," in *Proceedings of the First Workshop on Predicting and Improving Text Readability for target reader populations*, pp. 49–57, 2012.

[4] T. Brown, B. Mann, N. Ryder, M. Subbiah, J. D. Kaplan, P. Dhariwal, A. Neelakantan, P. Shyam, G. Sastry, A. Askell, *et al.*, "Language models are few-shot learners," *Advances in neural information processing systems*, vol. 33, pp. 1877–1901, 2020.

[5] X. He, Z. Lin, Y. Gong, A.-L. Jin, H. Zhang, C. Lin, J. Jiao, S. M. Yiu, N. Duan, and W. Chen, "Annollm: Making large language models to be better crowdsourced annotators," 2023.

[6] J. Savelka and K. D. Ashley, "The unreasonable effectiveness of large language models in zero-shot semantic annotation of legal texts," *Frontiers in Artificial Intelligence*, vol. 6, 2023.

[7] Y. Suhara, J. Li, Y. Li, D. Zhang, Ç. Demiralp, C. Chen, and W.-C. Tan, "Annotating columns with pre-trained language models," in *Proceedings of the 2022 International Conference on Management of Data*, pp. 1493–1503, 2022.

[8] N. Pangakis, S. Wolken, and N. Fasching, "Automated annotation with generative ai requires validation," *arXiv preprint arXiv:2306.00176*, 2023.

[9] K. S. McGrew, "The cattell-horn-carroll theory of cognitive abilities: Past, present, and future," in *Contemporary intellectual assessment: Theories, tests, and issues* (D. P. Flanagan and P. L. Harrison, eds.), pp. 136–181, The Guilford Press, 2 ed., 2005.

[10] J. Rust, M. Kosinski, and D. Stillwell, *Modern psychometrics: The science of psychological assessment*. 4th Edition, Routledge, 2021.

[11] J. Hernández-Orallo and K. Vold, "AI extenders: the ethical and societal implications of humans cognitively extended by AI," in *Proceedings of the 2019 AAAI/ACM Conference on AI, Ethics, and Society*, pp. 507–513, 2019.

[12] S. Tolan, A. Pesole, F. Martínez-Plumed, E. Fernández-Macías, J. Hernández-Orallo, and E. Gómez, "Measuring the occupational impact of AI: tasks, cognitive abilities and AI benchmarks," *Journal of Artificial Intelligence Research*, vol. 71, pp. 191–236, 2021.

[13] L. L. Thurstone, "Primary mental abilities: Psychometric monographs no. 1," in *The measurement of intelligence*, pp. 131–136, Springer, 1938.

[14] E. A. Wasserman and T. R. Zentall, *Comparative cognition: Experimental explorations of animal intelligence*. Oxford University Press, 2006.

[15] S. Adams, I. Arel, J. Bach, R. Coop, R. Furlan, B. Goertzel, J. S. Hall, A. Samsonovich, M. Scheutz, M. Schlesinger, *et al.*, "Mapping the landscape of human-level artificial general intelligence," *AI magazine*, vol. 33, no. 1, pp. 25–42, 2012.

[16] J. Hernández-Orallo, *The measure of all minds: evaluating natural and artificial intelligence*. Cambridge University Press, 2017.

[17] J. Edwards and G. Dall'Alba, "Development of a scale of cognitive demand for analysis of printed secondary science materials," *Research in Science Education*, vol. 11, no. 1, pp. 158–170, 1981.

[18] S. Hughes, A. Pollitt, and A. Ahmed, "The development of a tool for gauging the demands of gcse and a level exam questions," *BERA, Queen's University Belfast*, 1998.

[19] V. Crisp and N. z. d. Novaković, "Is this year's exam as demanding as last year's? using a pilot method to evaluate the consistency of examination demands over time," *Evaluation & Research in Education*, vol. 22, no. 1, pp. 3–15, 2009.

[20] A. Pollitt, A. Ahmed, and V. Crisp, "The demands of examination syllabuses and question papers," *Techniques for monitoring the comparability of examination standards*, pp. 166–206, 2007.

[21] J. K. Miller and W. Tang, "Evaluating LLM metrics through real-world capabilities," *arXiv preprint arXiv:2505.08253*, 2025.

[22] B. S. Bloom, M. D. Engelhart, E. J. Furst, W. H. Hill, and D. R. Krathwohl, *Taxonomy of educational objectives*, vol. 2. Longmans, Green New York, 1964.

[23] D. R. Krathwohl, "A revision of Bloom's taxonomy: An overview," *Theory Into Practice*, vol. 41, no. 4, pp. 212–218, 2002.

[24] I. Kroupin, H. E. Davis, E. Burdett, A. B. Cuata, V. Hartley, and J. Henrich, "The cultural construction of "executive function"," *Proceedings of the National Academy of Sciences*, vol. 122, no. 27, p. e2407955122, 2025.

[25] Y. Chang, X. Wang, J. Wang, Y. Wu, L. Yang, K. Zhu, H. Chen, X. Yi, C. Wang, Y. Wang, *et al.*, "A survey on evaluation of large language models," *ACM Transactions on Intelligent Systems and Technology*, vol. 15, no. 3, pp. 1–45, 2024.

[26] Z. Jin, Y. Chen, F. Leeb, L. Gresele, O. Kamal, Z. Lyu, K. Blin, F. Gonzalez, M. Kleiman-Weiner, M. Sachan, and B. Schölkopf, "CLadder: Assessing causal reasoning in language models," in *NeurIPS*, 2023.

[27] A. Saparov and H. He, "Language models are greedy reasoners: A systematic formal analysis of chain-of-thought," in *The Eleventh International Conference on Learning Representations*, 2023.

[28] L. M. Schulze Buschoff, E. Akata, M. Bethge, and E. Schulz, "Visual cognition in multimodal large language models," *Nature Machine Intelligence*, pp. 1–11, 2025.

[29] J.-P. Fränken, K. Gandhi, T. Qiu, A. Khawaja, N. D. Goodman, and T. Gerstenberg, "Procedural dilemma generation for evaluating moral reasoning in humans and language models," *ArXiv*, vol. abs/2404.10975, 2024.

[30] J. Coda-Forno, M. Binz, J. X. Wang, and E. Schulz, "CogBench: a large language model walks into a psychology lab," in *Forty-first International Conference on Machine Learning, ICML 2024, Vienna, Austria, July 21-27, 2024*, OpenReview.net, 2024.

[31] I. Momennejad, H. Hasanbeig, F. V. Frujeri, H. Sharma, N. Jojic, H. Palangi, R. Ness, and J. Larson, "Evaluating cognitive maps and planning in large language models with CogEval," in *Thirty-seventh Conference on Neural Information Processing Systems*, 2023.

[32] Y. Wang and Y. Zhao, "RUPBench: Benchmarking reasoning under perturbations for robustness evaluation in large language models," *ArXiv*, vol. abs/2406.11020, 2024.

[33] B. Cao, M. Ren, H. Lin, X. Han, F. Zhang, J. Zhan, and L. Sun, "StructEval: Deepen and broaden large language model assessment via structured evaluation," in *Annual Meeting of the Association for Computational Linguistics*, 2024.

[34] K. Zhu, J. Wang, Q. Zhao, R. Xu, and X. Xie, "Dynamic evaluation of large language models by meta probing agents," in *Forty-first International Conference on Machine Learning*, 2024.

[35] Y. Moros-Daval, F. Martínez-Plumed, and J. Hernández-Orallo, "Language task difficulty prediction through LLM-annotated meta-features," in *ECAI 2024*, pp. 2434–2441, IOS Press, 2024.

[36] L. Zhou, Y. Farag, and A. Vlachos, "An llm feature-based framework for dialogue constructiveness assessment," in *Proceedings of the 2024 Conference on Empirical Methods in Natural Language Processing*, pp. 5389–5409, 2024.

[37] J. Burden, M. Tešić, L. Pacchiardi, and J. Hernández-Orallo, "Paradigms of AI evaluation: Mapping goals, methodologies and culture," 2025.

[38] J. Hernandez-Orallo, "Ai evaluation: On broken yardsticks and measurement scales," in *Workshop on evaluating evaluation of AI systems at AAAI*, 2020.

[39] R. Burnell, W. Schellaert, J. Burden, T. D. Ullman, F. Martinez-Plumed, J. B. Tenenbaum, D. Rutar, L. G. Cheke, J. Sohl-Dickstein, M. Mitchell, *et al.*, "Rethink reporting of evaluation results in ai," *Science*, vol. 380, no. 6641, pp. 136–138, 2023.

[40] S. Biderman, H. Schoelkopf, L. Sutawika, L. Gao, J. Tow, B. Abbasi, A. F. Aji, P. S. Ammanamanchi, S. Black, J. Clive, A. DiPofi, J. Etxaniz, B. Fattori, J. Z. Forde, C. Foster, M. Jaiswal, W. Y. Lee, H. Li, C. Lovering, N. Muennighoff, E. Pavlick, J. Phang, A. Skowron, S. Tan, X. Tang, K. A. Wang, G. I. Winata, F. Yvon, and A. Zou, "Lessons from the trenches on reproducible evaluation of language models," *ArXiv*, vol. abs/2405.14782, 2024.

[41] K. Gorman and S. Bedrick, "We need to talk about standard splits," in *Proceedings of the 57th Annual Meeting of the Association for Computational Linguistics*, pp. 2786–2791, Association for Computational Linguistics, July 2019.

[42] A. S. Bergman, L. A. Hendricks, M. Rauh, B. Wu, W. Agnew, M. Kunesch, I. Duan, I. Gabriel, and W. Isaac, "Representation in AI evaluations," in *Proceedings of the 2023 ACM Conference on Fairness, Accountability, and Transparency*, pp. 519–533, 2023.

[43] S. Göllner and M. Tropmann-Frick, "Bridging the gap between theory and practice: Towards responsible AI evaluation.," in *CHAI@ KI*, pp. 68–76, 2023.

[44] Y. Lyu and Y. Du, "The ethical evaluation of large language models and its optimization," *AI and Ethics*, pp. 1–14, 2025.

[45] M. Levy, A. Jacoby, and Y. Goldberg, "Same task, more tokens: the impact of input length on the reasoning performance of large language models," in *Annual Meeting of the Association for Computational Linguistics*, 2024.

[46] H. Wang, H. Shi, S. Tan, W. Qin, W. Wang, T. Zhang, A. U. Nambi, T. Ganu, and H. Wang, "Multimodal needle in a haystack: Benchmarking long-context capability of multimodal large language models," *ArXiv*, vol. abs/2406.11230, 2024.

[47] J. Achiam, S. Adler, S. Agarwal, L. Ahmad, I. Akkaya, F. L. Aleman, D. Almeida, J. Altenschmidt, S. Altman, S. Anadkat, *et al.*, "Gpt-4 technical report," *arXiv preprint arXiv:2303.08774*, 2023.

[48] M. Roberts, H. Thakur, C. Herlihy, C. White, and S. Dooley, "Data contamination through the lens of time," *arXiv preprint arXiv:2310.10628*, 2023.

[49] M. Jiang, K. Liu, M. Zhong, R. Schaeffer, S. Ouyang, J. Han, and S. Koyejo, "Does data contamination make a difference? insights from intentionally contaminating pre-training data for language models," in *ICLR 2024 Workshop on Navigating and Addressing Data Problems for Foundation Models*, 2024.

[50] S. I. Mirzadeh, K. Alizadeh, H. Shahrokhi, O. Tuzel, S. Bengio, and M. Farajtabar, "Gsm-symbolic: Understanding the limitations of mathematical reasoning in large language models," in *The Thirteenth International Conference on Learning Representations*, 2024.

[51] S. Srivastava, A. PV, S. Menon, A. Sukumar, A. Philipose, S. Prince, S. Thomas, *et al.*, "Functional benchmarks for robust evaluation of reasoning performance, and the reasoning gap," *arXiv preprint arXiv:2402.19450*, 2024.

[52] R. Dominguez-Olmedo, F. E. Dorner, and M. Hardt, "Training on the test task confounds evaluation and emergence," *arXiv preprint arXiv:2407.07890*, 2024.

[53] H. Wang, S. Zhao, Z. Qiang, N. Xi, B. Qin, and T. Liu, "LLMs may perform MCQA by selecting the least incorrect option," in *Proceedings of the 31st International Conference on Computational Linguistics*, pp. 5852–5862, 2025.

[54] N. Balepur, R. Rudinger, and J. L. Boyd-Graber, "Which of these best describes multiple choice evaluation with llms? a) forced b) flawed c) fixable d) all of the above," *arXiv preprint arXiv:2502.14127*, 2025.

[55] D. Schlangen, "Language tasks and language games: On methodology in current natural language processing research," *arXiv preprint arXiv:1908.10747*, 2019.

[56] R. Zellers, A. Holtzman, Y. Bisk, A. Farhadi, and Y. Choi, "Hellaswag: Can a machine really finish your sentence?," in *Proceedings of the 57th Annual Meeting of the Association for Computational Linguistics*, pp. 4791–4800, 2019.

[57] T. R. McIntosh, T. Susnjak, T. Liu, P. Watters, and M. N. Halgamuge, "Inadequacies of large language model benchmarks in the era of generative artificial intelligence," *ArXiv*, vol. abs/2402.09880, 2024.

[58] A. Kadian, J. Truong, A. Gokaslan, A. Clegg, E. Wijmans, S. Lee, M. Savva, S. Chernova, and D. Batra, "Sim2real predictivity: Does evaluation in simulation predict real-world performance?," *IEEE Robotics and Automation Letters*, vol. 5, no. 4, pp. 6670–6677, 2020.

[59] B. C. Stahl, J. Antoniou, N. Bhalla, L. Brooks, P. Jansen, B. Lindqvist, A. Kirichenko, S. Marchal, R. Rodrigues, N. Santiago, *et al.*, "A systematic review of artificial intelligence impact assessments," *Artificial Intelligence Review*, vol. 56, no. 11, pp. 12799–12831, 2023.

[60] M. Rauh, N. Marchal, A. Manzini, L. A. Hendricks, R. Comanescu, C. Akbulut, T. Stepleton, J. Mateos-Garcia, S. Bergman, J. Kay, *et al.*, "Gaps in the safety evaluation of generative ai," in *Proceedings of the AAAI/ACM Conference on AI, Ethics, and Society*, vol. 7, pp. 1200–1217, 2024.

[61] M. Lee, M. Srivastava, A. Hardy, J. Thickstun, E. Durmus, A. Paranjape, I. Gerard-Ursin, X. L. Li, F. Ladhak, F. Rong, *et al.*, "Evaluating human-language model interaction," *Transactions on Machine Learning Research*, 2024.

[62] K. M. Collins, A. Q. Jiang, S. Frieder, L. Wong, M. Zilka, U. Bhatt, T. Lukasiewicz, Y. Wu, J. B. Tenenbaum, W. Hart, *et al.*, "Evaluating language models for mathematics through interactions," *Proceedings of the National Academy of Sciences*, vol. 121, no. 24, p. e2318124121, 2024.

[63] A. Hardy, A. Reuel, K. Jafari Meimandi, L. Soder, A. Griffith, D. M. Asmar, S. Koyejo, M. S. Bernstein, and M. J. Kochenderfer, "More than marketing? on the information value of AI benchmarks for practitioners," in *Proceedings of the 30th International Conference on Intelligent User Interfaces*, pp. 1032–1047, 2025.

[64] M. Eriksson, E. Purificato, A. Noroozian, J. Vinagre, G. Chaslot, E. Gomez, and D. Fernandez-Llorca, "Can we trust ai benchmarks? an interdisciplinary review of current issues in ai evaluation," *arXiv preprint arXiv:2502.06559*, 2025.

[65] T. Liao, R. Taori, D. Raji, and L. Schmidt, "Are we learning yet? a meta review of evaluation failures across machine learning," in *Proceedings of the Neural Information Processing Systems Track on Datasets and Benchmarks* (J. Vanschoren and S. Yeung, eds.), vol. 1, 2021.

[66] S. Bowman and G. Dahl, "What will it take to fix benchmarking in natural language understanding?," in *Proceedings of the 2021 Conference of the North American Chapter of the Association for Computational Linguistics: Human Language Technologies*, pp. 4843–4855, 2021.

[67] A. Subramonian, X. Yuan, H. Daumé III, and S. L. Blodgett, "It takes two to tango: Navigating conceptualizations of NLP tasks and measurements of performance," in *Findings of the Association for Computational Linguistics: ACL 2023* (A. Rogers, J. Boyd-Graber, and N. Okazaki, eds.), pp. 3234–3279, Association for Computational Linguistics, July 2023.

[68] H. Wainer and H. I. Braun, *Test validity*. Routledge, 2013.

[69] J. Hu and M. Frank, "Auxiliary task demands mask the capabilities of smaller language models," in *First Conference on Language Modeling*, 2024.

[70] C. Siska, K. Marazopoulou, M. Ailem, and J. Bono, "Examining the robustness of LLM evaluation to the distributional assumptions of benchmarks," in *Proceedings of the 62nd Annual Meeting of the Association for Computational Linguistics (Volume 1: Long Papers)* (L.-W. Ku, A. Martins, and V. Srikumar, eds.), pp. 10406–10421, Association for Computational Linguistics, Aug. 2024.

[71] R. Ren, S. Basart, A. Khoja, A. Gatti, L. Phan, X. Yin, M. Mazeika, A. Pan, G. Mukobi, R. H. Kim, S. Fitz, and D. Hendrycks, "Safetywashing: Do AI safety benchmarks actually measure safety progress?," in *Advances in Neural Information Processing Systems 38: Annual Conference on Neural Information Processing Systems 2024, NeurIPS 2024, Vancouver, BC, Canada, December 10 - 15, 2024* (A. Globersons, L. Mackey, D. Belgrave, A. Fan, U. Paquet, J. M. Tomczak, and C. Zhang, eds.), 2024.

[72] Y. L. Liu, S. L. Blodgett, J. Cheung, Q. V. Liao, A. Olteanu, and Z. Xiao, "ECBD: Evidence-centered benchmark design for NLP," in *Proceedings of the 62nd Annual Meeting of the Association for Computational Linguistics (Volume 1: Long Papers)* (L.-W. Ku, A. Martins, and V. Srikumar, eds.), pp. 16349–16365, Association for Computational Linguistics, Aug. 2024.

[73] A. Reuel, A. Hardy, C. Smith, M. Lamparth, M. Hardy, and M. Kochenderfer, "BetterBench: Assessing AI benchmarks, uncovering issues, and establishing best practices," in *The Thirty-eight Conference on Neural Information Processing Systems Datasets and Benchmarks Track*, 2024.

[74] European Union, "EU Artificial Intelligence Act." Regulation (EU) 2024/1689, Official Journal, June 2024. Interinstitutional File: 2021/0106(COD).

[75] J. Hernández-Orallo, "Evaluation in artificial intelligence: from task-oriented to ability-oriented measurement," *Artificial Intelligence Review*, vol. 48, pp. 397–447, 2017.

[76] J. Burden, "Evaluating AI evaluation: Perils and prospects," *arXiv preprint arXiv:2407.09221*, 2024.

[77] L. Zhou, P. A. Moreno-Casares, F. Martínez-Plumed, J. Burden, R. Burnell, L. Cheke, C. Ferri, A. Marcoci, B. Mehrbakhsh, Y. Moros-Daval, *et al.*, "Predictable artificial intelligence," *arXiv preprint arXiv:2310.06167*, 2023.

[78] J. Burden, K. Voudouris, R. Burnell, D. Rutar, L. Cheke, and J. Hernández-Orallo, "Inferring capabilities from task performance with Bayesian triangulation," *arXiv preprint arXiv:2309.11975*, 2023.

[79] A. Srivastava, A. Rastogi, A. Rao, A. A. M. Shoeb, A. Abid, A. Fisch, A. R. Brown, A. Santoro, A. Gupta, A. Garriga-Alonso, *et al.*, "Beyond the imitation game: Quantifying and extrapolating the capabilities of language models," *Journal of Machine Learning Research*, 2023.

[80] V. Balachandran, J. Chen, N. Joshi, B. Nushi, H. Palangi, E. Salinas, V. Vineet, J. Woffinden-Luey, and S. Yousefi, "Eureka: Evaluating and understanding large foundation models," *arXiv preprint arXiv:2409.10566*, 2024.

[81] Z. Fountas, M. Benfeghoul, A. Oomerjee, F. Christopoulou, G. Lampouras, H. B. Ammar, and J. Wang, "Human-inspired episodic memory for infinite context LLMs," in *The Thirteenth International Conference on Learning Representations*, 2025.

[82] A. Masry, J. A. Rodriguez, T. Zhang, S. Wang, C. Wang, A. Feizi, A. K. Suresh, A. Puri, X. Jian, P.-A. Noël, *et al.*, "Alignvlm: Bridging vision and language latent spaces for multimodal understanding," *arXiv preprint arXiv:2502.01341*, 2025.

[83] J. Hernández-Orallo, B. S. Loe, L. Cheke, F. Martínez-Plumed, and S. Ó hÉigeartaigh, "General intelligence disentangled via a generality metric for natural and artificial intelligence," *Scientific reports*, vol. 11, no. 1, p. 22822, 2021.

[84] J. Hernandez-Orallo, "Caveats and solutions for characterising general-purpose AI," in *ECAI 2024*, pp. 2–9, IOS Press, 2024.

[85] R. Burnell, H. Hao, A. R. Conway, and J. H. Orallo, "Revealing the structure of language model capabilities," *arXiv preprint arXiv:2306.10062*, 2023.

[86] D. Ilić and G. E. Gignac, "Evidence of interrelated cognitive-like capabilities in large language models: Indications of artificial general intelligence or achievement?," *Intelligence*, vol. 106, p. 101858, 2024.

[87] Y. Ruan, C. J. Maddison, and T. Hashimoto, "Observational scaling laws and the predictability of langauge model performance," in *The Thirty-eighth Annual Conference on Neural Information Processing Systems*, 2024.

[88] L. Kuhn, Y. Gal, and S. Farquhar, "Semantic uncertainty: Linguistic invariances for uncertainty estimation in natural language generation," in *NeurIPS ML Safety Workshop*.

[89] J. Duan, H. Cheng, S. Wang, A. Zavalny, C. Wang, R. Xu, B. Kailkhura, and K. Xu, "Shifting attention to relevance: Towards the predictive uncertainty quantification of free-form large language models," in *Proceedings of the 62nd Annual Meeting of the Association for Computational Linguistics (Volume 1: Long Papers)*, pp. 5050–5063, 2024.

[90] R. Cohen, K. Dobler, E. Biran, and G. de Melo, "I don't know: Explicit modeling of uncertainty with an [idk] token," *Advances in Neural Information Processing Systems*, vol. 37, pp. 10935–10958, 2024.

[91] S. Kadavath, T. Conerly, A. Askell, T. Henighan, D. Drain, E. Perez, N. Schiefer, Z. Hatfield-Dodds, N. DasSarma, E. Tran-Johnson, *et al.*, "Language models (mostly) know what they know," *CoRR*, 2022.

[92] M. Xiong, Z. Hu, X. Lu, Y. LI, J. Fu, J. He, and B. Hooi, "Can llms express their uncertainty? an empirical evaluation of confidence elicitation in llms," in *The Twelfth International Conference on Learning Representations*.

[93] V. Shrivastava, P. Liang, and A. Kumar, "Llamas know what gpts don't show: Surrogate models for confidence estimation," *arXiv preprint arXiv:2311.08877*, 2023.

[94] J. Hernández-Orallo, W. Schellaert, and F. Martínez-Plumed, "Training on the test set: Mapping the system-problem space in ai," in *Proceedings of the AAAI Conference on Artificial Intelligence*, pp. 12256–12261, 2022.

[95] L. Zhou, F. Martínez-Plumed, J. Hernández-Orallo, C. Ferri, and W. Schellaert, "Reject before you run: Small assessors anticipate big language models," *Proceedings of the Workshop on AI Evaluation Beyond Metrics co-located with the 31st International Joint Conference on Artificial Intelligence (IJCAI-ECAI 2022)*, 2022.

[96] W. Schellaert, F. Martínez-Plumed, and J. Hernández-Orallo, "Analysing the predictability of language model performance," *ACM Transactions on Intelligent Systems and Technology*, vol. 16, no. 2, pp. 1–26, 2025.

[97] P. Drapal, R. B. Prudêncio, and T. M. S. Filho, "Towards explainable evaluation: Explaining predicted performance using local performance regions," *Applied Soft Computing*, vol. 167, p. 112351, 2024.

[98] L. Pacchiardi, L. G. Cheke, and J. Hernández-Orallo, “100 instances is all you need: predicting the success of a new LLM on unseen data by testing on a few instances,” 2024.

[99] Z. Ye, P. Liu, J. Fu, and G. Neubig, “Towards more fine-grained and reliable nlp performance prediction,” *arXiv preprint arXiv:2102.05486*, 2021.

[100] A. Srinivasan, S. Sitaram, T. Ganu, S. Dandapat, K. Bali, and M. Choudhury, “Predicting the performance of multilingual nlp models,” *arXiv preprint arXiv:2110.08875*, 2021.

[101] K. Ahuja, S. Dandapat, S. Sitaram, and M. Choudhury, “Beyond static models and test sets: Benchmarking the potential of pre-trained models across tasks and languages,” *arXiv preprint arXiv:2205.06356*, 2022.

[102] P. Drapal, T. Silva-Filho, and R. B. C. Prudêncio, “Meta-Learning and Novelty Detection for Machine Learning with Reject Option,” in *2024 International Joint Conference on Neural Networks (IJCNN)*, pp. 1–8, 2024.

[103] K. Hendrickx, L. Perini, D. Van der Plas, W. Meert, and J. Davis, “Machine learning with a reject option: A survey,” *Machine Learning*, vol. 113, no. 5, pp. 3073–3110, 2024.

[104] O. Shorinwa, Z. Mei, J. Lidard, A. Z. Ren, and A. Majumdar, “A survey on uncertainty quantification of large language models: Taxonomy, open research challenges, and future directions,” *arXiv preprint arXiv:2412.05563*, 2024.

[105] S. E. Embretson and S. P. Reise, *Item response theory*. Psychology Press, 2013.

[106] F. M. Polo, L. Weber, L. Choshen, Y. Sun, G. Xu, and M. Yurochkin, “tinybenchmarks: evaluating LLMs with fewer examples,” in *ICLR 2024 Workshop on Mathematical and Empirical Understanding of Foundation Models*, 2024.

[107] L. Pacchiardi, K. Voudouris, B. Slater, F. Martínez-Plumed, J. Hernández-Orallo, L. Zhou, and W. Schellaert, “PredictaBoard: Benchmarking LLM score predictability,” 2025.

[108] N. Carlini, “A GPT-4 capability forecasting challenge.” `https://nicholas.carlini.com/writing/llm-forecast/question/Capital-of-Paris`, 2024. Accessed: 2024-09-08.

[109] K. Vafa, A. Rambachan, and S. Mullainathan, “Do large language models perform the way people expect? measuring the human generalization function,” in *Forty-first International Conference on Machine Learning*, 2024.

[110] L. Zhou, W. Schellaert, F. Martínez-Plumed, Y. Moros-Daval, C. Ferri, and J. Hernández-Orallo, “Larger and more instructable language models become less reliable,” *Nature*, vol. 634, no. 8032, pp. 61–68, 2024.

[111] M. Steyvers, H. Tejeda, A. Kumar, C. Belem, S. Karny, X. Hu, L. W. Mayer, and P. Smyth, “What large language models know and what people think they know,” *Nature Machine Intelligence*, pp. 1–11, 2025.

[112] R. D. Bock and R. D. Gibbons, *Item response theory*. John Wiley & Sons, 2021.

[113] C. D. Vale and D. J. Weiss, “A study of computer-administered stradaptive ability testing.,” tech. rep., Minnesota Univ. Minneapolis Dept. of Psychology, 1975.

[114] T. E. Trabin and D. J. Weiss, “The person response curve: Fit of individuals to item response theory models,” in *New horizons in testing*, pp. 83–108, Elsevier, 1983.

[115] F. Martínez-Plumed, R. B. Prudêncio, A. Martínez-Usó, and J. Hernández-Orallo, “Item response theory in ai: Analysing machine learning classifiers at the instance level,” *Artificial intelligence*, vol. 271, pp. 18–42, 2019.

[116] J. P. Lalor, H. Wu, and H. Yu, “Building an evaluation scale using item response theory,” in *Proceedings of the Conference on Empirical Methods in Natural Language Processing. Conference on Empirical Methods in Natural Language Processing*, vol. 2016, p. 648, NIH Public Access, 2016.

[117] A. Kipnis, K. Voudouris, L. M. S. Buschoff, and E. Schulz, “metabench–a sparse benchmark to measure general ability in large language models,” *arXiv preprint arXiv:2407.12844*, 2024.

[118] C. Vania, P. M. Htut, W. Huang, D. Mungra, R. Y. Pang, J. Phang, H. Liu, K. Cho, and S. R. Bowman, “Comparing test sets with item response theory,” in *Proceedings of the 59th Annual Meeting of the Association for Computational Linguistics and the 11th International Joint Conference on Natural Language Processing (Volume 1: Long Papers)* (C. Zong, F. Xia, W. Li, and R. Navigli, eds.), pp. 1141–1158, Association for Computational Linguistics, Aug. 2021.

[119] J. P. Lalor, P. Rodriguez, J. Sedoc, and J. Hernandez-Orallo, “Item response theory for natural language processing,” in *Proceedings of the 18th Conference of the European Chapter of the Association for Computational Linguistics: Tutorial Abstracts*, pp. 9–13, 2024.

[120] Q. Fang, D. L. Oberski, and D. Nguyen, “PATCH - psychometrics-assisted benchmarking of large language models: A case study of mathematics proficiency,” *ArXiv*, vol. abs/2404.01799, 2024.

[121] Y. Zhuang, Q. Liu, Y. Ning, W. Huang, Z. A. Pardos, P. C. Kyllonen, J. Zu, Q. Mao, R. Lv, Z. Huang, G. Zhao, Z. Zhang, S. Wang, and E. Chen, “From static benchmarks to adaptive testing: Psychometrics in AI evaluation,” 2024.

[122] F. Tang, W. Gao, L. Peng, and J. Zhan, “Agibench: A multi-granularity, multimodal, human-referenced, auto-scoring benchmark for large language models,” *ArXiv*, vol. abs/2309.06495, 2023.

[123] Z. Lei, T. Liang, H. Hu, J. Zhang, Y. Zhou, Y. Shao, L. Li, C. Li, C. Wang, H. Yan, and Q. Guo, “GAOKAO-eval: Does high scores truly reflect strong capabilities in LLMs?,” *ArXiv*, vol. abs/2412.10056, 2024.

[124] D. Federiakin, “Improving LLM leaderboards with psychometrical methodology,” *arXiv preprint arXiv:2501.17200*, 2025.

[125] M. Ding, C. Deng, J. Choo, Z. Wu, A. Agrawal, A. Schwarzschild, T. Zhou, T. Goldstein, J. Langford, A. Anandkumar, *et al.*, “Easy2hard-bench: Standardized difficulty labels for profiling llm performance and generalization,” *Advances in Neural Information Processing Systems*, vol. 37, pp. 44323–44365, 2024.

[126] M. D. Reckase, “Chapter 18 - multidimensional item response theory,” *Handbook of statistics*, vol. 26, pp. 607–642, 2006.

[127] W. Bonifay, *Multidimensional item response theory*. Sage Publications, 2019.

[128] T. A. Ackerman, “Unidimensional irt calibration of compensatory and noncompensatory multidimensional items,” *Applied Psychological Measurement*, vol. 13, no. 2, pp. 113–127, 1989.

[129] Q. Liu, Z. Gong, Z. Huang, C. Liu, H. Zhu, Z. Li, E. Chen, and H. Xiong, “Multi-dimensional ability diagnosis for machine learning algorithms,” *Science China Information Sciences*, vol. 67, no. 12, pp. 1–2, 2024.

[130] G. H. Fischer, “The linear logistic test model as an instrument in educational research,” *Acta psychologica*, vol. 37, no. 6, pp. 359–374, 1973.

[131] P. De Boeck, *Explanatory item response models: A generalized linear and nonlinear approach*. Springer Science & Business Media, 2004.

[132] P. De Boeck and M. Wilson, “Multidimensional explanatory item response modeling,” in *Handbook of item response theory modeling*, pp. 252–271, Routledge, 2014.

[133] M. von Davier and K. Yamamoto, “A class of models for cognitive diagnosis,” in *4th spearman conference, Philadelphia, PA*, 2004.

[134] M. Von Davier, “A general diagnostic model applied to language testing data,” *British Journal of Mathematical and Statistical Psychology*, vol. 61, no. 2, pp. 287–307, 2008.

[135] L. V. DiBello, R. A. Henson, and W. F. Stout, “A family of generalized diagnostic classification models for multiple choice option-based scoring,” *Applied psychological measurement*, vol. 39, no. 1, pp. 62–79, 2015.

[136] F. Wang, W. Gao, Q. Liu, J. Li, G. Zhao, Z. Zhang, Z. Huang, M. Zhu, S. Wang, W. Tong, *et al.*, “A survey of models for cognitive diagnosis: New developments and future directions,” *arXiv preprint arXiv:2407.05458*, 2024.

[137] F. F. Chen and Z. Zhang, “Bifactor models in psychometric test development,” *The Wiley handbook of psychometric testing: A multidisciplinary reference on survey, scale and test development*, pp. 325–345, 2018.

[138] X. Wang, L. Jiang, J. Hernandez-Orallo, D. Stillwell, L. Sun, F. Luo, and X. Xie, “Evaluating general-purpose AI with psychometrics,” *arXiv preprint arXiv:2310.16379*, 2023.

[139] B. D. Stucky and M. O. Edelen, “Using hierarchical irt models to create unidimensional measures from multidimensional data,” *Handbook of item response theory modeling*, pp. 183–206, 2014.

[140] D. Thissen and H. Wainer, “Test scoring,” 2002.

[141] G. Guinet, B. Omidvar-Tehrani, A. Deoras, and L. Callot, “Automated evaluation of retrieval-augmented language models with task-specific exam generation,” in *Proceedings of the 41st International Conference on Machine Learning*, pp. 16773–16801, 2024.

[142] J. Zhang, W. Huang, Z. Ma, O. Michel, D. He, T. Gupta, W.-C. Ma, A. Farhadi, A. Kembhavi, and R. Krishna, “Task me anything,” *Conference on Neural Information Processing Systems*, 2025.

[143] D. J. Hand, *Measurement: A very short introduction*. Oxford University Press, 2016.

[144] S. S. Stevens, “On the theory of scales of measurement,” *Science*, vol. 103, no. 2684, pp. 677–680, 1946.

[145] J. Michell, *Measurement in psychology: A critical history of a methodological concept*, vol. 53. Cambridge University Press, 1999.

[146] F. M. Lord, “The ‘ability’scale in item characteristic curve theory,” *Psychometrika*, vol. 40, no. 2, pp. 205–217, 1975.

[147] R. Freund, *Rasch and rationality: Scale typologies as applied to item response theory*. PhD thesis, UC Berkeley, 2019.

[148] J. Wallmark, M. Josefsson, and M. Wiberg, “Introducing flexible monotone multiple choice item response theory models and bit scales,” *arXiv preprint arXiv:2410.01480*, 2024.

[149] M. Kazemi, B. Fatemi, H. Bansal, J. Palowitch, C. Anastasiou, S. V. Mehta, L. K. Jain, V. Aglietti, D. Jindal, P. Chen, *et al.*, “Big-bench extra hard,” *arXiv preprint arXiv:2502.19187*, 2025.

[150] OECD, “Education at a glance 2024: Oecd indicators,” *OECD Publishing*, 2024.

[151] M. R. Morris, J. Sohl-Dickstein, N. Fiedel, T. Warkentin, A. Dafoe, A. Faust, C. Farabet, and S. Legg, “Levels of agi for operationalizing progress on the path to agi,” *arXiv preprint arXiv:2311.02462*, 2023.

[152] S. Embretson and S. Reise, *Item response theory for psychologists*. Mahwah, NJ: Erlbaum, 2000.

[153] W. J. Van der Linden and W. van der Linden, *Handbook of item response theory*, vol. 1. CRC press New York, 2016.

[154] F. B. Baker, *The basics of item response theory*. ERIC, 2001.

[155] J. Lumsden, “Person reliability,” *Applied Psychological Measurement*, vol. 1, no. 4, pp. 477–482, 1977.

[156] P. J. Ferrando, “A general approach for assessing person fit and person reliability in typical-response measurement,” *Applied Psychological Measurement*, vol. 38, no. 2, pp. 166–183, 2014.

[157] R. J. De Ayala, *Theory and practice of item response theory*. Guilford Publications, 2009.

[158] L. L. Thurstone, "Ability, motivation, and speed," *Psychometrika*, vol. 2, no. 4, pp. 249–254, 1937.

[159] E. Hüllermeier and W. Waegeman, "Aleatoric and epistemic uncertainty in machine learning: An introduction to concepts and methods," *Machine learning*, vol. 110, no. 3, pp. 457–506, 2021.

[160] M. Besta, J. Barth, E. Schreiber, A. Kubicek, A. Catarino, R. Gerstenberger, P. Nyczyk, P. Iff, Y. Li, S. Houliston, *et al.*, "Reasoning language models: A blueprint," *arXiv preprint arXiv:2501.11223*, 2025.

[161] I. Petrov, J. Dekoninck, L. Baltadzhiev, M. Drencheva, K. Minchev, M. Balunović, N. Jovanović, and M. Vechev, "Proof or bluff? evaluating llms on 2025 usa math olympiad," *arXiv preprint arXiv:2503.21934*, 2025.

[162] H. Sun, Y. Min, Z. Chen, W. X. Zhao, L. Fang, Z. Liu, Z. Wang, and J.-R. Wen, "Challenging the boundaries of reasoning: An olympiad-level math benchmark for large language models," *arXiv preprint arXiv:2503.21380*, 2025.

[163] Z. Chen, C. Qin, and Y. Shu, "Rimo: An easy-to-evaluate, hard-to-solve olympiad benchmark for advanced mathematical reasoning," *arXiv preprint arXiv:2509.07711*, 2025.

[164] D. Hendrycks, S. Basart, S. Kadavath, M. Mazeika, A. Arora, E. Guo, C. Burns, S. Puranik, H. He, D. Song, and J. Steinhardt, "Measuring coding challenge competence with APPS," 2021.

[165] Z. Lin, T. Liang, J. Xu, Q. Lin, X. Wang, R. Luo, C. Shi, S. Li, Y. Yang, and Z. Tu, "Critical tokens matter: Token-level contrastive estimation enhances llm's reasoning capability," *arXiv preprint arXiv:2411.19943*, 2024.

[166] Z. Hou, X. Lv, R. Lu, J. Zhang, Y. Li, Z. Yao, J. Li, J. Tang, and Y. Dong, "Advancing language model reasoning through reinforcement learning and inference scaling," *arXiv preprint arXiv:2501.11651*, 2025.

[167] X. Ma, Q. Liu, D. Jiang, G. Zhang, Z. Ma, and W. Chen, "General-reasoner: Advancing llm reasoning across all domains," *arXiv preprint arXiv:2505.14652*, 2025.

[168] K. Cobbe, V. Kosaraju, M. Bavarian, M. Chen, H. Jun, L. Kaiser, M. Plappert, J. Tworek, J. Hilton, R. Nakano, *et al.*, "Training verifiers to solve math word problems," *arXiv preprint arXiv:2110.14168*, 2021.

[169] J. Wei, X. Wang, D. Schuurmans, M. Bosma, F. Xia, E. Chi, Q. V. Le, D. Zhou, *et al.*, "Chain-of-thought prompting elicits reasoning in large language models," *Advances in neural information processing systems*, vol. 35, pp. 24824–24837, 2022.

[170] T. Kojima, S. S. Gu, M. Reid, Y. Matsuo, and Y. Iwasawa, "Large language models are zero-shot reasoners," *Advances in neural information processing systems*, vol. 35, pp. 22199–22213, 2022.

[171] A. Gladstone, G. Nanduru, M. M. Islam, P. Han, H. Ha, A. Chadha, Y. Du, H. Ji, J. Li, and T. Iqbal, "Energy-based transformers are scalable learners and thinkers," *arXiv preprint arXiv:2507.02092*, 2025.

[172] D. Rein, B. L. Hou, A. C. Stickland, J. Petty, R. Y. Pang, J. Dirani, J. Michael, and S. R. Bowman, "GPQA: A graduate-level google-proof q&a benchmark," *arXiv preprint arXiv:2311.12022*, 2023.

[173] X. Tian, S. Zhao, H. Wang, S. Chen, Y. Peng, Y. Ji, H. Zhao, and X. Li, "Deepdistill: Enhancing llm reasoning capabilities via large-scale difficulty-graded data training," *arXiv preprint arXiv:2504.17565*, 2025.

[174] A. Dubey, A. Jauhri, A. Pandey, A. Kadian, A. Al-Dahle, A. Letman, A. Mathur, A. Schelten, A. Yang, A. Fan, *et al.*, "The llama 3 herd of models," *arXiv preprint arXiv:2407.21783*, 2024.

[175] A. Zeng, X. Lv, Q. Zheng, Z. Hou, B. Chen, C. Xie, C. Wang, D. Yin, H. Zeng, J. Zhang, *et al.*, "Glm-4.5: Agentic, reasoning, and coding (arc) foundation models," *arXiv preprint arXiv:2508.06471*, 2025.

[176] Anthropic, "Claude Sonnet 4.5 system card." https://www.anthropic.com/claude-sonnet-4-5-system-card, Oct. 2025. Safety evaluations and additional technical details.

[177] X. Du, Y. Yao, K. Ma, B. Wang, T. Zheng, K. Zhu, M. Liu, Y. Liang, X. Jin, Z. Wei, *et al.*, "Supergpqa: Scaling llm evaluation across 285 graduate disciplines," *arXiv preprint arXiv:2502.14739*, 2025.

[178] ByteDance Seed Team, "Seed-oss-36b-instruct — model card." https://huggingface.co/ByteDance-Seed/Seed-OSS-36B-Instruct, 2025. Evaluation section includes SuperGPQA scores.

[179] "Qwen3 235b a22b thinking 2507 — api, providers, stats." https://openrouter.ai/qwen/qwen3-235b-a22b-thinking-2507, 2025. Lists SuperGPQA among core reasoning benchmarks.

[180] L. Xiaomi, B. Xia, B. Shen, D. Zhu, D. Zhang, G. Wang, H. Zhang, H. Liu, J. Xiao, J. Dong, *et al.*, "Mimo: Unlocking the reasoning potential of language model–from pretraining to posttraining," *arXiv preprint arXiv:2505.07608*, 2025.

[181] A. Patel, S. Bhattamishra, and N. Goyal, "Are nlp models really able to solve simple math word problems?," *arXiv preprint arXiv:2103.07191*, 2021.

[182] M. Xu, Y. Li, K. Sun, and T. Qian, "Adaption-of-thought: Learning question difficulty improves large language models for reasoning," in *Proceedings of the 2024 Conference on Empirical Methods in Natural Language Processing*, (Miami, USA), pp. 5468–5495, Association for Computational Linguistics, 2024.

[183] Z. Xi, W. Chen, B. Hong, S. Jin, R. Zheng, W. He, Y. Ding, S. Liu, X. Guo, J. Wang, H. Guo, W. Shen, X. Fan, Y. Zhou, S. Dou, X. Wang, X. Zhang, P. Sun, T. Gui, Q. Zhang, and X. Huang, "Training large language models for reasoning through reverse curriculum reinforcement learning," 2024.

[184] f. n. n. s. i. P. s. Yang and colleagues, "Enhancing llm complex reasoning capability through hyperbolic geometry," 2024. OpenReview preprint; evaluates on MAWPS, SVAMP, GSM8K, AQuA.

[185] Y. Zhang, X. Wang, L. Wu, and J. Wang, "Enhancing chain of thought prompting in large language models via reasoning patterns," in *Proceedings of the AAAI Conference on Artificial Intelligence*, AAAI Press, 2025.

[186] P. Shojaee, I. Mirzadeh, K. Alizadeh, M. Horton, S. Bengio, and M. Farajtabar, "The illusion of thinking: Understanding the strengths and limitations of reasoning models via the lens of problem complexity," *arXiv preprint arXiv:2506.06941*, 2025.

[187] Y. Sun, S. Hu, G. Zhou, K. Zheng, H. Hajishirzi, N. Dziri, and D. Song, "Omega: Can llms reason outside the box in math? evaluating exploratory, compositional, and transformative generalization," *arXiv preprint arXiv:2506.18880*, 2025.

[188] W. Zhong, R. Cui, Y. Guo, Y. Liang, S. Lu, Y. Wang, A. Saied, W. Chen, and N. Duan, "Agieval: A human-centric benchmark for evaluating foundation models," in *Findings of the Association for Computational Linguistics: NAACL 2024*, pp. 2299–2314, 2024.

[189] T. Guo, B. Nan, Z. Liang, Z. Guo, N. Chawla, O. Wiest, X. Zhang, *et al.*, "What can large language models do in chemistry? a comprehensive benchmark on eight tasks," *Advances in Neural Information Processing Systems*, vol. 36, pp. 59662–59688, 2023.

[190] C. White, S. Dooley, M. Roberts, A. Pal, B. Feuer, S. Jain, R. Shwartz-Ziv, N. Jain, K. Saifullah, S. Naidu, *et al.*, "Livebench: A challenging, contamination-free llm benchmark," *arXiv preprint arXiv:2406.19314*, 2024.

[191] Y. Wang, X. Ma, G. Zhang, Y. Ni, A. Chandra, S. Guo, W. Ren, A. Arulraj, X. He, Z. Jiang, *et al.*, "MMLU-PRO: A more robust and challenging multi-task language understanding benchmark," *Advances in Neural Information Processing Systems*, vol. 37, pp. 95266–95290, 2025.

[192] N. Khandekar, Q. Jin, G. Xiong, S. Dunn, S. S. Applebaum, Z. Anwar, M. Sarfo-Gyamfi, C. W. Safranek, A. A. Anwar, A. Zhang, *et al.*, "Medcalc-bench: Evaluating large language models for medical calculations," *arXiv preprint arXiv:2406.12036*, 2024.

[193] B. Gao, F. Song, Z. Yang, Z. Cai, Y. Miao, Q. Dong, L. Li, C. Ma, L. Chen, R. Xu, *et al.*, "Omni-math: A universal olympiad level mathematic benchmark for large language models," *arXiv preprint arXiv:2410.07985*, 2024.

[194] X. Wang, Z. Hu, P. Lu, Y. Zhu, J. Zhang, S. Subramaniam, A. R. Loomba, S. Zhang, Y. Sun, and W. Wang, "Scibench: Evaluating college-level scientific problem-solving abilities of large language models," *arXiv preprint arXiv:2307.10635*, 2023.

[195] Z. Chu, J. Chen, Q. Chen, W. Yu, H. Wang, M. Liu, and B. Qin, "Timebench: A comprehensive evaluation of temporal reasoning abilities in large language models," *arXiv preprint arXiv:2311.17667*, 2023.

[196] P. Mondorf and B. Plank, "Liar, liar, logical mire: A benchmark for suppositional reasoning in large language models," *arXiv preprint arXiv:2406.12546*, 2024.

[197] T. van der Weij, F. Hofstätter, O. Jaffe, S. F. Brown, and F. R. Ward, "Ai sandbagging: Language models can strategically underperform on evaluations," *arXiv preprint arXiv:2406.07358*, 2024.

[198] F. Barez, T. Fu, A. Prabhu, S. Casper, A. Sanyal, A. Bibi, A. O'Gara, R. Kirk, B. Bucknall, T. Fist, *et al.*, "Open problems in machine unlearning for ai safety," *arXiv preprint arXiv:2501.04952*, 2025.

[199] M. Estévez Almenzar, D. Fernández Llorca, E. Gómez, and F. Martinez Plumed, "Glossary of human-centric artificial intelligence," *Sevilla: Joint Research Centre (Seville Site)*, 2022.

2. DeLeAn Rubric Set v.1.0

In our framework, we comprehensively characterise the cognitive demands of a task in a way that is both interpretable and predictive. Our annotation scheme, which we call the `DeLeAn` Rubric Set, is organised into several groups. First, the *Elemental* dimensions, which include `Attention and Scan`, `Comprehension and Expression`, `Conceptualisation`, `Learning and Abstraction`, `Metacognition and Critical Thinking`, `Mind Modelling and Social Cognition`, `Quantitative and Logical Reasoning`, and `Spatial Reasoning and Navigation`, are adapted from the work of Tolan et al. [12] so that each dimension reflects a core human cognitive ability. Next, our *Knowledge* dimensions are based on typical levels of education—from primary to postgraduate—to measure the depth of domain-specific knowledge required by a task. Finally, the three *Extraneous* dimensions (`Volume`, `Atypicality`, and `Unguessability`) are included to capture aspects that affect item difficulty but are not intrinsic components of cognition. For example, volume captures the time required to complete an item; atypicality measures how unique or memorable an item is; and unguessability quantifies the inherent chance of guessing the answer.

The following subsections describe each rubric in detail, along with a brief explanation of its motivation and intended role.

2.1. Elemental

These rubrics are largely derived from an combination of human cognitive psychology, animal cognition and AI domains taxonomies. They follow the framework proposed by Tolan et al. [12]. They capture general cognitive capabilities that are essential for any intelligent system. We call these dimensions “elemental”, as they come from the original framework, and to distinguish them from the knowledge dimensions.

The following subsections describe each *elemental* capability scale.

Attention and Scan (AS)

This criterion assesses the level of attention and scan required to focus on or locate specific elements within a given stream of information or environment in the whole process of solving a task. During this process, there is the need to actively scan for or retrieve elements that meet predetermined criteria. The level represents the extent to which the task requires locating and focusing on specific target information, ranging from situations where the target is immediately obvious to those requiring sustained tracking of multiple targets among numerous distractors—any elements that are irrelevant to solve the task, such as visual objects, sounds, pieces of text, noise, or other stimuli, but compete for attention with the target information—in complex, dynamic environments. The challenge is not on determining what to look for but focusing the attention to find it within a larger context. This differs from tasks where there's a need to identify which pieces of information are relevant from a set already under consideration. While both processes may overlap in complex tasks like reading comprehension or image understanding, "attention and scan" specifically focuses on the deployment of attention during scan processes when solving the task, rather than the selection or evaluation of information.

Levels

Level 0 **None.** No attention or scan is required. The target information is immediately obvious or is the only information present. **Examples**:

- "Given a single word input, determine if it starts with a capital letter."
- "Look at the only object in the centre of the white page and tell what colour it is."
- "Is Madrid the capital of Spain?"

Level 1 **Very low.** Minimal attention or scanning is required. The target information is easily distinguishable with little to almost no distraction. **Examples**:

- "Find the only blue car in a car park full of red cars."
- "Find the letter 'X' among a row of 'O's"
- "Spot the tall tree in a row of short bushes."

Level 2 **Low.** Some attention or basic scanning is required. The target information is visible among a few distractors or in a small scan area. **Examples**:

- "Find all the vowels in the following sentence: 'The quick brown fox jumps over the lazy dog.'"
- "Find who's wearing glasses in this photo of students at commencement, with 2 rows of 5 students each, all facing forward, taken by a professional photographer."
- "Who authored the Queensberry rules, which were published in 1867 for the sport of boxing? Choices: A. John Douglas (in his late twenties)
  B. John Graham Chambers (in his mid-twenties)
  C. Marquess of Queensberry (in his early thirties)
  D. James Figg (in his forties)."

Level 3 **Intermediate.** Moderate attention and scan are required. The target information is mixed with several distractors or spread over a fairly large scan area. **Examples:**

- "Find everyone wearing glasses in this casual BBQ photo where 15 people are gathered around a table. Some are sitting, some standing, some looking at the camera while others are in conversation."
- "In a 5-page technical document about basic geometry, locate all explicit references to the Pythagorean theorem ($a^2 + b^2 = c^2$), where the equation appears 5 times mixed among references to 15 other geometric formulas, with occasional inconsistent equation numbering but standard mathematical notation.
- "As we all know, the Queensberry Rules are a set of rules for boxing that govern both amateur and professional matches. Who authored the Queensberry rules, which were published in 1867 for the sport of boxing? Choices: A. John Douglas (in his late twenties)
  B. John Graham Chambers (in his mid-twenties)
  C. Marquess of Queensberry (in his early thirties)
  D. James Figg (in his forties)
  E. James Zou (in his fifties)
  F. Lucy Grande (in her late twenties)
  G. Xiaoxiao Li (in her early forties)
  H. Enrique Garcia (in his late thirties)."

Level 4 **High.** Sustained tracking of one or various targets is required. The target information is in an environment mixed with numerous distractors and changing conditions. requires some continuous monitoring amid competing signals. **Examples**:

- "Listening to a symphony, identify all instances where the clarinet plays in a minor key, even when it's not playing the main melody.
- "Track three orange spheres among twenty red spheres as they move randomly across a black screen (40 cm × 30 cm) at varying speeds (1-3 cm/s), with spheres frequently intersecting paths and maintaining a minimum separation distance of 2 cm. Each sphere is 1 cm in diameter."
- "In a real-time video feed of a busy airport, finding the locations of ten blue suitcases."

Level 5+ **Very High.** Requires sustained attention and scan for simultaneous tracking of multiple targets across different domains or contexts, with continuous adaptation to fast-changing conditions. The target information is extremely difficult to distinguish from distractors or is hidden in a vast or constantly changing environment. **Examples**:

- "While seated courtside at a professional basketball game, track two specific players throughout the entire game as they move at speeds up to 8m/s, frequently cluster with other players during rebounds, and weave through screens and defensive formations."
- "Monitor four simultaneous video feeds of a crowded airport terminal from different angles, detecting subtle security-relevant changes (e.g. brief interactions < 2 seconds, crowd flow changes, small object exchanges) across feeds."
- "While monitoring multiple simultaneous customer service chat conversations in different languages, identify instances where customers are expressing the same underlying technical issue, even though they're describing it using different metaphors, technical terms, or cultural references specific to their region."

Comprehension and Expression (CE)

R1. Verbal Comprehension (CEc)

This rubric evaluates the difficulty of a task's comprehension requirements, encompassing the understanding of text, stories or the semantic content of other representations of ideas in different formats or modalities. It may include the interpretation of explicit and implicit meanings, recognition of relationships between concepts, processing of contextual information, and understanding of abstract ideas and complex systems. Noteworthy, the mere presence of specialized terminology or jargon does not necessarily indicate a high difficulty level in this rubric, as these terms may appear within tasks that follow simple, straightforward linguistic structures and are more reflective of domain knowledge rather than comprehension complexity. Further, for specialized formal languages (e.g., molecular structures, programming code) the task will be hard to comprehend only if the sequence in that formal language (e.g. molecular expression, snippet of code) is convoluted, but simple molecules or pieces of code should be easy. The rubric include difficult levels that range from tasks requiring no semantic comprehension to those demanding an understanding of highly convoluted, interconnected concepts, including the ability to process sophisticated theoretical frameworks, understand nuanced implications, and synthesize multiple complex perspectives across different domains and levels of abstraction.

Levels

Level 0 **None.** Tasks at this level require no comprehension of language or semantic content, such as those that can be completed by non-human animals. **Examples**:

- Pulling levers in a specific sequence (pull middle lever, then right lever, then left lever) to release food from a container, learning the pattern through trial and error.
- Manipulating a twist-lid container with multiple appendages in a rotating motion until the lid separates from the base, then retrieving the contents inside.
- Using a stick to push a banana that's out of reach through a fence gap, by positioning and moving the stick in the correct direction.

Level 1 **Very low.** Tasks at this level require understanding of basic, explicit meanings in simple formats, including recognition of common words, straightforward statements, and clear one-to-one relationships between symbols and their meanings. Comprehension is limited to surface-level, literal interpretations without need for context or inference. **Examples**:

- Identifying basic subject-verb relationships that describe observable actions (e.g., "Context: The blue bird was flying high in the sky. Question: who was flying?").
- Understanding simple questions that do not require sophisticated language skills such as "Why is the sky blue?"
- Understanding single-step instructions where the action directly matches the command (e.g., comprehending the sentence "close the door for me" and mentally connecting these words and the corresponding physical action).

Level 2 **Low.** Tasks at this level involve comprehending straightforward messages with basic context, including simple cause-effect relationships, clear sequential instructions, and explicit connections between ideas. Understanding requires basic inference but remains tied to concrete, clearly stated information. **Examples**:

- Capable of answering "why" questions about a simple story (e.g., "Why did the girl take an umbrella?" after reading "Sarah saw dark clouds in the sky. She grabbed her umbrella before leaving home.")
- Understanding simple explanations of processes (e.g., "Plants need water and sunlight to grow, otherwise they will not survive, especially in harsh climate.")
- In a recipe interface, interpreting "Add milk slowly while stirring continuously until mixture thickens" by understanding that the stirring must occur simultaneously with the milk addition, not after.

Level 3 **Intermediate.** Tasks at this level require understanding of moderately complex information including implicit meanings, metaphorical language, and relationships between multiple concepts. Comprehension may involve processing both explicit and implicit information. **Examples:**

- In a high school student's history essay about the Industrial Revolution, following their argument that "While factories created more jobs in cities, this urbanization ironically decreased quality of life because cramped living conditions and poor sanitation led to disease outbreaks." This requires understanding how the student is connecting multiple historical factors (industrialization, urbanization, living conditions, public health) and recognizing their use of "ironically" to highlight the unexpected negative consequence of economic progress.
- In an employee handbook, understanding that the statement "The company values work-life balance" combined with "Employees are expected to be responsive to urgent matters outside office hours" represents a potential policy contradiction requiring contextual judgment.
- In a technical manual, interpreting a troubleshooting section that requires understanding how different error messages might indicate the same underlying problem depending on the system's state.

Level 4 **High.** Tasks at this level demand comprehension of sophisticated content with multiple layers of meaning, complex relationships between concepts, and nuanced implications. Understanding requires integration of various information sources and recognition of subtle patterns and connections. **Examples**:

- Following an accessible fiction story told from multiple viewpoints where each narrator provides partial, biased information, requiring the reader to construct the true sequence of events by reconciling conflicting accounts and recognizing each narrator's limitations and motivations.
- Understanding a complex academic argument that develops through multiple chapters, where key terms are gradually redefined and earlier arguments are recontextualized by later developments.
- Interpreting a modern theatrical play where dialogue has multiple meanings based on staging directions, character backgrounds, and historical context, requiring simultaneous understanding of textual and performative elements.

Level 5+ **Very High.** Tasks at this level require mastery in understanding highly convoluted, abstract, and interconnected information systems, including sophisticated theoretical frameworks, convoluted narratives and nuanced philosophical arguments. Comprehension involves synthesizing multiple complex perspectives and understanding subtle distinctions. **Examples**:

- Understanding well a convoluted legal document that requires tracking multiple cross-references, understanding nested conditions, and comprehending how different clauses modify each other.
- Comprehending a modernist novel that uses a stream-of-consciousness narrative technique where multiple timelines, memories, and internal thoughts are interwoven without clear demarcation, requiring readers to track subtle linguistic shifts (changes in tense, pronouns, or narrative voice) to understand when the narrative moves between present action, past memories, imagined futures, and other characters' perspectives.
- Understanding a convoluted visual narrative where multiple story threads are told simultaneously through different visual styles on the same page, requiring understanding of how the visual elements interact, conflict, and complement each other to create meaning. For example, a graphic novel page where realistic drawings depict current events, sketchy portions represent memories, and geometric patterns show emotional states, all interacting to tell a coherent story.

R2. Verbal Expression (CEe)

This rubric evaluates the difficulty of a task's expression requirements, encompassing the generation and articulation of ideas, stories, or semantic content in different formats or modalities. It may include the usage of the right vocabulary, adoption of the appropriate genre, formulation of explicit and implicit meanings, creation of relationships between concepts, incorporation of contextual information, expression of abstract ideas and complex systems, and transformation of sophisticated content into a smooth narrative. Noteworthy, the need of specialized vocabulary or jargon in the expression does not necessarily indicate a high level of difficulty, as these terms may be used within simple, straightforward linguistic structures requiring minimal compositional complexity and are more reflective of domain knowledge rather than expression sophistication. In addition, the difficulty level should correspond to the simplest expression effort to successfully solve the task, given that a solution to a task may be formulated in various ways with varied linguistic complexity; multiple-choice questions, even if the options are long and complex, generally do not require language expression beyond the basic level, so they are typically level 1. The rubric ranges from tasks requiring no meaningful expression to those demanding the generation of highly sophisticated, interconnected content, including the ability to create convoluted narratives, convey nuanced implications, and express multiple perspectives across different domains and levels of abstraction.

Levels

Level 0 **None.** Tasks at this level involve no meaningful expression or communication, limited to automatic responses or simple pattern reproduction. The task can be completed through purely mechanical or algorithmic processes without any generation of meaning. **Examples**:

- Repeating a sound pattern exactly as heard without understanding or modifying its meaning.
- Copying text from one format to another without generating or modifying content.
- Reproducing a sequence of gestures through simple mimicry.

Level 1 **Very low.** Tasks at this level require expressing basic, explicit meanings in simple formats, including use of common words, straightforward statements, and clear one-to-one relationships between ideas and their expression. Expression is limited to surface-level, literal articulation without need for context or nuance. **Examples**:

- Stating immediate needs like "I need water" in a simple, direct, unambiguous way.
- Solving a task that requires domain expertise to get the right answer but the answer only requires basic expression ability (e.g. "Given the product SMILES: `O=C(NC1CCN(CCc2ccccc2)CC1)c1c[nH]c2ccc(F)cc12`, predict the reactants SMILES".
- Multiple-choice QA questions, where the subject only needs to choose one readily available option, even though the accurate answer option may be formulated in a linguistically complex manner (e.g. "The correct answer is option C. Reynolds and Khripkova would not make suitable business partners, [...], if they quarrel, know how to resolve their differences.")

Level 2 **Low.** Tasks at this level involve producing straightforward messages with basic context, such as simple cause-effect relationships, clear sequential instructions, and explicit connections between ideas. Expression requires basic organization but remains tied to concrete, clearly stated information. **Examples**:

- "Writing step-by-step instructions for making a sandwich, clearly indicating the sequence of actions and basic quantities needed."
- Creating a brief email to schedule a meeting, specifying time, place, and basic purpose.
- Describing a simple process like plant growth, connecting the basic sequence of events: "First the seed needs soil and water, then it grows roots, then it sprouts leaves."

Level 3 **Intermediate.** Tasks at this level require generating moderately nuanced information, with attention to both content and presentation style. This includes selecting field-appropriate vocabulary, adapting to specific genres (like technical documentation or clinical notes), and creating coherent narratives that smoothly connect ideas. Expression may involve conveying both explicit and implicit information while maintaining consistent tone and voice throughout the text. **Examples:**

- Writing explanatory notes for a simple geometry proof that guides the reader through the logic: "To prove these triangles are similar, we first show their angles are equal. The alternate angles formed by these parallel lines are equal, and since both triangles share this angle at point A, we can conclude..."
- Writing product documentation that anticipates user confusion: "While the red indicator light typically signals an error, in sleep mode it indicates normal operation. If the light flashes red during active use, consult the troubleshooting guide."
- Writing short clinical notes that connect symptoms with potential causes: "Patient presents with persistent cough and fatigue for 2 weeks. Given their recent travel history and exposure to dusty environments, considering both viral upper respiratory infection and environmental allergies as potential causes."

Level 4 **High.** Tasks at this level demand generating sophisticated content with multiple layers of meaning, complex relationships between concepts, and nuanced implications. Such expressions may include the usage of linguistically advanced vocabulary and rhetorical devices, careful attention to genre conventions, and the ability to integrate multiple perspectives and communicate subtle patterns and connections. **Examples**:

- Writing lecture notes that integrate multivariable calculus with linear algebra to explain the connection between Jacobian matrices, coordinate transformations, and volume changes in higher dimensions.
- Writing technical documentation that addresses multiple user levels simultaneously: "The API's modular design allows for both simple plug-and-play implementation for basic use cases and sophisticated customization through advanced configuration options, ensuring scalability as your needs evolve."
- Writing a detailed legal brief that weaves together statutory requirements, case law precedents, and policy implications: "While Smith v. Jones (2019) established a broad interpretation of 'reasonable care,' the specific circumstances of our case, combined with the legislative history of Section 47(b), suggest that this standard should be qualified when applied to specialized industrial settings..."

Level 5+ **Very High.**Tasks at this level require mastery in generating convoluted, abstract, and interconnected content, including nuanced vocabulary, convoluted narratives, deep arguments, and conveying multiple perspectives and subtle distinctions simultaneously. **Examples**:

- Writing a few paragraphs of a graduate-level textbook section that develops the relationship between Lie groups, Lie algebras, and differential manifolds.
- Creating a convoluted multi-layered narrative that simultaneously develops several plot threads through carefully structured revelations, such as a novel seemingly disconnected opening chapters gradually revealing their interconnections through subtle linguistic echoes and thematic resonances, allowing readers to piece together the full story while maintaining tension across multiple timelines.
- Writing well-thought comprehensive hospital policy guidelines that address complex medical, legal, and ethical considerations: "The protocol for experimental treatments must balance patient autonomy, clinical evidence requirements, and legal liability considerations. When standard treatments are exhausted, the following decision tree integrates real-time clinical assessment, informed consent documentation, ethics committee review, and liability mitigation steps, while maintaining compliance with both state regulations and international medical ethics standards..."

Conceptualisation, Learning, and Abstraction Rubric (CL)

This rubric assesses the difficulty level of tasks requiring conceptualization, learning, and abstraction during the completion of tasks. It evaluates the extent to which a task necessitates the formation of new concepts, engagement in inductive and analogical reasoning, mapping of relationships between domains, and the generation of abstractions from concrete examples. Higher levels on this scale represent increasing demands for real-time learning, pattern identification, hypothesis formation, analogical transfer, and the contrast of knowledge across diverse domains.

Levels

Level 0 **None.** The task requires no conceptualization, learning, abstraction, inductive or analogical reasoning. It involves applying well-established procedures or recalling known information, even for complex tasks. No new abstractions, analogies, or learning occur during task execution. **Examples**:

- Performing basic one-digit arithmetic multiplications using memorized multiplication tables (like 3x3 = 9).
- Sorting short texts into predefined categories based on a list of indicator words, without inferring new indicators.
- What was the name of Abraham Lincoln's father?

Level 1 **Very low.** The task involves minimal conceptualization, learning, inductive or analogical reasoning. It requires simple pattern identification or following basic instructions, with very limited generalization or basic surface-level analogies occurring during the task. **Examples**:

- Continuing a basic letter sequence (e.g., "a, c, e, g, __").
- "Given a red circle, a red square, a red triangle and a blue pentagon, find the one out, which is the blue pentagon."
- Given a pair of words (like "hot and cold"), choose another pair from a list that shares the same relationship. For example, if "hot" and "cold" are opposites, you'd look for another opposite pair like "up and down."

Level 2 **Low.** The task requires basic conceptualization, learning, inductive and analogical reasoning. It involves generalizing from a small set of examples, applying simple analogies to closely related domains, or applying simple instructions to new but closely related tasks. **Examples**:

- Given the sentence 'As it started raining, Alice opened her brolly.' inferring the meaning of the unknown word (brolly) by using surrounding context clues, forming a basic abstraction about its possible definition.
- In a fictional planet, observing in a garden where light yellow and light orange plants grow towards light sources over time but dark blue and dark red plants don't, and forming a basic hypothesis between the colors and plant behavior.
- Adapting a solution from a previously solved secondary school math problem to a new problem with very similar structure but different surface features (e.g. numbers, names and context). While the core mathematical approach remains similar, the adaptation still requires recognizing how small variations in the new problem might require adjustments to the original solution method.

Level 3 **Intermediate.** The task involves moderate conceptualization, learning, and inductive and analogical reasoning. It requires recognizing broader patterns, applying analogies across moderately different domains, and forming more complex hypotheses through analogical reasoning. **Examples:**

- Reading passages where certain words are consistently replaced with nonsense words: 'The zork lives in a tree. The small zork ate berries. Many zorks gather in winter [...]. The tired zork slept quietly'. Through the multiple examples, learning not just that 'zork' likely means 'squirrel', but also understanding how it follows plural rules ('zorks'), can be modified by adjectives ('small zork', 'tired zork'), and performs actions typical of animals.
- While playing a strategy game named Xiangqi (also known as Chinese chess) without any prior experience on it, coming up with some effective tactics through repeated observations and trials as well as some past experience playing chess.
- Given data about plant growth in artificial conditions where light color, temperature, and humidity vary cyclically, observing that plants develop different leaf patterns depending on which factor changes first each day. Through systematic observation, forming basic hypotheses about how the sequence of environmental changes affects growth patterns.

Level 4 **High.** The task requires substantial conceptualization, inductive and analogical reasoning, and abstraction, involving the integration of multiple concepts, creating complex analogical mappings across diverse domains, and forming and testing complex hypotheses. **Examples**:

- Working with a collection of text messages where response times vary significantly. Through analysis, discovering that certain word combinations, sentence structures, and punctuation patterns consistently correlate with faster or slower response times, then using these insights to predict likely response speeds for new messages.
- While learning Go after experience with chess and Xiangqi, discovering how stone formations serve multiple strategic purposes that differ fundamentally from piece-based games. Through systematic play and analysis, understanding how a group of stones can simultaneously secure territory, threaten invasion, and maintain connectivity with other groups. This requires substantial abstraction beyond piece-movement games to grasp how value emerges from stone relationships rather than individual pieces.
- Working with a sequence of pattern acceptance tests where rules change systematically. For instance, in judging whether grid arrangements of colored shapes are "valid": early patterns are accepted based on color adjacency (e.g., "red must never touch blue"), then the rule shifts to consider shape orientation (e.g., "triangles must point toward squares"), and finally combines both aspects (e.g., "red triangles must point toward blue squares"). The systematic nature of the rule changes follows a clear progression from simple single-attribute rules to combined rules. The subject must track these rule evolutions to correctly predict which new grid arrangements would be considered valid, understanding that rules become progressively more complex by combining previous attributes rather than introducing entirely new concepts.

Level 5+ **Very High.** The task involves very advanced conceptualization, inductive and analogical reasoning, and abstraction. It requires generating new analogical frameworks in real-time, mapping relationships across highly diverse and abstract domains, or solving complex problems through novel analogical insights. **Examples**:

- Solving a visual puzzle where three different properties (symmetry, rotation, and scaling) must be understood at both the element level and the pattern level. For instance, individual shapes follow one set of transformation rules, while the overall arrangement follows a different set of rules, and the relationship between these two rule sets must be discovered to predict the next state.
- Designing a new electronic musical instrument after studying blueprints of synthesizers, amplifiers, and effect pedals. This requires abstracting core principles of signal generation, processing, and control from each device (oscillation, filtering, envelope shaping, feedback), understanding how these principles create different sonic characteristics, and then creatively recombining them to produce new types of sounds. The task demands identifying how fundamental concepts manifest differently across devices (like how feedback creates sustain in an amplifier but modulation in a ring modulator), then synthesizing these insights to create sound-generating mechanisms.
- Working with a sequence of pattern acceptance tests where rules evolve with increasing abstraction and self-reference. Starting from 'red triangles must point toward blue squares', patterns evolve to where shapes establish relationships based on their relative properties. For instance, shapes with more sides must point toward shapes with fewer sides, but this relationship inverts when the shapes share colors. Furthermore, each valid pattern must mirror a small-scale arrangement within its larger structure - if three triangles form a particular relationship on one side of the grid, the overall shape arrangement of the entire grid must follow that same relationship. The subject must discover these nested self-referential patterns and predict how they apply at different scales, requiring both pattern recognition and the generation of new frameworks for understanding how rules can reference themselves.

Metacognition and Critical Thinking (MC)

R1. Critical Thinking Processes (MCt)

This rubric assesses the difficulty level of metacognitive engagement required by the question. More concretely, the level represents the extent to which the question requires the respondent to monitor or regulate multiple thought processes to answer the question effectively, ranging from simple recall to high-level critical thinking.

Levels

Level 0 **None.** No critical thinking or analysis is needed. **Examples**:

- Clapping one's hands with another entity.
- Simple recall of facts without further processing.
- Recognizing a familiar face from a photograph.

Level 1 **Very Low.** The task requires recall or recognition of facts, with a low level information processing required. The respondent needs to retrieve information directly from memory or identify very obvious relationships. There is no need for critical thinking or analysis beyond the most elementary level. **Examples**:

- Selecting the correct meaning of a common word from multiple clearly distinct definitions.
- Matching simple synonyms, such as "big" and "large".
- Question: What was the time 5 years and 6 months before Jan, 1956?

Level 2 **Low.** The task involves mostly straightforward comprehension or application of known concepts, with some information processing. The respondent may need to demonstrate understanding by explaining ideas, making simple comparisons, or applying concepts in familiar contexts. A low-level of critical thinking is required, such as recognizing generally obvious patterns or making simple categorizations. **Examples**:

- Answering the question: 'What happens in Cinderella when the clock approaches midnight?' The answer requires explaining that Cinderella must flee because her magical transformation will end.
- Giving the smallest amount of coins as change from a purchase.
- Answering the question: "How many solid $1 \times 1 \times 1$ cubes are required to make a solid $2 \times 2 \times 2$ cube?"

Level 3 **Intermediate. Description:** The task necessitates a considerable amount of analysis or synthesis of information. The respondent needs to engage in moderate critical thinking, such as identifying patterns, making inferences, or applying concepts to new situations. **Examples**:

- Analyzing the symbolism in a poem and explaining how it contributes to the overall theme.
- Identifying potential biases in a news article and explaining their impact on the information presented.
- Explaining how a price reduction could lead to increased overall revenue through its effect on sales volume.

Level 4 **High.** The task demands advanced critical thinking skills, including evaluation of complex ideas, analysis of multiple perspectives and assumptions, or creation of new concepts. The respondent must maintain consistent awareness of thinking processes and potential biases. **Examples**:

- Evaluating a school's proposal to extend the lunch period by examining the evidence for improved student focus, considering impacts on different stakeholders like teachers and students, and analyzing how personal preferences might affect one's assessment of the policy.
- Designing a study to compare two teaching methods for basic math by identifying potential sources of bias, developing fair assessment criteria, and planning how to control for differences in student ability levels.
- Analyzing the role of bread prices in the French Revolution by examining economic data from different regions, comparing its major impact between urban and rural areas, and evaluating how food scarcity combined with tax burdens and wage stagnation influenced public unrest.

Level 5+ **Very High.** The task demands the highest level of critical thinking, requiring sophisticated metacognitive strategies focused on examining reasoning processes, identifying logical fallacies, evaluating competing arguments, and reaching well-reasoned conclusions. The respondent must reflect on their own thinking processes, assumptions, and biases while engaging with complex ideas. **Examples**:

- Analyzing a proposed economic study that claims to prove racial discrimination in hiring by examining the researchers' unstated assumptions about causality, identifying potential confounding variables they haven't controlled for, evaluating whether their statistical methods actually support their conclusions, examining your own potential biases about the topic, and determining what can and cannot be legitimately concluded from their methodology - all while maintaining awareness of how your own socioeconomic background might influence your analysis.
- Evaluating a complex court case by dissecting the logical structure of competing arguments from prosecution and defense, identifying unstated assumptions in witness testimony, examining how your own biases about the defendant might affect your judgment, analyzing the credibility and limitations of different pieces of evidence, and reaching a conclusion while explicitly acknowledging areas of reasonable doubt and uncertainty.
- Breaking down a philosophical argument about consciousness by identifying circular reasoning and unstated premises, examining how different definitions of key terms affect the argument's validity, evaluating the credibility of thought experiments used as evidence, testing the argument's logical consistency, recognizing your own presuppositions about the nature of mind and reality, and determining which conclusions are truly warranted by the premises.

R2. Calibrating Knowns and Unknowns (MCu)

This rubric assesses how difficult it is for the respondent to accurately evaluate whether they know or don't know something. It focuses on metacognitive assessment — the ability to recognize the boundaries of one's knowledge and confidently identify what one knows they know, knows they don't know, or is uncertain about.

Levels

Level 0 **None.** The respondent can immediately and unambiguously determine whether they know something or know they don't know it. No metacognitive effort is required. **Examples**:

- "Tell me the typical colour of the sky on Earth during the day?"
- "Can it snow when the environmental temperature goes up to 40 degree celsius?"
- "What's the name of Taylor Swift's maternal grandmother?"

Level 1 **Very low.** Minimal metacognitive effort is needed to determine whether one has the knowledge to answer. The boundaries between knowing and not knowing are very clear. **Examples**:

- "Calculate 164942+26250737, tell me if you think you have done it well." (the score is good if the sum is right and says correct, or if the sum is wrong and says incorrect)
- "How many hairs did Barack Obama have exactly when he woke up on the morning of March 1, 2024?" (the score is good if the answer given or chosen is that it can't be known)
- "Given $2w + 4t = 14$ and $4w + 5t = 25$, calculate the value of $2w + 3t$ and tell me if you think you have done it well." (the score is good if the answer is right and says correct, or if the answer is wrong and says incorrect)

Level 2 **Low.** Some metacognitive effort is required to assess the boundaries of one's knowledge, but the assessment is still relatively straightforward. **Examples**:

- "Given the new breakthroughs in chemistry this year, explain how to synthesise gold out of boiling both blonde hair and metals made of bronze" (the score is good if the task is refused, as it is clearly not possible).
- "Say something that indicates your level of Estonian" (assuming you know a bit of Estonian).
- Determine for some simple arithmetic operations with logarithms of base 2 when you can do it yourself or require a calculator.

Level 3 **Medium.** Moderate metacognitive effort is required to determine the boundaries of one's knowledge. There may be some uncertainty about whether one truly knows something or just thinks they might know it. **Examples**:

- "Solve a simple 9x9 Sudoku puzzle" (since it requires tracking which numbers are certain about in each 3x3 box versus numbers that have only been partially eliminated).
- "Given a detailed passage about the American Civil War mentioning several battles and dates, identify which specific facts you're confident enough to verify versus those you'd need to research."
- "When presented with a system of three linear equations, determine whether you know enough about elimination and substitution methods to solve it completely or if you might be missing key steps."

Level 4 **High.** Significant metacognitive effort is needed to determine whether one truly knows something or just has partial knowledge. The line between knowing and not knowing becomes blurry. **Examples**:

- "Given a dataset of 100 observations with 20% missing values randomly appearing across different variables of interest and potential sampling bias, assess whether you can confidently identify which statistical conclusions are reliable versus which might be affected by unknown factors in the data collection process."
- "Given a patient with symptoms of fever, fatigue, and joint pain, determine which potential diagnoses you can confidently rule out versus which require additional information or testing."
- "When analyzing a legal document with multiple clauses and cross-references, identify those parts you can interpret with certainty versus those that require expert consultation."

Level 5+ **Very High.** Extremely challenging to determine the boundaries between what one knows and doesn't know. Requires sophisticated metacognitive assessment to avoid overconfidence or underconfidence. **Examples**:

- "Predict how much a machine learning model's accuracy will drop (if any) over the next 6 months for a system that classifies which emergency room patients are at high risk of developing complications within the next 24 hours, given evolving disease patterns, changes in hospital protocols, potential new variants, seasonal effects, varying patient demographics, and changing physician response patterns."
- "In a Texas Hold'em poker hand, after the flop, determine your winning probability with J♠K♠, three low hearts on the board, and five opponents you've been playing with for some time."
- "During an ongoing international crisis (e.g., a major conflict or global financial crisis), determine whether to invest a significant portion of your portfolio in seemingly undervalued stocks, considering factors such as market psychology, geopolitical developments, supply chain disruptions, currency fluctuations, central bank responses, and potential long-term structural changes to affected industries."

R3. Identifying Relevant Information (MCr)

This rubric assesses the difficulty of the metacognitive processing required by the respondent to identify the information necessary during the process of solving the task with a given set of information. More concretely, it involves the respondent's ability to recognize what information helps solve the task or does not, and how this recognition process unfolds as they work toward the solution.

Levels

Level 0 **None.** All necessary information is immediately apparent and directly applicable to solving the task, or no information is provided and none is needed. No metacognitive processing is required to identify relevant information during problem-solving. **Examples**:

- "What is the capital of France?"
- "What is 2513441 + 7519239519281?"
- "How many sports correspond to IPTC Newscode mediatopic/20000960?"

Level 1 **Very low.** Most relevant information is readily identifiable, with minimal extraneous details. The respondent needs to do simple filtering or selection of information as they proceed with solving the task, but the relevance of information remains clear throughout the process. **Examples**:

- "John has 5 apples and 3 oranges. How many apples does John have?"
- "Alice's mother has several brothers, one married to Helen, who currently lives in Barcelona. What's Helen with respect to Alice?"
- "The recipe calls for 4 cups of flour and 2 cups of sugar. How many cups of flour are needed?"

Level 2 **Low.** A fair amount of potentially relevant information is provided, mixed with some irrelevant details. As the respondent works through the problem, they need to evaluate which pieces of information are useful for the next step in their solution process, requiring ongoing but straightforward metacognitive assessment. **Examples**:

- "Sarah went to the grocery store on Tuesday. She bought 3 oranges for $0.50 each, 2 apples for $0.75 each, and a loaf of bread for $2.25. What was the total cost of the fruit Sarah purchased?"
- "In preparation for a marathon, James ran 5 miles on Monday, 8 miles on Wednesday, and 6 miles on Saturday. He also trained at the gym for 2 hours each week. How many miles did James run in total?"
- "Mary took photos of the Colosseum at sunset and visited the Vatican Museums where she spent two hours studying the famous ceiling of the Sistine Chapel. She also bought souvenirs for her friends and got lost trying to find her hotel. What did Mary observe at the Vatican Museums?"

Level 3 **Medium.** The respondent must engage in moderate metacognitive processing throughout the problem-solving process in one or both of these ways: (1) evaluating and reconciling potentially conflicting or redundant information that serves as distractors within a manageable search space, or (2) recognizing what additional information or problem-solving approaches are needed when not all relevant information is explicitly provided, but the possible solution paths remain relatively constrained. Some information that seems irrelevant initially may become important later, or some unstated information may become crucial to identify as the solution progresses. **Examples**:

- "A student's short essay discusses how Shakespeare's character Hamlet shows signs of depression. In the essay, it states that Hamlet speaks harshly to Ophelia in Act 3, telling her 'Get thee to a nunnery' and refusing her love. The essay also mentions his soliloquy 'To be or not to be,' his wearing of dark clothes at court, and his Act 1 conversation with Horatio about his father's ghost. The essay is 1000 words long and includes quotes from Acts 1, 3, and 5. What evidence does the essay present about Hamlet's interactions with Ophelia?"
- "A news article discusses a local park renovation project, mentioning the park's historical significance from the 1950s, current visitor numbers, planned new features including a playground and walking paths, the project's $500,000 budget split across different improvements, debates about preserving old trees versus adding parking spaces, and quotes from both the project manager and local residents about their memories of childhood visits. What are the specific new features planned for the park renovation?"

Level 4 **High.** The problem-solving process requires sophisticated metacognitive strategies throughout, with a large search space to navigate. This could involve either: (1) evaluating multiple possible interpretations of significant amounts of conflicting/redundant information that serves as distractors, requiring exploration of various combination possibilities, or (2) identifying crucial unstated information or approaches needed for solution while considering multiple possible solution paths and their implications. The respondent must frequently reassess their understanding and adjust their approach as they either discover new connections between provided information or recognize important unstated elements needed for solving the task. **Examples**:

- "In this escape room scenario, you find a desk with a locked drawer, a calendar marked with different colored circles, a bookshelf with titles in various languages, and a wall clock showing 3:45. On the desk, there's a note that reads 'Time reveals knowledge, knowledge unlocks secrets.' A painting on the wall shows a sunset over a library, and there's a globe with certain cities marked with stars. Each time you examine an object, you notice new details that might connect to others. How can you open the locked drawer?"
- "Assume that there exist only two types of people: knights and knaves. Knights always tell the truth, while knaves always lie. You are given the statements from 6 characters. Based on their statements, infer who is a knight and who is a knave. A: E is a liar if and only if C is a liar. B: If D is a liar, then E is a liar. C: E is a truth-teller and F is a truth-teller. D: C is a liar if and only if B is a liar. E: If B is a liar, then C is a truth-teller. F: B is a liar if and only if A is a liar."
- "A customer survey about a new phone model gathered feedback through three methods: online reviews mentioned battery life lasting 'all day', 'about 12 hours', or '14-16 hours'; in-person interviews reported battery performance as 'excellent', 'better than previous model by 4 hours', or 'lasting from morning to night'; and usage data showed power consumption patterns varying between 10-18 hours depending on features used. Technical specs list battery capacity, screen brightness impact, and various power-saving modes. What can be concluded about the phone's actual battery life?"

Level 5+ **Very High.** The problem-solving process demands constant high-level metacognitive monitoring and regulation in challenging conditions: either most of the provided information is redundant, misleading, or contradictory (while remaining solvable), or crucial information about solution approaches and constraints is left unstated and must be discovered. The respondent must maintain awareness of many possible interpretation frameworks or solution paths simultaneously, regularly revisiting their understanding as they either recontextualize conflicting information or identify necessary unstated information and constraints. **Examples**:

- Riddles such as: "I am found in ancient scrolls and modern screens, made of nothing but seen by all, I dance between light and dark, born in storms yet living in peace, flowing like water but dry as sand, silent as night but telling stories, changing shape with every eye yet always staying the same. Sometimes I march in straight lines, other times I curl and twist, I can be bold or gentle, thick or thin, but I never truly exist. What am I?" (the answer is "shadow")
- "Solve this cryptic crossword puzzle: 'Stop for break, drink coffee and tea endlessly, stir milk around in a mug - useless without morning essentials!'" (the answer is "breakfast")
- "A restaurant review contains extensive details about the reviewer's experience: describes the rainy drive to the location, the hostess's friendly greeting, memories of their grandmother's cooking, opinions about the restaurant's decor choices, a lengthy story about their career as a food critic, descriptions of fellow diners' conversations, commentary about parking difficulties, their favorite recipes, the day's weather forecast, and briefly mentions in different places that the pasta was 'perfectly cooked', 'somewhat firm', 'just right', and 'could have been softer'. What was the reviewer's assessment of the pasta's texture?"

Mind Modelling and Social Cognition (MS)

This criterion assesses the level of cognitive demands associated with mind modelling of others and social cognition. The level of cognitive demands progresses from tasks that require no mind modelling (specifically, the ability to model the minds of other agents) or social cognition to those that require reasoning about how the beliefs, desires, intentions, and emotions of multiple other agents might interact to determine future behaviours.

Levels

Level 0 **None.** The task does not require mind modelling or social cognition. It may not involve other agents, or if it does, perceiving or interacting with those agents is not necessary to complete the task. **Examples**:

- Solving a Sudoku puzzle independently.
- Operating a dishwasher according to its instruction manual.
- Reading a book silently to yourself, even if others are present in the room.

Level 1 **Very low.** Performance in this task is improved through the detection or recognition of other agents and by basic social learning (e.g., imitation). Critically, reasoning about observed behaviour or attributing mental states to others is not required for good performance in this task. **Examples**:

- Mimicking someone's hand gestures during a conversation.
- Following another person's gaze to find where they left their keys.
- Copying the sequence of buttons someone presses to operate a vending machine.

Level 2 **Low.** This task requires some basic intuition about the behaviour of others, but only minimal levels of mental state attribution. Good performance might be based on developing accurate associations between other's responses and the stimuli that caused them. Note, this reasoning need not be explicit. **Examples**:

- Recognizing that someone using a rock to crack open a coconut is trying to get to the food inside.
- Identifying that someone's scrunched nose and turned head means they don't like the smell of spoiled milk.
- Solving an abstract logic problem in which only minimal levels of mind modelling is needed (e.g. "Assume that there exist only two types of people: knights and knaves. Knights always tell the truth, while knaves always lie. You are given the statements from 3 characters. Based on their statements, infer who is a knight and who is a knave. A: C is a liar. B: C is a truth-teller and A is a truth-teller. C: B is a truth-teller and A is a liar.")

Level 3 **Intermediate.** This task goes beyond simple state–behaviour associations and involves attributing cognitive or affective states (i.e., mentalising). That is, it involves inferring specific mental properties about others (e.g., "they believe the moon landing was a hoax", "they want a glass of water"). The task may not, however, require explicit reasoning about these mental states (i.e., full-blown theory of mind). **Examples**:

- Telling a colleague about a mutual friend's new job, knowing they haven't heard the news yet and thus might be interested.
- Finding a good hiding spot in hide-and-seek by visualizing where the seeker might look.
- Recognizing that someone checking their watch repeatedly during a meeting probably wants to leave.

Level 4 **High.** This task requires a full theory of mind to be solved effectively. It requires not only the attribution of mental states to others, but explicit reasoning about those states. It may also require the integration of social knowledge and heuristics about normal agentic behaviour to accurately predict future behaviour. Importantly, this task also requires a clear distinction between self- and other-related representations. **Examples**:

- Developing an intuitive theory about an agent's future behaviour such as understanding that Sally will look for her marble in the basket where she left it, even though Anne moved it to the box when Sally was away.
- Distinguishing between one's own emotional reaction to a friend's story and what the friend is feeling.
- Recognizing not to point out a spelling mistake in your manager's presentation based on their emotional state, personality, and the social context.

Level 5+ **Very High.** This task requires exceptional mind modelling and social cognition abilities. It goes beyond generating intuitive theories about another agent within a dyadic interaction, and instead requires the combination of multiple theories of mind corresponding to the intentions, emotions, and beliefs of a range of different agents. Expanding the scope of mind-modelling and social cognition to include multiple agents would enable more sophisticated forms of collaborative action. Tasks at this level may require an understanding of the complex networks and hierarchies that form within social groups. **Examples**:

- Comprehending the plot of a romance novel or the "social drama" at a dinner party that requires modelling the mental states of multiple agents (e.g., "I heard that Jane told Steve that his girlfriend Abigail wanted to leave, but that he didn't believe her, thinking Jane was just causing trouble because she had seen Abigail talking to her boyfriend Andrew. . . ").
- Appreciating the behaviour of individuals within a work team and managing the situation in which one employee has misinterpreted another's actions as deliberately unhelpful, which has created tension that affects the whole group's dynamics.
- Leading a negotiation between multiple stakeholders where each party has different beliefs about others' intentions and bottom lines, while managing the complex emotional dynamics between opposing personalities.

Quantitative and Logical Reasoning (QL)

We capture here those kinds of reasoning that are mostly deductive: a first subdomain for logical reasoning and another subdomain for quantitative reasoning. Inductive reasoning is included in another rubric (Conceptualisation, Learning and Abstraction).

R1. Quantitative Reasoning (QLq)

This criterion assesses the difficulty level of a task in requiring working with and reasoning about quantities, numbers, and numerical relationships. More specifically, the level represents the complexity of numerical operations and quantitative concepts needed to solve the task, ranging from simple counting and arithmetic to sophisticated analysis involving multiple quantitative variables, relationships, and transformations. The scale's difficulty increases based on factors such as the number of quantities involved, the complexity of numerical relationships, and how much quantitative information must be derived.

Levels

Level 0 **None.** The task does not require quantitative reasoning. **Examples**:

- "Describe the color of the sky on a clear day."
- "Name a type of pet commonly found in households."
- "What is the capital city of Japan?"

Level 1 **Very low.** The task involves only basic or rudimentary quantitative concepts (e.g., simple counting, basic comparisons). Requires only simple recall of basic quantitative facts. Requires minimal quantitative reasoning. **Examples**:

- "Count the number of eggs in a small basket."
- "If your friend has 7 apples and you have 5, who has more apples?"
- "Given this molecular requirements description, design a new molecule: The molecule is trianion of xanthosine 5'-triphosphate arising from deprotonation of three of the four free triphosphate OH groups. It is a conjugate base of a XTP."

Level 2 **Low.** The task requires relatively simple quantitative operations and concepts. Involves generally straightforward application of low-level mathematical principles. Some explicit quantitative reasoning is required. **Examples**:

- "Calculate the average rainfall for a week if it rained 2 litres per square meter on Monday, 1 litre per square meter on Wednesday, and 4 litres per square meter on Friday."
- "If a recipe calls for 2 cups of flour to make 12 cookies, how much flour is needed to make 18 cookies?"
- "A store is offering a 20% discount on a $50 shirt. What is the final price of the shirt?"

Level 3 **Intermediate.** Involves moderately complex quantitative concepts and relationships. Requires application of non-trivial mathematical principles. Some necessary quantitative information may need to be inferred or calculated. Requires active engagement in quantitative reasoning processes. **Examples**:

- "If bacteria double every 30 minutes and you start with 100 bacteria, how long will it take to reach 10,000 bacteria? Show your reasoning process."
- "Calculate the future value of a $1000 investment over 5 years at an annual rate of 5%, compounded quarterly, if $200 is added to the investment at the end of each year. Show the impact of these additional contributions."
- "A car travels at 60 kph for 2 hours, then at 45 kph for 1.5 hours, and finally at 30 kph for 1 hour over varied terrain. What is the average speed of the car and what is the total distance traveled?"

Level 4 **High.** Requires complex quantitative operations. Involves the application of advanced mathematical concepts. Uses sophisticated numerical representations and relationships. Much necessary quantitative information may need to be inferred or generated. Requires an advanced level of quantitative reasoning processes. **Examples**:

- "Using calculus, find the volume of the solid formed when the region bounded by the curves $y = x^2$ and $y = 4 - x^2$ is rotated around the y-axis."
- "Determine the stability of a system of differential equations by finding the eigenvalues and eigenvectors of its matrix representation, and then analyze whether the system converges to an equilibrium and discuss the implications of different eigenvalue scenarios on system stability."
- "Solve the second-order differential equation $\frac{d^2y}{dx^2} + 4\frac{dy}{dx} + 4y = 0$, and analyze the stability of its solutions."

Level 5+ **Very High.** Involves extremely complex quantitative reasoning and mastery of mathematical insight. Requires the very complex integration of mathematical concepts. Uses abstract mathematical representations and theoretical quantitative concepts. Most necessary quantitative information may need to be inferred or generated through complex reasoning. Requires constant engagement and adjustment of quantitative reasoning at the expert level. **Examples**:

- "Create a stochastic model for predicting future stock market trends using simulations and accounting for variables such as economic indicators, investor sentiment, and geopolitical events."
- "Using advanced statistical methods, design an experiment to test the efficacy of a new drug while accounting for multiple confounding variables and potential interaction effects."
- "Formulate a non-linear optimisation model for maximizing renewable energy output in a smart grid system, incorporating constraints such as energy storage capacity, variable supply and demand, and government regulations."

R2. Logical Reasoning (QLI)

This rubric evaluates the logical reasoning demands of tasks across six levels of difficulty, focusing exclusively on deductive reasoning, which is required whenever a task involves matching and applying rules, procedures, or algorithms to solve problems, making structured decisions based on given premises, or deriving conclusions through systematic steps. This includes tasks ranging from basic arithmetic operations to sophisticated multi-step problem solving that requires careful chaining of logical relationships. Each level increases in complexity based on the intricacy of logical constructs, the depth of reasoning required, the number of premises involved, and the abstraction of concepts.

Levels

Level 0 **None.** Tasks at this level do not require any logical reasoning. They involve simple identification or recalling memorized facts without the application of rules or relationships, as no logical constructs or premises are present. **Examples**:

- Naming the capital city of a country without considering any relationships (e.g., “What is the capital of Austria?”).
- A pure knowledge question like “Which country won the football world cup in 2008?”
- “Match this circle to another circle.”

Level 1 **Very low.** Tasks require recognition of basic logical relationships and straightforward deductions from clearly stated premises. They may involve matching objects based on a single criterion or recognizing a direct implication without any complex logical operators, quantifiers, or negations. **Examples**:

- Simple sequential logic without requiring complex reasoning (e.g., “If today is Monday, what day will it be tomorrow?”)
- “When the light is red, stop. The light is red. What should you do?”
- “Compute the sample standard deviation of $\{-12, 51, 21, -9, -8, -7\}$.”

Level 2 **Low.** Tasks involve simple sequential reasoning or basic syllogistic reasoning with clear premises. Logical statements may include basic quantifiers such as “all” or “some” but involve direct relationships without a complex integration of multiple premises. **Examples**:

- Identifying characteristics within a biological classification (e.g., “If all mammals are warm-blooded and all whales are mammals, then all whales are warm-blooded.”)
- Evaluating logical inferences from categorical statements (e.g., “If some books are fiction and all fiction books are interesting, can we conclude that some books are interesting?”)
- In the equations below, $b = 2.35 + 0.25$ x and $c = 1.75 + 0.40x$ represent the price per pound, in dollars, of beef and chicken, respectively, x weeks after July 1 during last summer. What was the price per pound of beef when it was equal to the price per pound of chicken?

Level 3 **Intermediate.** Tasks involve multi-step logical deductions that require integrating multiple statements and the use of simple negations and varied quantifiers. Tasks may include combinations of different types of logical statements, necessitating the linking of several ideas to reach a conclusion. **Examples**:

- “If no reptiles have fur, all snakes are reptiles, and some pets are snakes, then some pets do not have fur.”
- “Solve this puzzle that requires linking multiple premises: In a group of people, if everyone wearing red shirts is older than 30 and no one older than 30 likes ice cream, can a person wearing a red shirt like ice cream?”
- Find the characteristic polynomial of the following matrix: $\begin{pmatrix} -\frac{61}{7} & -\frac{41}{7} & -\frac{87}{7} \\ -\frac{83}{7} & \frac{93}{7} & -6 \\ \frac{23}{7} & -\frac{26}{7} & \frac{95}{7} \end{pmatrix}$

Level 4 **High.** Tasks involve complex chains of logical deductions, using advanced logical constructs such as conditionals (“if-then”), biconditionals (“if and only if”), and multiple quantifiers. They require synthesizing numerous premises with intricate relationships to navigate through the task and reach a valid conclusion. **Examples**:

- “If all A are B, some B are C, no C are D, and all D are E, what can be inferred about the relationship between A and E?”
- "If all people at the party are over 18, some party attendees are graduate students, all graduate students have completed college, some college students are under 18, and no one under 21 can serve on the party planning committee, what can we determine about whether all graduate students at the party are eligible to be planners?"
- “A music producer is recording 7 albums one after another: F, G, H, J, K, L, and M, but it is not necessary to record them in this order. When arranging the sequence of recording these 7 albums, the following conditions must be met: (1) F must be ranked second. (2) J cannot be ranked seventh. (3) G can neither be directly in front of H nor immediately after H. (4) H must be somewhere in front of L. (5) L must be somewhere before M. Question: Which of the following can be the order of recording these 7 records from 1 to 7? Choices: A. F, K, G, L, H, J, M. B. G, F, H, K, L, J, M. C. G, F, H, K, L, M, J. D. K, F, G, H, J, L, M.”

Level 5+ **Very High.** Tasks require abstract and complex logical reasoning involving intricate deductive chains and advanced logical structures, such as nested conditionals and multiple levels of negation. They require deep analytical thinking and the ability to handle multiple premises with subtle interactions in order to evaluate sophisticated hypotheses or identify inconsistencies and fallacies. **Examples**:

- “Solving this advanced logic puzzle involving the manipulation of several interrelated statements: In a group of five people, if each person knows exactly two others and no one knows the same two people, how can the relationships be arranged?”
- “In a voting system with three committees (A, B, C), where each committee must have at least four members, a proposition is valid if and only if it satisfies one of these two conditions: either (1) it receives support from all members of at least two committees — except when there exists a member who serves on all three committees and chooses to abstain, in which case validity requires support from every member of exactly two committees, none of whom has ever voted the same way as any currently abstaining member on any past proposition — or (2) for any committee that unanimously opposes the proposition, there must exist exactly two members from each of the other committees who both (a) currently support the proposition and (b) have cast different votes from each other on every past proposition they both voted on, with the additional requirement that no supporting member has ever voted the same way as any current abstaining member on any proposition that achieved supermajority approval. Given a specific set of committee memberships, their complete voting histories, and their votes on a current proposition, determine whether the proposition is valid according to these rules.”
- “A convex 2019-gon $A_1A_2 \ldots A_{2019}$ is cut into smaller pieces along its 2019 diagonals of the form A_iA_{i+3} for $1 \leq i \leq 2019$, where $A_{2020} = A_1$, $A_{2021} = A_2$, $A_{2022} = A_3$. What is the least possible number of resulting pieces?”

Spatial Reasoning and Navigation (SN)

R1. Spatio-physical Reasoning (SNs)

This rubric assesses the complexity of spatial and physical understanding and reasoning required by a task. More specifically, the level represents the extent to which the task requires understanding spatial relationships between objects and predicting physical interactions, ranging from simple recognition of static relationships to complex mental manipulations involving multiple objects, transformations, and physical predictions across different dimensions. Notably, this should focus on the minimum way of solving the task since many tasks do not actually require spatio-physical reasoning to be successfully solved, even though spatio-physical reasoning may help.

Levels

Level 0 **None.** Tasks at this level do not require spatial reasoning or physical intuition. There is no need to manipulate or visualize spatial relationships. **Examples**:

- Copying text from one document to another without considering the layout.
- Transcribing a sentence such as "The book is on the table" from an audio recording without needing to interpret or visualize the spatial reference.
- Reciting a previously memorized sequence of numbers without spatial meaning.

Level 1 **Very low.** Tasks involve simple recognition of spatial relationships but no mental manipulation is required for a successful task completion. Objects and their relationships are static and visible. **Examples**:

- Identifying which shape matches a given template without rotating or manipulating it.
- Recognizing whether a flat plate on the top of a book is stable or likely to remain balanced in its current position.
- A convex 2019-gon $A_1A_2\ldots A_{2019}$ is cut into smaller pieces along its 2019 diagonals of the form A_iA_{i+3} for $1 \leq i \leq 2019$, where $A_{2020} = A_1$, $A_{2021} = A_2$, and $A_{2022} = A_3$. What is the least possible number of resulting pieces?

Level 2 **Low.** Tasks require basic common-sense predictions about physical interactions, or combined with simple spatial manipulations. The transformations are straightforward and involve only one or two objects. **Examples**:

- Estimating whether a box will fit through a door based on a visual comparison of dimensions, where the difference in size between the box and the door is fairly noticeable.
- Predicting the outcome of a very light ball (e.g., a table tennis ball) rolling into a much heavier, stationary object (e.g., a bowling ball). The task requires estimating the motion of the stationary heavy object, which will hardly move due to the significant difference in mass between the two objects.
- Predicting whether a piece of soft clay will flatten when being pushed.

Level 3 **Intermediate.** Tasks require coordinating multiple spatial relationships and performing sequential transformations. Mental models need to be constructed, involving intermediate levels of both physical prediction and spatial reasoning (3D reasoning or multiple steps in a 2D space), but focussing on the spatial. **Examples**:

- Given a scenario where a glass vase is knocked off a table, hits the edge of a wooden chair, and then falls to the floor, predict the outcome in terms of break or survive.
- Mentally visualize the trajectory of a thrown ball that arcs in the air and predict where it will land, accounting for a simple curve and the presence of a mild wind.
- Interpreting a sequence of spatial instructions (e.g., "Walk past the bridge, turn right after the post office, then take the second left") and constructing a mental model of the path based on multiple spatial references.

Level 4 **High.** Tasks involve complex spatial transformations and integration of multiple spatial operations. Requires construction and manipulation of mental models, often involving prediction of outcomes of non-trivial spatial transformations. **Examples**:

- Predicting how shadows will change as the position of a light source moves around a 3D object, requiring an understanding of light, geometry, and perspective.
- A passage describes a football game where multiple players are moving relative to one another: "John passed the ball to Mark, who was standing to the left of him, but then Sarah ran past both of them, taking the ball from the right side." The model must maintain spatial continuity by updating the players' relative positions and summarizing their final locations after each described action.
- In a detective novel, a room's layout is described as follows: "The safe is hidden behind the large painting on the wall, to the right of the door. Opposite the painting is a window, and next to the window is a desk." The subject must interpret and visualize the spatial arrangement of objects within the room and answer questions about their relative positions (e.g., "Where is the safe in relation to the desk?").

Level 5+ **Very High.** Tasks require very advanced spatial reasoning involving multiple simultaneous transformations and the prediction of highly complex spatial outcomes. These tasks involve predicting intricate chains of physical interactions and visualizing sophisticated spatial relationships that evolve over time or through multiple steps. **Examples**:

- Writing or interpreting technical instructions for assembling a complex mechanical device, requiring precise descriptions of spatial relationships and the correct sequence of assembly steps, often involving 3D reasoning about how parts fit together.
- A passage describes a complex laser setup: "The initial laser beam enters horizontally through a beam splitter that divides it into two identical beams. The first beam reflects off three mirrors - one at 30 degrees on the wall, another mounted 15 degrees from the ceiling, and a third on the floor at 45 degrees. The second beam reflects off two mirrors - one perpendicular to the ground and another at 60 degrees on the opposite wall." The subject must trace both beams' paths, predict their final directions and positions after all the reflections, and determine whether the beams will intersect at any point in their paths.
- A scientific article describes the structure of a protein: "The alpha-helix folds back on itself, forming hydrogen bonds with the beta-sheet that runs parallel, while the N-terminal aligns perpendicularly to the hydrophobic core." The task requires visualizing the 3D configuration of the protein and explaining the spatial relationships between the molecular elements described.

2.2. Knowledge

The Knowledge dimensions in our framework are designed to capture the domain-specific information or conceptual understanding required to respond successfully to a particular task. Unlike the 'elemental' dimensions—which reflect innate cognitive processes such as attention, language comprehension or reasoning—the knowledge scales measure the extent to which a task requires specialised information. Their levels are calibrated against the typical progression of formal education. In this way, they approximate the stage of knowledge development that a human student might experience: from the basic facts and simple concepts taught in primary school to the highly complex, specialised information that is generally only acquired at graduate or research level.

The following subsections describe each *knowledge* rubric in more detail.

Domain Knowledge (KN)

R1. Natural Sciences (KNn)

This rubric assesses the conceptual sophistication level of tasks based solely on the depth of knowledge or conceptual understanding required in the fields of natural sciences (e.g., physics, chemistry, biology, astronomy, earth sciences, ecology). This does not include social sciences and humanities (e.g., history, psychology, sociology, anthropology, literature, art, philosophy, linguistics) or formal sciences (e.g., mathematics, logic, computer science, statistics). It's important to note that this rubric focuses exclusively on the domain-specific knowledge needed, not considering other cognitive demands such as reasoning or metacognition. This reflects the conceptual depth and specificity of the knowledge in natural sciences required, rather than the mere presence of scientific content.

Levels

Level 0 **None.** Tasks do not require any knowledge of natural sciences. **Examples**:

- "Write a python script to train a machine learning classifier for fake news detection."
- "Analyze the symbolism in Shakespeare's Hamlet".
- "Calculate the cost of groceries."

Level 1 **Very low.** Tasks that require knowledge in natural sciences typically acquired through elementary school education. **Examples**:

- Living things need food, water, and air to survive.
- Basic parts of a plant (roots, stem, leaves).
- Day and night cycle and seasons.

Level 2 **Low.** Tasks that require knowledge in natural sciences typically acquired through middle school education. **Examples**:

- The water cycle (evaporation, condensation, precipitation).
- Basic cellular structure (nucleus, membrane, cytoplasm).
- Simple food chains and ecosystems.

Level 3 **Intermediate.** Tasks that require knowledge in natural sciences typically acquired through high school education. **Examples:**

- Mendel's laws of inheritance and basic genetics.
- The ideal gas law (PV = nRT).
- Newton's three laws of motion.

Level 4 **High.** Tasks that require knowledge in natural sciences typically acquired through undergraduate education. **Examples**:

- Hardy-Weinberg equilibrium and population genetics.
- Molecular orbital theory.
- The process of cellular respiration and its relationship to photosynthesis.

Level 5+ **Very High.** Tasks that require knowledge in natural sciences typically acquired through graduate education or beyond. **Examples**:

- The theoretical frameworks of string theory and its implications.
- The six forms of quark flavors in particle physics.
- The role of quantum entanglement in biological systems.

R2. Social Sciences and Humanities (KNs)

The following rubric is designed to annotate the conceptual sophistication level of tasks based exclusively on the depth of knowledge or conceptual understanding required in the fields of social sciences and humanities (e.g., history, psychology, sociology, anthropology, literature, art, philosophy, linguistics). This does not include natural sciences (e.g., physics, chemistry, biology, astronomy, earth sciences, ecology) and formal sciences (e.g., mathematics, logic, computer science, statistics). It's important to note that this rubric focuses exclusively on the domain-specific knowledge needed, not considering other cognitive demands such as reasoning or metacognition. This reflects purely the depth of social sciences and humanities knowledge required.

Levels

Level 0 **None.** Tasks do not require any knowledge or understanding of social sciences or humanities. **Examples**:

- “Calculating the area of a rectangle with length 5 cm and width 3 cm.”
- “Explaining the process of photosynthesis in plants.”
- “Explaining the mathematical principles behind differential calculus.”

Level 1 **Very low.** Tasks that require knowledge in social sciences and humanities typically acquired through elementary school education. **Examples**:

- Basic concepts of past, present, and future in history.
- Different types of communities (family, school, neighborhood).
- Traditional holidays and their basic meanings.

Level 2 **Low.** Tasks that require knowledge in social sciences and humanities typically acquired through middle school education. **Examples**:

- Basic historical periods like “ancient” vs “modern” times.
- Different types of government (democracy, monarchy).
- Major world religions and their basic beliefs.

Level 3 **Intermediate.** Tasks that require knowledge in social sciences and humanities typically acquired through high school education. **Examples**:

- The role of the Silk Road in cultural exchange.
- Basic principles of cognitive psychology.
- Major literary movements (Romanticism, Realism).

Level 4 **High.** Tasks that require knowledge in social sciences and humanities typically acquired through undergraduate education. **Examples**:

- The socio-economic factors that led to the Industrial Revolution.
- Major sociological theories of social stratification.
- The main theoretical approaches in anthropology.

Level 5+ **Very High.** Tasks that require knowledge in social sciences and humanities typically acquired through graduate education or beyond. **Examples**:

- The major schools of Sanskrit poetics.
- The primary theoretical frameworks in phenomenology.
- Advanced theories in historical linguistics and their implications.

R3. Formal Sciences (KNf)

The following rubric is designed to annotate the conceptual sophistication level of tasks based strictly on the depth of knowledge or conceptual understanding required in the fields of formal sciences (e.g., mathematics, logic, computer science, statistics). This does not include the natural sciences (e.g., physics, chemistry, biology, astronomy, earth sciences, ecology) or the social sciences and humanities (e.g., history, psychology, sociology, anthropology, literature, art, philosophy, linguistics). It's crucial to understand that this rubric measures only the level of formal scientific knowledge needed, not considering other cognitive demands such as reasoning or metacognition. This indicates solely the depth of formal sciences knowledge required.

Levels

Level 0 **None.** Tasks require no knowledge or understanding of formal sciences. **Examples**:

- "Explaining the biological mechanisms of cellular respiration."
- "Describing the major artistic movements of the Renaissance period."
- "Explaining the rules of a sport."

Level 1 **Very low.** Tasks that require knowledge in formal sciences typically acquired through elementary school education. **Examples**:

- Basic arithmetic operations (+, -, ×, ÷).
- Names and properties of basic shapes (square, circle, triangle).
- Understanding that a programming loop with 10 repetitions takes double time than a loop with 5 repetitions.

Level 2 **Low.** Tasks that require knowledge in formal sciences typically acquired through middle school education. **Examples**:

- Basic algebraic expressions and variables.
- Calculating mean, median, and mode.
- Properties of basic number systems (integers, decimals, fractions).

Level 3 **Intermediate.** Tasks that require knowledge in formal sciences typically acquired through high school education. **Examples**:

- Basic geometric shapes and their properties.
- What an algorithm is.
- Fundamental concepts of logic and syllogisms.

Level 4 **High.** Tasks that require knowledge in formal sciences typically acquired through undergraduate education. **Examples**:

- The fundamental theorem of calculus.
- Principles of object-oriented programming.
- Basic concepts in linear algebra and matrix operations.

Level 5+ **Very High.** Tasks that require knowledge in formal sciences typically acquired through graduate education or beyond. **Examples**:

- Principles of homological algebra.
- Mathematical foundations of quantum computing.
- Advanced concepts in cryptography and their mathematical basis.

R4. Applied Sciences and Professions (KNa)

The following rubric is designed to annotate the conceptual sophistication level of tasks based entirely on the depth of knowledge or conceptual understanding required in the fields of applied sciences and professions (e.g., medicine, law, education, business, agriculture, engineering except software and data engineering). Noteworthy, this rubric only focuses on applied knowledge and practical implementations rather than purely theoretical frameworks or abstract concepts from natural sciences, formal sciences, social sciences and humanities. For instance, understanding chemical reactions is only part of natural sciences, but applying this knowledge in pharmaceutical manufacturing would fall under applied sciences. Similarly, economic theory belongs only to social sciences, but practical business management and operations fall under applied sciences and professions. The focus is exclusively on the level of domain-specific knowledge needed, disregarding other cognitive demands such as reasoning or metacognition. This reflects only the depth of applied sciences and professional knowledge required.

Levels

Level 0 **None.** Tasks requiring no knowledge or understanding of applied sciences or professional fields. **Examples**:

- "Let $\triangle ABC$ be an acute triangle, with M being the midpoint of $\overline{BC}$, such that $AM = BC$. Let D and E be the intersection of the internal angle bisectors of $\angle AMB$ and $\angle AMC$ with AB and AC, respectively. Find the ratio of the area of $\triangle DME$ to the area of $\triangle ABC$."
- "In a chemical reaction at pH 1, an unknown substance was added that changed the pH to 4 and slowed down the reaction. What could have caused this?"
- "If a star 20 light-years away explodes, would gravitational waves reach Earth faster than light?"

Level 1 **Very low.** Tasks that require knowledge in applied sciences and professions typically acquired through elementary school education. **Examples**:

- Basic personal hygiene and hand washing procedures.
- Common road signs and traffic signals.
- Basic safety rules at home and school.

Level 2 **Low.** Tasks that require knowledge in applied sciences and professions typically acquired through middle school education. **Examples**:

- The use of basic measurement tools (thermometer, ruler, scale).
- Basic principles of personal finance and saving.
- Simple first aid for minor injuries.

Level 3 **Intermediate.** Tasks that require knowledge in applied sciences and professions typically acquired through high school education. **Examples**:

- Common legal terms (plaintiff, defendant, contract).
- Basic business concepts (budget, profit, loss).
- Fundamental principles of agricultural science.

Level 4 **High.** Tasks that require knowledge in applied sciences and professions typically acquired through undergraduate education. **Examples**:

- Basic principles of bridge design in civil engineering.
- Core concepts of supply chain management.
- Fundamentals of clinical assessment in healthcare.

Level 5+ **Very High.** Tasks that require knowledge in applied sciences and professions typically acquired through graduate education or beyond. **Examples**:

- Gene therapy techniques in precision medicine.
- Engineering principles of nuclear fusion reactor design.
- Legal frameworks for regulating artificial intelligence systems.

R5. Customary Everyday Knowledge (KNc)

The following rubric is designed to annotate the conceptual sophistication level of tasks based solely on the depth of customary everyday knowledge required. This knowledge encompasses information that most people in a given society typically acquire through daily life experiences, social interactions, and exposure to popular media, rather than through formal education or specialized training. This does not include specialized knowledge from the natural sciences, social sciences, humanities, formal sciences, or applied sciences and professions. This reflects only the depth of customary everyday knowledge required.

Levels

Level 0 **None.** Tasks do not require any customary everyday knowledge. **Examples**:

- Looking in a mirror and checking if there's any dirt in your face.
- Recognizing that two objects are of the same color.
- Basic arithmetic calculations.

Level 1 **Very low.** Tasks that require basic knowledge universally shared within a society. **Examples**:

- Knowing that "10h" may refer to the morning or to the evening.
- A brownie is better served as a dessert than an appetiser.
- Common objects in daily life (chairs, tables, cars).

Level 2 **Low.** Tasks that require customary everyday knowledge typically possessed by most adults in a society. **Examples**:

- Basic kitchen tools and their uses.
- Common traffic signs and their meanings.
- Major holidays in one's culture.

Level 3 **Intermediate.** Tasks that require general knowledge typically possessed by socially engaged members of society. **Examples**:

- Different types of payment methods (cash, credit cards, digital wallets).
- Common technology features (touch screens, wireless connectivity, cloud storage).
- Standard retail practices (return policies, warranties, seasonal sales).

Level 4 **High.** Tasks that require extensive everyday knowledge gained through active engagement in society. **Examples**:

- Major generational trends in technology adoption (from landlines to smartphones).
- Common real estate concepts (mortgages, leases, property taxes).
- Dietary restrictions across different groups (religious, health-based, ethical).

Level 5+ **Very High.** Tasks that require comprehensive everyday knowledge across diverse cultural and social contexts. **Examples**:

- Gift-giving customs and taboos across different cultures.
- Business etiquette variations in major world regions.
- Dining customs and table manners in different societies.

2.3. Extraneous

These dimensions do not reflect cognitive capabilities per se, but rather aspects of item design that may affect observed performance such as presentation and design. They are critical because item design can artificially inflate or obscure performance scores. For example, if an item is unusually long, a model may perform poorly not because it lacks the relevant skill, but simply because it is overwhelmed by the amount of text. Similarly, if an item is highly prototypical or has been seen repeatedly in the training data, memorisation rather than genuine reasoning may lead to success. Finally, if a question is structured in such a way that a correct answer can be obtained by chance (for example, in a multiple-choice format), this introduces guessability.

The following subsections describe each *Extraneous* rubric in more detail.

Volume (VO)

This rubric defines a scale that evaluates the task purely based on its *volume*, i.e., the time a fully competent, experienced and motivated human would need to both read and complete the task in ideal conditions, not counting breaks or interruptions, regardless of the difficulty or cognitive demands of the task. The scale ranges from tasks requiring less than a second to those requiring more than 16 hours (1000 minutes), focusing purely on the volume of work rather than on cognitive complexity or skill requirements. Time estimates assume the task performer has all necessary information, tools, and skills readily available, but works autonomously without the assistance of other humans or AI tools.

Levels

Level 0 **None.** Volume: Negligible, requiring less than 1 second. **Examples**:

- Checking the status of an indicator (e.g., a light on a machine).
- Selecting a checkbox to confirm agreement.
- Opening a pre-configured app or program.

Level 1 **Very Low.** Requiring between 1 second and 1 minute. **Examples**:

- Read a short online comment about merchandise to identify whether it has positive, neutral, or negative emotion.
- Saving and uploading a single document to a pre-arranged folder.
- Reading a short email and writing a brief confirmation email (e.g., confirming attendance to a meeting).

Level 2 **Low.** Requiring between 1 minute and 10 minutes. **Examples**:

- Writing a simple summary in half a page of the main points from a short memo or meeting.
- Listening to an audio recording of one minute from a high-school level history class and answering a list of ten short factual questions.
- Reading an inquiry and writing a few paragraphs long personalized email in response to an inquiry.

Level 3 **Intermediate.** Requiring between 10 minutes and 100 minutes. **Examples**:

- Proofreading and lightly editing a 4-page research article to improve flow and clarity.
- Organizing and cataloguing a personal collection of 50 books by genre, year, and author.
- Reading a set of instructions and data based on a small experiment, and then writing a 500-word report based on it.

Level 4 **High.** Requiring between 100 minutes (roughly 1.5 hours) and 1,000 minutes (about 16 hours). **Examples**:

- Creating a 10-page technical manual including screenshots, step-by-step procedures, and troubleshooting guides for a specific software application.
- Configuring 25 workstations with standardized software (5 applications per machine), security settings, and network access protocols.
- Reading a 12-page position paper on language models' impact on education and verbally summarizing the paper's key insights for colleagues.

Level 5+ **Very High.** Requiring more than 1,000 minutes (roughly 16 hours). **Examples**:

- Planning and executing a 3-day workshop for 100 attendees, including scheduling 15 speakers and arranging catering for 6 meals.
- Writing a high-quality 10-page research paper on the field of data science, requiring analysis of 50+ academic sources, including data visualization and statistical analysis of 3 datasets.
- Reviewing the correctness of 2000+ simple geography exercises written by high-school students.
- Conducting a financial audit covering 12 months of transactions (approximately 5,000 entries) across 5 department budgets, including reconciliation and variance analysis.

Atypicality (AT)

This rubric defines a scale that evaluates tasks based on how unlikely they appear in various sources (internet, textbooks, tests) and how unlikely the specific instance is to have been previously encountered and memorized. The scale ranges from exactly identical instances that are widely known to completely novel task formulations, focusing on the uniqueness of both the task type and the specific instance rather than its difficulty or complexity.

Levels

Level 0 **None.** The task is a staple one. Exactly the same instance of the task appears many times on the Internet, textbooks or common psychological or achievement tests, and the solution is generally well-known and memorized. **Examples**:

- "What is 2 + 2?"
- "Name the capital of France."
- "What gets wetter and wetter the more it dries?"

Level 1 **Very low.** The task is very common and the specific task instance is likely to frequently appear on the Internet, textbooks or common psychological or achievement tests, so the chance that the solution is well-known and memorized is high. **Examples**:

- "What is the derivative of sin(x)?"
- "Define opportunity cost."
- "Name the seven continents."

Level 2 **Low.** The task is moderately common and the specific task instance varies somewhat from other common examples or is unlikely to have seen it before in exactly the same form, but possibly in variations. **Examples**:

- "What is 21251 + 2835?"
- "Given the molecular SMILES: `COC[C@H]1OC(=O)c2coc3c2[C@@]1(C)C1=C(C3=O)[C@@H]2CCC(=O)[C@@]2(C)C[C@H]1OC(C)=O`, your task is to provide the detailed description of the molecule using your experienced chemical Molecular knowledge."
- "Solve the following Math Olympiad question: Determine the greatest real number C, such that for every positive integer $n \geq 2$, there exists $x_1, x_2, \ldots, x_n \in [-1, 1]$ so that $\prod_{1\leq i<j\leq n}(x_i - x_j) \geq C^{\frac{n(n-1)}{2}}$."

Level 3 **Intermediate.** The task is somewhat common but the specific task instance is quite rare, and it is unlikely to appear in common sources (Internet, textbooks or common psychological or achievement tests). **Examples:**

- "What is 5205175017521571 + 68270867426872052?"
- "Among the following exoplanets, which one has the lowest density? a) An Earth-mass and Earth-radius planet. b) A planet with 3 Earth masses and a density of approximately 4.6 g/cm^3. c) A planet with the same composition as Earth but 1.5 times more massive than Earth. d) A planet with the same composition as Earth but half the mass of Earth."
- "Get answers for the question based on the context, where answers derived from substrings in the context or categorized as [unanswerable]. Context: ['On May 1 , 2015 , Quentin announced his retirement .Quentin signed a minor league deal with the Seattle Mariners on April 22 , 2015 , and was assigned to the Tacoma Rainiers .On April 5 , 2015 , Quentin was traded to the Atlanta Braves along with Cameron Maybin , Matt Wisler , and Jordan Paroubeck , for Craig Kimbrel and Melvin Upton Jr . The Braves designated him for assignment later that day , and released him on April 14 .', 'On July 22 , 2012 , Quentin agreed to a three-year , $27 million contract extension through 2015 with a $10 million mutual option for 2016 , including a no-trade clause . This is an amazing opportunity to stay and play in the city I grew up in . said Quentin .'] Question: Who did Carlos Quentin work for in April 2016?"

Level 4 **High.** The task is not extremely uncommon and the specific task instance is infrequent or presented in notably different ways from standard formulations. **Examples**:

- "Create a measurement system where accuracy is expressed through different shapes rather than decimal places."
- "Assume that there exist only two types of people: knights and knaves. Knights always tell the truth, while knaves always lie. You are given the statements from 5 characters. Based on their statements, infer who is a knight and who is a knave. A: B is a liar and D is a truth-teller. B: D is a truth-teller. C: If A is a truth-teller, then E is a liar. D: B is a truth-teller and A is a truth-teller. E: A is a liar."
- "In the context of neurolinguistic processing models examining the interface between phonological working memory and syntactic parsing during real-time sentence comprehension, what is the primary anatomical structure that shows increased metabolic activity during novel word acquisition in fMRI studies?" [This is a very sophisticated way of asking "which part of the brain lights up when we learn new words?"

Level 5+ **Very High.**The task is fundamentally different from those typically appearing on the Internet, textbooks or psychological or achievement tests, or, the specific task instance is very unlikely to have close analogues in those sources. Noteworthy, any tasks (simple or elaborate) that can be found in standard tests or benchmarks should be considered less than level 5. **Examples**:

- "Take 20 major sky constellations and design 20 Formula1 race circuits that follow the FIA regulations but mimic the shapes of the constellations."
- "Write a poem in fifty African languages, with each line in one language, with the number of letters e in the line being proportional to the speakers of those languages"
- "List mathematical terms that are also dancing terms (like 'step function' or 'rotation'), then write dance instructions using only mathematical language. However, the dance must stem from an Asian country since I'm teaching a creative Asian dance course today to one student who happens to be a mathematician."

Unguessability (UG)

This rubric classifies questions based on their answer format, determining whether they are multiple-choice (explicit or implicit) or open-ended. The output is either an integer representing the number of choices (for multiple-choice questions) or the word “open” (for open-ended questions).

Answer Format Classification Prompt

QUESTION: {Question}
REFERENCE ANSWER: {Answer}

You are tasked with analyzing the question and its reference answer above, and classifying them based on their answer format. To this end, you need to determine if it’s multiple-choice (explicit or implicit) or open-ended. Output a single integer representing the number of possible choices in an open-ended question, or "open" for an open-ended question.

Classification Rules

1. **Explicit Multiple Choice Questions. Examples**:
   - “Which color is best: Red, Blue or Green?” → Output: 3
   - “Choose from: A) Earth B) Mars C) Venus D) Jupiter” → Output: 4
   - “Which of these explanations best describes photosynthesis? [followed by 4 detailed explanations]” → Output: 4
2. **Implicit Multiple Choice Questions (ONLY for well-known or given sets).**
   - Yes/No Questions → Output: 2
   - True/False questions → Output: 2
   - Questions using well-known or given sets. Examples include, but not limited to:
     - Days of the week → Output: 7
     - Months of the year → Output: 12
     - Continents → Output: 7
     - Cardinal directions (N/S/E/W) → Output: 4

   Examples:
   - “What day of the week does the event start?” → Output: 7
   - “Which season is warmest?” → Output: 4
   - “Is this statement correct?” → Output: 2
3. **Open-ended Questions.**
   - Questions requiring free-form responses. **Example**:
     - "Explain why the sky is blue." → Output: open
   - Questions with no explicit options provided. **Example**:
     - “What factors contributed to the Industrial Revolution?” → Output: open
   - Questions where options must be discovered or deduced as part of solving the problem. **Example**:
     - “What city with over 1M inhabitants is furthest east in Spain?” → Output: open
   - Questions with finite but non-obvious sets of answers. **Example**:
     - “Which company will become the most valuable by 2030?” → Output: open

Instructions for Processing

1. Read the question carefully.
2. Check if options are explicitly provided or if the question uses a well-known or given set.
3. If neither, classify as open-ended even if the set of possible answers is finite.
4. Output ONLY the number or "open" without any explanation.

Note: Only classify as multiple-choice if:
- The options are explicitly listed in the question, OR
- The options come from a fine (well-known or given) set like days of the week.

Format your output as a single line containing either:
- An integer (for multiple-choice questions) and nothing else.
- The word "open" (for open-ended questions) and nothing else.

Output:

3. Rubrics for Baseline Taxonomies

3.1. Miller & Tang

R1. Summarization (RWs)

This rubric evaluates whether a task requires analyzing large amounts of content to extract key information and present concise summaries. This may involve objective summarization (e.g., extracting measurable data trends) or subjective summarization (e.g., condensing viewpoints or interpretations).

Levels

Level 0 **None.** The task does not require summarizing, condensing, or extracting key information from larger content. No analysis of extensive material is needed to present concise overviews. **Examples**:

- “What is the capital of France?”
- “Calculate 15 + 27.”
- “Translate ‘hello’ into Spanish.”

Level 1 **Present.** The task requires analyzing large amounts of content to extract key information and present concise summaries. This includes condensing lengthy documents, extracting main arguments, identifying key data trends, or providing overviews of complex topics. **Examples**:

- “Summarize the author’s main arguments in the web pages.”
- “Calculate price trends from this quarterly sales dataset.”
- “Provide a brief overview of key findings from this 50-page research report.”

R2. Technical Assistance (RWt)

This rubric evaluates whether a task requires providing clear, actionable instructions to diagnose, fix, or implement solutions in technical systems (software, hardware, code, or configuration). This capability requires problem-solving and procedural reasoning within technical domains.

Levels

Level 0 **None.** The task does not require technical problem-solving, troubleshooting, or step-by-step technical guidance. No diagnosis or procedural instruction is needed. **Examples**:

- “Write a poem about nature.”
- “What are the benefits of exercise?”
- “Explain what cloud computing is.”

Level 1 **Present.** The task requires providing clear technical instructions, diagnosing technical issues, or suggesting procedural solutions for software and hardware problems. This includes coding assistance, troubleshooting, and configuration guidance. **Examples**:

- “Write optimized code to handle a leaderboard.”
- “Debug this Python script that’s throwing an error.”
- “How do I set up a database connection in Node.js?”

R3. Reviewing Work (RWr)

This rubric evaluates whether a task requires identifying issues, evaluating systems or processes, and recommending improvements or solutions. It can involve both objective evaluation (e.g., correctness) and subjective assessment (e.g., tone or effectiveness).

Levels

Level 0 **None.** The task does not require evaluating, reviewing, or providing feedback on existing work, systems, or processes. No assessment of quality, correctness, or improvement suggestions is needed. **Examples**:

- “Generate a list of vacation destinations.”
- “What is machine learning?”
- “Write a business proposal.”

Level 1 **Present.** The task requires identifying issues, evaluating systems or processes, and recommending improvements or solutions. This includes proofreading, quality assessment, performance evaluation, and providing constructive feedback. **Examples**:

- “Correct math homework answers.”
- “Review my email for tone and clarity and suggest improvements.”
- “Analyze this business proposal and identify potential weaknesses.”

R4. Data Structuring (RWd)

This rubric evaluates whether a task requires efficiently managing, logging, and maintaining accurate records of transactions, interactions, or documentation. It focuses on organizing and formatting information systematically.

Levels

Level 0 **None.** The task does not require organizing, structuring, formatting, or managing information systematically. No record-keeping or structured data management is needed. **Examples**:

- "Tell me a joke."
- "What is the meaning of democracy?"
- "Generate creative story ideas."

Level 1 **Present.** The task requires organizing or formatting information systematically, such as logging, record-keeping, or converting unstructured content into structured formats. **Examples**:

- "Change academic reference list from Chicago to APA style."
- "Organize this customer feedback data into categories."
- "Convert this unstructured text into a formatted table."

R5. Generation (RWg)

This rubric evaluates whether a task requires creating original ideas, drafting written content, or proposing creative solutions based on given parameters or goals. It measures generative capabilities beyond mere retrieval or restructuring.

Levels

Level 0 **None.** The task does not require generating original content or ideas. No creative or generative work is needed. **Examples**:

- "Define the term 'ecosystem.'"
- "What year did World War II end?"
- "Describe the process of photosynthesis."

Level 1 **Present.** The task requires generating original ideas, creating written content, or producing novel material based on goals or parameters. This includes ideation, writing, and creative problem-solving. **Examples**:

- "Create content for an About Us page for a small bakery."
- "Generate three innovative marketing strategies for a tech startup."
- "Write a short story about time travel."
- "Brainstorm unique solutions for reducing office energy consumption."

R6. Information Retrieval (RWi)

This rubric evaluates whether a task requires finding and delivering relevant factual or background information in response to queries, often based on pre-trained knowledge or retrieval-augmented generation (RAG). It focuses on accessing and providing existing information rather than generating new content.

Levels

Level 0 **None.** The task does not require retrieving or providing factual or background information. No lookup or factual access is needed. **Examples**:

- "Create a fictional backstory for Marie Curie."
- "Write a personal opinion about modern art."
- "Generate a random password."

Level 1 **Present.** The task requires finding and providing relevant factual or background information to specific queries. This includes accessing databases, retrieving facts, and supplying current or verified knowledge. **Examples**:

- "Looking at several supermarkets online, what is the current price of mangoes in Australia?"
- "Who won the Nobel Prize in Physics in 2023?"
- "What are the symptoms of Type 2 diabetes?"

Bloom's Taxonomy (Cognitive Domain)

Bloom's rubric analyses instructional goals and assessments of a given task item. The six levels below (Knowledge, Comprehension, Application, Analysis, Synthesis, Evaluation).

Levels

Level 1 **Knowledge.** Recall of specifics, universals, methods, processes, or settings; chiefly remembering and recognizing information. **Examples**:

- Multiple choice: Which phrase best defines the *period* of a wave? (1) maximum displacement; (2) time for one vibration; (3) vibrations per second; (4) time rate of change of distance.
- Which of the following is *not* payment for labor services? (commission; marriage fee; dividend; senator's salary).
- Best description of 18th-century colonial social structure (choose from four characterizations).

Level 2 **Comprehension.** Lowest level of understanding—grasping the meaning so the material can be used as communicated without deeper integration. **Examples**:

- Identify the philosophy expressed by the quotation about ideas as products of experience (positivism, rationalism, idealism, empiricism, pragmatism).
- Interpret Wordsworth's metaphor "she is a fen of stagnant waters" (progress, turmoil, stasis, corruption).
- From lines in Shakespeare's Sonnet XV, infer the claim about perfection and time (choose the closest paraphrase).

Level 3 **Application.** Use of abstractions (rules, procedures, principles, theories) in concrete situations. **Examples**:

- Predict the long-term state of a sealed aquarium and justify the reasoning.
- Judge policy items for their effect on income inequality (reduce / increase / none).
- Ball thrown upward in an elevator descending with acceleration g: choose the ball's motion relative to the car.

Level 4 **Analysis.** Break a communication into parts to reveal hierarchy and relations; clarify organization and logic. **Examples**:

- Galileo's inclined-plane study: identify an assumption implicit in extrapolating to free fall.
- Open-meeting speech (A–F): determine what statement A is offered as a reason for.
- Two-grade (S/U) proposal: the conclusion depends fundamentally on which proposition?

Level 5 **Synthesis.** Combine elements to form a new structure or pattern not clearly present before. **Examples**:

- Devise a short story connecting two given sentences via an obstacle you invent.
- Given facts on gases X and Y, evaluate hypotheses and select the best experiment to test each.

Level 6 **Evaluation.** Make judgments about value/quality using criteria and standards (quantitative or qualitative). **Examples**:

- Choose the most effective path toward more uniform divorce laws (among five policy mechanisms).
- For several business goods, judge whether consumer-media ads would reach enough prospects to justify cost.
- Write a brief evaluative essay on a poem, stating criteria and demonstrating recognition of formal features.

3.3. *Generic Difficulty*

Generic Difficulty (GD)

This rubric assesses the overall cognitive difficulty or demand of a task. It represents the overall difficulty level that emerges from the combination of cognitive processes (e.g., attention, comprehension, expression, reasoning, metacognition, social cognition, spatial reasoning) and knowledge requirements (e.g., natural sciences, social sciences, formal sciences, applied sciences, everyday knowledge). The level represents the total cognitive demand and expertise required to successfully complete a task, ranging from simple responses requiring minimal cognitive engagement to highly complex tasks demanding advanced domain-expertise, sustained attention, complex language, sophisticated reasoning processes, and/or sustained metacognitive effort, among other complex demands.

Levels

Level 1 **Very Low.** Minimal cognitive engagement; straightforward operations with immediately apparent solutions; basic attention; little to no specialized knowledge, reasoning, or metacognitive effort. **Examples**:

- "Given a single word input, determine if it starts with a capital letter."
- "What is the capital of France?"
- Stating immediate needs like "I need water" in a simple, direct, unambiguous way.

Level 2 **Low.** Basic cognitive operations beyond automatic responses; elementary reasoning; simple comprehension and expression; limited attention demands; small metacognitive requirements. Typically solved via direct application of basic principles or simple logical steps. **Examples**:

- Find all the vowels in "The quick brown fox jumps over the lazy dog."
- Answer simple "why" questions about a short story (e.g., why the girl took an umbrella after seeing dark clouds and grabbing it before leaving home).
- "If your friend has 7 apples and you have 5, who has more apples?"

Level 3 **Intermediate.** Moderate engagement; sustained attention to moderately complex information; comprehension of implicit meanings and relationships; organized expression; integration of reasoning processes; requires conceptual understanding and some metacognitive monitoring. **Examples**:

- "Find everyone wearing glasses in this casual BBQ photo with about 15 people, some sitting and some standing."
- Follow a high-school essay argument connecting industrialization, urbanization, living conditions, and public health to explain an "ironic" consequence.
- "If bacteria double every 30 minutes and you start with 100, how long to reach 10,000? Show your reasoning."

Level 4 **High.** Sophisticated processing: integrates advanced knowledge, complex reasoning chains, sustained metacognitive monitoring, and coordination of multiple processes; track multiple targets amid distractors; interpret multi-layered content; significant critical evaluation and self-awareness of reasoning limits. **Examples**:

- Track three orange spheres among twenty red ones as they move on a black screen (40 cm $\times$ 30 cm) at 1–3 cm/s, with intersecting paths and $\geq$2 cm separation; each sphere is 1 cm in diameter.
- Follow a multi-viewpoint fiction narrative, reconciling biased, partial accounts to reconstruct events.
- Evaluate a school's lunch-period extension proposal by weighing evidence, stakeholder impacts, and personal-bias effects.

Level 5 **Very High.** Mastery-level performance: exceptional attention and tracking; comprehension of highly convoluted, interconnected information; generation of multi-layered content; advanced logical structures; continuous high-level metacognition; expert knowledge integration, sophisticated social cognition, and complex spatio-temporal reasoning. **Examples**:

- Monitor four simultaneous airport-terminal video feeds to detect subtle changes (e.g., interactions < 2 s, crowd-flow shifts, small object exchanges).
- Comprehend a stream-of-consciousness modernist novel interweaving timelines, memories, and perspectives without clear demarcations.
- Analyze a proposed economic study on discrimination: interrogate causal assumptions, confounders, methods, conclusions, and your own biases.
